# MADRe: Strain-level metagenomic classification through assembly-driven database reduction

**DOI:** 10.1093/gigascience/giag030

**Published:** 2026-03-23

**Authors:** Josipa Lipovac, Mile Šikić, Riccardo Vicedomini, Krešimir Križanović

**Affiliations:** Laboratory for Bioinformatics and Computational Biology, Faculty of Electrical Engineering and Computing, University of Zagreb, 3 Unska Street, 10000, Zagreb, Croatia; Laboratory for Bioinformatics and Computational Biology, Faculty of Electrical Engineering and Computing, University of Zagreb, 3 Unska Street, 10000, Zagreb, Croatia; Laboratory of AI in Genomics, Genome Institute of Singapore, Agency for Science, Technology and Research (A*STAR), 60 Biopolis Street, Genome, 138672, Singapore, Singapore; Univ Rennes, CNRS, Inria, IRISA - UMR 6074, F-35000 Rennes, France; Laboratory for Bioinformatics and Computational Biology, Faculty of Electrical Engineering and Computing, University of Zagreb, 3 Unska Street, 10000, Zagreb, Croatia

**Keywords:** metagenomics, strain-level, metagenomic classification, database reduction

## Abstract

Strain-level metagenomic classification is essential for understanding microbial diversity and functional potential, yet remains challenging, particularly when sample composition is unknown and reference databases are large and redundant. Here, we present MADRe, a modular and scalable pipeline for long-read strain-level metagenomic classification based on **M**etagenome **A**ssembly-Driven **D**atabase **Re**duction. Beyond system-level integration, MADRe introduces statistical strategies that leverage assembly-derived genomic context to guide database reduction and probabilistic read reassignment. Specifically, it combines long-read metagenome assembly, contig-to-reference reassignment using an expectation–maximization framework for reference reduction, and probabilistic read mapping reassignment on a reduced database to achieve sensitive and precise strain-level classification. We extensively evaluated MADRe on simulated datasets, mock communities, and a real anaerobic digester sludge metagenome. Across diverse similarity and coverage conditions, MADRe consistently improves precision by reducing false-positive strain detections. MADRe’s design allows users to apply either the database reduction or read classification step individually. Using only the read classification step shows results on par with other tested tools. MADRe is open source and publicly available at https://github.com/lbcb-sci/MADRe.

Key Points

- MADRe implements a two-step strategy for strain-level classification: It first identifies candidate strains via assembly-to-database mapping with EM-based reassignment, then classifies reads by mapping to a reduced database via probabilistic reassignment.
- Despite incorporating assembly, MADRe significantly reduces runtime and memory usage compared to mapping all reads to the full reference database.
- MADRe enables the use of large, diverse reference databases without prior knowledge of sample content, focusing classification on confidently assembled strains and substantially reducing false positives while maintaining high strain-level resolution.

## Background

Metagenomics enables the study of genetic material from complex microbial communities found in environments such as human gut, soil, or marine ecosystems. It provides a comprehensive view of microbial diversity and interactions within these environments [1, 2]. Modern microbiome studies increasingly rely on modular and multi-omics analysis pipelines to integrate taxonomic and functional signals across data types [3]. Despite these advances, a central challenge in metagenomic analysis is the accurate identification of organisms present in a sample, typically performed by comparing sequencing reads to reference genome databases [4].

A wide range of metagenomic classification tools have been developed, which can be broadly categorized into marker-based, DNA-to-protein and DNA-to-DNA approaches, as described in [5]. Marker-based tools, such as MetaPhlAn [6, 7], StrainPhlAn, mOTUs [8], and Melon [9], classify taxa using conserved, clade-specific marker genes. In addition to marker-based methods, SNV-based profilers (e.g., metaSNV [10] and InStrain [11], which combines both approaches) represent important strategies for strain detection and population tracking. However, most of these approaches are optimized for short-read data and rely on predefined marker sets or variant catalogs, which may not fully capture genomic diversity in complex or underrepresented microbial communities. DNA-to-protein tools, including Kaiju [12], DIAMOND [13], MMseqs2 [14], and MEGAN-LR [15], translate reads into amino acid sequences before aligning them to protein databases. DNA-to-DNA tools compare reads directly against genomic sequences and are commonly divided into k-mer-based and mapping-based tools [16]. K-mer-based tools such as Kraken2 [17], KrakenUniq [18], Bracken [19], Centrifuge [20], Centrifuger [21], CLARK/CLARK-S [22, 23], Ganon [24, 25], Taxor [26], and Sylph [27] are known for their speed and scalability to large databases, but often trade precision for speed. In contrast, mapping-based tools such as MetaMaps [28], PathoScope2 [29, 30], EMU [31] and MORA [32], which rely on read alignments and reassignment algorithms, offer higher precision at a greater computational cost.

Although k-mer-based tools, especially Kraken2 or Sylph, perform well at the species level, strain-level classification becomes increasingly challenging when sequences originate from closely related genomes [33]. However, resolving strain-level diversity is essential, as even closely related strains can exhibit substantial differences in gene content and function, with implications for microbial ecology, pathogenesis, and treatment outcomes [34–39].

While most existing tools are optimized for short reads due to their low cost and high accuracy, long-read sequencing technologies such as Oxford Nanopore (ONT) and PacBio HiFi are rapidly improving. Longer read lengths provide advantages for genome assembly, structural-variant detection, and improved strain-level resolution.

Several short-read-based tools are designed for strain-level classification within a single species, such as StrainGE [40], StrainEST [41], and StrainSeeker [42], as well as the long-read-based ORI [43]. Other tools, including PanTax [5], MetaMaps [28], Centrifuge [20], Centrifuger [21], PathoScope2 [29, 30], and MORA [32], are suitable for more complex, multi-species datasets and support short- and long-read strain-level metagenomic classification. PanTax is a pangenome-based approach that, while supporting multi-species datasets, faces scalability limitations when applied to very large reference databases. MetaMaps is a mapping-based tool capable of high-resolution classification but is known to be extremely computationally demanding [16]. Centrifuge is a k-mer-based tool designed to perform strain-level classification, but like PanTax, it encounters limitations when constructing indexes for very large reference databases. However, its successor, Centrifuger, introduces improved compression and indexing strategies that enable efficient classification across large-scale genome databases. PathoScope2 is an older tool that is no longer maintained and cannot be reliably executed due to outdated dependencies and software incompatibilities. Originally developed for strain-level classification of short reads, it is based on an expectation–maximization (EM) algorithm for read reassignment [44]. As part of the MORA study, the authors introduced a continuation of PathoScope2, referred to as AugPatho, which includes a modified version of the original algorithm adapted for use with long reads [32]. MORA extends this approach by combining the EM algorithm from Agamemnon [45] with a read reassignment strategy based on the Weapon–Target Assignment (WTA) problem. According to its authors, MORA represents the current state-of-the-art in mapping-based long-read metagenomic classification.

Beyond algorithmic differences among tools, practical performance in metagenomic workflows can also be influenced by upstream preprocessing decisions and dataset composition, which can substantially affect downstream taxonomic inference [46].

Reference databases often contain multiple assemblies of the same strain and typically lack consistent organization. To address this, some strain-level classification tools perform database pre-clustering according to average nucleotide identity (ANI) scores. Previous studies have shown that there is no universal ANI threshold for defining strains [34, 40, 41, 47, 48]. Setting the threshold too high may erroneously separate assemblies of the same strain, whereas setting a threshold too low may incorrectly group different strains together.

Using large and diverse databases is important for accurate strain-level classification [49], but it also makes the analysis much more demanding to run. MetaAlign [50] uses containment MinHash [51] to reduce the reference database prior to alignment, improving runtime while maintaining high species-level precision. However, it is primarily designed for short reads, and strain-level resolution is not its main focus.

In this work, we introduce MADRe, a pipeline for long-read, strain-level metagenomic classification enhanced with Metagenome Assembly-Driven Database Reduction, consisting of two main phases: database reduction and read classification. In the database reduction step, MADRe combines long-read assembly with an EM algorithm that assigns assembled contigs to one or more references, reducing the reference database.

In the second, read classification step, MADRe performs mappings-based read reassignment. It resolves ambiguous read mappings by assigning each read to the most likely reference, based on mapping scores and probabilistic support.

We conducted an extensive evaluation of MADRe using simulated datasets, Zymo mock communities, and a real anaerobic digester sludge metagenome. The results demonstrate that MADRe achieves high precision and strain-level resolution while maintaining lower memory usage and runtime compared to existing tools. Additionally, the two steps of the MADRe pipeline can be run independently, and our results show that the read classification module (MADRe_RC) alone performs competitively. MADRe’s approach enables the use of large, diverse reference databases spanning multiple taxonomic levels, making it well-suited for scenarios where no prior knowledge about the sample is available. Using assembled contigs to detect potentially present strains, MADRe focuses on confidently represented organisms. As a result, compared to state-of-the-art tools, it significantly reduces the number of false positive identifications while maintaining high-resolution strain-level classification.

## Results

### MADRe—method overview

The MADRe pipeline is designed for strain-level metagenomic classification, particularly in scenarios where prior knowledge of the sample composition is not available. Its primary goal is to enable accurate strain identification while reliably distinguishing truly abundant strains from false positives.

**Figure 1:**
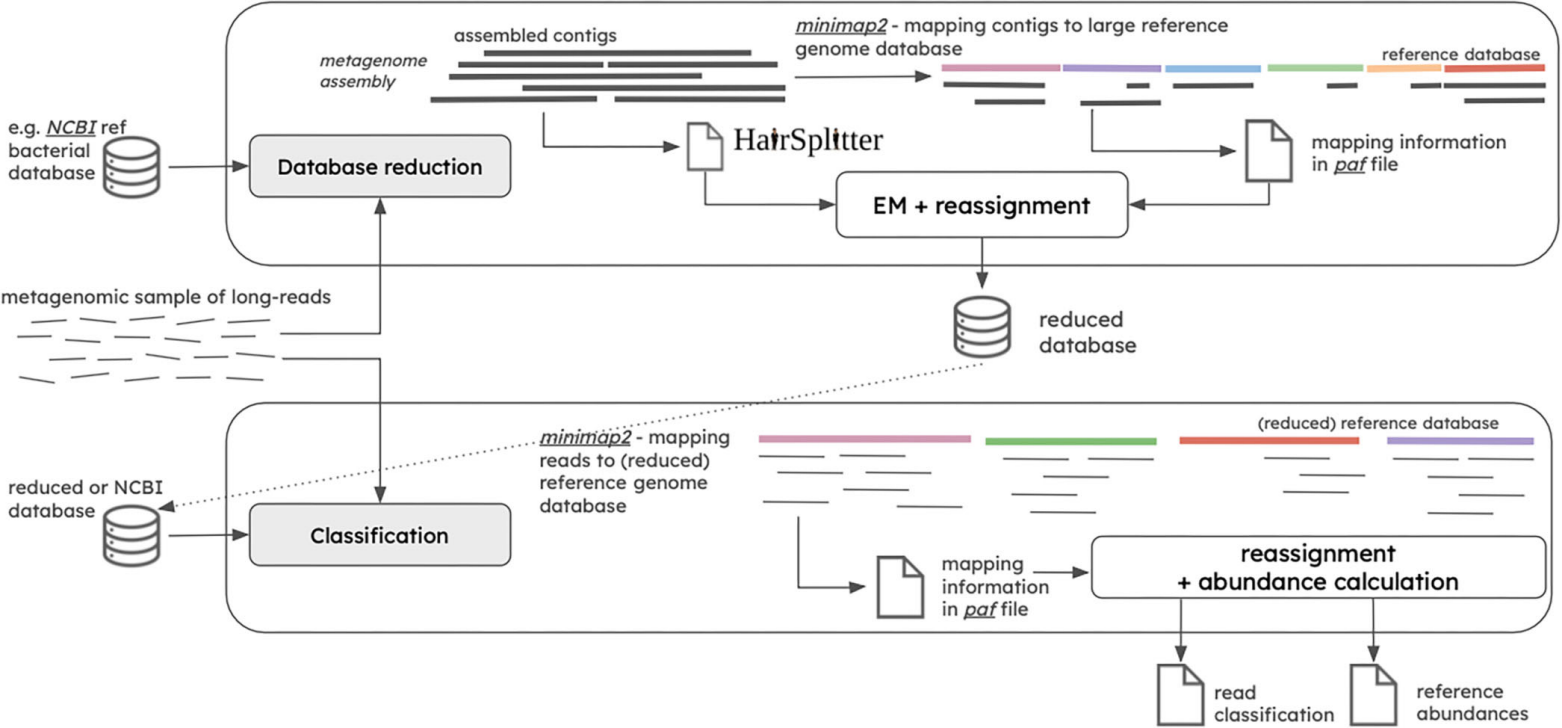
**MADRe overall pipeline**. The first step of the pipeline performs database reduction using an EM-based contig-to-reference mapping procedure to identify organisms present in the sample. The second step involves read classification, which applies probabilistic read reassignment based on mapping information.

As illustrated in Figure 1, the MADRe pipeline consists of two main steps: database reduction and read classification. It takes as input a large bacterial reference database and raw long metagenomic reads, and produces two main outputs: a read classification file and a reference abundance file.

In the database reduction step, long reads are first assembled, and the resulting contigs are mapped to the large reference database. MADRe employs HairSplitter [52] to estimate the number of collapsed strains represented by each contig. Using both contig-to-reference mappings and the collapsed strain estimates, an EM algorithm is applied, followed by additional read-reassignment steps. This process generates a reduced reference database that highlights strains likely present in the sample. Although this reduction effectively narrows the search space, it may still include false positives introduced during the assembly process. These could be further filtered during the read classification step.

In the second step, reads are mapped to the reduced reference database, and ambiguous mappings are resolved through a reassignment procedure that leverages mapping scores and probabilistic support.

Both steps of the pipeline can be run independently, so when prior knowledge about the sample exists or when a reduced reference set is already available, the read classification step can be used on its own.

### Benchmarking details

We benchmarked the MADRe pipeline against state-of-the-art tools developed for the same purpose: handling large reference databases while enabling strain-level classification. These tools include MORA and AugPatho (PathoScope2), for which we evaluated both of its key modules, PathoID and PathoReport. In some of the experiments, we also evaluated k-mer-based tool Kraken2, one of the most widely used metagenomic classification tools, which is often considered the standard for species-level classification. Although Kraken2 is capable of assigning reads at the strain level, its evaluation is complicated by the use of taxonomic identifiers (taxIDs) that may refer to either species or strain ranks. Despite its popularity, recent work has shown that Sylph achieves superior performance for species-level abundance estimation, reporting fewer false positives. However, since Sylph is primarily designed for abundance profiling rather than direct read classification, we did not include it in our benchmarking, which focuses explicitly on classification accuracy. Additionally, we included MADRe_RC, a variant of MADRe that performs only the second step (i.e., read classification) without prior database reduction.

We did not include MetaMaps, PanTax, or Centrifuge in our benchmarking analysis. In the case of MetaMaps, previous studies have reported crashes when attempting to build an index for the full Genome Taxonomy Database (GTDB), highlighting its scalability limitations [26, 32]. Similarly, our attempts to construct the same reference database for PanTax and Centrifuge, used successfully with other benchmarking tools, also failed due to crashes during the indexing process. Instead, we evaluated Centrifuger, a recent successor of Centrifuge that introduces improved compression and indexing strategies, enabling efficient classification on large-scale genome databases.

By default, MADRe employs metaFlye [53] for assembling ONT reads and metaMDBG [54] for assembling PacBio HiFi reads. To assess the effect of different assembly strategies on database reduction, we also performed additional experiments using Myloasm [55], a recently developed assembler showing promising performance on metagenomic datasets.

All commands used to run the benchmarking tools are available in the Supplementary Material (Tools versions and commands).

### Datasets

As part of the benchmarking process, we evaluated the mentioned tools on simulated metagenome datasets, Zymo mock communities, and a real anareobic digester sludge metagenome.

For medium-sized simulated datasets, we selected a smaller subset of genomes representing species commonly found in the human gut microbiome. Reference genomes were required to be labeled as “complete” or “chromosome” in NCBI, and to have a strain-level taxID distinct from their species-level taxID. This criterion ensured that Kraken2 could be included in the evaluation.

Using Badread tool [56] we simulated three different metagenomic datasets:

1. **sim_small (4 strains)** – This dataset includes four different strain references: two strains from *Adlercreutzia equolifaciens* and two from *Streptococcus anginosus*, with varying relative abundances.
2. **sim_medium (15 strains)** – This dataset contains 15 strain references distributed across five bacterial species (i.e., *Helicobacter pylori, Cutibacterium acnes, Streptococcus intermedius, Streptococcus mutans*, and *Lactococcus lactis*), with each species represented by three strains. At species level the abundances are different while strains of one species are equally abundant.
3. **sim_expanded (30 strains)** – An extension of the sim_medium dataset, incorporating 15 additional strains from distinct species and maintaining variable abundance levels across species. Newly added species are listed in Supplementary Table S1.

Exact genome information, including accession numbers, strain and species taxIDs, genome lengths, genome coverages, ANI values (calculated usin fastANI [47], and number of simulated reads, can be found in the Supplementary Tables S1– S4.

Although these datasets can be used to assess MADRe’s performance, they remain relatively simple and do not fully reflect the complexity of real metagenomic samples. Therefore, we expanded our benchmarking to include four additional simulated datasets originally used in the PanTax study [5] and we called them large-sized simulated datasets. Three of these datasets each contain 60 genomes coming from 30 species, simulated using ONT R9.4.1, ONT R10.4.1, and PacBio HiFi error profiles, respectively. These datasets were obtained directly from the PanTax Zenodo repository [57]. In addition, we generated a fourth, large-scale dataset comprising 1,000 genomes from over 300 species, inspired by the CAMI challenge design. As simulated reads for this dataset were not available due to its size, we used the published reference genomes and expected abundances to simulate reads with the Badread tool. For these datasets, we additionally present distributions of ANI scores (calculated using fastANI), illustrating how many genome pairs exceed predefined ANI thresholds, as shown in Supplementary Table S10.

We also tested MADRe using three Zymo mock communities: D6322 (ONT), D6331 (ONT) [58], and D6331 (PacBio HiFi) [59]. We included both D6311 ONT and D6311 PacBio HiFi datasets to demonstrate the pipeline’s capability across different sequencing technologies.

To evaluate performance on a complex real-world dataset, we analyzed an anaerobic digester sludge metagenome dataset sequenced using ONT R10.4.1. reads [60]. This real metagenome represents the type of scenario for which MADRe is designed, where a highly diverse sample is analyzed without prior knowledge of its taxonomic composition.

### Database

To thoroughly evaluate strain-level classification and ensure sufficient taxonomic divergence for accurate strain-level detection, we used a database obtained via the Kraken2 interface by selecting the bacterial database. This database consists of 102,639 sequences, encompassing all RefSeq [61] complete bacterial genomes. The database was downloaded in December 2024. The exact command used for downloading the database is provided in the Supplementary Material (Tools versions and commands).

For consistency, the same database was used across all tools and experiments.

### Database reduction

To assess the effectiveness of strain identification and database reduction, we evaluated the output of MADRe’s database reduction step on simulated datasets by comparing it to two baseline models. More precisely, assembled contigs were mapped to the large reference database, and the identification of organisms was carried out using the following strategies:

- Baseline Model 1 (BM1): For the reduced database, each contig’s top reference genome, determined by the highest summarized harmonic mean mapping value (Methods, Equation 2), was included without performing any reassignment steps. BM1 represents the ideal reduction level under the assumption of a perfect assembly, where each contig corresponds to a single strain reference.
- Baseline Model 2 (BM2): For the reduced database, each contig’s top three reference genomes, based on the highest summarized harmonic mean mapping values (Methods, Equation 2), were included without performing any reassignment steps. BM2 defines an upper bound on the number of references expected in the reduced database. Since our simulated metagenomes contain at most three strains per species, we assume that at most three strains could be collapsed into a single contig. This approach allows us to determine how many references should be retained in the reduced database to ensure that no true reference from the sample is missed.

The results, presented in Table 1, demonstrate that MADRe’s database reduction achieves a high level of reduction while successfully retaining all expected strains. Additionally, when compared to BM2, MADRe reduces the number of false positive species, further highlighting its effectiveness.

**Table 1:** **Database reduction results on medium-sized simulated datasets**. Comparison of MADRe’s database reduction performance with two baseline models on simulated datasets using a large database containing **102,639** sequences: baseline model 1 (BM1), which includes only the top-1 mapping for each contig, and baseline model 2 (BM2), which includes the top-3 mappings.

	Metric	sim_small	sim_medium	sim_expanded
Number of genomes in dataset		4	15	30
**BM1**	# in reduced	7	34	68
	# missing strains	0	1	3
	# FP strains	3	19	38
	# FP species	1	0	6
**BM2**	# in reduced	14	107	234
	# missing strains	0	0	0
	# FP strains	10	92	204
**MADReDatabase Reduction**	# FP species	5	11	78
	# in reduced	7	42	84
	# missing strains	0	0	0
	# FP strains	3	27	54
	# FP species	1	0	6

### Classification of medium-sized simulated datasets

For medium-sized simulated datasets we compared the classification performance of MADRe, MADRe_RC, MORA, AugPatho (in both PathoID and PathoReport modes), Kraken2, and Centrifuger. Since the exact source of each read is known, we define classification outcomes as follows: true positive (TP) if the read is classified under the expected strain (or expected cluster); true negative (TN) if the read is not classified and its Badread label is *random* or *junk*; false positive (FP) if the read is classified under the incorrect strain (or incorrect cluster); and false negative (FN) if the read is not classified but its label is different from *random* or *junk*.

We evaluated classification of simulated data with and without post-clustering. The post-clustering method described in the Methods section groups closely related strains based on read-to-reference mappings and assigns reads to clusters instead of individual strains. The same clustering approach was applied to all tools, utilizing read mappings to the full reference database. Kraken2’s clustering results are not included, as taxID alone does not allow for an accurate evaluation of post-clustering performance.

**Figure 2:**
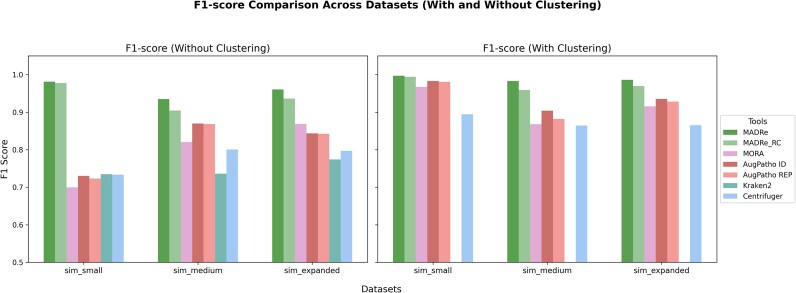
**Classification performance on medium-sized simulated datasets **.  F1 scores of strain-level classification on medium-sized simulated reads, shown with and without post-clustering (grouping highly similar strains). Kraken2 results were omitted from the clustering analysis, as its output format does not support proper clustering.

**Figure 3:**
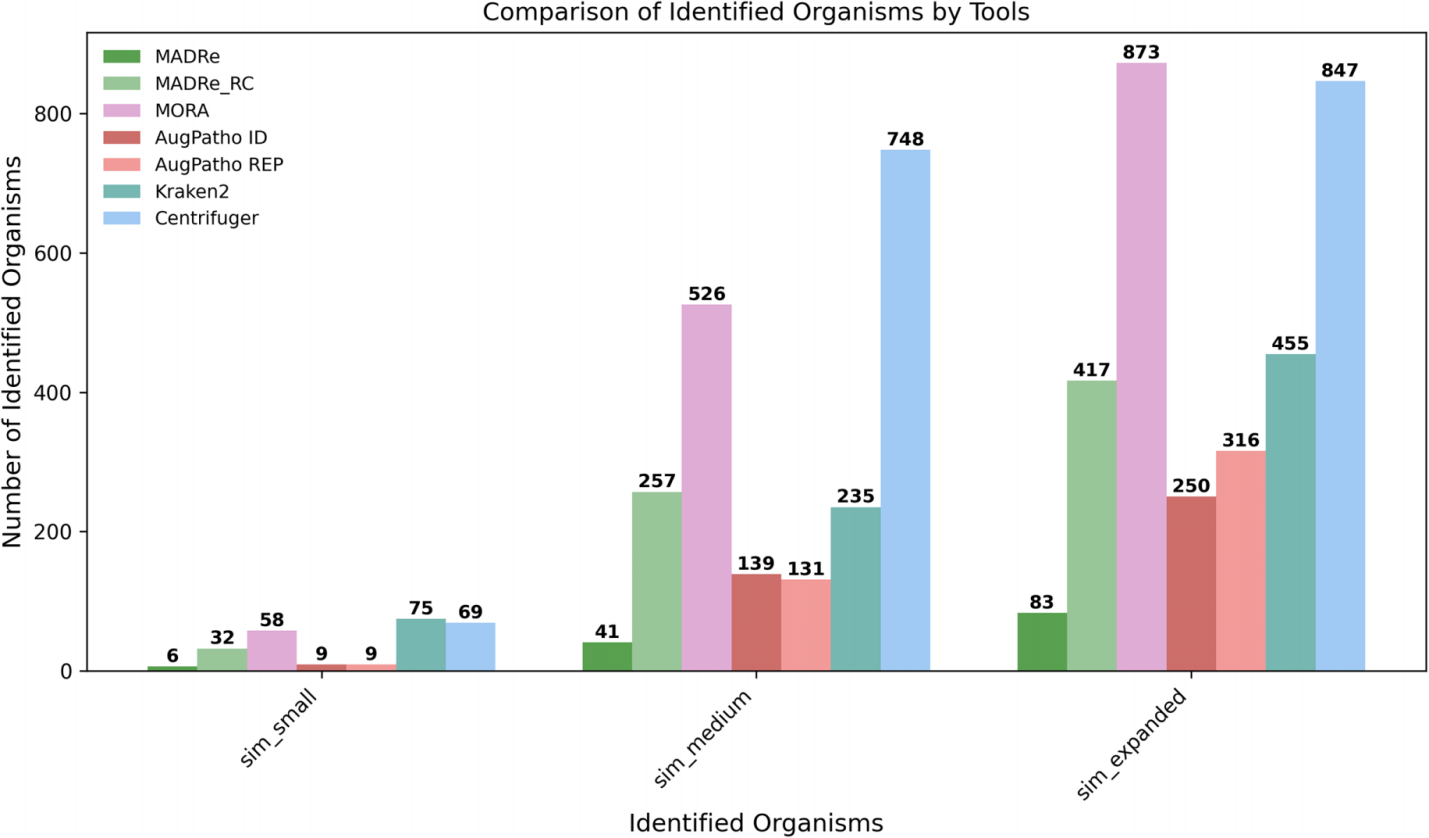
**Number of identified organisms on medium-sized simulated datasets**. An organism is considered identified if at least one read is classified under it. The sim_small dataset contains 4 strains, sim_medium contains 15 strains, and sim_expanded contains 30 strains. All tools successfully identified all expected strains, resulting in no false negatives.

The classification results of medium-sized simulated datasets (sim_small, sim_medium and sim_expanded) are presented in Figure Figure 2 , which shows the F1 scores for classification with and without post-clustering. The results demonstrate that both MADRe and MADRe_RC outperform all other approaches. Interestingly, on the sim_small dataset, which includes differently abundant strains of the same species, Kraken2 performs slightly better than Centrifuger, while Centrifuger achieves higher scores than MORA and AugPatho. For the remaining datasets, Centrifuger performs better than Kraken2 but worse than MORA and AugPatho. In the sim_expanded dataset (without clustering), MORA outperforms AugPatho’s modes, but in all other cases, including all clustering scenarios, both AugPatho’s modes perform significantly better than MORA. When post-clustering is applied, Centrifuger shows the lowest performance across all datasets, while post-clustering further improves AugPatho’s results, bringing them close to MADRe_RC. Overall, MADRe_RC achieves performance comparable to MADRe, although this difference becomes more pronounced on more complex datasets. Figure 3 shows the number of organisms identified by different tools on simulated datasets. An organism is considered identified if at least one read is classified under it. In all cases, there were no false negatives—all tools successfully identified the expected organisms. However, the number of additional (false positive) identifications varies. MADRe consistently reports significantly fewer false positives. For example, in the sim_small dataset, only six organisms were reported compared to the four expected, with two of them being extremely similar strains to those actually present in the sample. Additional metrics for organism-level identification, including TPs, FPs, FNs, accuracy, precision, recall, and F1 scores, are provided in Supplementary Table S8, while classified read counts for each organism are listed in Supplementary Tables S5–S7.

In addition, a Supplementary Fig. S4 and Table S9 show Bray–Curtis (BC) distances [62] between the observed read count abundances and the ground-truth abundances, offering further insight into the similarity between the predicted and true community compositions. BC distance is one of the most commonly used distances to calculate the microbial abundance differences, and is described in the Methods section. From these results, it is evident that MADRe achieves the closest match to the ground truth, while AugPatho ID reports the poorest scores among strain-level tools, including Centrifuger. Kraken2, although computationally efficient, shows the weakest overall performance.

### Classification of large-sized simulated datasets

To further assess classification performance under more realistic metagenomic conditions, we used the simulated datasets from the *PanTax* study. We used the same evaluation procedure as the one considered for the medium-sized simulated datasets. In addition to benchmarking the standard MADRe pipeline, we also evaluated a variant in which Myloasm was used as the assembler during the database reduction step in order to examine how different assembly approaches influence MADRe’s performance. This variant was not tested on the sim_low R9.4.1 dataset, as Myloasm is not suitable for reads with that error profile.

**Figure 4:**
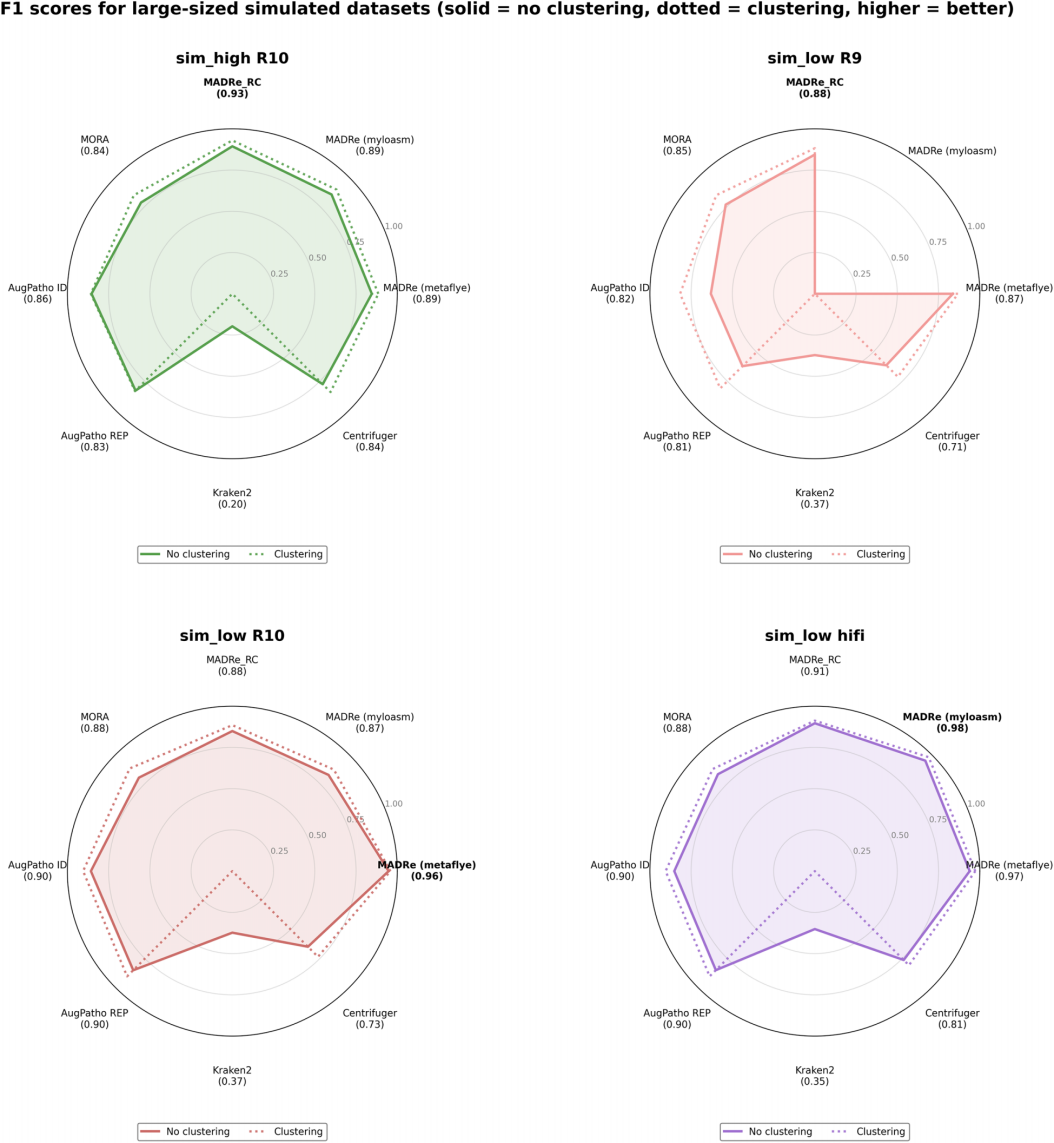
**F1 scores for large-sized simulated datasets**. Solid lines represent results without clustering, while dotted lines indicate results with clustering. Kraken2 clustering results are omitted, as its output format does not support clustering. Similarly, MADRe (Myloasm) results are excluded for ONT R9 data, since Myloasm is not designed for this type of sequencing data. In each plot, the best-performing tool is highlighted in bold, and the values in parentheses indicate the best performance achieved by each tool, with and without clustering.

Figure 4 presents four radar plots, each corresponding to one of the four large-sized datasets, and showing F1 scores for all evaluated tools, both with and without post-clustering. (Note that post-clustering values for Kraken2 are zero, since this step was not performed for that tool.) For each tool, the best obtained F1 score is indicated in parentheses beneath its name. Across most datasets, MADRe achieved the highest scores, including its versions using different assemblers. In some cases, MADRe_RC slightly outperformed MADRe, particularly on the sim_high dataset. Detailed evaluation statistics are provided in Supplementary Table S15. Exact read counts obtained from classifications are listed in Supplementary Tables S11–S14, while BC distances between observed and expected read-count abundances are shown in Supplementary Fig. S5 and Table S16.

Inspection of read-count abundances in the sim_high dataset revealed that several genomes were not detected during the database reduction step, leading to a modest decrease in MADRe’s performance compared to MADRe_RC.

When comparing assembler performance, MADRe runs based on Myloasm assemblies achieved slightly higher F1 scores than those using metaFlye. A closer look at read-count abundances revealed that 24 genomes were detected exclusively with Myloasm and 11 exclusively with metaFlye. Among the 11 missed by Myloasm, 9 belonged to the more abundant half of the community, whereas among the 24 detected only by Myloasm, just one was highly abundant. This pattern suggests that Myloasm-based contigs perform better for low-abundance strains, while metaFlye contigs remain more reliable for highly abundant ones.

Another observation from the sim_high dataset is that, for several highly abundant strains, most benchmarking tools reported substantially lower read-count abundances than the ground truth. For example, reads originating from NZ_CP012672.1 were predominantly assigned to NZ_CP102233.1, a nearly identical genome (ANI = 99.998) annotated under a different species (Sorangium cellulosum vs. Sorangium sp. So ce836). Because clustering was performed only among strains within the same species, this near-duplicate across species boundaries could not be resolved even after clustering.

As shown in Figure 4, all tools achieved higher F1 scores on ONT R10 and PacBio HiFi datasets compared to ONT R9, reflecting the higher base accuracy of these sequencing platforms. Interestingly, for the ONT R10 dataset, MADRe using Myloasm performed slightly worse than the metaFlye version, whereas for PacBio HiFi reads, Myloasm yielded marginally better results than the metaMDBG-based variant.

Taken together, these results, including the analyses of BC distances, demonstrate that MADRe, in all assembler configurations, consistently outperforms the other evaluated tools on the large-sized simulated datasets.

### Classification of Zymo mock communities datasets

To evaluate MADRe on real sequencing data, we conducted experiments on three different Zymo mock community datasets: ONT Zymo D6322, which consists of eight organisms (seven bacterial species and one fungus), and both the ONT and HiFi versions of Zymo D6331, which contain 21 organisms, including two fungi and five different strains of *Escherichia coli*. The primary challenge in the Zymo D6331 dataset is the ability to distinguish between these closely related *E. coli* strains. In this analysis, we excluded fungal genomes, focusing solely on bacterial classifications.

For Zymo mock communities, exact reference genomes of the strains present in the sample are available, along with their theoretical relative abundances provided by ZymoBIOMICS [63]. We supplemented our database with the Zymo reference genomes, assigning them separate labels. However, we did not use provided theoretical abundances in our analysis, as they may deviate from the expected values due to variations in library preparation [9, 31]. Instead, we established ground-truth read classifications. We mapped all reads to the expected bacterial reference genomes using Minimap2 and assigned true labels based on the best hit. These assignments were also used to determine the relative abundances. However, this process was not straightforward for the five *E. coli* strains, as their high similarity led to ambiguous mappings. To address this, we leveraged our clustering method (explained in *Similar strains clustering* section), which grouped these five strains into three clusters. Specifically, strains B766 and B3008 each formed separate clusters, while the remaining three strains were grouped into a single cluster, indicating that they were too similar to be reliably distinguished at the strain level. This clustering result aligns with previous findings from metagenome assembly procedures [64], where B766 and B3008 were successfully assembled, while the other three strains were not.

Benchmarking with Centrifuger and Kraken2 was not performed for this experiment, as their database construction procedures do not support the inclusion of references with custom labels, which is essential for this evaluation.

Using this information, we incorporated the clustering results into our ground-truth labeling: reads originating from the same cluster were assigned the same label, ensuring a more accurate classification.

To evaluate performance, we calculated the BC distances (equation 9) between the observed read count abundances and the ground-truth abundances, both with and without post-clustering.

**Figure 5:**
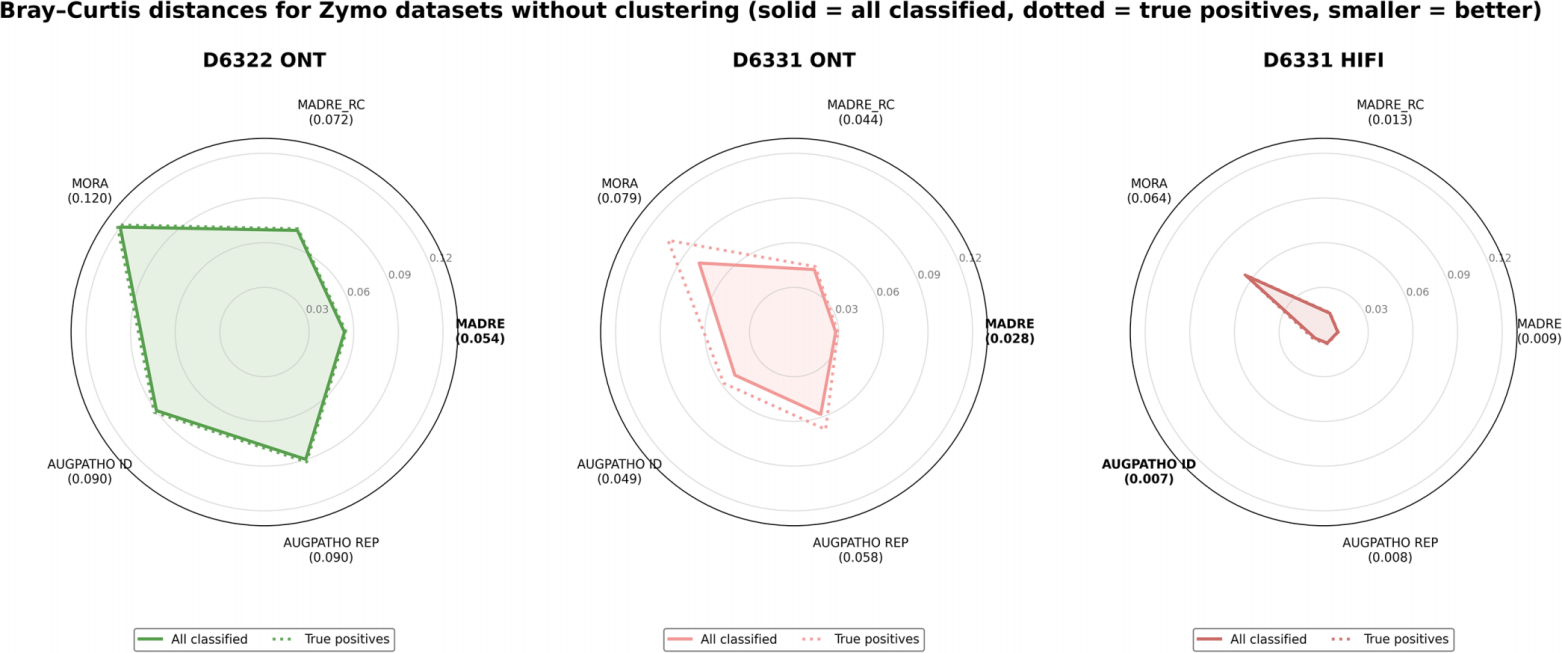
**Bray–Curtis distances for Zymo datasets**. The plots show changes in BC distance without post-clustering of similar strains. Solid lines represent distances based on all classified read counts, while dashed lines show distances calculated using only true positive (TP) read counts.

Figure 5 depicts radar plots showing the BC distances for the zymo D6322 ONT, zymo D6331 ONT, and zymo D6331 HiFi datasets. Dotted lines indicate BC distances computed using only TP classifications based on the ground truth. In the first plot, which reports results for the D6322 dataset, MADRe clearly outperforms all other tools. The second and third plots display BC distances for the D6331 ONT and HiFi datasets, respectively. For the ONT dataset, when considering all classified reads, MADRe achieves the lowest BC distance. When focusing only on TP, MADRe and MADRe_RC show comparable performance, indicating that the majority of reads classified by these tools are correctly assigned. In contrast, MORA exhibits a notably higher BC distance when evaluated only on TPs, suggesting less precise classification. For the HiFi dataset, overall distances for all the tools are significantly lower. Both AugPatho modes achieve slightly lower BC distances compared to MADRe. In Supplementary Fig. S6, we present the corresponding results obtained after post-classification clustering of similar strains. Interestingly, for the ONT datasets, BC distances increased for both AugPatho and MADRe following clustering. Although the increase is not substantial, the clustering step led to elevated abundance estimates, resulting in a higher number of both false positives and TP. This trend was not observed for the HiFi dataset, where MADRe achieved the best performance after clustering.

The exact read counts, used to calculate BC distance, are listed in the Supplementary Tables S17–S19.

Table 2 presents the number of false-positive species and strain identifications. MADRe reports a significantly lower number of false positives at both levels compared to other tools. Supplementary Table S20 provides a more detailed breakdown of the number of identifications. From this table, it is evident that MADRe’s main limitation is the higher number of false negatives, primarily originating from low-abundance organisms that could not be detected using the assembly-based approach on which MADRe relies. This is further supported by the MADRe_RC results, where the number of false negatives is comparable to other tools. Nevertheless, MADRe consistently reports a substantially lower number of false positives. The table also includes AugPatho results from report outputs from both modes. These reports are generated after the final reassignment step and contain only abundance estimates. Consequently, they cannot be used directly for classification evaluation. While these reports show a significantly lower number of false positives, this reduction comes at the cost of a higher number of false negatives.

**Table 2:** **False positive (FP) species and strains detected by different tools on Zymo datasets**. An organism is considered a false positive if at least one read is classified under it, but it is not in the true community.

	Tool	D6331 ONT	D6331 HiFi	D6322 ONT
**FP Species**	MADRe	5	5	6
	MADRe_RC	391	53	385
	MORA	639	162	517
	AugPatho ID	266	10	327
	AugPatho REP	249	20	260
	MADRe	386	114	52
	MADRe_RC	3,441	1,189	6,441
**FP Strains**	MORA	6,251	4,010	10,357
	AugPatho ID	2,641	518	6,133
	AugPatho REP	2,316	889	4,675

### Classification of real anaerobic digester sludge metagenome

While Zymo mock communities represent real metagenomic data, they do not fully capture the complexity typically found in environmental or host-associated microbial communities. To better reflect realistic classification scenarios, we evaluated MADRe and the other competing tools on a real anaerobic digester sludge metagenome. As this dataset lacks ground truth, we focused on comparative analysis of classification outputs. All results presented here include post-clustering.

In this dataset, MADRe identified 1,320 reference strains (1,502 without clustering), while MADRe_RC reported 14,304 (19,067 without clustering), MORA 15,835 (23,516 without clustering), AugPatho ID 11,785 (16,488 without clustering), AugPatno REP 11,134 (14,604 without clustering) and Centrifuger 23,950 (28,450 without clustering). Out of 3,646,771 total reads, MADRe classified 575,052 ($\sim 16\%$), MADRe_RC 696,961 ($\sim 19\%$), MORA 696,839 ($\sim 19\%$), AugPatho ID 898,906 ($\sim 25\%$), AugPatho REP 737,714 ($\sim 20\%$), and Centrifuger 1,350,537 ($\sim 37\%$) reads.

**Figure 6:**
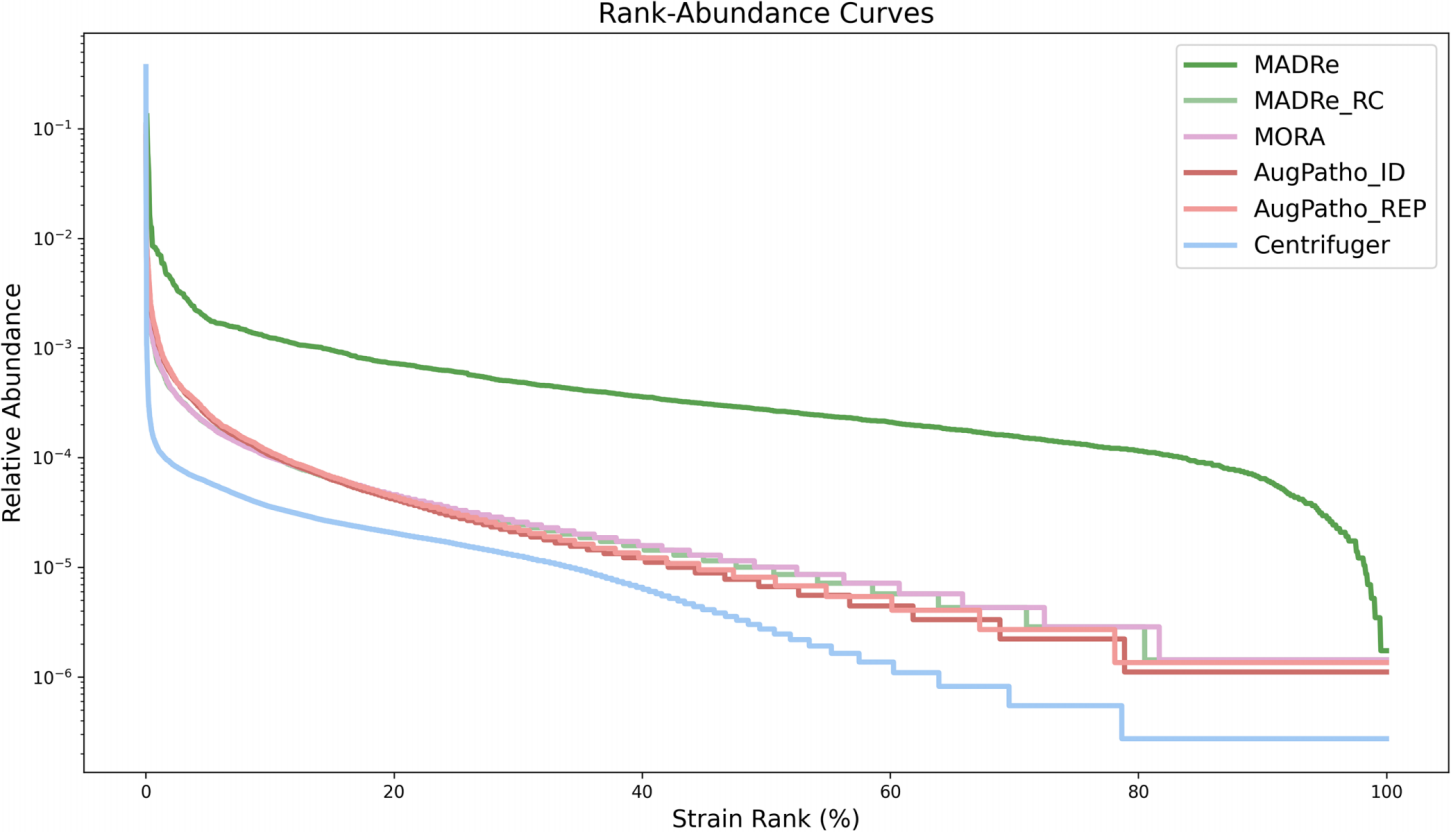
**Percentile-normalized rank–abundance curves for the anaerobic digester sludge metagenome**. The *x*-axis shows strain ranks expressed as percentiles, while the *y*-axis represents the relative abundance of each strain on a logarithmic scale.

Figure 6 illustrates percentile-normalized rank-abundance curves, highlighting differences in strain-level classification across the tools. The underlying read count abundance data used to generate this figure is provided in Supplementary Table S21.

The curve for MADRe displays a consistent, moderately steep gradient throughout, with notable deviations at the beginning and end. The sharp rise at the beginning indicates the presence of a highly abundant strain, significantly more dominant than the others. This can be seen for the other tools as well. Toward the end, the curve drops sharply, likely reflecting false positives or low-confidence strain assignments. Compared to the other tools, MADRe shows a smoother and more gradual decline in the abundances of lower-ranked strains. In contrast, MADRe_RC, MORA, AugPatho, and Centrifuger report a larger number of low-abundance strains, resulting in a more stepwise decline. The flat tail in their curves suggests that many strains are assigned near-zero abundances.

**Figure 7:**
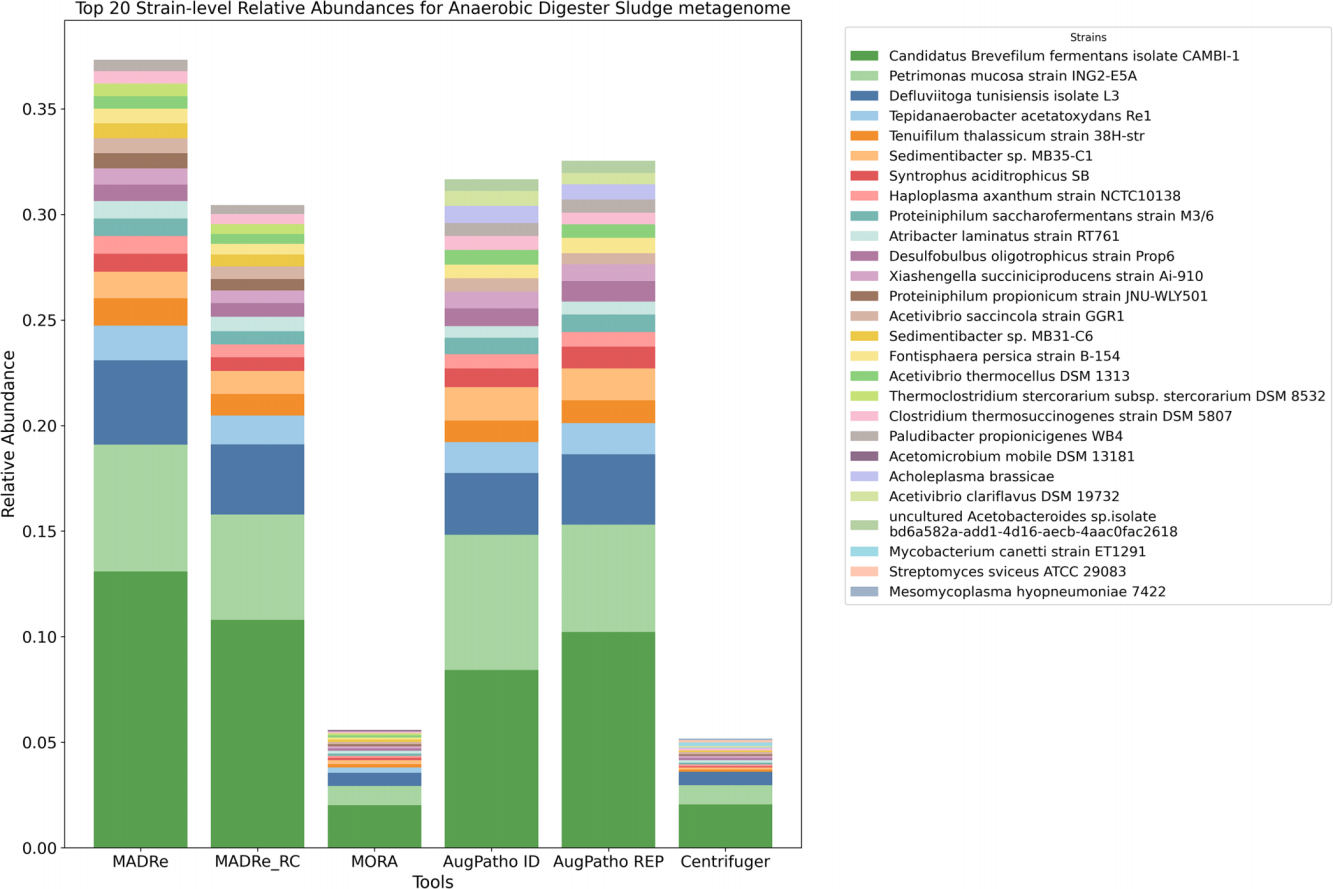
**Strain-level abundances of the top 20 strains across tools on the anaerobic digester sludge metagenome**. MADRe achieves the highest cumulative abundance, while MORA and Centrifuger show substantially lower contributions. A notable discrepancy is observed for *Acholeplasma brassicae* (NC_022549.1), highlighted in red, which appears among the top 20 only in AugPatho, whereas other tools assign these reads to different species within the same genus, indicating ambiguity caused by shared genomic regions and the absence of the true reference genome.

Figure 7 shows the relative abundances of the 20 most abundant strains reported by each tool, calculated relative to the total number of classified reads. We also generated an analogous visualization at the species level (Supplementary Fig. S7), which additionally includes Kraken2 results. Among all tools, MADRe achieved the highest cumulative relative abundance for the top 20 strains, followed by AugPatho and MADRe_RC, while MORA and Centrifuger exhibited similar but substantially lower overall contributions from their top strains. Figure 7 highlights one notable strain-level discrepancy: the strain *Acholeplasma brassicae* (accession number NC_022549.1, taxID 61635), which appeared among the top 20 only in AugPatho results. To investigate this discrepancy, we examined how reads classified as taxID 61635 by AugPatho were assigned by other tools. We found that most of these reads were classified as taxID 2148 or 264636 by the other approaches. As all three of these strains belong to the *Acholeplasma* genus, this pattern suggests the presence of shared genomic regions and potentially an unrepresented or novel species within this genus. To further examine this case, we mapped the relevant reads to all three references and found that none yielded strong, confident alignments, indicating that the true source strain is likely missing from the reference database. We then assembled the corresponding reads into contigs and classified them using Kraken2 against the full database. In 17 contigs classified under the expected family, the highest number of k-mers matched strain 2148, although the counts were low, again supporting the hypothesis of a missing true reference. Interestingly, strain 61635 is longer than both 2148 and 264636, and prior work on MORA has shown that AugPatho’s scoring tends to favor longer, more complete genomes, which likely explains its preference for strain 61635 in this case.

### Time and memory resources

**Figure 8:**
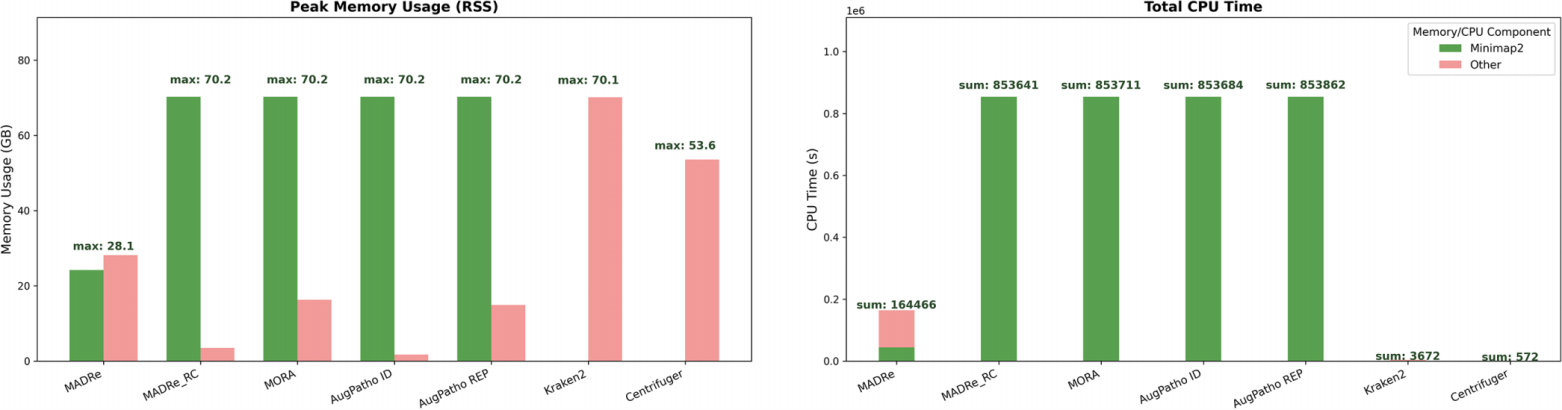
**Computational resource usage across tools on the ZymoD6331 ONT dataset**. Peak memory usage (RSS, in GB) and CPU time (in seconds) for each tool on the ZymoD6331 ONT dataset. Results are separated into Minimap2 mapping and other processing steps to highlight the contribution of each stage to the overall computational cost.

Figure 8 presents the runtime and peak memory usage of the benchmarking tools on the ZymoD6331 ONT dataset, which contained $\sim$1.7 M reads.

Since the majority of the tools, except Kraken2 and Centrifuger, rely on Minimap2 for read mapping, we categorized peak memory usage into components: memory used by Minimap2 and memory used by other operations. Similarly, CPU time was divided into time spent by Minimap2 and time spent on all other processing steps.

In the case of MADRe_RC, MORA, and AugPatho, the “other operations” category solely consists of the read reassignment algorithm. In contrast, for MADRe, it includes assembly, HairSplitter, database reduction, and read reassignment. The role of Minimap2 also differs across MADRe and other tools. In MADRe_RC, MORA, and AugPatho, it is used for mapping reads to the large reference database, whereas in MADRe, it is used both to map contigs to the large database and reads to the reduced one.

For HiFi reads, Minimap2 uses different parameters, and the MADRe pipeline employs metaMDBG instead of metaFlye for assembly. To account for these differences, Supplementary Table S22 reports the same performance metrics for HiFi data.

When the dataset size increases, the situation changes. To illustrate this, we included runtime and memory usage results for the large simulated dataset sim_high (containing $\sim$5 M reads) in Supplementary Table S22. In this case, the peak RSS for MADRe is substantially higher (exceeding 200 GB), primarily due to the assembly process, while the peak RSS for Minimap2 during read mapping to the large database remains unchanged. However, mapping reads to such a large database requires the “–split-prefix” parameter in Minimap2, which generates temporary alignment files that are later merged at the end of the process. For this particular dataset, that procedure consumes ~1.2 TB of disk space, whereas the complete MADRe pipeline requires  160 GB (excluding database and read file sizes in both cases). Moreover, the entire MADRe pipeline is ~3.2× faster than the combination of Minimap2 with MORA or AugPatho. In contrast, Kraken2 and Centrifuge are substantially faster than mapping-based approaches and require considerably less disk space.

## Discussion

In this work, we introduced MADRe, a metagenomic classification pipeline based on assembly-driven database reduction followed by read classification through mapping and reassignment. By introducing statistical strategies for database reduction and read reassignment that explicitly leverage assembly-derived genomic context, MADRe represents a methodological contribution rather than an engineering integration alone. This approach enables accurate strain-level classification from large, multi-species databases without requiring prior knowledge of sample composition.

The first phase of MADRe reduces the reference database by identifying candidate strains that are likely to be present in the sample. Using assembly and an EM soft clustering algorithm, this step aims to retain only the relevant references while eliminating unrelated ones. To take advantage of the longer contigs produced by standard assemblers, we avoided using strain-aware metagenome assemblers such as Strainberry [65], MetaBooster [66], HyLight [66], Strainy [67], and HairSplitter [52], which are known to yield shorter contigs. Instead, we used HairSplitter’s functionality to estimate the number of collapsed strains for each contig and integrated this information with the mapping data of the initially strain-collapsed contigs. Our evaluation demonstrates that MADRe achieves effective database reduction while maintaining high recall.

Most existing strain-level classifiers either require single-species input or do not scale to large reference databases. Tools such as Kraken2, Sylph, or Centrifuger perform well at the species level and can handle large databases, making them valuable for pre-classification in strain-level workflows. However, such approaches generally require additional database preparation steps that are computationally intensive and impractical for complex metagenomic samples.

Mapping-based methods such as MORA and AugPatho represent another way to perform strain-level analysis on large databases. Nevertheless, our experiments showed that although MADRe incorporates an assembly step, typically considered both memory- and time-intensive, the overall memory footprint was lower than mapping raw reads directly to a large reference database, and even lower in memory than running Kraken2 on the same reference, when applied to a dataset of ~1.6 million ONT reads. This indicates that the upfront cost of assembly can be offset by the reduced complexity of downstream mapping. Importantly, the EM-based refinement and the reference reduction algorithm itself contribute only minimal additional computational overhead compared to assembly and large-scale read mapping. For larger datasets that exceed 5 million reads, the assembly process becomes more memory demanding. However, MADRe remains substantially faster and requires considerably less disk space than mapping-based approaches. While the runtime and resource usage of Minimap2 could be reduced by using a smaller reference database, this would again require prior knowledge of the sample composition or risk omitting relevant strains. Among the evaluated strain-level tools, MADRe is the fastest, providing an effective balance between computational efficiency and classification accuracy, and is thus well suited for scalable strain-level metagenomic analyses.

Our benchmarking analysis compared MADRe to MADRe_RC, MORA, the two AugPatho modes (PathoID and PathoREP), and, in several cases, to Centrifuger and Kraken2. For selected datasets, we additionally evaluated abundance re-estimation using Centrifuger-quant (Supplementary Tables S5–S7, which reports read-count-based abundance estimates rather than read-level classifications and showed only minor differences compared to standard Centrifuger results; therefore, it was not included in all analyses. For AugPatho, we used the updated SAM files generated during its reassignment step, in which individual reads, in some cases, can be associated with multiple references. This format may improve the detection of expected references but can also introduce ambiguity, potentially contributing to higher false-positive rates. On the new simulated datasets, MADRe achieved up to a 28% improvement over other state-of-the-art methods when no clustering of similar strains was applied, and up to a 10% improvement when clustering was used. A similar trend was observed for the more complex large-sized simulated datasets. Although MADRe occasionally missed low-abundance strains in these datasets, it still produced more accurate classifications than competing tools. This is particularly important since MADRe focuses on precise read-level classification rather than on abundance estimation.

A major challenge in metagenomic evaluation is the scarcity of realistic benchmark datasets, which can lead to parameter overfitting across methods, often to well-known datasets such as the Zymo communities. This may explain observations like those in the D6331 ONT dataset, where MORA and AugPatho showed substantial discrepancies between BC distances calculated from all classified reads and those derived only from TP—the distances for TPs were notably higher. In contrast, MADRe consistently achieved better results than other tools for both evaluation types, demonstrating robust classification performance.

One limitation of MADRe observed in the Zymo benchmarks is the higher number of false-negative identifications, largely stemming from low-abundance organisms that are difficult to capture through the assembly-based approach, particularly when these organisms belong to species represented by a large number of closely related strains in the reference database. However, a similar effect can be seen in AugPatho’s final reports, which include only abundance estimates from the last reassignment step—these also exhibit increased false negatives. This highlights a broader issue in metagenomic classification: setting thresholds for reporting low-abundance taxa inevitably trades off between reducing false positives and increasing false negatives [68]. The identification and quantification of low-abundance organisms remain challenging problems. MADRe does not apply any automatic post-filtering, leaving the decision of whether to perform additional filtering or manual investigation of low-abundance taxa to the user.

A closer look at the composition of the Zymo datasets and the definitions of ground-truth labels provides additional insight into the observed differences in tool performance. We can clearly observe performance variation across the three Zymo datasets, which can be attributed to both the sequencing technology and the evaluation methodology. As expected, the D6331 HiFi dataset yielded the best results, reflecting the higher base-level accuracy of HiFi reads compared to ONT. At first glance, it may seem surprising that performance on D6322 ONT was lower than on D6331 ONT, since D6322 contains species from different genera and should, in principle, be easier to classify. The main factor explaining this discrepancy lies in how ground-truth labels were defined. For D6322, the evaluation was straightforward—each genome represented a distinct species, and thus, an exact species-level match was required for a correct classification. In contrast, D6331 includes five *E. coli* genomes, three of which have very high sequence identity (>99.3% ANI score—calculated using fastANI). When constructing the ground truth for D6331, we clustered these three genomes and considered a read originating from any of them as correctly classified if it was assigned to any genome within that cluster. This less stringent criterion results in higher apparent performance for D6331 compared to D6322, an effect that applies uniformly across all evaluated tools.

In the real metagenomic dataset, MADRe classified fewer strains and focused on a confident subset of dominant organisms. In contrast, MADRe_RC, MORA, AugPatho, and Centrifuger reported a much larger number of low-abundance strains. While this may suggest higher sensitivity, many of these additional detections are likely spurious strain-level assignments, particularly in cases where the data do not support precise strain resolution. In this dataset, certain true references were absent from the database. Under these conditions, AugPatho tended to favor longer, highly similar genomes; MORA and MADRe_RC dispersed reads across multiple low-abundance strains, whereas MADRe mostly assigned reads to the reference sharing the greatest number of similar regions with the true organism.

MADRe’s reduced detection of low-abundance strains arises primarily from limitations inherent to assembly-based approaches. At very low coverage, strains may fail to assemble or may produce contigs that are too short or fragmented to provide sufficient strain-specific signal for confident detection during database reduction. As a result, low-abundance strains may be excluded early in the workflow. This effect can be further amplified when closely related, higher-abundance strains are present, as assembly may preferentially represent the dominant genome, reducing the amount of discriminative sequence available for reliable separation.

These observations reflect an intentional precision–recall trade-off in the design of MADRe. By reducing the reference database, MADRe prioritizes minimizing false-positive strain detections. At the genome-identification level, this may lead to reduced recall for extremely low-abundance strains. However, at the read level, the impact is typically limited because such strains contribute only a small fraction of total reads. When a strain is not retained during database reduction, its reads are frequently assigned to a highly similar reference, resulting in stable recall but a potential reduction in precision. From a biological perspective, this behavior is advantageous in scenarios where false-positive strain detection carries greater consequences than missing very low-abundance organisms.

As noted above, challenges extend beyond low-abundance effects and arise when both the reference database and the analyzed dataset contain genomes exhibiting extremely high sequence similarity. This was investigated through a dedicated high-similarity stress test (Supplementary Material: Similar Strains Experiment; Supplementary Table S23) and additional synthetic mixtures spanning combinations of similarity and coverage depth (Supplementary Material: Coverage and Similarity Experiment; Supplementary Table S24). These experiments demonstrate that when sequence similarity exceeds 99.9% ANI, strain-level discrimination becomes intrinsically ambiguous, largely independent of coverage. While very low coverage can limit detectability, the dominant constraint in these regimes is sequence similarity rather than abundance. Under such conditions, all evaluated methods approach fundamental resolution limits, although they differ in how ambiguity is handled. MADRe addresses this ambiguity by consistently assigning reads to the most similar available reference (the centroid), yielding stable behavior even when exact strain-level resolution is not supported by the data.

These observations also emphasize a broader limitation of current long-read metagenomic classifiers: All existing methods struggle to resolve strains at extremely low sequence divergence. For this reason, in our evaluation we additionally report results at the cluster level, where highly similar genomes are grouped together based on their mapping profiles. This approach avoids penalizing tools for inevitable redistribution within such groups and provides a more biologically meaningful measure of performance. Unlike conventional clustering by ANI, our method groups references according to shared mapping profiles, focusing on patterns reflected in the data rather than static reference similarity. This design supports the concept of sample-aware reference groups that better capture functional and ecological relationships and could enhance classification accuracy in the presence of closely related organisms. Such clustering could also guide adaptive reference construction or real-time database refinement as additional samples are analyzed. Although clustering was used only for evaluation in this study and applied uniformly across all tools, future work will include deeper investigation of this method and its integration into the full MADRe pipeline.

As MADRe relies on metagenome assembly for database reduction, its performance can be influenced by assembly quality, particularly in highly complex or low-coverage samples. To assess sensitivity to assembler choice, we evaluated MADRe on large simulated datasets using Myloasm in addition to metaFlye and metaMDBG. Overall performance trends were comparable across assemblers, suggesting that MADRe is not strongly dependent on a specific assembly tool. We observed complementary behavior, with Myloasm showing improved detection of low-abundance strains and metaFlye performing slightly better for highly abundant ones. Strain-level assemblers were not considered, as they typically generate shorter and more fragmented contigs, whereas MADRe’s reduction algorithm benefits from longer genomic context. While different parameter settings may influence assembly quality, all assemblers were executed using recommended configurations, and extensive parameter tuning was beyond the scope of this study.

Beyond strain-level classification, MADRe’s modular design, particularly its database reduction and probabilistic reassignment components, offers potential for broader applications. These include contig binning, assembly refinement, and functional gene profiling, where confident reference reduction and ambiguity-aware read handling are equally valuable.

## Conclusion

In this study, we introduced MADRe, a novel pipeline for strain-level metagenomic classification of long-read sequencing data. MADRe combines long-read assembly, EM-based contig-to-reference mapping reassignment for database reduction, and probabilistic read reassignment to deliver accurate and efficient classification, even without prior knowledge of sample composition. Unlike many existing tools, MADRe is designed to operate with large, diverse databases spanning multiple taxonomic levels, enabling high-resolution classification while minimizing false positives.

The pipeline consists of two distinct steps: database reduction and read classification, both of which can be executed independently. If general insight into the strains present in a sample is required, the first step can be used alone. Conversely, when prior knowledge about the sample exists, or when a reduced reference set is already available, the read classification step can be applied independently. MADRe provides a practical, scalable, and modular solution for strain-level classification in complex microbial communities.

## Methods

### MADRe database reduction

The database reduction step, shown in Figure 1 and illustrated in more detail in Supplementary Fig. S1, consists of two main phases: input file preparation and the database reduction.

In the input file preparation phase, raw long metagenomic reads are first assembled using metaFlye for ONT reads or metaMDBG for HiFi reads. When multiple strains of the same species are present in a sample, the assembly process can lead to strain collapse, producing contigs that represent a blend of closely related strains rather than distinct strain-specific sequences. Instead of using strain-aware metagenome assemblers, which typically generate shorter contigs, we chose to retain the longer contigs and infer strain-level complexity using HairSplitter functionality, which estimates the number of collapsed strains per contig.

Assembled contigs are mapped to the reference database using Minimap2 with the *asm5* parameter preset, generating a PAF file as output. We chose this preset because, compared to *asm10* and *asm20*, it provides higher sensitivity, which is crucial for capturing more accurate and complete alignments of contigs to highly similar reference genomes. The MADRe database reduction process takes two key inputs: the estimated number of collapsed strains per contig determined by HairSplitter and the contig-to-reference mappings from Minimap2.

The database reduction process is based on the EM algorithm, which reassigns contigs to different references while performing soft clustering, allowing a single contig to be assigned to multiple references with different probabilities. The EM algorithm is widely used for handling ambiguous mappings in metagenomic classification [29–32, 45, 69]. The implementation of the EM algorithm in MADRe is inspired by PathoScope2 [30] and EMU [31].

The database reduction process consists of three main steps. We can define a set of contigs as $C = \lbrace c_1, c_2, \dots , c_x\rbrace $, where $x$ is the number of contigs in the assembly. The set of references to which at least one contig is mapped is defined as $R = \lbrace r_1, r_2, \dots , r_g\rbrace $, where $g$ is the number of references. Additionally, let $M$ represent the set of all of the mappings. In the first step, we compute a mapping score ${H}$ for each mapping in the PAF file using the equation:


(1) \begin{eqnarray*}
{H} = 2*\frac{{N} \times ml}{{N} + ml},
\end{eqnarray*}

which represents the harmonic mean between the exact number of matches ${N}$ and the mapping length $ml$. The $ml$ is defined as the maximum value between the query mapping length and the reference mapping length. Applying the harmonic mean allows us to emphasize the smaller value, ensuring that a mapping does not receive an inflated score due, e.g., to a very long but low-quality alignment.

The summarized mapping value ${S}$ is then calculated for each contig-reference pair using:


(2) \begin{eqnarray*}
{S}(c_i, r_j) = \sum _{m \in M(c_i, r_j)} {H}(p) ,
\end{eqnarray*}

where $M(c_i, r_j)$ represents the set of mappings of contig $c_i$ to reference $r_j$. This ensures that $\mathrm{S}(c_i, r_j)$ is computed by summing the mapping values of all instances where $c_i$ maps to $r_j$, thus capturing all possible alignments between the contig and the reference. Following this, we divided mappings into *unique* and *non-unique*. Unique mappings occur when a contig maps exclusively to a single reference, whereas non-unique mappings represent ambiguous cases that require further resolution.

In the second step, non-unique mappings are processed using the EM algorithm, which iteratively refines contig assignments based on mapping probabilities. The E-step updates the expected assignments of contigs, while the M-step re-estimates the parameters using the newly computed assignment probabilities from the previous iteration.

The probability of selecting a reference $r_i$ is given by


(3) \begin{eqnarray*}
P(r_i) = \frac{1}{G}, \quad \mathrm{where} \quad G = |R|.
\end{eqnarray*}

The conditional probability of $c_i$ given $r_i$ is expressed as


(4) \begin{eqnarray*}
P(c_i | r_i) = \frac{\mathrm{S}(c_i, r_i)}{\max _{r_j \in R} \mathrm{S}(c_i, r_j)}.
\end{eqnarray*}

The log-likelihood function $L(X)$ is given by


(5) \begin{eqnarray*}
L(C) = \sum _{i=1}^{X} \log \left( \sum _{j=1}^{G} P(c_i \mid r_j) \cdot P(r_j) \right).
\end{eqnarray*}

The expectation step (E-step) updates the posterior probability $P(r_i \mid c_i)$ as follows:


(6) \begin{eqnarray*}
P(r_i \mid c_i) = \frac{P(c_i \mid r_i) \cdot P(r_i)}{\sum _{j=1}^{G} P(c_i \mid r_j) \cdot P(r_j)}.
\end{eqnarray*}

The maximization step (M-step) updates the prior probability $P(r_i)$ as follows:


(7) \begin{eqnarray*}
P(r_i) = \frac{\sum _{j=1}^{X} P(r_i \mid c_j)}{|M|}.
\end{eqnarray*}

The EM algorithm runs iteratively until it converges or reaches the maximum number of iterations set by the stopping criteria. Once the algorithm outputs posterior probabilities, these values are used to determine which references will be included in the reduced database.

Before selecting references, we first classify each contig at the species level. This is done by summing the posterior probabilities across all references belonging to a species and assigning the contig to the species with the highest total probability. After determining the species classification, we retain only posterior probabilities associated with references belonging to the selected species. Finally, for each contig, we select *N* + 2 reference genomes to include in the reduced database. The value of *N* is estimated based on the number of collapsed strains identified by HairSplitter. By default, MADRe adds two additional references to avoid excluding expected strains, although this offset can be adjusted through user parameters.

### MADRe read classification

The read classification step in MADRe is designed to operate both with and without prior database reduction. The only requirement is a PAF file, the Minimap2 output containing read-to-database mappings, where each database sequence includes the corresponding taxIDs. The MADRe read classification workflow is illustrated in Supplementary Fig. S2.

The process begins by computing a mapping score for each alignment, defined as the ratio between the number of exact matches (*N*) and the mapping length ($ml$):


\begin{eqnarray*}
S = \frac{N}{ml}.
\end{eqnarray*}

Mappings are then categorized into unique and non-unique. Since a read can have multiple alignments, only the alignment with the highest score is retained for each read-reference pair. If a read has a single best-scoring mapping, it is classified as a unique mapping. Conversely, if multiple mappings share the same highest score, they are considered non-unique, and the read will go through a reassignment process. Formally,


\begin{eqnarray*}
\text{Unique if } S_{r,i} = \max _j(S_{r,j}) \text{ and this maximum is unique;}
\end{eqnarray*}

\begin{eqnarray*}
\text{Non-unique if } S_{r,i} = \max _j(S_{r,j}) \text{ for two or more } j.
\end{eqnarray*}

Before reassigning non-unique mappings, reads are first classified at the species level. For each read *r*, the maximum mapping score among all references belonging to a species *s* is computed as


\begin{eqnarray*}
S_{r,s}^{\max } = \max _{i \in s} (S_{r,i}).
\end{eqnarray*}

The species with the highest $S_{r,s}^{\max }$ is selected as the species-level assignment for that read. Although the default assumption is that a read can be uniquely mapped to a single species but may map ambiguously to multiple strains within that species, this assumption does not always hold. In rare cases, two genomes from different species may share highly similar regions, making it difficult to determine the true origin of a read. In such situations, reads are randomly distributed between the corresponding species. These cases are uncommon and typically arise from taxonomic inconsistencies, e.g., when nearly identical strains according to taxonomy belong to different species.

To reassign non-unique mappings at the strain level, a species-specific clustering algorithm is applied. This algorithm evaluates the number of unique and non-unique mappings associated with each reference within the same species, identifying groups of references that share many mappings, indicating that they likely represent overlapping genomic regions and should form clusters.

The fundamental assumption is that a reference truly present in the sample should accumulate the highest number of mappings (both unique and high-confidence non-unique). Let $M_i$ denote the total number of mappings to reference *i*:


\begin{eqnarray*}
M_i = U_i + N_i,
\end{eqnarray*}

where $U_i$ and $N_i$ represent the counts of unique and non-unique mappings, respectively. The expected references are identified as those with the highest $M_i$ within each cluster, and all non-unique reads are reassigned to the most probable reference in that cluster:


\begin{eqnarray*}
r \in \mathrm{cluster}(i) \Rightarrow r \rightarrow \arg \max _{j \in \mathrm{cluster}(i)} M_j .
\end{eqnarray*}

This procedure ensures that ambiguous reads are redistributed toward references that are both well supported by unique evidence and consistent with the mapping structure observed across the sample. By reassigning reads to the most representative reference within each cluster, this methodology establishes MADRe’s centroid-based behavior, maintaining stable and interpretable classifications even in the presence of highly similar strains.

#### Abundances calculation

At this stage, each read is assigned to a single reference genome. Based on these assignments, MADRe calculates the abundance of each detected strain. The primary abundance output file reports the number of reads assigned to each reference. However, MADRe also provides an option to compute a length-normalized abundance, which accounts for both read and reference lengths. This alternative abundance metric is calculated as


(8) \begin{eqnarray*}
\mathrm{Abundance}(r) = \frac{\sum \limits _{i \in \mathrm{Reads}_r} Length(i)}{\mathrm{Length}(r)} \quad \text{for } r \in R ,
\end{eqnarray*}

where *R* is the set of the references and $Reads_r$ is set of the reads classified under reference *r*.

#### Similar strains clustering

The high similarity between closely related strains and the lack of a clear threshold for defining when two sequences represent the same strain make it difficult to ensure that a reference database contains only unique strain sequences [34]. Some entries may correspond to highly similar strains or even to multiple assemblies of the same strain. To address this, MADRe includes an optional reference clustering step within the read classification process, designed to group similar references based on shared read mappings.

This clustering step operates on the same mapping file used in the classification stage and produces two output files: one reporting the abundances of identified clusters and the other listing the representative reference for each cluster.

The clustering procedure is illustrated in Supplementary Fig. S3. It begins by using the calculated mapping scores and species-level labels. For each species-specific subset, the algorithm identifies the highest-quality mapping for each read. A binary vector is constructed for each reference, where each bit indicates whether a given read strongly supports that reference. These binary vectors are then clustered using DBSCAN with precomputed Jaccard distances, eps = 0.9, and min_samples = 1. Cluster-level abundances are computed accordingly. As a result, the post-clustering abundance files report only the representative references for each cluster.

### Evaluation details

Our evaluation is primarily focused on exact read-level taxonomic assignments and read count-based abundance estimates.

To ensure a fair comparison, all tools were benchmarked using the same large reference database. For tools requiring mapping files as input, namely MORA, AugPatho, and MADRe_RC, we used a unified set of read-to-reference alignments generated by Minimap2. All reads were mapped to the full database, and the resulting SAM file was used directly for MORA and AugPatho. This SAM file was subsequently converted to PAF format using Paftools, as required by MADRe_RC.

Simulated reads were generated with the Badread tool [56], applying the whole-metagenome simulation mode with default values for chimeric, junk, and random reads. The simulation was performed using the *nanopore2023* model, which corresponds to the ONT R10.4.1. The exact command used is provided in the Supplementary Material.

In case of simulated datasets, ground truth was available for every read, including its corresponding strain-level taxID and reference accession. Using this information, we computed TP, FP, TN, and FN by comparing the assigned strain-level taxIDs with the expected ones. For Kraken2, we extracted read IDs and assigned taxIDs from its output. If a read was assigned to a higher taxonomic level, even if it was taxonomic correct, we treated it as a false positive, as the evaluation strictly focused on strain-level classification.

For the simulated datasets and Zymo mock communities we calculated BC distance as


(9) \begin{eqnarray*}
\mathrm{BC}(x, y) = \frac{\sum _{i=1}^{n} |x_i - y_i|}{\sum _{i=1}^{n} (x_i + y_i)} ,
\end{eqnarray*}

where $x = (x_1, x_2, \dots , x_n)$ and $y = (y_1, y_2, \dots , y_n)$ are the abundance vectors for two samples or profiles, $n$ is the number of strains, $x_i$ and $y_i$ are the abundances of the $i{\mathrm{th}}$ strain in samples $x$ and $y$, respectively. $\mathrm{BC}(x, y)$ is the BC dissimilarity or distance, ranging from 0 (identical composition) to 1 (completely disjoint).

In the case of medium-sized simulated datasets (sim_small, sim_medium and sim_expanded) and the real dataset, the evaluation was also performed at the species level (results presented in Supplementary Table S8 and Figure S7). All strain-level classifications were uplifted to their corresponding species, and read count abundances were calculated. For Kraken2, we used the species-level abundances reported in its summary file, limited to entries labeled with an “S” (species rank).

MADRe, MADRe_RC, and MORA each produce read-level classification files that associate each read with a reference genome. Since all tools shared the same mapping input, for AugPatho, we ran PathoID and PathoREPORT steps, which output an updated SAM file and a report containing reference abundance estimates. In these updated SAM files, a single read can be associated with multiple references. For evaluation purposes, we allowed such multi-reference assignments, which may slightly benefit AugPatho by increasing the number of TP, while also increasing the risk of false positives. These trade-offs are largely neutralized when clustering is applied, as similar strains typically end up grouped in the same cluster. With Centrifuger we encountered one limitation—Centrifuger cannot confidently assign a read to a specific reference sequence (e.g., when multiple chromosomes belong to the same strain), it often classifies the read under the NCBI strain-level taxid. In some cases, this strain taxid is identical to the species taxid, making it impossible to directly and fairly compare such classifications to those of other tools that operate at the sequence level. For benchmarking consistency, we therefore considered as TP only the reads correctly classified under the expected reference sequence. It is important to note that this issue affected a relatively small fraction of reads (~9,000 out of  5 million reads in the 1,000-genome dataset).

We used the same clustering across all tools to ensure consistency in cluster-based evaluation. Our clustering is based on read-to-reference mapping profiles, which can differ depending on the size and composition of the database. For example, when reads are mapped to a reduced database, the absence of certain similar references can make ambiguous mappings more resolvable. To avoid such inconsistencies, clustering was performed only once on the PAF file used for MADRe_RC, which contains read mappings to the complete reference database. During cluster-level evaluation, a classification was considered a TP if the read was assigned to a reference that belongs to the same cluster as the ground truth reference:


\begin{eqnarray*}
\mathrm{TP} = \left\lbrace \begin{array}{@{}l@{\quad }l@{}}1, & \text{if } C(\hat{r}) = C(r_{\mathrm{true}}) \\0, & \mathrm{otherwise} \end{array}\right.,
\end{eqnarray*}

where $C(\hat{r})$ denotes the cluster of the predicted reference and $C(r_{\mathrm{true}})$ denotes the cluster of the true reference. A classification is considered a TP if both references belong to the same cluster.

All commands used to perform classification with the evaluated tools are listed in the Supplementary Material.

## Availability of source code and requirements

- Project name: MADRe—Metagenome Assembly driven Database Reduction
- Project home page:  https://github.com/lbcb-sci/MADRe
- Operating system(s): UNIX
- Programming language: Python
- Other requirements: Environment Modules, Conda, see  https://github.com/lbcb-sci/MADRe
- License: MIT

## Supplementary Material

giag030_Supplemental_Files

giag030_Authors_Response_To_Reviewer_Comments_original_submission

giag030_GIGA-D-25-00468_original_submission

giag030_GIGA-D-25-00468_Revision_1

giag030_Reviewer_1_Report_original_submission Reviewer 1 -- 11/19/2025

giag030_Reviewer_2_Report_original_submission Reviewer 2 -- 1/23/2026

giag030_Reviewer_2_Report_revision_1 Reviewer 2 -- 3/8/2026

## Data Availability

The source code for MADRe is available at [70]. Simulated data can be accessed via Zenodo [71]. Zymo D6322 ONT dataset is obtained from BioProject PRJNA1240873, zymo D6331 ONT dataset is obtained from [58], and zymo D6331 PacBio HiFi dataset from [59].

## Additional files

**Supplementary Fig. S1**: MADRe Database Reduction pipeline. The process begins with assembling long reads using metaFlye or metaMDBG. HairSplitter is then applied to estimate the number of collapsed strains per contig. The resulting contigs are mapped to the reference database, and the mapping information is stored in a PAF file. Organism identification within the reduced database is performed using an expectation–maximization (EM) algorithm that integrates both the mapping data and the estimated number of collapsed strains. The EM algorithm terminates after 25 iterations or when the change in pi values between two consecutive iterations (epsilon) falls <0.0001. The number of collapsed strains estimated by HairSplitter is, by default, extended by two in MADRe, although this value can be adjusted through MADRe’s parameters.

**Supplementary Fig. S2**: MADRe Read Classification pipeline. Reads are mapped to the (reduced) reference database, and the mapping information is stored in a PAF file. Based on mapping scores, reads are first grouped at the species level. Within each species, read assignments are further refined through a mapping-based probability reassignment procedure, which analyzes both unique and non-unique mapping profiles for each reference. In the final step, non-uniquely mapped reads are assigned to the reference with the highest number of unique and high-confidence non-unique mappings, as it is considered the most probable representative. Finally, reference-level abundances are computed.

**Supplementary Fig. S3**: MADRe Clustering pipeline. Reads are mapped to the reference database, and the mapping information is stored in a PAF file. Based on mapping scores, reads are first grouped by species. For each species, every reference is represented by a binary vector, where each position corresponds to a read: a value of 1 indicates a high-quality mapping between the read and the reference, and 0 otherwise. These binary vectors are then used as input for DBSCAN clustering to group highly similar references (eps = 0.9).

**Supplementary Fig. S4**: Bray–Curtis distances for medium-sized simulated datasets. The plots show BC distance between predicted and expected abundances in simulated datasets (sim_small, sim_medium, sim_extended) with and without post-clustering of similar strains. Since Kraken2 outputs taxID labels as classification labels, the clustering was not performed with it.

**Supplementary Fig. S5**: Bray–Curtis distances for large-sized simulated datasets. The plots show BC distance between predicted and expected abundances in simulated datasets (sim_low_r9, sim_low_r10, sim_low_hifi and sim_high_r10) with and without post-clustering of similar strains. Since Kraken2 outputs taxID labels as classification labels, the clustering was not performed with it.

**Supplementary Fig. S6**: Bray–Curtis distances for Zymo datasets with clustering of similar strains. The plots show the BC distance between ground-truth read counts and classified read counts after post-classification clustering of similar strains. The solid line represents distances calculated using all classified reads, while the dotted line represents distances calculated using only true-positive classifications.

**Supplementary Fig. S7**: Anaerobic Digester Sludge metagenome species-level abundances of top-20 most abundant species for every of the tools. MORA’s and Centrifuger’s top 20 species account for the lowest cumulative abundance, suggesting its assignments are more broadly spread across a larger number of species.

**Supplementary Table S1**: Information about organisms used for data simulation of medium-sized datasets.

**Supplementary Table S2**: fastANI similarities between reference genomes from sim_small dataset.

**Supplementary Table S3**: fastANI similarities between reference genomes from sim_medium dataset. Note: Although.

**Supplementary Table S4**: fastANI similarities between reference genomes from sim_expanded dataset. Note: Although fastANI can output values <80, such scores are generally considered unreliable due to high estimation error. ANI values between 70 and 80% have substantially greater uncertainty compared to those in the 80–100% range.

**Supplementary Table S5**: Reported read count for simulated dataset simsmall.

**Supplementary Table S6**: Reported read count for simulated dataset sim_medium.

**Supplementary Table S7**: Reported read count for simulated dataset sim_expanded.

**Supplementary Table S8**: Number of TP/TN/FP/FN, accuracy, precision, recall and F1 for medium-sized simulated.

**Supplementary Table S9**: Bray–Curtis distances for medium-sized simulated datasets with and without post-clustering applied.

**Supplementary Table S10**: Number of genome pairs with average nucleotide identity (ANI) values above the indicated thresholds for large-sized simulated datasets.

**Supplementary Table S11**: Reported read count for large-sized simulated dataset sim_low_ontR9.

**Supplementary Table S12**: Reported read count for large-sized simulated dataset sim_low_ontR10.

**Supplementary Table S13**: Reported read count for large-sized simulated dataset sim_low_hifi.

**Supplementary Table S14**: Reported read count for large-sized simulated dataset sim_high_ont (R10).

**Supplementary Table S15**: Number of TP/TN/FP/FN, accuracy, precision, recall and F1 for large-sized simulated datasets on strain level (with and without clustering).

**Supplementary Table S16**: Bray-Curtis distances for large-sized simulated datasets with and without post-clustering applied.

**Supplementary Table S17**: Read counts for expected bacteria strains for zymo D6322.

**Supplementary Table S18**: Read counts for expected bacteria strains for zymo D6331.

**Supplementary Table S19**: Read counts for expected bacteria strains for zymo D6331.

**Supplementary Table S20**: Sequence-level identification statistics in Zymo datasets for different tools. Each identification corresponds to a single sequence expected in the Zymo datasets, including distinct genome components (e.g., in Prevotella corporis) or plasmids. The slightly lower recall observed for MADRe can be attributed to low-abundance strains. For instance, Clostridium perfringens in Zymo D6331, the least abundant strain in the dataset, which was not expected to be identified by MADRe, contains five plasmids.

**Supplementary Table S21**: Read counts anaerobic digester sludge metagenome - * represents that reference is the representative of the cluster with more than one reference.

**Supplementary Table S22**: CPU time and memory usage for running zymo D6331 ONT ( 1.7M reads) and HiFi ( 2M reads), and large-sized sim_high ( 5M reads) simulated datasets. All tests ran using 64 CPUs.

**Supplementary Table S23**: Similar strains experiments. List of genomes in datasets, ANI values and read counts in case of different experiments.

**Supplementary Table S24**: Coverage and similarity experiment

## Abbreviations

ANI: average nucleotide identity; BC: Bray–Curtis; EM: expectation–maximization; FP: false positive; FN: false negative; ONT: Oxford Nanopore Technologies; taxID: taxonomy identifier; TP: true positive; TN: true negative.
