# Supplementary material for: MADRe: Strain-level metagenomic classification through assembly-driven database reduction
Source: Gigascience. 2026 Mar 23;15:giag030. doi: 10.1093/gigascience/giag030 (PMC13211987; doi:10.1093/gigascience/giag030)
Supplement: giag030_GIGA-D-25-00468_original_submission [file giag030_giga-d-25-00468_original_submission.pdf]

GigaScience

MADRe: Strain-Level Metagenomic Classification Through Assembly-Driven Database Reduction

--Manuscript Draft--

**Manuscript Number:**	GIGA-D-25-00468
**Full Title:**	MADRe: Strain-Level Metagenomic Classification Through Assembly-Driven Database Reduction
**Article Type:**	Technical Note
**Funding Information:**	Hrvatska Zaklada za Znanost (IP-2018-01-5886) — prof.dr.sc. Mile Šikić<br>Hrvatska Zaklada za Znanost (MOBDOK-2023-2941) — Ms Josipa Lipovac<br>Singapore Ministry of Health’s National Medical Research Council (MOH-000649-01) — prof.dr.sc. Mile Šikić
**Abstract:**	Strain-level metagenomic classification is essential for understanding microbial diversity and functional potential, but remains challenging, particularly in the absence of prior knowledge about the composition of the sample. In this paper we present MADRe, a modular and scalable pipeline for long-read strain-level metagenomic classification, enhanced with Metagenome Assembly-Driven Database Reduction. MADRe combines long-read metagenome assembly, contig-to-reference mapping reassignment based on an expectation-maximization algorithm for database reduction, and probabilistic read mapping reassignment to achieve sensitive and precise classification. We extensively evaluated MADRe on simulated datasets, mock communities, and a real anaerobic digester sludge metagenome, demonstrating that it consistently outperforms existing tools by achieving higher precision with reduced false positives. MADRe's design allows users to apply either the database reduction or read classification step individually. Using only the read classification step shows results on par with other tested tools. MADRe is open source and publicly available at https://github.com/lbcb-sci/MADRe.
**Corresponding Author:**	Josipa Lipovac<br>University of Zagreb Faculty of Electrical Engineering and Computing: Sveuciliste u Zagrebu Fakultet Elektrotehnike i Racunarstva<br>Zagreb, CROATIA
**Corresponding Author Secondary Information:**	
**Corresponding Author's Institution:**	University of Zagreb Faculty of Electrical Engineering and Computing: Sveuciliste u Zagrebu Fakultet Elektrotehnike i Racunarstva
**Corresponding Author's Secondary Institution:**	
**First Author:**	Josipa Lipovac
**First Author Secondary Information:**	
**Order of Authors:**	Josipa Lipovac<br>Mile Šikić<br>Riccardo Vicedomini<br>Krešimir Križanović
**Order of Authors Secondary Information:**	
**Additional Information:**	
**Question**	**Response**
Are you submitting this manuscript to a	No

Powered by Editorial Manager® and ProduXion Manager® from Aries Systems Corporation

special series or article collection?	
**Experimental design and statistics**<br><br>Full details of the experimental design and statistical methods used should be given in the Methods section, as detailed in our Minimum Standards Reporting Checklist. Information essential to interpreting the data presented should be made available in the figure legends.<br><br>Have you included all the information requested in your manuscript?	Yes
**Resources**<br><br>A description of all resources used, including antibodies, cell lines, animals and software tools, with enough information to allow them to be uniquely identified, should be included in the Methods section. Authors are strongly encouraged to cite Research Resource Identifiers (RRIDs) for antibodies, model organisms and tools, where possible.<br><br>Have you included the information requested as detailed in our Minimum Standards Reporting Checklist?	Yes
**Availability of data and materials**<br><br>All datasets and code on which the conclusions of the paper rely must be either included in your submission or deposited in publicly available repositories (where available and ethically appropriate), referencing such data using a unique identifier in the references and in the "Availability of Data and Materials" section of your manuscript.<br><br>Have you have met the above requirement as detailed in our Minimum Standards Reporting Checklist?	Yes

GigaScience has policies and guidelines in place for the use of generative AI-writing tools such as ChatGPT. If you have used such writing tools to assist with writing the manuscript this must be declared and cited in the text. Authors should not list AI-writing tools and other AI-assisted technologies as an author or co-author and should acknowledge that they are fully responsible for text generated or refined by AI-writing tools.<p><br><br>A summary of use (particularly in the introduction or among methods) needs to be included at the end of the paper, and the outputs should also be included as a supplementary file hosted in GigaDB or other open repositories. Please read our guidelines for more information. <p><br><br>By submitting to GigaScience, you are aware of the journal's AI-writing tools policy, and if you have declared use of such tools below, you have acknowledged this where appropriate in your manuscript and have made a summary of use and outputs available. <p><br><b>AI-assisted writing tools have been used in the preparation of this manuscript?	No

Main latex file Click here to access/download;LaTeX - Main Document (TeX file);MADRe_main.tex

```
This is pdfTeX, Version 3.141592653-2.6-1.40.26 (TeX Live 2024) 
(preloaded format=pdflatex 2024.8.2)  6 NOV 2025 10:35
entering extended mode
 restricted \write18 enabled.
 %&-line parsing enabled.
**madre_main.tex
(./MADRe_main.tex
LaTeX2e <2024-06-01> patch level 2
L3 programming layer <2024-05-27>
(./oup-contemporary.cls
Document Class: oup-contemporary 2023/06/12, v1.2
(c:/texlive/2024/texmf-dist/tex/latex/base/article.cls
Document Class: article 2024/02/08 v1.4n Standard LaTeX document class
(c:/texlive/2024/texmf-dist/tex/latex/base/size10.clo
File: size10.clo 2024/02/08 v1.4n Standard LaTeX file (size option)
)
\c@part=\count194
\c@section=\count195
\c@subsection=\count196
\c@subsubsection=\count197
\c@paragraph=\count198
\c@subparagraph=\count199
\c@figure=\count266
\c@table=\count267
\abovecaptionskip=\skip49
\belowcaptionskip=\skip50
\bibindent=\dimen141
) (c:/texlive/2024/texmf-dist/tex/latex/base/inputenc.sty
Package: inputenc 2024/02/08 v1.3d Input encoding file
\inpenc@prehook=\toks17
\inpenc@posthook=\toks18
) (c:/texlive/2024/texmf-dist/tex/latex/base/fontenc.sty
Package: fontenc 2021/04/29 v2.0v Standard LaTeX package
) (c:/texlive/2024/texmf-dist/tex/generic/iftex/ifpdf.sty
Package: ifpdf 2019/10/25 v3.4 ifpdf legacy package. Use iftex instead.
(c:/texlive/2024/texmf-dist/tex/generic/iftex/iftex.sty
Package: iftex 2022/02/03 v1.0f TeX engine tests
)) (c:/texlive/2024/texmf-dist/tex/latex/microtype/microtype.sty
Package: microtype 2024/03/29 v3.1b Micro-typographical refinements (RS)
(c:/texlive/2024/texmf-dist/tex/latex/graphics/keyval.sty
Package: keyval 2022/05/29 v1.15 key=value parser (DPC)
\KV@toks@=\toks19
) (c:/texlive/2024/texmf-dist/tex/latex/etoolbox/etoolbox.sty
Package: etoolbox 2020/10/05 v2.5k e-TeX tools for LaTeX (JAW)
\etb@tempcnta=\count268
)
\MT@toks=\toks20
\MT@tempbox=\box52
\MT@count=\count269
LaTeX Info: Redefining \noprotrusionifhmode on input line 1061.
LaTeX Info: Redefining \leftprotrusion on input line 1062.
\MT@prot@toks=\toks21
LaTeX Info: Redefining \rightprotrusion on input line 1081.
LaTeX Info: Redefining \textls on input line 1392.
```

```
\MT@outer@kern=\dimen142
LaTeX Info: Redefining \textmicrotypecontext on input line 2013.
\MT@listname@count=\count270
(c:/texlive/2024/texmf-dist/tex/latex/microtype/microtype-pdftex.def
File: microtype-pdftex.def 2024/03/29 v3.1b Definitions specific to
pdftex (RS)

LaTeX Info: Redefining \lsstyle on input line 902.
LaTeX Info: Redefining \lslig on input line 902.
\MT@outer@space=\skip51
)
Package microtype Info: Loading configuration file microtype.cfg.
(c:/texlive/2024/texmf-dist/tex/latex/microtype/microtype.cfg
File: microtype.cfg 2024/03/29 v3.1b microtype main configuration file
(RS)
)) (c:/texlive/2024/texmf-dist/tex/latex/euler/euler.sty
Package: euler 1995/03/05 v2.5
Package: `euler' v2.5 <1995/03/05> (FJ and FMi)
LaTeX Font Info:    Redeclaring symbol font `letters' on input line 35.
LaTeX Font Info:    Encoding `OML' has changed to `U' for symbol font
(Font)              `letters' in the math version `normal' on input line
35.
LaTeX Font Info:    Overwriting symbol font `letters' in version `normal'
(Font)                  OML/cmm/m/it --> U/eur/m/n on input line 35.
LaTeX Font Info:    Encoding `OML' has changed to `U' for symbol font
(Font)              `letters' in the math version `bold' on input line
35.
LaTeX Font Info:    Overwriting symbol font `letters' in version `bold'
(Font)                  OML/cmm/b/it --> U/eur/m/n on input line 35.
LaTeX Font Info:    Overwriting symbol font `letters' in version `bold'
(Font)                  U/eur/m/n --> U/eur/b/n on input line 36.
LaTeX Font Info:    Redeclaring math symbol \Gamma on input line 47.
LaTeX Font Info:    Redeclaring math symbol \Delta on input line 48.
LaTeX Font Info:    Redeclaring math symbol \Theta on input line 49.
LaTeX Font Info:    Redeclaring math symbol \Lambda on input line 50.
LaTeX Font Info:    Redeclaring math symbol \Xi on input line 51.
LaTeX Font Info:    Redeclaring math symbol \Pi on input line 52.
LaTeX Font Info:    Redeclaring math symbol \Sigma on input line 53.
LaTeX Font Info:    Redeclaring math symbol \Upsilon on input line 54.
LaTeX Font Info:    Redeclaring math symbol \Phi on input line 55.
LaTeX Font Info:    Redeclaring math symbol \Psi on input line 56.
LaTeX Font Info:    Redeclaring math symbol \Omega on input line 57.
\symEulerFraktur=\mathgroup4
LaTeX Font Info:    Overwriting symbol font `EulerFraktur' in version
`bold'
(Font)                  U/euf/m/n --> U/euf/b/n on input line 63.
LaTeX Info: Redefining \oldstylenums on input line 85.
\symEulerScript=\mathgroup5
LaTeX Font Info:    Overwriting symbol font `EulerScript' in version
`bold'
(Font)                  U/eus/m/n --> U/eus/b/n on input line 93.
LaTeX Font Info:    Redeclaring math symbol \aleph on input line 97.
LaTeX Font Info:    Redeclaring math symbol \Re on input line 98.
LaTeX Font Info:    Redeclaring math symbol \Im on input line 99.
```

```
LaTeX Font Info:    Redeclaring math delimiter \vert on input line 101.
LaTeX Font Info:    Redeclaring math delimiter \backslash on input line
103.
LaTeX Font Info:    Redeclaring math symbol \neg on input line 106.
LaTeX Font Info:    Redeclaring math symbol \wedge on input line 108.
LaTeX Font Info:    Redeclaring math symbol \vee on input line 110.
LaTeX Font Info:    Redeclaring math symbol \setminus on input line 112.
LaTeX Font Info:    Redeclaring math symbol \sim on input line 113.
LaTeX Font Info:    Redeclaring math symbol \mid on input line 114.
LaTeX Font Info:    Redeclaring math delimiter \arrowvert on input line
116.
LaTeX Font Info:    Redeclaring math symbol \mathsection on input line
117.
\symEulerExtension=\mathgroup6
LaTeX Font Info:    Redeclaring math symbol \coprod on input line 125.
LaTeX Font Info:    Redeclaring math symbol \prod on input line 125.
LaTeX Font Info:    Redeclaring math symbol \sum on input line 125.
LaTeX Font Info:    Redeclaring math symbol \intop on input line 130.
LaTeX Font Info:    Redeclaring math symbol \ointop on input line 131.
LaTeX Font Info:    Redeclaring math symbol \braceld on input line 132.
LaTeX Font Info:    Redeclaring math symbol \bracerd on input line 133.
LaTeX Font Info:    Redeclaring math symbol \bracelu on input line 134.
LaTeX Font Info:    Redeclaring math symbol \braceru on input line 135.
LaTeX Font Info:    Redeclaring math symbol \infty on input line 136.
LaTeX Font Info:    Redeclaring math symbol \nearrow on input line 153.
LaTeX Font Info:    Redeclaring math symbol \searrow on input line 154.
LaTeX Font Info:    Redeclaring math symbol \nwarrow on input line 155.
LaTeX Font Info:    Redeclaring math symbol \swarrow on input line 156.
LaTeX Font Info:    Redeclaring math symbol \Leftrightarrow on input line
157.
LaTeX Font Info:    Redeclaring math symbol \Leftarrow on input line 158.
LaTeX Font Info:    Redeclaring math symbol \Rightarrow on input line
159.
LaTeX Font Info:    Redeclaring math symbol \leftrightarrow on input line
160.
LaTeX Font Info:    Redeclaring math symbol \leftarrow on input line 161.
LaTeX Font Info:    Redeclaring math symbol \rightarrow on input line
163.
LaTeX Font Info:    Redeclaring math delimiter \uparrow on input line
166.
LaTeX Font Info:    Redeclaring math delimiter \downarrow on input line
168.
LaTeX Font Info:    Redeclaring math delimiter \updownarrow on input line
170.
LaTeX Font Info:    Redeclaring math delimiter \Uparrow on input line
172.
LaTeX Font Info:    Redeclaring math delimiter \Downarrow on input line
174.
LaTeX Font Info:    Redeclaring math delimiter \Updownarrow on input line
176.
LaTeX Font Info:    Redeclaring math symbol \leftharpoonup on input line
177.
LaTeX Font Info:    Redeclaring math symbol \leftharpoondown on input
line 178.
```

```
LaTeX Font Info:    Redeclaring math symbol \rightharpoonup on input line
179.
LaTeX Font Info:    Redeclaring math symbol \rightharpoondown on input
line 180
.
LaTeX Font Info:    Redeclaring math delimiter \lbrace on input line 182.
LaTeX Font Info:    Redeclaring math delimiter \rbrace on input line 184.
\symcmmigroup=\mathgroup7
LaTeX Font Info:    Overwriting symbol font `cmmigroup' in version `bold'
(Font)                  OML/cmm/m/it --> OML/cmm/b/it on input line 200.
LaTeX Font Info:    Redeclaring math accent \vec on input line 201.
LaTeX Font Info:    Redeclaring math symbol \triangleleft on input line
202.
LaTeX Font Info:    Redeclaring math symbol \triangleright on input line
203.
LaTeX Font Info:    Redeclaring math symbol \star on input line 204.
LaTeX Font Info:    Redeclaring math symbol \lhook on input line 205.
LaTeX Font Info:    Redeclaring math symbol \rhook on input line 206.
LaTeX Font Info:    Redeclaring math symbol \flat on input line 207.
LaTeX Font Info:    Redeclaring math symbol \natural on input line 208.
LaTeX Font Info:    Redeclaring math symbol \sharp on input line 209.
LaTeX Font Info:    Redeclaring math symbol \smile on input line 210.
LaTeX Font Info:    Redeclaring math symbol \frown on input line 211.
LaTeX Font Info:    Redeclaring math accent \grave on input line 245.
LaTeX Font Info:    Redeclaring math accent \acute on input line 246.
LaTeX Font Info:    Redeclaring math accent \tilde on input line 247.
LaTeX Font Info:    Redeclaring math accent \ddot on input line 248.
LaTeX Font Info:    Redeclaring math accent \check on input line 249.
LaTeX Font Info:    Redeclaring math accent \breve on input line 250.
LaTeX Font Info:    Redeclaring math accent \bar on input line 251.
LaTeX Font Info:    Redeclaring math accent \dot on input line 252.
LaTeX Font Info:    Redeclaring math accent \hat on input line 254.
) (c:/texlive/2024/texmf-dist/tex/latex/merriweather/merriweather.sty
Package: merriweather 2022/09/20 (Bob Tennent) Supports
Merriweather(Sans) font
s for all LaTeX engines.
(c:/texlive/2024/texmf-dist/tex/generic/iftex/ifxetex.sty
Package: ifxetex 2019/10/25 v0.7 ifxetex legacy package. Use iftex
instead.
) (c:/texlive/2024/texmf-dist/tex/generic/iftex/ifluatex.sty
Package: ifluatex 2019/10/25 v1.5 ifluatex legacy package. Use iftex
instead.
) (c:/texlive/2024/texmf-dist/tex/latex/base/textcomp.sty
Package: textcomp 2024/04/24 v2.1b Standard LaTeX package
) (c:/texlive/2024/texmf-dist/tex/latex/xkeyval/xkeyval.sty
Package: xkeyval 2022/06/16 v2.9 package option processing (HA)
(c:/texlive/2024/texmf-dist/tex/generic/xkeyval/xkeyval.tex
(c:/texlive/2024/te
xmf-dist/tex/generic/xkeyval/xkvutils.tex
\XKV@toks=\toks22
\XKV@tempa@toks=\toks23
)
\XKV@depth=\count271
```

```
File: xkeyval.tex 2014/12/03 v2.7a key=value parser (HA)
)) (c:/texlive/2024/texmf-dist/tex/latex/base/fontenc.sty
Package: fontenc 2021/04/29 v2.0v Standard LaTeX package
) (c:/texlive/2024/texmf-dist/tex/latex/fontaxes/fontaxes.sty
Package: fontaxes 2020/07/21 v1.0e Font selection axes
LaTeX Info: Redefining \upshape on input line 29.
LaTeX Info: Redefining \itshape on input line 31.
LaTeX Info: Redefining \slshape on input line 33.
LaTeX Info: Redefining \swshape on input line 35.
LaTeX Info: Redefining \scshape on input line 37.
LaTeX Info: Redefining \sscshape on input line 39.
LaTeX Info: Redefining \ulcshape on input line 41.
LaTeX Info: Redefining \textsw on input line 47.
LaTeX Info: Redefining \textssc on input line 48.
LaTeX Info: Redefining \textulc on input line 49.
)) (c:/texlive/2024/texmf-dist/tex/latex/mathastext/mathastext.sty
Package: mathastext 2024/07/27 v1.4b Use the text font in math mode (JFB)

Package mathastext Info: Starting the math mode configuration.
\mst@exists@muskip=\muskip17
\mst@forall@muskip=\muskip18
\mst@prime@muskip=\muskip19
\mst@do@nonletters=\toks24
\mst@undo@nonletters=\toks25
\mst@do@easynonletters=\toks26
\mst@undo@easynonletters=\toks27
\symmtoperatorfont=\mathgroup8
\symmtletterfont=\mathgroup9
(   mathastext:   ) ! and ?
(   mathastext:   ) punctuation: , . : ; and \colon
LaTeX Info: Redefining \relbar on input line 1201.
LaTeX Info: Redefining \rightarrowfill on input line 1202.
LaTeX Info: Redefining \leftarrowfill on input line 1205.
(   mathastext:   ) + and =
LaTeX Info: Redefining \Relbar on input line 1298.
(   mathastext:   ) adding = ; and + to \nfss@catcodes
(   mathastext:   ) parentheses ( ) [ ] and slash /
(   mathastext:   ) alldelims: < > \backslash \setminus | \vert \mid \{
\}
LaTeX Font Info:    Redeclaring math symbol \setminus on input line 1364.
LaTeX Info: Redefining \models on input line 1383.
(   mathastext:   ) \# \mathdollar \% \&
(   mathastext:   ) \imath and \jmath
LaTeX Font Info:    Overwriting math alphabet `\Mathnormalbold' in
version `nor
mal'
(Font)                  T1/Merriwthr-OsF/b/it --> T1/Merriwthr-OsF/b/it
on inpu
t line 2863.
LaTeX Font Info:    Overwriting math alphabet `\Mathnormalbold' in
version `bol
d'
(Font)                  T1/Merriwthr-OsF/b/it --> T1/Merriwthr-OsF/b/it
on inpu
```

```
t line 2863.
LaTeX Font Info:    Overwriting symbol font `mtletterfont' in version
`normal'
(Font)                  T1/Merriwthr-OsF/m/it --> T1/Merriwthr-OsF/m/it
on inpu
t line 2863.
LaTeX Font Info:    Overwriting symbol font `mtletterfont' in version
`bold'
(Font)                  T1/Merriwthr-OsF/m/it --> T1/Merriwthr-OsF/b/it
on inpu
t line 2863.
LaTeX Font Info:    Overwriting symbol font `mtoperatorfont' in version
`normal
'
(Font)                  T1/Merriwthr-OsF/m/n --> T1/Merriwthr-OsF/m/n on
input
line 2863.
LaTeX Font Info:    Overwriting symbol font `mtoperatorfont' in version
`bold'
(Font)                  T1/Merriwthr-OsF/m/n --> T1/Merriwthr-OsF/b/n on
input
line 2863.
LaTeX Font Info:    Overwriting math alphabet `\Mathbf' in version
`normal'
(Font)                  T1/Merriwthr-OsF/b/n --> T1/Merriwthr-OsF/b/n on
input
line 2863.
LaTeX Font Info:    Overwriting math alphabet `\Mathbf' in version `bold'
(Font)                  T1/Merriwthr-OsF/b/n --> T1/Merriwthr-OsF/b/n on
input
line 2863.
LaTeX Font Info:    Overwriting math alphabet `\Mathit' in version
`normal'
(Font)                  T1/Merriwthr-OsF/m/it --> T1/Merriwthr-OsF/m/it
on inpu
t line 2863.
LaTeX Font Info:    Overwriting math alphabet `\Mathit' in version `bold'
(Font)                  T1/Merriwthr-OsF/m/it --> T1/Merriwthr-OsF/b/it
on inpu
t line 2863.
LaTeX Font Info:    Overwriting math alphabet `\Mathsf' in version
`normal'
(Font)                  T1/MerriwthrSans-OsF/m/n --> T1/MerriwthrSans-
OsF/m/n o
n input line 2863.
LaTeX Font Info:    Overwriting math alphabet `\Mathsf' in version `bold'
(Font)                  T1/MerriwthrSans-OsF/m/n --> T1/MerriwthrSans-
OsF/b/n o
n input line 2863.
LaTeX Font Info:    Overwriting math alphabet `\Mathtt' in version
`normal'
(Font)                  T1/lmtt/m/n --> T1/lmtt/m/n on input line 2863.
LaTeX Font Info:    Overwriting math alphabet `\Mathtt' in version `bold'
(Font)                  T1/lmtt/m/n --> T1/lmtt/b/n on input line 2863.
```

```
(   mathastext:   ) Latin letters in the `normal', resp. `bold',
(   mathastext:   ) math versions are now set up to use the fonts
(   mathastext:   ) T1/Merriwthr-OsF/m/it, resp. T1/Merriwthr-OsF/b/it.
(   mathastext:   ) Other characters (digits, ...) and \log-like names
will be
(   mathastext:   ) typeset with the n shape.
(   mathastext:   ) \hbar
(   mathastext:   ) minus as endash
(   mathastext:   ) The italic option is in effect.
(   mathastext:   ) \HUGE has been (re)-defined.
(   mathastext:   ) mathastext has declared larger sizes for subscripts.
(   mathastext:   ) To keep LaTeX defaults, use option
`defaultmathsizes'.

Package mathastext Info: Loading is complete.  You can now use
\Mathastext to
(mathastext)             modify the normal and bold math versions.  Use
it
(mathastext)             with optional argument or use \MTDeclareVersion
to
(mathastext)             declare additional math versions.
) (c:/texlive/2024/texmf-dist/tex/latex/relsize/relsize.sty
Package: relsize 2013/03/29 ver 4.1
) (c:/texlive/2024/texmf-dist/tex/latex/ragged2e/ragged2e.sty
Package: ragged2e 2023/06/22 v3.6 ragged2e Package
\CenteringLeftskip=\skip52
\RaggedLeftLeftskip=\skip53
\RaggedRightLeftskip=\skip54
\CenteringRightskip=\skip55
\RaggedLeftRightskip=\skip56
\RaggedRightRightskip=\skip57
\CenteringParfillskip=\skip58
\RaggedLeftParfillskip=\skip59
\RaggedRightParfillskip=\skip60
\JustifyingParfillskip=\skip61
\CenteringParindent=\skip62
\RaggedLeftParindent=\skip63
\RaggedRightParindent=\skip64
\JustifyingParindent=\skip65
) (c:/texlive/2024/texmf-dist/tex/latex/xcolor/xcolor.sty
Package: xcolor 2023/11/15 v3.01 LaTeX color extensions (UK)
(c:/texlive/2024/texmf-dist/tex/latex/graphics-cfg/color.cfg
File: color.cfg 2016/01/02 v1.6 sample color configuration
)
Package xcolor Info: Driver file: pdftex.def on input line 274.
(c:/texlive/2024/texmf-dist/tex/latex/graphics-def/pdftex.def
File: pdftex.def 2024/04/13 v1.2c Graphics/color driver for pdftex
) (c:/texlive/2024/texmf-dist/tex/latex/graphics/mathcolor.ltx)
Package xcolor Info: Model `cmy' substituted by `cmy0' on input line
1350.
Package xcolor Info: Model `hsb' substituted by `rgb' on input line 1354.
Package xcolor Info: Model `RGB' extended on input line 1366.
Package xcolor Info: Model `HTML' substituted by `rgb' on input line
1368.
```

```
Package xcolor Info: Model `Hsb' substituted by `hsb' on input line 1369.
Package xcolor Info: Model `tHsb' substituted by `hsb' on input line
1370.
Package xcolor Info: Model `HSB' substituted by `hsb' on input line 1371.
Package xcolor Info: Model `Gray' substituted by `gray' on input line
1372.
Package xcolor Info: Model `wave' substituted by `hsb' on input line
1373.
) (c:/texlive/2024/texmf-dist/tex/latex/colortbl/colortbl.sty
Package: colortbl 2024/07/06 v1.0i Color table columns (DPC)
(c:/texlive/2024/texmf-dist/tex/latex/tools/array.sty
Package: array 2024/06/14 v2.6d Tabular extension package (FMi)
\col@sep=\dimen143
\ar@mcellbox=\box53
\extrarowheight=\dimen144
\NC@list=\toks28
\extratabsurround=\skip66
\backup@length=\skip67
\ar@cellbox=\box54
)
\everycr=\toks29
\minrowclearance=\skip68
\rownum=\count272
) (c:/texlive/2024/texmf-dist/tex/latex/graphics/graphicx.sty
Package: graphicx 2021/09/16 v1.2d Enhanced LaTeX Graphics (DPC,SPQR)
(c:/texlive/2024/texmf-dist/tex/latex/graphics/graphics.sty
Package: graphics 2024/05/23 v1.4g Standard LaTeX Graphics (DPC,SPQR)
(c:/texlive/2024/texmf-dist/tex/latex/graphics/trig.sty
Package: trig 2023/12/02 v1.11 sin cos tan (DPC)
) (c:/texlive/2024/texmf-dist/tex/latex/graphics-cfg/graphics.cfg
File: graphics.cfg 2016/06/04 v1.11 sample graphics configuration
)
Package graphics Info: Driver file: pdftex.def on input line 106.
)
\Gin@req@height=\dimen145
\Gin@req@width=\dimen146
) (c:/texlive/2024/texmf-dist/tex/latex/xpatch/xpatch.sty
(c:/texlive/2024/texm
f-dist/tex/latex/l3kernel/expl3.sty
Package: expl3 2024-05-27 L3 programming layer (loader)
(c:/texlive/2024/texmf-dist/tex/latex/l3backend/l3backend-pdftex.def
File: l3backend-pdftex.def 2024-05-08 L3 backend support: PDF output
(pdfTeX)
\l__color_backend_stack_int=\count273
\l__pdf_internal_box=\box55
))
Package: xpatch 2020/03/25 v0.3a Extending etoolbox patching commands
(c:/texlive/2024/texmf-dist/tex/latex/l3packages/xparse/xparse.sty
Package: xparse 2024-05-08 L3 Experimental document command parser
)) (c:/texlive/2024/texmf-dist/tex/latex/environ/environ.sty
Package: environ 2014/05/04 v0.3 A new way to define environments
(c:/texlive/2024/texmf-dist/tex/latex/trimspaces/trimspaces.sty
Package: trimspaces 2009/09/17 v1.1 Trim spaces around a token list
)
```

```
\@envbody=\toks30
) (c:/texlive/2024/texmf-dist/tex/latex/lastpage/lastpage.sty
Package: lastpage 2024/07/07 v2.1c lastpage: 2.09 or 2e? (HMM)
(c:/texlive/2024/texmf-dist/tex/latex/lastpage/lastpage2e.sty
Package: lastpage2e 2024/07/07 v2.1c Decide which 2e lastpage version to
use (H
MM)
(c:/texlive/2024/texmf-dist/tex/latex/lastpage/lastpagemodern.sty
Package: lastpagemodern 2024-07-07 v2.1c Refers to last page's name (HMM;
JPG)
\c@lastpagecount=\count274
)
)) (c:/texlive/2024/texmf-dist/tex/latex/graphics/rotating.sty
Package: rotating 2016/08/11 v2.16d rotated objects in LaTeX
(c:/texlive/2024/texmf-dist/tex/latex/base/ifthen.sty
Package: ifthen 2024/03/16 v1.1e Standard LaTeX ifthen package (DPC)
)
\c@r@tfl@t=\count275
\rotFPtop=\skip69
\rotFPbot=\skip70
\rot@float@box=\box56
\rot@mess@toks=\toks31
) (c:/texlive/2024/texmf-dist/tex/latex/graphics/lscape.sty
Package: lscape 2020/05/28 v3.02 Landscape Pages (DPC)
) (c:/texlive/2024/texmf-dist/tex/latex/tools/afterpage.sty
Package: afterpage 2023/07/04 v1.08 After-Page Package (DPC)
\AP@output=\toks32
\AP@partial=\box57
\AP@footins=\box58
) (c:/texlive/2024/texmf-dist/tex/latex/textpos/textpos.sty
Package: textpos 2022/07/23 v1.10.1
Package textpos Info: choosing support for LaTeX3 on input line 60.
\TP@textbox=\box59
\TP@holdbox=\box60
\TPHorizModule=\dimen147
\TPVertModule=\dimen148
\TP@margin=\dimen149
\TP@absmargin=\dimen150
Grid set 16 x 16 = 37.34424pt x 52.81541pt
\TPboxrulesize=\dimen151
\TP@ox=\dimen152
\TP@oy=\dimen153
\TP@tbargs=\toks33
TextBlockOrigin set to 0pt x 0pt
) (c:/texlive/2024/texmf-dist/tex/latex/url/url.sty
\Urlmuskip=\muskip20
Package: url 2013/09/16  ver 3.4  Verb mode for urls, etc.
) (c:/texlive/2024/texmf-dist/tex/latex/newfloat/newfloat.sty
Package: newfloat 2023/10/01 v1.2 Defining new floating environments (AR)
Package newfloat Info: `rotating' package detected.
) (c:/texlive/2024/texmf-dist/tex/latex/mdframed/mdframed.sty
Package: mdframed 2013/07/01 1.9b: mdframed
(c:/texlive/2024/texmf-dist/tex/latex/kvoptions/kvoptions.sty
```

```
Package: kvoptions 2022-06-15 v3.15 Key value format for package options
(HO)
(c:/texlive/2024/texmf-dist/tex/generic/ltxcmds/ltxcmds.sty
Package: ltxcmds 2023-12-04 v1.26 LaTeX kernel commands for general use
(HO)
) (c:/texlive/2024/texmf-dist/tex/latex/kvsetkeys/kvsetkeys.sty
Package: kvsetkeys 2022-10-05 v1.19 Key value parser (HO)
)) (c:/texlive/2024/texmf-dist/tex/latex/zref/zref-abspage.sty
Package: zref-abspage 2023-09-14 v2.35 Module abspage for zref (HO)
(c:/texlive/2024/texmf-dist/tex/latex/zref/zref-base.sty
Package: zref-base 2023-09-14 v2.35 Module base for zref (HO)
(c:/texlive/2024/texmf-dist/tex/generic/infwarerr/infwarerr.sty
Package: infwarerr 2019/12/03 v1.5 Providing info/warning/error messages
(HO)
) (c:/texlive/2024/texmf-dist/tex/generic/kvdefinekeys/kvdefinekeys.sty
Package: kvdefinekeys 2019-12-19 v1.6 Define keys (HO)
) (c:/texlive/2024/texmf-dist/tex/generic/pdftexcmds/pdftexcmds.sty
Package: pdftexcmds 2020-06-27 v0.33 Utility functions of pdfTeX for
LuaTeX (HO
)
Package pdftexcmds Info: \pdf@primitive is available.
Package pdftexcmds Info: \pdf@ifprimitive is available.
Package pdftexcmds Info: \pdfdraftmode found.
) (c:/texlive/2024/texmf-dist/tex/generic/etexcmds/etexcmds.sty
Package: etexcmds 2019/12/15 v1.7 Avoid name clashes with e-TeX commands
(HO)
) (c:/texlive/2024/texmf-dist/tex/latex/auxhook/auxhook.sty
Package: auxhook 2019-12-17 v1.6 Hooks for auxiliary files (HO)
)
Package zref Info: New property list: main on input line 767.
Package zref Info: New property: default on input line 768.
Package zref Info: New property: page on input line 769.
)
\c@abspage=\count276
Package zref Info: New property: abspage on input line 67.
) (c:/texlive/2024/texmf-dist/tex/latex/needspace/needspace.sty
Package: needspace 2010/09/12 v1.3d reserve vertical space
)
\mdf@templength=\skip71
\c@mdf@globalstyle@cnt=\count277
\mdf@skipabove@length=\skip72
\mdf@skipbelow@length=\skip73
\mdf@leftmargin@length=\skip74
\mdf@rightmargin@length=\skip75
\mdf@innerleftmargin@length=\skip76
\mdf@innerrightmargin@length=\skip77
\mdf@innertopmargin@length=\skip78
\mdf@innerbottommargin@length=\skip79
\mdf@splittopskip@length=\skip80
\mdf@splitbottomskip@length=\skip81
\mdf@outermargin@length=\skip82
\mdf@innermargin@length=\skip83
\mdf@linewidth@length=\skip84
\mdf@innerlinewidth@length=\skip85
```

```
\mdf@middlelinewidth@length=\skip86
\mdf@outerlinewidth@length=\skip87
\mdf@roundcorner@length=\skip88
\mdf@footenotedistance@length=\skip89
\mdf@userdefinedwidth@length=\skip90
\mdf@needspace@length=\skip91
\mdf@frametitleaboveskip@length=\skip92
\mdf@frametitlebelowskip@length=\skip93
\mdf@frametitlerulewidth@length=\skip94
\mdf@frametitleleftmargin@length=\skip95
\mdf@frametitlerightmargin@length=\skip96
\mdf@shadowsize@length=\skip97
\mdf@extratopheight@length=\skip98
\mdf@subtitleabovelinewidth@length=\skip99
\mdf@subtitlebelowlinewidth@length=\skip100
\mdf@subtitleaboveskip@length=\skip101
\mdf@subtitlebelowskip@length=\skip102
\mdf@subtitleinneraboveskip@length=\skip103
\mdf@subtitleinnerbelowskip@length=\skip104
\mdf@subsubtitleabovelinewidth@length=\skip105
\mdf@subsubtitlebelowlinewidth@length=\skip106
\mdf@subsubtitleaboveskip@length=\skip107
\mdf@subsubtitlebelowskip@length=\skip108
\mdf@subsubtitleinneraboveskip@length=\skip109
\mdf@subsubtitleinnerbelowskip@length=\skip110
(c:/texlive/2024/texmf-dist/tex/latex/mdframed/md-frame-0.mdf
File: md-frame-0.mdf 2013/07/01\ 1.9b: md-frame-0
)
\mdf@frametitlebox=\box61
\mdf@footnotebox=\box62
\mdf@splitbox@one=\box63
\mdf@splitbox@two=\box64
\mdf@splitbox@save=\box65
\mdfsplitboxwidth=\skip111
\mdfsplitboxtotalwidth=\skip112
\mdfsplitboxheight=\skip113
\mdfsplitboxdepth=\skip114
\mdfsplitboxtotalheight=\skip115
\mdfframetitleboxwidth=\skip116
\mdfframetitleboxtotalwidth=\skip117
\mdfframetitleboxheight=\skip118
\mdfframetitleboxdepth=\skip119
\mdfframetitleboxtotalheight=\skip120
\mdffootnoteboxwidth=\skip121
\mdffootnoteboxtotalwidth=\skip122
\mdffootnoteboxheight=\skip123
\mdffootnoteboxdepth=\skip124
\mdffootnoteboxtotalheight=\skip125
\mdftotallinewidth=\skip126
\mdfboundingboxwidth=\skip127
\mdfboundingboxtotalwidth=\skip128
\mdfboundingboxheight=\skip129
\mdfboundingboxdepth=\skip130
\mdfboundingboxtotalheight=\skip131
```

```
\mdf@freevspace@length=\skip132
\mdf@horizontalwidthofbox@length=\skip133
\mdf@verticalmarginwhole@length=\skip134
\mdf@horizontalspaceofbox=\skip135
\mdfsubtitleheight=\skip136
\mdfsubsubtitleheight=\skip137
\c@mdfcountframes=\count278

****** mdframed patching \endmdf@trivlist

****** -- success******

\mdf@envdepth=\count279
\c@mdf@env@i=\count280
\c@mdf@env@ii=\count281
\c@mdf@zref@counter=\count282
Package zref Info: New property: mdf@pagevalue on input line 895.
) (c:/texlive/2024/texmf-dist/tex/latex/titlesec/titlesec.sty
Package: titlesec 2023/10/27 v2.16 Sectioning titles
\ttl@box=\box66
\beforetitleunit=\skip138
\aftertitleunit=\skip139
\ttl@plus=\dimen154
\ttl@minus=\dimen155
\ttl@toksa=\toks34
\titlewidth=\dimen156
\titlewidthlast=\dimen157
\titlewidthfirst=\dimen158
) (c:/texlive/2024/texmf-dist/tex/latex/koma-script/scrextend.sty
Package: scrextend 2023/07/07 v3.41 KOMA-Script package (extend other
classes w
ith features of KOMA-Script classes)
(c:/texlive/2024/texmf-dist/tex/latex/koma-script/scrkbase.sty
Package: scrkbase 2023/07/07 v3.41 KOMA-Script package (KOMA-Script-
dependent b
asics and keyval usage)
(c:/texlive/2024/texmf-dist/tex/latex/koma-script/scrbase.sty
Package: scrbase 2023/07/07 v3.41 KOMA-Script package (KOMA-Script-
independent
basics and keyval usage)
(c:/texlive/2024/texmf-dist/tex/latex/koma-script/scrlfile.sty
Package: scrlfile 2023/07/07 v3.41 KOMA-Script package (file load hooks)
(c:/texlive/2024/texmf-dist/tex/latex/koma-script/scrlfile-hook.sty
Package: scrlfile-hook 2023/07/07 v3.41 KOMA-Script package (using LaTeX
hooks)

(c:/texlive/2024/texmf-dist/tex/latex/koma-script/scrlogo.sty
Package: scrlogo 2023/07/07 v3.41 KOMA-Script package (logo)
)))
Applying: [2021/05/01] Usage of raw or classic option list on input line
252.
Already applied: [0000/00/00] Usage of raw or classic option list on
input line
 368.
```

```
))
Package scrextend Info: unexpected definition of `\@makefnmark'.
(scrextend)             Trying to patch it on input line 1762.
Package scrextend Info: patch seems to be successfull on input line 1762.
)

LaTeX Font Warning: Font shape `T1/cmr/m/n' in size <7.5> not available
(Font)              size <7> substituted on input line 69.

(c:/texlive/2024/texmf-dist/tex/latex/tools/calc.sty
Package: calc 2023/07/08 v4.3 Infix arithmetic (KKT,FJ)
\calc@Acount=\count283
\calc@Bcount=\count284
\calc@Adimen=\dimen159
\calc@Bdimen=\dimen160
\calc@Askip=\skip140
\calc@Bskip=\skip141
LaTeX Info: Redefining \setlength on input line 80.
LaTeX Info: Redefining \addtolength on input line 81.
\calc@Ccount=\count285
\calc@Cskip=\skip142
) (c:/texlive/2024/texmf-dist/tex/latex/geometry/geometry.sty
Package: geometry 2020/01/02 v5.9 Page Geometry
(c:/texlive/2024/texmf-dist/tex/generic/iftex/ifvtex.sty
Package: ifvtex 2019/10/25 v1.7 ifvtex legacy package. Use iftex instead.
)
\Gm@cnth=\count286
\Gm@cntv=\count287
\c@Gm@tempcnt=\count288
\Gm@bindingoffset=\dimen161
\Gm@wd@mp=\dimen162
\Gm@odd@mp=\dimen163
\Gm@even@mp=\dimen164
\Gm@layoutwidth=\dimen165
\Gm@layoutheight=\dimen166
\Gm@layouthoffset=\dimen167
\Gm@layoutvoffset=\dimen168
\Gm@dimlist=\toks35
) (c:/texlive/2024/texmf-dist/tex/latex/preprint/authblk.sty
Package: authblk 2001/02/27 1.3 (PWD)
\affilsep=\skip143
\@affilsep=\skip144
\c@Maxaffil=\count289
\c@authors=\count290
\c@affil=\count291
) (c:/texlive/2024/texmf-dist/tex/latex/footmisc/footmisc.sty
Package: footmisc 2023/07/05 v6.0f a miscellany of footnote facilities
\FN@temptoken=\toks36
\footnotemargin=\dimen169
\@outputbox@depth=\dimen170
Package footmisc Info: Declaring symbol style bringhurst on input line
696.
Package footmisc Info: Declaring symbol style chicago on input line 704.
Package footmisc Info: Declaring symbol style wiley on input line 713.
```

```
Package footmisc Info: Declaring symbol style lamport-robust on input
line 724.

Package footmisc Info: Declaring symbol style lamport* on input line 744.
Package footmisc Info: Declaring symbol style lamport*-robust on input
line 765
.
) (c:/texlive/2024/texmf-dist/tex/latex/fancyhdr/fancyhdr.sty
Package: fancyhdr 2024/07/23 v4.3.1 Extensive control of page headers and
foote
rs
\f@nch@headwidth=\skip145
\f@nch@O@elh=\skip146
\f@nch@O@erh=\skip147
\f@nch@O@olh=\skip148
\f@nch@O@orh=\skip149
\f@nch@O@elf=\skip150
\f@nch@O@erf=\skip151
\f@nch@O@olf=\skip152
\f@nch@O@orf=\skip153
) (c:/texlive/2024/texmf-dist/tex/generic/alphalph/alphalph.sty
Package: alphalph 2019/12/09 v2.6 Convert numbers to letters (HO)
(c:/texlive/2024/texmf-dist/tex/generic/intcalc/intcalc.sty
Package: intcalc 2019/12/15 v1.3 Expandable calculations with integers
(HO)
))
\c@authorfn=\count292
(c:/texlive/2024/texmf-dist/tex/latex/abstract/abstract.sty
Package: abstract 2009/06/08 v1.2a configurable abstracts
\abstitleskip=\skip154
\absleftindent=\skip155
\absrightindent=\skip156
\absparindent=\skip157
\absparsep=\skip158
)
Package newfloat Info: New float `keypoints' with options
`placement=t!,name=kp
t' on input line 291.
\c@keypoints=\count293
\newfloat@ftype=\count294
Package newfloat Info: float type `keypoints'=8 on input line 291.
(c:/texlive/2024/texmf-dist/tex/latex/enumitem/enumitem.sty
Package: enumitem 2019/06/20 v3.9 Customized lists
\labelindent=\skip159
\enit@outerparindent=\dimen171
\enit@toks=\toks37
\enit@inbox=\box67
\enit@count@id=\count295
\enitdp@description=\count296
) (c:/texlive/2024/texmf-dist/tex/latex/quoting/quoting.sty
Package: quoting 2014/01/28 v0.1c Consolidated environment for displayed
text
\quo@toppartop=\skip160
) (c:/texlive/2024/texmf-dist/tex/latex/sttools/stfloats.sty
```

```
Package: stfloats 2017/03/27 v3.3 Improve float mechanism and
baselineskip sett
ings
\@dblbotnum=\count297
\c@dblbotnumber=\count298
) (c:/texlive/2024/texmf-dist/tex/latex/booktabs/booktabs.sty
Package: booktabs 2020/01/12 v1.61803398 Publication quality tables
\heavyrulewidth=\dimen172
\lightrulewidth=\dimen173
\cmidrulewidth=\dimen174
\belowrulesep=\dimen175
\belowbottomsep=\dimen176
\aboverulesep=\dimen177
\abovetopsep=\dimen178
\cmidrulesep=\dimen179
\cmidrulekern=\dimen180
\defaultaddspace=\dimen181
\@cmidla=\count299
\@cmidlb=\count300
\@aboverulesep=\dimen182
\@belowrulesep=\dimen183
\@thisruleclass=\count301
\@lastruleclass=\count302
\@thisrulewidth=\dimen184
) (c:/texlive/2024/texmf-dist/tex/latex/tools/tabularx.sty
Package: tabularx 2023/12/11 v2.12a `tabularx' package (DPC)
\TX@col@width=\dimen185
\TX@old@table=\dimen186
\TX@old@col=\dimen187
\TX@target=\dimen188
\TX@delta=\dimen189
\TX@cols=\count303
\TX@ftn=\toks38
)
\enitdp@tablenotes=\count304
(c:/texlive/2024/texmf-dist/tex/latex/caption/caption.sty
Package: caption 2023/08/05 v3.6o Customizing captions (AR)
(c:/texlive/2024/texmf-dist/tex/latex/caption/caption3.sty
Package: caption3 2023/07/31 v2.4d caption3 kernel (AR)
\caption@tempdima=\dimen190
\captionmargin=\dimen191
\caption@leftmargin=\dimen192
\caption@rightmargin=\dimen193
\caption@width=\dimen194
\caption@indent=\dimen195
\caption@parindent=\dimen196
\caption@hangindent=\dimen197
Package caption Info: Standard document class detected.
)
\c@caption@flags=\count305
\c@continuedfloat=\count306
Package caption Info: rotating package is loaded.
Package caption Info: scrextend package is loaded.
\caption@addmargin@hsize=\dimen198
```

```
\caption@addmargin@linewidth=\dimen199
) (c:/texlive/2024/texmf-dist/tex/latex/natbib/natbib.sty
Package: natbib 2010/09/13 8.31b (PWD, AO)
\bibhang=\skip161
\bibsep=\skip162
LaTeX Info: Redefining \cite on input line 694.
\c@NAT@ctr=\count307
)) (c:/texlive/2024/texmf-dist/tex/latex/siunitx/siunitx.sty
Package: siunitx 2024-06-24 v3.3.19 A comprehensive (SI) units package
\l__siunitx_number_uncert_offset_int=\count308
\l__siunitx_number_exponent_fixed_int=\count309
\l__siunitx_number_min_decimal_int=\count310
\l__siunitx_number_min_integer_int=\count311
\l__siunitx_number_round_precision_int=\count312
\l__siunitx_number_lower_threshold_int=\count313
\l__siunitx_number_upper_threshold_int=\count314
\l__siunitx_number_group_first_int=\count315
\l__siunitx_number_group_size_int=\count316
\l__siunitx_number_group_minimum_int=\count317
\l__siunitx_angle_tmp_dim=\dimen256
\l__siunitx_angle_marker_box=\box68
\l__siunitx_angle_unit_box=\box69
\l__siunitx_compound_count_int=\count318
(c:/texlive/2024/texmf-dist/tex/latex/translations/translations.sty
Package: translations 2022/02/05 v1.12 internationalization of LaTeX2e
packages
 (CN)
) (c:/texlive/2024/texmf-dist/tex/latex/amsmath/amstext.sty
Package: amstext 2021/08/26 v2.01 AMS text
(c:/texlive/2024/texmf-dist/tex/latex/amsmath/amsgen.sty
File: amsgen.sty 1999/11/30 v2.0 generic functions
\@emptytoks=\toks39
\ex@=\dimen257
))
\l__siunitx_table_tmp_box=\box70
\l__siunitx_table_tmp_dim=\dimen258
\l__siunitx_table_column_width_dim=\dimen259
\l__siunitx_table_integer_box=\box71
\l__siunitx_table_decimal_box=\box72
\l__siunitx_table_uncert_box=\box73
\l__siunitx_table_before_box=\box74
\l__siunitx_table_after_box=\box75
\l__siunitx_table_before_dim=\dimen260
\l__siunitx_table_carry_dim=\dimen261
\l__siunitx_unit_tmp_int=\count319
\l__siunitx_unit_position_int=\count320
\l__siunitx_unit_total_int=\count321
) (c:/texlive/2024/texmf-dist/tex/latex/multirow/multirow.sty
Package: multirow 2021/03/15 v2.8 Span multiple rows of a table
\multirow@colwidth=\skip163
\multirow@cntb=\count322
\multirow@dima=\skip164
\bigstrutjot=\dimen262
) (c:/texlive/2024/texmf-dist/tex/latex/amsmath/amsmath.sty
```

```
Package: amsmath 2024/05/23 v2.17q AMS math features
\@mathmargin=\skip165
For additional information on amsmath, use the `?' option.
(c:/texlive/2024/texmf-dist/tex/latex/amsmath/amsbsy.sty
Package: amsbsy 1999/11/29 v1.2d Bold Symbols
\pmbraise@=\dimen263
) (c:/texlive/2024/texmf-dist/tex/latex/amsmath/amsopn.sty
Package: amsopn 2022/04/08 v2.04 operator names
)
\inf@bad=\count323
LaTeX Info: Redefining \frac on input line 233.
\uproot@=\count324
\leftroot@=\count325
LaTeX Info: Redefining \overline on input line 398.
LaTeX Info: Redefining \colon on input line 409.
\classnum@=\count326
\DOTSCASE@=\count327
LaTeX Info: Redefining \ldots on input line 495.
LaTeX Info: Redefining \dots on input line 498.
LaTeX Info: Redefining \cdots on input line 619.
\Mathstrutbox@=\box76
\strutbox@=\box77
LaTeX Info: Redefining \big on input line 721.
LaTeX Info: Redefining \Big on input line 722.
LaTeX Info: Redefining \bigg on input line 723.
LaTeX Info: Redefining \Bigg on input line 724.
\big@size=\dimen264
LaTeX Font Info:    Redeclaring font encoding OML on input line 742.
LaTeX Font Info:    Redeclaring font encoding OMS on input line 743.
\macc@depth=\count328
LaTeX Info: Redefining \bmod on input line 904.
LaTeX Info: Redefining \pmod on input line 909.
LaTeX Info: Redefining \smash on input line 939.
LaTeX Info: Redefining \relbar on input line 969.
LaTeX Info: Redefining \Relbar on input line 970.
\c@MaxMatrixCols=\count329
\dotsspace@=\muskip21
\c@parentequation=\count330
\dspbrk@lvl=\count331
\tag@help=\toks40
\row@=\count332
\column@=\count333
\maxfields@=\count334
\andhelp@=\toks41
\eqnshift@=\dimen265
\alignsep@=\dimen266
\tagshift@=\dimen267
\tagwidth@=\dimen268
\totwidth@=\dimen269
\lineht@=\dimen270
\@envbody=\toks42
\multlinegap=\skip166
\multlinetaggap=\skip167
\mathdisplay@stack=\toks43
```

```
LaTeX Info: Redefining \[ on input line 2953.
LaTeX Info: Redefining \] on input line 2954.
) (c:/texlive/2024/texmf-dist/tex/latex/lineno/lineno.sty
Package: lineno 2023/05/20 line numbers on paragraphs v5.3
\linenopenalty=\count335
\output=\toks44
\linenoprevgraf=\count336
\linenumbersep=\dimen271
\linenumberwidth=\dimen272
\c@linenumber=\count337
\c@pagewiselinenumber=\count338
\c@LN@truepage=\count339
\c@internallinenumber=\count340
\c@internallinenumbers=\count341
\quotelinenumbersep=\dimen273
\bframerule=\dimen274
\bframesep=\dimen275
\bframebox=\box78
\linenoamsmath@ams@eqpen=\count342
LaTeX Info: Redefining \\ on input line 3180.
) (c:/texlive/2024/texmf-dist/tex/generic/soul/soul.sty
Package: soul 2023-06-14 v3.1 Permit use of UTF-8 characters in soul (HO)
(c:/texlive/2024/texmf-dist/tex/generic/soul/soul-ori.sty
Package: soul-ori 2023-06-14 v3.1 letterspacing/underlining (mf)
\SOUL@word=\toks45
\SOUL@lasttoken=\toks46
\SOUL@syllable=\toks47
\SOUL@cmds=\toks48
\SOUL@buffer=\toks49
\SOUL@token=\toks50
\SOUL@syllgoal=\dimen276
\SOUL@syllwidth=\dimen277
\SOUL@charkern=\dimen278
\SOUL@hyphkern=\dimen279
\SOUL@dimen=\dimen280
\SOUL@dimeni=\dimen281
\SOUL@minus=\count343
\SOUL@comma=\count344
\SOUL@apo=\count345
\SOUL@grave=\count346
\SOUL@spaceskip=\skip168
\SOUL@ttwidth=\dimen282
\SOUL@uldp=\dimen283
\SOUL@ulht=\dimen284
)) (c:/texlive/2024/texmf-dist/tex/latex/hyperref/hyperref.sty
Package: hyperref 2024-07-10 v7.01j Hypertext links for LaTeX
(c:/texlive/2024/texmf-dist/tex/generic/pdfescape/pdfescape.sty
Package: pdfescape 2019/12/09 v1.15 Implements pdfTeX's escape features
(HO)
) (c:/texlive/2024/texmf-dist/tex/latex/hycolor/hycolor.sty
Package: hycolor 2020-01-27 v1.10 Color options for hyperref/bookmark
(HO)
) (c:/texlive/2024/texmf-dist/tex/latex/hyperref/nameref.sty
Package: nameref 2023-11-26 v2.56 Cross-referencing by name of section
```

```
(c:/texlive/2024/texmf-dist/tex/latex/refcount/refcount.sty
Package: refcount 2019/12/15 v3.6 Data extraction from label references
(HO)
) (c:/texlive/2024/texmf-
dist/tex/generic/gettitlestring/gettitlestring.sty
Package: gettitlestring 2019/12/15 v1.6 Cleanup title references (HO)
)
\c@section@level=\count347
) (c:/texlive/2024/texmf-dist/tex/generic/stringenc/stringenc.sty
Package: stringenc 2019/11/29 v1.12 Convert strings between diff.
encodings (HO
)
)
\@linkdim=\dimen285
\Hy@linkcounter=\count348
\Hy@pagecounter=\count349
(c:/texlive/2024/texmf-dist/tex/latex/hyperref/pd1enc.def
File: pd1enc.def 2024-07-10 v7.01j Hyperref: PDFDocEncoding definition
(HO)
Now handling font encoding PD1 ...
... no UTF-8 mapping file for font encoding PD1
)
\Hy@SavedSpaceFactor=\count350
(c:/texlive/2024/texmf-dist/tex/latex/hyperref/puenc.def
File: puenc.def 2024-07-10 v7.01j Hyperref: PDF Unicode definition (HO)
Now handling font encoding PU ...
... no UTF-8 mapping file for font encoding PU
)
Package hyperref Info: Option `colorlinks' set `true' on input line 4040.
Package hyperref Info: Hyper figures OFF on input line 4157.
Package hyperref Info: Link nesting OFF on input line 4162.
Package hyperref Info: Hyper index ON on input line 4165.
Package hyperref Info: Plain pages OFF on input line 4172.
Package hyperref Info: Backreferencing OFF on input line 4177.
Package hyperref Info: Implicit mode ON; LaTeX internals redefined.
Package hyperref Info: Bookmarks ON on input line 4424.
\c@Hy@tempcnt=\count351
LaTeX Info: Redefining \url on input line 4763.
\XeTeXLinkMargin=\dimen286
(c:/texlive/2024/texmf-dist/tex/generic/bitset/bitset.sty
Package: bitset 2019/12/09 v1.3 Handle bit-vector datatype (HO)
(c:/texlive/2024/texmf-dist/tex/generic/bigintcalc/bigintcalc.sty
Package: bigintcalc 2019/12/15 v1.5 Expandable calculations on big
integers (HO
)
))
\Fld@menulength=\count352
\Field@Width=\dimen287
\Fld@charsize=\dimen288
Package hyperref Info: Hyper figures OFF on input line 6042.
Package hyperref Info: Link nesting OFF on input line 6047.
Package hyperref Info: Hyper index ON on input line 6050.
Package hyperref Info: backreferencing OFF on input line 6057.
Package hyperref Info: Link coloring ON on input line 6060.
```

```
Package hyperref Info: Link coloring with OCG OFF on input line 6067.
Package hyperref Info: PDF/A mode OFF on input line 6072.
(c:/texlive/2024/texmf-dist/tex/latex/base/atbegshi-ltx.sty
Package: atbegshi-ltx 2021/01/10 v1.0c Emulation of the original atbegshi
package with kernel methods
)
\Hy@abspage=\count353
\c@Item=\count354
\c@Hfootnote=\count355
)
Package hyperref Info: Driver (autodetected): hpdftex.
(c:/texlive/2024/texmf-dist/tex/latex/hyperref/hpdftex.def
File: hpdftex.def 2024-07-10 v7.01j Hyperref driver for pdfTeX
(c:/texlive/2024/texmf-dist/tex/latex/base/atveryend-ltx.sty
Package: atveryend-ltx 2020/08/19 v1.0a Emulation of the original
atveryend pac
kage
with kernel methods
)
\HyAnn@Count=\count356
\Fld@listcount=\count357
\c@bookmark@seq@number=\count358
(c:/texlive/2024/texmf-dist/tex/latex/rerunfilecheck/rerunfilecheck.sty
Package: rerunfilecheck 2022-07-10 v1.10 Rerun checks for auxiliary files
(HO)
(c:/texlive/2024/texmf-dist/tex/generic/uniquecounter/uniquecounter.sty
Package: uniquecounter 2019/12/15 v1.4 Provide unlimited unique counter
(HO)
)
Package uniquecounter Info: New unique counter `rerunfilecheck' on input
line 2
85.
)
\Hy@SectionHShift=\skip169
)
Package translations Info: No language package found. I am going to use
`englis
h' as default language. on input line 53.
LaTeX Font Info:    Trying to load font information for T1+Merriwthr-OsF
on inp
ut line 53.
(c:/texlive/2024/texmf-dist/tex/latex/merriweather/T1Merriwthr-OsF.fd
File: T1Merriwthr-OsF.fd 2020/08/30 (autoinst) Font definitions for
T1/Merriwth
r-OsF.
)
LaTeX Font Info:    Font shape `T1/Merriwthr-OsF/m/n' will be
(Font)              scaled to size 7.5pt on input line 53.
(./MADRe_main.aux

LaTeX Warning: Label `fig:F1_simulated_num_idenf' multiply defined.

)
\openout1 = `MADRe_main.aux'.
```

```
LaTeX Font Info:    Checking defaults for OML/cmm/m/it on input line 53.
LaTeX Font Info:    ... okay on input line 53.
LaTeX Font Info:    Checking defaults for OMS/cmsy/m/n on input line 53.
LaTeX Font Info:    ... okay on input line 53.
LaTeX Font Info:    Checking defaults for OT1/cmr/m/n on input line 53.
LaTeX Font Info:    ... okay on input line 53.
LaTeX Font Info:    Checking defaults for T1/cmr/m/n on input line 53.
LaTeX Font Info:    ... okay on input line 53.
LaTeX Font Info:    Checking defaults for TS1/cmr/m/n on input line 53.
LaTeX Font Info:    ... okay on input line 53.
LaTeX Font Info:    Checking defaults for OMX/cmex/m/n on input line 53.
LaTeX Font Info:    ... okay on input line 53.
LaTeX Font Info:    Checking defaults for U/cmr/m/n on input line 53.
LaTeX Font Info:    ... okay on input line 53.
LaTeX Font Info:    Checking defaults for PD1/pdf/m/n on input line 53.
LaTeX Font Info:    ... okay on input line 53.
LaTeX Font Info:    Checking defaults for PU/pdf/m/n on input line 53.
LaTeX Font Info:    ... okay on input line 53.
LaTeX Info: Redefining \microtypecontext on input line 53.
Package microtype Info: Applying patch `item' on input line 53.
Package microtype Info: Applying patch `toc' on input line 53.
Package microtype Info: Applying patch `eqnum' on input line 53.
Package microtype Info: Applying patch `footnote' on input line 53.
Package microtype Info: Applying patch `verbatim' on input line 53.
Package microtype Info: Generating PDF output.
Package microtype Info: Character protrusion enabled (level 2).
Package microtype Info: Using default protrusion set `alltext'.
Package microtype Info: Automatic font expansion enabled (level 2),
(microtype)             stretch: 20, shrink: 20, step: 1, non-selected.
Package microtype Info: Using default expansion set `alltext-nott'.
LaTeX Info: Redefining \showhyphens on input line 53.
Package microtype Info: No adjustment of tracking.
Package microtype Info: No adjustment of interword spacing.
Package microtype Info: No adjustment of character kerning.
Package microtype Info: Loading generic protrusion settings for font
family
(microtype)             `Merriwthr-OsF' (encoding: T1).
(microtype)             For optimal results, create family-specific
settings.
(microtype)             See the microtype manual for details.
LaTeX Font Info:    Redeclaring symbol font `operators' on input line 53.
LaTeX Font Info:    Encoding `OT1' has changed to `T1' for symbol font
(Font)              `operators' in the math version `normal' on input
line 53.
LaTeX Font Info:    Overwriting symbol font `operators' in version
`normal'
(Font)                  OT1/cmr/m/n --> T1/Merriwthr-OsF/m/up on input
line 53.

LaTeX Font Info:    Encoding `OT1' has changed to `T1' for symbol font
(Font)              `operators' in the math version `bold' on input line
53.
LaTeX Font Info:    Overwriting symbol font `operators' in version `bold'
```

```
(Font)                  OT1/cmr/bx/n --> T1/Merriwthr-OsF/m/up on input
line 53
.
LaTeX Font Info:    Overwriting symbol font `operators' in version `bold'
(Font)                  T1/Merriwthr-OsF/m/up --> T1/Merriwthr-OsF/b/up
on inpu
t line 53.
LaTeX Font Info:    Redeclaring math alphabet \mathbf on input line 53.
LaTeX Font Info:    Overwriting math alphabet `\mathbf' in version
`normal'
(Font)                  OT1/cmr/bx/n --> T1/Merriwthr-OsF/b/up on input
line 53
.
LaTeX Font Info:    Overwriting math alphabet `\mathbf' in version `bold'
(Font)                  OT1/cmr/bx/n --> T1/Merriwthr-OsF/b/up on input
line 53
.
LaTeX Font Info:    Redeclaring math alphabet \mathsf on input line 53.
LaTeX Font Info:    Overwriting math alphabet `\mathsf' in version
`normal'
(Font)                  OT1/cmss/m/n --> T1/MerriwthrSans-OsF/m/up on
input lin
e 53.
LaTeX Font Info:    Overwriting math alphabet `\mathsf' in version `bold'
(Font)                  OT1/cmss/bx/n --> T1/MerriwthrSans-OsF/m/up on
input li
ne 53.
LaTeX Font Info:    Redeclaring math alphabet \mathit on input line 53.
LaTeX Font Info:    Overwriting math alphabet `\mathit' in version
`normal'
(Font)                  OT1/cmr/m/it --> T1/Merriwthr-OsF/m/it on input
line 53
.
LaTeX Font Info:    Overwriting math alphabet `\mathit' in version `bold'
(Font)                  OT1/cmr/bx/it --> T1/Merriwthr-OsF/m/it on input
line 5
3.
LaTeX Font Info:    Redeclaring math alphabet \mathtt on input line 53.
LaTeX Font Info:    Overwriting math alphabet `\mathtt' in version
`normal'
(Font)                  OT1/cmtt/m/n --> T1/lmtt/m/up on input line 53.
LaTeX Font Info:    Overwriting math alphabet `\mathtt' in version `bold'
(Font)                  OT1/cmtt/m/n --> T1/lmtt/m/up on input line 53.
LaTeX Font Info:    Overwriting math alphabet `\mathsf' in version `bold'
(Font)                  T1/MerriwthrSans-OsF/m/up --> T1/MerriwthrSans-
OsF/b/up
 on input line 53.
LaTeX Font Info:    Overwriting math alphabet `\mathit' in version `bold'
(Font)                  T1/Merriwthr-OsF/m/it --> T1/Merriwthr-OsF/b/it
on inpu
t line 53.
\c@mv@tabular=\count359
\c@mv@boldtabular=\count360
(c:/texlive/2024/texmf-dist/tex/context/base/mkii/supp-pdf.mkii
```

```
[Loading MPS to PDF converter (version 2006.09.02).]
\scratchcounter=\count361
\scratchdimen=\dimen289
\scratchbox=\box79
\nofMPsegments=\count362
\nofMParguments=\count363
\everyMPshowfont=\toks51
\MPscratchCnt=\count364
\MPscratchDim=\dimen290
\MPnumerator=\count365
\makeMPintoPDFobject=\count366
\everyMPtoPDFconversion=\toks52
) (c:/texlive/2024/texmf-dist/tex/latex/epstopdf-pkg/epstopdf-base.sty
Package: epstopdf-base 2020-01-24 v2.11 Base part for package epstopdf
Package epstopdf-base Info: Redefining graphics rule for `.eps' on input
line 4
85.
(c:/texlive/2024/texmf-dist/tex/latex/latexconfig/epstopdf-sys.cfg
File: epstopdf-sys.cfg 2010/07/13 v1.3 Configuration of (r)epstopdf for
TeX Liv
e
))
*geometry* driver: auto-detecting
*geometry* detected driver: pdftex
*geometry* verbose mode - [ preamble ] result:
* driver: pdftex
* paper: a4paper
* layout: <same size as paper>
* layoutoffset:(h,v)=(0.0pt,0.0pt)
* modes: includefoot twoside
* h-part:(L,W,R)=(54.64pt, 488.22787pt, 54.64pt)
* v-part:(T,H,B)=(66.0pt, 745.04684pt, 34.0pt)
* \paperwidth=597.50787pt
* \paperheight=845.04684pt
* \textwidth=488.22787pt
* \textheight=715.04684pt
* \oddsidemargin=-17.62999pt
* \evensidemargin=-17.62999pt
* \topmargin=-47.76999pt
* \headheight=17.5pt
* \headsep=24.0pt
* \topskip=10.0pt
* \footskip=30.0pt
* \marginparwidth=48.0pt
* \marginparsep=10.0pt
* \columnsep=18.0pt
* \skip\footins=22.0pt plus 2.0pt
* \hoffset=0.0pt
* \voffset=0.0pt
* \mag=1000
* \@twocolumntrue
* \@twosidetrue
* \@mparswitchtrue
* \@reversemarginfalse
```

```
* (1in=72.27pt=25.4mm, 1cm=28.453pt)

Package caption Info: Begin \AtBeginDocument code.
Package caption Info: hyperref package is loaded.
Package caption Info: End \AtBeginDocument code.

(c:/texlive/2024/texmf-dist/tex/latex/translations/translations-basic-
dictionar
y-english.trsl
File: translations-basic-dictionary-english.trsl (english translation
file `tra
nslations-basic-dictionary')
)
Package translations Info: loading dictionary `translations-basic-
dictionary' f
or `english'. on input line 53.
Package hyperref Info: Link coloring ON on input line 53.
(./MADRe_main.out) (./MADRe_main.out)
\@outlinefile=\write3
\openout3 = `MADRe_main.out'.

\@gscitedetails=\box80
\@gscitedetailsheight=\skip170
\@gsheadbox=\box81
\@gsheadboxheight=\skip171
LaTeX Font Info:    Font shape `T1/Merriwthr-OsF/b/n' will be
(Font)              scaled to size 6.5pt on input line 53.
LaTeX Font Info:    Calculating math sizes for size <7.5> on input line
53.

LaTeX Font Warning: Font shape `T1/Merriwthr-OsF/m/up' undefined
(Font)              using `T1/Merriwthr-OsF/m/n' instead on input line
53.

LaTeX Font Info:    Font shape `T1/Merriwthr-OsF/m/up' will be
(Font)              scaled to size 6.24973pt on input line 53.
LaTeX Font Info:    Font shape `T1/Merriwthr-OsF/m/up' will be
(Font)              scaled to size 5.24997pt on input line 53.
LaTeX Font Info:    Trying to load font information for U+eur on input
line 53.

(c:/texlive/2024/texmf-dist/tex/latex/amsfonts/ueur.fd
File: ueur.fd 2013/01/14 v3.01 Euler Roman
) (c:/texlive/2024/texmf-dist/tex/latex/microtype/mt-eur.cfg
File: mt-eur.cfg 2006/07/31 v1.1 microtype config. file: AMS Euler Roman
(RS)
)

LaTeX Font Warning: Font shape `OMS/cmsy/m/n' in size <7.5> not available
(Font)              size <7> substituted on input line 53.

LaTeX Font Info:    Trying to load font information for U+euf on input
line 53.
```

```
(c:/texlive/2024/texmf-dist/tex/latex/amsfonts/ueuf.fd
File: ueuf.fd 2013/01/14 v3.01 Euler Fraktur
) (c:/texlive/2024/texmf-dist/tex/latex/microtype/mt-euf.cfg
File: mt-euf.cfg 2006/07/03 v1.1 microtype config. file: AMS Euler
Fraktur (RS)

)
LaTeX Font Info:    Trying to load font information for U+eus on input
line 53.

(c:/texlive/2024/texmf-dist/tex/latex/amsfonts/ueus.fd
File: ueus.fd 2013/01/14 v3.01 Euler Script
) (c:/texlive/2024/texmf-dist/tex/latex/microtype/mt-eus.cfg
File: mt-eus.cfg 2006/07/28 v1.2 microtype config. file: AMS Euler Script
(RS)
)
LaTeX Font Info:    Trying to load font information for U+euex on input
line 53
.
(c:/texlive/2024/texmf-dist/tex/latex/amsfonts/ueuex.fd
File: ueuex.fd 2013/01/14 v3.01 Euler extra symbols
)

LaTeX Font Warning: Font shape `OML/cmm/m/it' in size <7.5> not available
(Font)              size <7> substituted on input line 53.

LaTeX Font Info:    Font shape `T1/Merriwthr-OsF/m/n' will be
(Font)              scaled to size 6.24973pt on input line 53.
LaTeX Font Info:    Font shape `T1/Merriwthr-OsF/m/n' will be
(Font)              scaled to size 5.24997pt on input line 53.
LaTeX Font Info:    Font shape `T1/Merriwthr-OsF/m/it' will be
(Font)              scaled to size 7.5pt on input line 53.
LaTeX Font Info:    Font shape `T1/Merriwthr-OsF/m/it' will be
(Font)              scaled to size 6.24973pt on input line 53.
LaTeX Font Info:    Font shape `T1/Merriwthr-OsF/m/it' will be
(Font)              scaled to size 5.24997pt on input line 53.
LaTeX Font Info:    Font shape `T1/Merriwthr-OsF/m/n' will be
(Font)              scaled to size 8.0pt on input line 53.
LaTeX Font Info:    Font shape `T1/Merriwthr-OsF/m/it' will be
(Font)              scaled to size 8.0pt on input line 53.
LaTeX Font Info:    Font shape `T1/Merriwthr-OsF/b/it' will be
(Font)              scaled to size 8.0pt on input line 53.
TextBlockOrigin set to 4pc+6.64pt x 4pc+6pt
<gigascience-logo.pdf, id=121, 99.37125pt x 33.12375pt>
File: gigascience-logo.pdf Graphic file (type pdf)
<use gigascience-logo.pdf>
Package pdftex.def Info: gigascience-logo.pdf  used on input line 66.
(pdftex.def)             Requested size: 126.00902pt x 42.0pt.

Overfull \hbox (54.64pt too wide) in paragraph at lines 66--66
[][]
 []

LaTeX Font Info:    Font shape `T1/Merriwthr-OsF/m/n' will be
```

```
(Font)              scaled to size 14.0pt on input line 66.
LaTeX Font Info:    Font shape `T1/Merriwthr-OsF/m/n' will be
(Font)              scaled to size 8.99997pt on input line 66.
LaTeX Font Info:    Calculating math sizes for size <14> on input line
66.
LaTeX Font Info:    Font shape `T1/Merriwthr-OsF/m/up' will be
(Font)              scaled to size 14.0pt on input line 66.
LaTeX Font Info:    Font shape `T1/Merriwthr-OsF/m/up' will be
(Font)              scaled to size 11.66617pt on input line 66.
LaTeX Font Info:    Font shape `T1/Merriwthr-OsF/m/up' will be
(Font)              scaled to size 9.79996pt on input line 66.
LaTeX Font Info:    Font shape `T1/Merriwthr-OsF/m/n' will be
(Font)              scaled to size 11.66617pt on input line 66.
LaTeX Font Info:    Font shape `T1/Merriwthr-OsF/m/n' will be
(Font)              scaled to size 9.79996pt on input line 66.
LaTeX Font Info:    Font shape `T1/Merriwthr-OsF/m/it' will be
(Font)              scaled to size 14.0pt on input line 66.
LaTeX Font Info:    Font shape `T1/Merriwthr-OsF/m/it' will be
(Font)              scaled to size 11.66617pt on input line 66.
LaTeX Font Info:    Font shape `T1/Merriwthr-OsF/m/it' will be
(Font)              scaled to size 9.79996pt on input line 66.
LaTeX Font Info:    Font shape `T1/Merriwthr-OsF/b/n' will be
(Font)              scaled to size 18.0pt on input line 66.
LaTeX Font Info:    Font shape `T1/Merriwthr-OsF/m/n' will be
(Font)              scaled to size 13.0pt on input line 66.
LaTeX Font Info:    Calculating math sizes for size <13> on input line
66.
LaTeX Font Info:    Font shape `T1/Merriwthr-OsF/m/up' will be
(Font)              scaled to size 13.0pt on input line 66.
LaTeX Font Info:    Font shape `T1/Merriwthr-OsF/m/up' will be
(Font)              scaled to size 10.83287pt on input line 66.
LaTeX Font Info:    Font shape `T1/Merriwthr-OsF/m/up' will be
(Font)              scaled to size 9.09996pt on input line 66.

LaTeX Font Warning: Font shape `OMS/cmsy/m/n' in size <13> not available
(Font)              size <12> substituted on input line 66.


LaTeX Font Warning: Font shape `OMX/cmex/m/n' in size <13> not available
(Font)              size <12> substituted on input line 66.


LaTeX Font Warning: Font shape `OML/cmm/m/it' in size <13> not available
(Font)              size <12> substituted on input line 66.

LaTeX Font Info:    Font shape `T1/Merriwthr-OsF/m/n' will be
(Font)              scaled to size 10.83287pt on input line 66.
LaTeX Font Info:    Font shape `T1/Merriwthr-OsF/m/n' will be
(Font)              scaled to size 9.09996pt on input line 66.
LaTeX Font Info:    Font shape `T1/Merriwthr-OsF/m/it' will be
(Font)              scaled to size 13.0pt on input line 66.
LaTeX Font Info:    Font shape `T1/Merriwthr-OsF/m/it' will be
(Font)              scaled to size 10.83287pt on input line 66.
LaTeX Font Info:    Font shape `T1/Merriwthr-OsF/m/it' will be
```

```
(Font)              scaled to size 9.09996pt on input line 66.
LaTeX Font Info:    Trying to load font information for TS1+Merriwthr-OsF
on in
put line 66.
(c:/texlive/2024/texmf-dist/tex/latex/merriweather/TS1Merriwthr-OsF.fd
File: TS1Merriwthr-OsF.fd 2020/08/30 (autoinst) Font definitions for
TS1/Merriw
thr-OsF.
)
LaTeX Font Info:    Font shape `TS1/Merriwthr-OsF/m/n' will be
(Font)              scaled to size 10.83287pt on input line 66.
Package microtype Info: Loading generic protrusion settings for font
family
(microtype)             `Merriwthr-OsF' (encoding: TS1).
(microtype)             For optimal results, create family-specific
settings.
(microtype)             See the microtype manual for details.
LaTeX Font Info:    Font shape `T1/Merriwthr-OsF/m/n' will be
(Font)              scaled to size 9.0pt on input line 66.
LaTeX Font Info:    Font shape `T1/Merriwthr-OsF/m/up' will be
(Font)              scaled to size 9.0pt on input line 66.
LaTeX Font Info:    Font shape `T1/Merriwthr-OsF/m/up' will be
(Font)              scaled to size 7.0pt on input line 66.
LaTeX Font Info:    Font shape `T1/Merriwthr-OsF/m/up' will be
(Font)              scaled to size 5.0pt on input line 66.
LaTeX Font Info:    Font shape `T1/Merriwthr-OsF/m/n' will be
(Font)              scaled to size 7.0pt on input line 66.
LaTeX Font Info:    Font shape `T1/Merriwthr-OsF/m/n' will be
(Font)              scaled to size 5.0pt on input line 66.
LaTeX Font Info:    Font shape `T1/Merriwthr-OsF/m/it' will be
(Font)              scaled to size 9.0pt on input line 66.
LaTeX Font Info:    Font shape `T1/Merriwthr-OsF/m/it' will be
(Font)              scaled to size 7.0pt on input line 66.
LaTeX Font Info:    Font shape `T1/Merriwthr-OsF/m/it' will be
(Font)              scaled to size 5.0pt on input line 66.
LaTeX Font Info:    Font shape `T1/Merriwthr-OsF/m/n' will be
(Font)              scaled to size 6.5pt on input line 66.
LaTeX Font Info:    Calculating math sizes for size <6.5> on input line
66.
LaTeX Font Info:    Font shape `T1/Merriwthr-OsF/m/up' will be
(Font)              scaled to size 6.5pt on input line 66.
LaTeX Font Info:    Font shape `T1/Merriwthr-OsF/m/up' will be
(Font)              scaled to size 5.41643pt on input line 66.
LaTeX Font Info:    Font shape `T1/Merriwthr-OsF/m/up' will be
(Font)              scaled to size 4.54997pt on input line 66.

LaTeX Font Warning: Font shape `OMS/cmsy/m/n' in size <6.5> not available
(Font)              size <6> substituted on input line 66.


LaTeX Font Warning: Font shape `OMS/cmsy/m/n' in size <5.41643> not
available
(Font)              size <5> substituted on input line 66.
```

```
LaTeX Font Warning: Font shape `OMS/cmsy/m/n' in size <4.54997> not
available
(Font)              size <5> substituted on input line 66.


LaTeX Font Warning: Font shape `OML/cmm/m/it' in size <6.5> not available
(Font)              size <6> substituted on input line 66.


LaTeX Font Warning: Font shape `OML/cmm/m/it' in size <5.41643> not
available
(Font)              size <5> substituted on input line 66.


LaTeX Font Warning: Font shape `OML/cmm/m/it' in size <4.54997> not
available
(Font)              size <5> substituted on input line 66.

LaTeX Font Info:    Font shape `T1/Merriwthr-OsF/m/n' will be
(Font)              scaled to size 5.41643pt on input line 66.
LaTeX Font Info:    Font shape `T1/Merriwthr-OsF/m/n' will be
(Font)              scaled to size 4.54997pt on input line 66.
LaTeX Font Info:    Font shape `T1/Merriwthr-OsF/m/it' will be
(Font)              scaled to size 6.5pt on input line 66.
LaTeX Font Info:    Font shape `T1/Merriwthr-OsF/m/it' will be
(Font)              scaled to size 5.41643pt on input line 66.
LaTeX Font Info:    Font shape `T1/Merriwthr-OsF/m/it' will be
(Font)              scaled to size 4.54997pt on input line 66.
LaTeX Font Info:    Font shape `TS1/Merriwthr-OsF/m/n' will be
(Font)              scaled to size 5.41643pt on input line 66.

Overfull \hbox (54.64pt too wide) in paragraph at lines 66--66
[][][]
 []

LaTeX Font Info:    Font shape `T1/Merriwthr-OsF/b/n' will be
(Font)              scaled to size 10.0pt on input line 66.
LaTeX Font Info:    Font shape `T1/Merriwthr-OsF/b/n' will be
(Font)              scaled to size 8.0pt on input line 66.

Overfull \hbox (54.64pt too wide) in paragraph at lines 66--66
[][][]
 []

Package mdframed Info: mdframed works in twoside mode on input line 69.
LaTeX Font Info:    Font shape `T1/Merriwthr-OsF/b/n' will be
(Font)              scaled to size 8.2pt on input line 69.
LaTeX Font Info:    Font shape `TS1/Merriwthr-OsF/m/n' will be
(Font)              scaled to size 7.5pt on input line 71.
Package mdframed Info: mdframed inside float
 mdframed uses option nobreak mdframed on input line 75.
Package mdframed Info: mdframed inside a box
 mdframed uses option nobreak mdframed on input line 75.
```

```
LaTeX Font Info:    Font shape `T1/Merriwthr-OsF/m/n' will be
(Font)              scaled to size 10.0pt on input line 77.
LaTeX Font Info:    Font shape `T1/Merriwthr-OsF/m/n' will be
(Font)              scaled to size 3.75pt on input line 77.
LaTeX Font Info:    Trying to load font information for T1+MerriwthrSans-
OsF on
 input line 77.
(c:/texlive/2024/texmf-dist/tex/latex/merriweather/T1MerriwthrSans-OsF.fd
File: T1MerriwthrSans-OsF.fd 2020/08/30 (autoinst) Font definitions for
T1/Merr
iwthrSans-OsF.
)
LaTeX Font Info:    Font shape `T1/MerriwthrSans-OsF/m/n' will be
(Font)              scaled to size 3.75pt on input line 77.
Package microtype Info: Loading generic protrusion settings for font
family
(microtype)             `MerriwthrSans-OsF' (encoding: T1).
(microtype)             For optimal results, create family-specific
settings.
(microtype)             See the microtype manual for details.
LaTeX Font Info:    Font shape `T1/Merriwthr-OsF/b/n' will be
(Font)              scaled to size 7.5pt on input line 80.

Package natbib Warning: Citation `gilbert2018current' on page 1 undefined
on in
put line 80.


Package natbib Warning: Citation `ling2015new' on page 1 undefined on
input lin
e 80.


Package natbib Warning: Citation `lu2022metagenome' on page 1 undefined
on inpu
t line 80.


Package natbib Warning: Citation `zhang2024strain' on page 1 undefined on
input
 line 82.


Package natbib Warning: Citation `truong2015metaphlan2' on page 1
undefined on
input line 83.


Package natbib Warning: Citation `blanco2023extending' on page 1
undefined on i
nput line 83.
```

```
Package natbib Warning: Citation `ruscheweyh2021motus' on page 1
undefined on i
nput line 83.


Package natbib Warning: Citation `chen2024melon' on page 1 undefined on
input l
ine 83.


Package natbib Warning: Citation `costea2017metasnv' on page 1 undefined
on inp
ut line 84.


Package natbib Warning: Citation `olm2021instrain' on page 1 undefined on
input
 line 84.


Package natbib Warning: Citation `menzel2016fast' on page 1 undefined on
input
line 85.


Package natbib Warning: Citation `buchfink2015fast' on page 1 undefined
on inpu
t line 85.


Package natbib Warning: Citation `steinegger2017mmseqs2' on page 1
undefined on
 input line 85.


Package natbib Warning: Citation `huson2018megan' on page 1 undefined on
input
line 85.


Package natbib Warning: Citation `maric2024comparative' on page 1
undefined on
input line 86.


Package natbib Warning: Citation `wood2019improved' on page 1 undefined
on inpu
t line 87.


Package natbib Warning: Citation `breitwieser2018krakenuniq' on page 1
undefine
d on input line 87.
```

Package natbib Warning: Citation `lu2017bracken' on page 1 undefined on input l
ine 87.

Package natbib Warning: Citation `kim2016centrifuge' on page 1 undefined on inp
ut line 87.

Package natbib Warning: Citation `song2024centrifuger' on page 1 undefined on i
nput line 87.

Package natbib Warning: Citation `ounit2015clark' on page 1 undefined on input
line 87.

Package natbib Warning: Citation `ounit2016higher' on page 1 undefined on input
 line 87.

Package natbib Warning: Citation `piro2020ganon' on page 1 undefined on input l
ine 87.

Package natbib Warning: Citation `piro2023ganon2' on page 1 undefined on input
line 87.

Package natbib Warning: Citation `ulrich2024fast' on page 1 undefined on input
line 87.

Package natbib Warning: Citation `shaw2023metagenome' on page 1 undefined on in
put line 87.

Package natbib Warning: Citation `dilthey2019strain' on page 1 undefined on inp
ut line 87.

Package natbib Warning: Citation `hong2014pathoscope' on page 1 undefined on in
put line 87.

```
Package natbib Warning: Citation `francis2013pathoscope' on page 1
undefined on
 input line 87.


Package natbib Warning: Citation `curry2022emu' on page 1 undefined on
input li
ne 87.


Package natbib Warning: Citation `zheng2024mora' on page 1 undefined on
input l
ine 87.


Underfull \vbox (badness 10000) has occurred while \output is active []




Underfull \vbox (badness 10000) has occurred while \output is active []



LaTeX Font Info:    Font shape `T1/Merriwthr-OsF/m/n' will be
(Font)              scaled to size 7.8pt on input line 88.
LaTeX Font Info:    Font shape `T1/Merriwthr-OsF/b/n' will be
(Font)              scaled to size 7.8pt on input line 88.
[1{c:/texlive/2024/texmf-
var/fonts/map/pdftex/updmap/pdftex.map}{c:/texlive/202
4/texmf-
dist/fonts/enc/dvips/merriweather/merriwthr_posqbl.enc}{c:/texlive/2024
/texmf-dist/fonts/enc/dvips/merriweather/merriwthr_owzwzj.enc}


{c:/texlive/2024/texmf-
dist/fonts/enc/dvips/merriweather/merriwthr_ags7qn.enc}
<./gigascience-logo.pdf>]

Package natbib Warning: Citation `schaeffer2017pseudoalignment' on page 2
undef
ined on input line 89.


Package natbib Warning: Citation `van2020diversity' on page 2 undefined
on inpu
t line 89.


Package natbib Warning: Citation `luo2011genome' on page 2 undefined on
input l
ine 89.
```

Package natbib Warning: Citation `kashtan2014single' on page 2 undefined on inp
ut line 89.

Package natbib Warning: Citation `schloissnig2013genomic' on page 2 undefined o
n input line 89.

Package natbib Warning: Citation `yassour2018strain' on page 2 undefined on inp
ut line 89.

Package natbib Warning: Citation `van2022strainge' on page 2 undefined on input
 line 93.

Package natbib Warning: Citation `albanese2017strain' on page 2 undefined on in
put line 93.

Package natbib Warning: Citation `roosaare2017strainseeker' on page 2 undefined
 on input line 93.

Package natbib Warning: Citation `siekaniec2021identification' on page 2 undefi
ned on input line 93.

Package natbib Warning: Citation `zhang2024strain' on page 2 undefined on input
 line 94.

Package natbib Warning: Citation `dilthey2019strain' on page 2 undefined on inp
ut line 94.

Package natbib Warning: Citation `kim2016centrifuge' on page 2 undefined on inp
ut line 94.

Package natbib Warning: Citation `song2024centrifuger' on page 2 undefined on i

nput line 94.

Package natbib Warning: Citation `hong2014pathoscope' on page 2 undefined on in
put line 94.

Package natbib Warning: Citation `francis2013pathoscope' on page 2 undefined on
 input line 94.

Package natbib Warning: Citation `zheng2024mora' on page 2 undefined on input l
ine 94.

Package natbib Warning: Citation `maric2024comparative' on page 2 undefined on
input line 96.

Package natbib Warning: Citation `dempster1977maximum' on page 2 undefined on i
nput line 98.

Package natbib Warning: Citation `zheng2024mora' on page 2 undefined on input l
ine 98.

Package natbib Warning: Citation `skoufos2022agamemnon' on page 2 undefined on
input line 98.

Package natbib Warning: Citation `van2022strainge' on page 2 undefined on input
 line 100.

Package natbib Warning: Citation `albanese2017strain' on page 2 undefined on in
put line 100.

Package natbib Warning: Citation `jain2018high' on page 2 undefined on input li
ne 100.

```
Package natbib Warning: Citation `koslicki2024yacht' on page 2 undefined
on inp
ut line 100.


Package natbib Warning: Citation `van2020diversity' on page 2 undefined
on inpu
t line 100.



Package natbib Warning: Citation `anyansi2020computational' on page 2
undefined
 on input line 102.


Package natbib Warning: Citation `lapierre2020metalign' on page 2
undefined on
input line 102.


Package natbib Warning: Citation `koslicki2019improving' on page 2
undefined on
 input line 102.


Underfull \hbox (badness 2556) in paragraph at lines 104--106
[]\T1/Merriwthr-OsF/m/n/7.5 (+20) In this work, we in-tro-duce MADRe, a
pipelin
e for long-
 []


Underfull \hbox (badness 2707) in paragraph at lines 104--106
\T1/Merriwthr-OsF/m/n/7.5 (+20) read, strain-level metage-nomic clas-si-
fi-ca-t
ion en-hanced with
 []


Underfull \hbox (badness 2503) in paragraph at lines 107--108
[]\T1/Merriwthr-OsF/m/n/7.5 (+20) In the sec-ond, read clas-si-fi-ca-tion
step,
 MADRe per-forms
 []

LaTeX Font Info:    Font shape `T1/Merriwthr-OsF/b/n' will be
(Font)              scaled to size 8.5pt on input line 114.
LaTeX Font Info:    Font shape `T1/Merriwthr-OsF/m/n' will be
(Font)              scaled to size 8.5pt on input line 114.

Package natbib Warning: Citation `faure2024hairsplitter' on page 2
undefined on
```

```
 input line 120.



LaTeX Font Info:    Font shape `T1/Merriwthr-OsF/m/it' will be
(Font)              scaled to size 7.8pt on input line 121.
[2]

Package natbib Warning: Citation `ulrich2024fast' on page 3 undefined on
input
line 137.


Package natbib Warning: Citation `zheng2024mora' on page 3 undefined on
input l
ine 137.


Package natbib Warning: Citation `kolmogorov2020metaflye' on page 3
undefined o
n input line 139.


Package natbib Warning: Citation `benoit2024high' on page 3 undefined on
input
line 139.


Package natbib Warning: Citation `shaw2025high' on page 3 undefined on
input li
ne 139.




Package natbib Warning: Citation `wick2019badread' on page 3 undefined on
input
 line 149.


Underfull \hbox (badness 10000) in paragraph at lines 153--154
[]\T1/Merriwthr-OsF/b/n/7.5 (+20) sim_expanded (30 strains)
\T1/Merriwthr-OsF/m
/n/7.5 (+20) ^^U An ex-ten-sion of the
 []


Package natbib Warning: Citation `jain2018high' on page 3 undefined on
input li
ne 155.


Package natbib Warning: Citation `zhang2024strain' on page 3 undefined on
input
```

```
 line 157.


Package natbib Warning: Citation `zhang2025pantax_datasets' on page 3
undefined
 on input line 157.


Package natbib Warning: Citation `liu2022nanopore' on page 3 undefined on
input
 line 159.


Package natbib Warning: Citation `portik2022evaluation' on page 3
undefined on
input line 159.


Package natbib Warning: Citation `sereika2022oxford' on page 3 undefined
on inp
ut line 161.


Package natbib Warning: Citation `o2016reference' on page 3 undefined on
input
line 165.



[3]
LaTeX Font Info:    Font shape `T1/Merriwthr-OsF/b/n' will be
(Font)              scaled to size 7.0pt on input line 178.

Overfull \hbox (5.76605pt too wide) in paragraph at lines 181--206
 [][]
 []




Package natbib Warning: Citation `bray1957ordination' on page 4 undefined
on in
put line 233.



[4]

Package natbib Warning: Citation `zymobiomics' on page 5 undefined on
input lin
e 264.
```

```
Package natbib Warning: Citation `chen2024melon' on page 5 undefined on input l
ine 264.


Package natbib Warning: Citation `curry2022emu' on page 5 undefined on input li
ne 264.


Package natbib Warning: Citation `feng2022metagenome' on page 5 undefined on in
put line 264.




[5]
Underfull \hbox (badness 10000) in paragraph at lines 312--312
|\T1/Merriwthr-OsF/b/n/8.5 (+20) Classification of real anaer-o-bic di-gester s
ludge
 []

LaTeX Font Info:    Font shape `T1/Merriwthr-OsF/m/up' will be
(Font)              scaled to size 7.5pt on input line 328.



Underfull \vbox (badness 10000) has occurred while \output is active []



[6]

Package natbib Warning: Citation `vicedomini2021strainberry' on page 7 undefine
d on input line 370.


Package natbib Warning: Citation `luo2022enhancing' on page 7 undefined on inpu
t line 370.


Package natbib Warning: Citation `luo2022enhancing' on page 7 undefined on inpu
t line 370.


Package natbib Warning: Citation `kazantseva2024strainy' on page 7 undefined on
 input line 370.
```

```
Package natbib Warning: Citation `faure2024hairsplitter' on page 7
undefined on
 input line 370.


Underfull \hbox (badness 1122) in paragraph at lines 372--373
[]\T1/Merriwthr-OsF/m/up/7.5 (+20) Most ex-ist-ing strain-level clas-si-
fiers e
i-ther re-quire single-
 []




Package natbib Warning: Citation `simon2019benchmarking' on page 7
undefined on
 input line 381.



[7]


Package natbib Warning: Citation `zheng2024mora' on page 8 undefined on
input l
ine 412.


Package natbib Warning: Citation `francis2013pathoscope' on page 8
undefined on
 input line 412.


Package natbib Warning: Citation `hong2014pathoscope' on page 8 undefined
on in
put line 412.


Package natbib Warning: Citation `curry2022emu' on page 8 undefined on
input li
ne 412.


Package natbib Warning: Citation `sapoval2024lightweight' on page 8
undefined o
n input line 412.


Package natbib Warning: Citation `skoufos2022agamemnon' on page 8
undefined on
input line 412.
```

```
Package natbib Warning: Citation `francis2013pathoscope' on page 8
undefined on
 input line 412.


Package natbib Warning: Citation `curry2022emu' on page 8 undefined on
input li
ne 412.



[8]

LaTeX Font Info:    Font shape `T1/Merriwthr-OsF/b/sl' in size <7.5> not
availa
ble
(Font)              Font shape `T1/Merriwthr-OsF/b/it' tried instead on
input l
ine 543.
LaTeX Font Info:    Font shape `T1/Merriwthr-OsF/b/it' will be
(Font)              scaled to size 7.5pt on input line 543.


[9]

Package natbib Warning: Citation `van2020diversity' on page 10 undefined
on inp
ut line 553.


Package natbib Warning: Citation `wick2019badread' on page 10 undefined
on inpu
t line 566.



LaTeX Font Info:    Trying to load font information for T1+lmtt on input
line 6
05.
(c:/texlive/2024/texmf-dist/tex/latex/lm/t1lmtt.fd
File: t1lmtt.fd 2015/05/01 v1.6.1 Font defs for Latin Modern
)
Package microtype Info: Loading generic protrusion settings for font
family
(microtype)             `lmtt' (encoding: T1).
(microtype)             For optimal results, create family-specific
settings.
(microtype)             See the microtype manual for details.


[10{c:/texlive/2024/texmf-dist/fonts/enc/dvips/lm/lm-ec.enc}]
```

```
Package natbib Warning: Citation `liu2022nanopore' on page 11 undefined
on inpu
t line 618.


Package natbib Warning: Citation `portik2022evaluation' on page 11
undefined on
 input line 618.

No file MADRe_main.bbl.

LaTeX Warning: File `figures/Fig1-MADRe_pipeline.pdf' not found on input
line 6
55.


! Package pdftex.def Error: File `figures/Fig1-MADRe_pipeline.pdf' not
found: u
sing draft setting.

See the pdftex.def package documentation for explanation.
Type  H <return>  for immediate help.
 ...

l.655 ...xtwidth]{figures/Fig1-MADRe_pipeline.pdf}

Try typing  <return>  to proceed.
If that doesn't work, type  X <return>  to quit.

LaTeX Font Info:    Font shape `T1/Merriwthr-OsF/m/n' will be
(Font)              scaled to size 6.0pt on input line 657.
LaTeX Font Info:    Font shape `T1/Merriwthr-OsF/b/n' will be
(Font)              scaled to size 6.0pt on input line 657.

LaTeX Warning: File `figures/Fig2-F1_scores_medium_size_datasets.pdf' not
found
 on input line 661.


! Package pdftex.def Error: File `figures/Fig2-
F1_scores_medium_size_datasets.p
df' not found: using draft setting.

See the pdftex.def package documentation for explanation.
Type  H <return>  for immediate help.
 ...

l.661 .../Fig2-F1_scores_medium_size_datasets.pdf}

Try typing  <return>  to proceed.
If that doesn't work, type  X <return>  to quit.
```

```
LaTeX Warning: File `figures/Fig3-
Number_of_identified_organisms_medium_size_da
tasets.pdf' not found on input line 666.


! Package pdftex.def Error: File `figures/Fig3-
Number_of_identified_organisms_m
edium_size_datasets.pdf' not found: using draft setting.

See the pdftex.def package documentation for explanation.
Type  H <return>  for immediate help.
 ...

l.666 ...ified_organisms_medium_size_datasets.pdf}

Try typing  <return>  to proceed.
If that doesn't work, type  X <return>  to quit.


LaTeX Warning: File `figures/Fig4-F1_scores_large_size_datasets.pdf' not
found
on input line 672.


! Package pdftex.def Error: File `figures/Fig4-
F1_scores_large_size_datasets.pd
f' not found: using draft setting.

See the pdftex.def package documentation for explanation.
Type  H <return>  for immediate help.
 ...

l.672 ...s/Fig4-F1_scores_large_size_datasets.pdf}

Try typing  <return>  to proceed.
If that doesn't work, type  X <return>  to quit.


LaTeX Warning: File `figures/Fig5-BC_distances_zymo.pdf' not found on
input lin
e 678.


! Package pdftex.def Error: File `figures/Fig5-BC_distances_zymo.pdf' not
found
: using draft setting.

See the pdftex.def package documentation for explanation.
Type  H <return>  for immediate help.
 ...

l.678 ...idth]{figures/Fig5-BC_distances_zymo.pdf}

Try typing  <return>  to proceed.
```

```
If that doesn't work, type  X <return>  to quit.


LaTeX Warning: File `figures/Fig6-rank_abundances_real_data.pdf' not
found on i
nput line 683.


! Package pdftex.def Error: File `figures/Fig6-
rank_abundances_real_data.pdf' n
ot found: using draft setting.

See the pdftex.def package documentation for explanation.
Type  H <return>  for immediate help.
 ...

l.683 ...gures/Fig6-rank_abundances_real_data.pdf}

Try typing  <return>  to proceed.
If that doesn't work, type  X <return>  to quit.


LaTeX Warning: File `figures/Fig7-relative_abundances_real_data.pdf' not
found
on input line 688.


! Package pdftex.def Error: File `figures/Fig7-
relative_abundances_real_data.pd
f' not found: using draft setting.

See the pdftex.def package documentation for explanation.
Type  H <return>  for immediate help.
 ...

l.688 ...s/Fig7-relative_abundances_real_data.pdf}

Try typing  <return>  to proceed.
If that doesn't work, type  X <return>  to quit.


LaTeX Warning: File `figures/Fig8-computational_resources.pdf' not found
on inp
ut line 693.


! Package pdftex.def Error: File `figures/Fig8-
computational_resources.pdf' not
 found: using draft setting.

See the pdftex.def package documentation for explanation.
Type  H <return>  for immediate help.
 ...
```

```
l.693 ...figures/Fig8-computational_resources.pdf}

Try typing  <return>  to proceed.
If that doesn't work, type  X <return>  to quit.


Package natbib Warning: There were undefined citations.




[11

] [12] [13] [14] [15] [16] [17] [18] [19]
enddocument/afterlastpage: lastpage setting LastPage.
(./MADRe_main.aux)
 ***********
LaTeX2e <2024-06-01> patch level 2
L3 programming layer <2020/03/25>
 ***********

LaTeX Font Warning: Size substitutions with differences
(Font)              up to 1.0pt have occurred.


LaTeX Font Warning: Some font shapes were not available, defaults
substituted.


LaTeX Warning: There were multiply-defined labels.

Package rerunfilecheck Info: File `MADRe_main.out' has not changed.
(rerunfilecheck)             Checksum:
E25649B1975C1822DA61035736629690;4958.
 )
Here is how much of TeX's memory you used:
 25798 strings out of 473583
 506737 string characters out of 5732343
 2109908 words of memory out of 5000000
 47586 multiletter control sequences out of 15000+600000
 1882505 words of font info for 566 fonts, out of 8000000 for 9000
 1141 hyphenation exceptions out of 8191
 123i,13n,131p,1537b,1101s stack positions out of
10000i,1000n,20000p,200000b,200000s
<c:/texlive/2024/texmf-dist/fonts/type1/sorkin/merriweather/Merriwthr-
Bold.pf
b><c:/texlive/2024/texmf-dist/fonts/type1/sorkin/merriweather/Merriwthr-
BoldIta
lic.pfb><c:/texlive/2024/texmf-
dist/fonts/type1/sorkin/merriweather/Merriwthr-I
talic.pfb><c:/texlive/2024/texmf-
dist/fonts/type1/sorkin/merriweather/Merriwthr
```

```
-Regular.pfb><c:/texlive/2024/texmf-
dist/fonts/type1/sorkin/merriweather/Merriw
thrSans-Regular.pfb><c:/texlive/2024/texmf-
dist/fonts/type1/public/amsfonts/cme
xtra/cmex7.pfb><c:/texlive/2024/texmf-
dist/fonts/type1/public/amsfonts/cm/cmsy6
.pfb><c:/texlive/2024/texmf-
dist/fonts/type1/public/amsfonts/cm/cmsy7.pfb><c:/t
exlive/2024/texmf-
dist/fonts/type1/public/amsfonts/euler/euex8.pfb><c:/texlive/
2024/texmf-
dist/fonts/type1/public/amsfonts/euler/eufm7.pfb><c:/texlive/2024/te
xmf-
dist/fonts/type1/public/amsfonts/euler/eurm7.pfb><c:/texlive/2024/texmf-
dis
t/fonts/type1/public/amsfonts/euler/eusm7.pfb><c:/texlive/2024/texmf-
dist/fonts
/type1/public/lm/lmtt8.pfb>
Output written on MADRe_main.pdf (19 pages, 502493 bytes).
PDF statistics:
 350 PDF objects out of 1000 (max. 8388607)
 297 compressed objects within 3 object streams
 73 named destinations out of 1000 (max. 500000)
 211694 words of extra memory for PDF output out of 221844 (max.
10000000)
```

Main manuscript file

Click here to access/download;Manuscript;MADRe-GigaScience.pdf

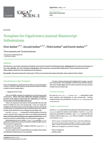

GigaScience, 2023, 1–17

doi: xx.xxxx/xxxx
Manuscript in Preparation
Technical Note

TECHNICAL NOTE

MADRe: Strain-Level Metagenomic Classification Through Assembly-Driven Database Reduction

Josipa Lipovac[1,*], Mile Šikić[1,2], Riccardo Vicedomini[3,†] and Krešimir Križanović[2,*†]

[1]Laboratory for Bioinformatics and Computational Biology, Faculty of Electrical Engineering and Computing, University of Zagreb, Zagreb, Croatia and [2]Laboratory of AI in Genomics, Genome Institute of Singapore, A*STAR, Singapore, Singapore and [3]Univ Rennes, CNRS, Inria, IRISA - UMR 6074, F-35000 Rennes, France

[*]josipa.lipovac@fer.unizg.hr; kresimir.krizanovic@fer.unizg.hr
[†]Contributed equally.

Abstract

Strain-level metagenomic classification is essential for understanding microbial diversity and functional potential, but remains challenging, particularly in the absence of prior knowledge about the composition of the sample. In this paper we present MADRe, a modular and scalable pipeline for long-read strain-level metagenomic classification, enhanced with **M**etagenome **A**ssembly-Driven **D**atabase **Re**duction. MADRe combines long-read metagenome assembly, contig-to-reference mapping reassignment based on an expectation-maximization algorithm for database reduction, and probabilistic read mapping reassignment to achieve sensitive and precise classification. We extensively evaluated MADRe on simulated datasets, mock communities, and a real anaerobic digester sludge metagenome, demonstrating that it consistently outperforms existing tools by achieving higher precision with reduced false positives. MADRe's design allows users to apply either the database reduction or read classification step individually. Using only the read classification step shows results on par with other tested tools. MADRe is open source and publicly available at https://github.com/lbcb-sci/MADRe.

Key words: metagenomics; strain-level; metagenomic classification; database reduction

Background

Metagenomics enables the study of genetic material from complex microbial communities found in environments such as human gut, soil, or marine ecosystems. It provides a comprehensive view of microbial diversity and interactions within these environments [1, 2]. A central challenge in metagenomic analysis is the accurate identification of organisms present in a sample, typically performed by comparing sequencing reads to reference genome databases [3].

A wide range of metagenomic classification tools have been developed, which can be broadly categorized into marker-based, DNA-to-protein and DNA-to-DNA approaches, as described in [4]. Marker-based tools, such as MetaPhlAn [5, 6], StrainPhlAn, mOTUs [7], and Melon [8], classify taxa using conserved, clade-specific marker genes. In addition to marker-based methods, SNV-based profilers (e.g., metaSNV [9] and InStrain [10], which combines both approaches) represent important strategies for strain detection and population tracking. However, most of these approaches are optimized for short-read data and rely on predefined marker sets or variant catalogs, which may not fully capture genomic diversity in complex or underrepresented microbial communities. DNA-to-protein tools, including Kaiju [11], DIAMOND [12], MMseqs2 [13], and MEGAN-LR [14] translate reads into amino acid sequences before aligning them to protein databases. DNA-to-DNA tools compare reads directly against genomic sequences and are commonly divided into k-mer-based and mapping-based tools [15]. K-mer-based tools such as Kraken2 [16], KrakenUniq [17], Bracken [18], Centrifuge [19], Centrifuger [20], CLARK/CLARK-S [21, 22], Ganon [23, 24], Taxor [25], and Sylph [26] are known for their speed and scalability to large databases, but often trade precision

Compiled on: November 6, 2025.
Draft manuscript prepared by the author.

Key Points

- MADRe implements a two-step strategy for strain-level classification: it first identifies candidate strains via assembly-to-database mapping with EM-based reassignment, then classifies reads by mapping to a reduced database via probabilistic reassignment.
- Despite incorporating assembly, MADRe significantly reduces runtime and memory usage compared to mapping all reads to the full reference database.
- MADRe enables the use of large, diverse reference databases without prior knowledge of sample content, focusing classification on confidently assembled strains and substantially reducing false positives while maintaining high strain-level resolution.

for speed. In contrast, mapping-based tools such as MetaMaps [27], PathoScope2 [28, 29], EMU [30] and MORA [31], which rely on read alignments and reassignment algorithms, offer higher precision at a greater computational cost.

Although k-mer-based tools, especially Kraken2 or Sylph, perform well at the species level, strain-level classification becomes increasingly challenging when sequences originate from closely related genomes [32]. However, resolving strain-level diversity is essential, as even closely related strains can exhibit substantial differences in gene content and function, with implications for microbial ecology, pathogenesis, and treatment outcomes [33, 34, 35, 36, 37].

While most existing tools are optimized for short reads due to their low cost and high accuracy, long-read sequencing technologies such as Oxford Nanopore and PacBio HiFi are rapidly improving. Longer read lengths provide advantages for genome assembly, structural-variant detection, and improved strain-level resolution.

Several short-read-based tools are designed for strain-level classification within a single species, such as StrainGE [38], StrainEST [39], and StrainSeeker [40], as well as the long-read-based ORI [41]. Other tools, including PanTax [4], MetaMaps [27], Centrifuge [19], Centrifuger [20], PathoScope2 [28, 29], and MORA [31], are suitable for more complex, multi-species datasets and support short- and long-read strain-level metagenomic classification. PanTax is a pangenome-based approach that, while supporting multi-species datasets, faces scalability limitations when applied to very large reference databases. MetaMaps is a mapping-based tool capable of high-resolution classification but is known to be extremely computationally demanding [15]. Centrifuge is a k-mer-based tool designed to perform strain-level classification, but like PanTax, it encounters limitations when constructing indexes for very large reference databases. However, its successor, Centrifuger, introduces improved compression and indexing strategies that enable efficient classification across large-scale genome databases. PathoScope2 is an older tool that is no longer maintained and cannot be reliably executed due to outdated dependencies and software incompatibilities. Originally developed for strain-level classification of short reads, it is based on an expectation-maximization (EM) algorithm for read reassignment [42]. As part of the MORA study, the authors introduced a continuation of PathoScope2, referred to as AugPatho, which includes a modified version of the original algorithm adapted for use with long reads [31]. MORA extends this approach by combining the EM algorithm from Agamemnon [43] with a read reassignment strategy based on the Weapon-Target Assignment (WTA) problem. According to its authors, MORA represents the current state-of-the-art in mapping-based long-read metagenomic classification.

Reference databases often contain multiple assemblies of the same strain and typically lack consistent organization. To address this, some strain-level classification tools perform database preclustering according to Average Nucleotide Identity (ANI) scores. Previous studies have shown that there is no universal ANI threshold for defining strains [38, 39, 44, 45, 33]. Setting the threshold too high may erroneously separate assemblies of the same strain, whereas setting a threshold too low may incorrectly group different strains together.

Using large and diverse databases is important for accurate strain-level classification [46], but it also makes the analysis much more demanding to run. MetaAlign [47] uses containment MinHash [48] to reduce the reference database prior to alignment, improving runtime while maintaining high species-level precision. However, it is primarily designed for short reads, and strain-level resolution is not its main focus.

In this work, we introduce MADRe, a pipeline for long-read, strain-level metagenomic classification enhanced with Metagenome Assembly-Driven Database Reduction, consisting of two main phases: database reduction and read classification. In the database reduction step, MADRe combines long-read assembly with an EM algorithm that assigns assembled contigs to one or more references, reducing the reference database.

In the second, read classification step, MADRe performs mappings-based read reassignment. It resolves ambiguous read mappings by assigning each read to the most likely reference, based on mapping scores and probabilistic support.

We conducted an extensive evaluation of MADRe using simulated datasets, Zymo mock communities, and a real anaerobic digester sludge metagenome. The results demonstrate that MADRe achieves high precision and strain-level resolution while maintaining lower memory usage and runtime compared to existing tools. Additionally, the two steps of the MADRe pipeline can be run independently, and our results show that the read classification module (MADRe_RC) alone performs competitively. MADRe's approach enables the use of large, diverse reference databases spanning multiple taxonomic levels, making it well-suited for scenarios where no prior knowledge about the sample is available. Using assembled contigs to detect potentially present strains, MADRe focuses on confidently represented organisms. As a result, compared to state-of-the-art tools, it significantly reduces the number of false positive identifications while maintaining high resolution strain-level classification.

Results

MADRe - method overview

The MADRe pipeline is designed for strain-level metagenomic classification, particularly in scenarios where prior knowledge of the sample composition is not available. Its primary goal is to enable accurate strain identification while reliably distinguishing truly abundant strains from false positives.

As illustrated in Figure 1, the MADRe pipeline consists of two main steps: database reduction and read classification. It takes as input a large bacterial reference database and raw long metagenomic reads, and produces two main outputs: a read classification file and a reference abundance file.

In the database reduction step, long reads are first assembled, and the resulting contigs are mapped to the large reference database. MADRe employs HairSplitter [49] to estimate the number of collapsed strains represented by each contig. Using both contig-to-reference mappings and the collapsed strain estimates, an EM algo-

rithm is applied, followed by additional read-reassignment steps. This process generates a reduced reference database that highlights strains likely present in the sample. Although this reduction effectively narrows the search space, it may still include false positives introduced during the assembly process. These could be further filtered during the read classification step.

In the second step, reads are mapped to the reduced reference database, and ambiguous mappings are resolved through a reassignment procedure that leverages mapping scores and probabilistic support.

Both steps of the pipeline can be run independently, so when prior knowledge about the sample exists or when a reduced reference set is already available, the read classification step can be used on its own.

Benchmarking details

We benchmarked the MADRe pipeline against state-of-the-art tools developed for the same purpose: handling large reference databases while enabling strain-level classification. These tools include MORA and AugPatho (PathoScope2), for which we evaluated both of its key modules, PathoID and PathoReport. In some of the experiments, we also evaluated k-mer based tool Kraken2, one of the most widely used metagenomic classification tools, which is often considered the standard for species-level classification. Although Kraken2 is capable of assigning reads at the strain level, its evaluation is complicated by the use of taxonomic identifiers (taxIDs) that may refer to either species or strain ranks. Despite its popularity, recent work has shown that Sylph achieves superior performance for species-level abundance estimation, reporting fewer false positives. However, since Sylph is primarily designed for abundance profiling rather than direct read classification, we did not include it in our benchmarking, which focuses explicitly on classification accuracy. Additionally, we included MADRe_RC, a variant of MADRe that performs only the second step (*i.e.*, read classification) without prior database reduction.

We did not include MetaMaps, PanTax, or Centrifuge in our benchmarking analysis. In the case of MetaMaps, previous studies have reported crashes when attempting to build an index for the full Genome Taxonomy Database (GTDB), highlighting its scalability limitations [25, 31]. Similarly, our attempts to construct the same reference database for PanTax and Centrifuge, used successfully with other benchmarking tools, also failed due to crashes during the indexing process. Instead, we evaluated Centrifuger, a recent successor of Centrifuge that introduces improved compression and indexing strategies, enabling efficient classification on large-scale genome databases.

By default, MADRe employs metaFlye [50] for assembling Oxford Nanopore (ONT) reads and metaMDBG [51] for assembling PacBio HiFi reads. To assess the effect of different assembly strategies on database reduction, we also performed additional experiments using Myloasm [52], a recently developed assembler showing promising performance on metagenomic datasets.

All commands used to run the benchmarking tools are available in the Supplementary File (Tools versions and commands).

Datasets

As part of the benchmarking process, we evaluated the mentioned tools on simulated metagenome datasets, Zymo mock communities, and a real anareobic digester sludge metagenome.

For medium-sized simulated datasets, we selected a smaller subset of genomes representing species commonly found in the human gut microbiome. Reference genomes were required to be labeled as "complete" or "chromosome" in NCBI, and to have a strain-level taxID distinct from their species-level taxID. This criterion ensured that Kraken2 could be included in the evaluation.

Using Badread tool [53] we simulated three different metagenomic datasets:

i. **sim_small (4 strains)** – This dataset includes four different strain references: two strains from *Adlercreutzia equolifaciens* and two from *Streptococcus anginosus*, with varying relative abundances.

ii. **sim_medium (15 strains)** – This dataset contains 15 strain references distributed across five bacterial species (*i.e.*, *Helicobacter pylori*, *Cutibacterium acnes*, *Streptococcus intermedius*, *Streptococcus mutans*, *Lactococcus lactis*), with each species represented by three strains. At species level the abundances are different while strains of one species are equally abundant.

iii. **sim_expanded (30 strains)** – An extension of the sim_medium dataset, incorporating 15 additional strains from distinct species and maintaining variable abundance levels across species. Newly added species are listed in Supplementary Table ST1.

Exact genome information including accession numbers, strain and species taxIDs, genome lengths, genome coverages, ANI values (calculated usin fastANI [44], and number of simulated reads can be found in the Supplementary Tables (ST1-ST4).

Although these datasets can be used to assess MADRe's performance, they remain relatively simple and do not fully reflect the complexity of real metagenomic samples. Therefore, we expanded our benchmarking to include four additional simulated datasets originally used in the PanTax study [4] and we called them large-sized simulated datasets. Three of these datasets each contain 60 genomes coming from 30 species, simulated using ONT R9.4.1, ONT R10.4.1, and PacBio HiFi error profiles, respectively. These datasets were obtained directly from the PanTax Zenodo repository [54]. In addition, we generated a fourth, large-scale dataset comprising 1000 genomes from over 300 species, inspired by the CAMI challenge design. As simulated reads for this dataset were not available due to its size, we used the published reference genomes and expected abundances to simulate reads with the Badread tool. For these datasets, we additionally present distributions of ANI scores (calculated using fastANI), illustrating how many genome pairs exceed predefined ANI thresholds, as shown in Supplementary Table ST10.

We also tested MADRe using three Zymo mock communities: D6322 (ONT), D6331 (ONT) [55], and D6331 (PacBio HiFi) [56]. We included both D6311 ONT and D6311 PacBio HiFi datasets to demonstrate the pipeline's capability across different sequencing technologies.

To evaluate performance on a complex real-world dataset, we analyzed an anaerobic digester sludge metagenome dataset sequenced using ONT R10.4.1. reads [57]. This real metagenome represents the type of scenario for which MADRe is designed, where a highly diverse sample is analyzed without prior knowledge of its taxonomic composition.

Database

To thoroughly evaluate strain-level classification and ensure sufficient taxonomic divergence for accurate strain-level detection, we used a database obtained via the Kraken2 interface by selecting the bacterial database. This database consists of 102,639 sequences, encompassing all RefSeq [58] complete bacterial genomes. The database was downloaded in December 2024. The exact command used for downloading the database is provided in the Supplementary File (Tools versions and commands).

For consistency, the same database was used across all tools and experiments.

Database Reduction

Table 1. Database reduction results. Comparison of MADRe's database reduction performance with two baseline models on simulated datasets using large database containing **102,639** sequences: baseline model 1 (BM1), which includes only the top-1 mapping for each contig, and baseline model 2 (BM2), which includes the top-3 mappings.

	Metric	sim_small	sim_medium	sim_expanded
number of genomes in dataset		4	15	30
**BM1**	# in reduced	7	34	68
	# missing strains	0	1	3
	# FP strains	3	19	38
	# FP species	1	0	6
**BM2**	# in reduced	14	107	234
	# missing strains	0	0	0
	# FP strains	10	92	204
	# FP species	5	11	78
**MADRe database reduction**	# in reduced	7	42	84
	# missing strains	0	0	0
	# FP strains	3	27	54
	# FP species	1	0	6

To assess the effectiveness of strain identification and database reduction, we evaluated the output of MADRe's database reduction step on simulated datasets by comparing it to two baseline models. More precisely, assembled contigs were mapped to the large reference database and the identification of organisms was carried out using the following strategies:

- Baseline Model 1 (BM1): For the reduced database, each contig's top reference genome, determined by the highest summarized harmonic mean mapping value (Methods, Equation 2), was included without performing any reassignment steps. BM1 represents the ideal reduction level under the assumption of a perfect assembly, where each contig corresponds to a single strain reference.
- Baseline Model 2 (BM2): For the reduced database, each contig's top three reference genomes, based on the highest summarized harmonic mean mapping values (Methods, Equation 2), were included without performing any reassignment steps. BM2 defines an upper bound on the number of references expected in the reduced database. Since our simulated metagenomes contain at most three strains per species, we assume that at most three strains could be collapsed into a single contig. This approach allows us to determine how many references should be retained in the reduced database to ensure that no true reference from the sample is missed.

The results, presented in Table 1, demonstrate that MADRe's database reduction achieves a high level of reduction while successfully retaining all expected strains. Additionally, when compared to BM2, MADRe reduces the number of false positive species, further highlighting its effectiveness.

Classification of medium-sized simulated datasets

For medium-sized simulated datasets we compared the classification performance of MADRe, MADRe_RC, MORA, AugPatho (in both PathoID and PathoReport modes), Kraken2, and Centrifuger. Since the exact source of each read is known, we define classification outcomes as follows: true positive (TP) if the read is classified under the expected strain (or expected cluster), true negative (TN) if the read is not classified and its Badread label is *random* or *junk*, false positive (FP) if the read is classified under the incorrect strain (or incorrect cluster), and false negative (FN) if the read is not classified but its label is different from *random* or *junk*.

We evaluated classification of simulated data with and without post-clustering. The post-clustering method described in the Methods section groups closely related strains based on read-to-reference mappings and assigns reads to clusters instead of individual strains. The same clustering approach was applied to all tools, utilizing read mappings to the full reference database. Kraken2's clustering results are not included, as taxID alone does not allow for an accurate evaluation of post-clustering performance.

The classification results of medium-sized simulated datasets (sim_small, sim_medium and sim_expanded) are presented in Figure 3 A., which shows the F1 scores for classification with and without post-clustering. The results demonstrate that both MADRe and MADRe_RC outperform all other approaches. Interestingly, on the sim_small dataset, which includes differently abundant strains of the same species, Kraken2 performs slightly better than Centrifuger, while Centrifuger achieves higher scores than MORA and AugPatho. For the remaining datasets, Centrifuger performs better than Kraken2 but worse than MORA and AugPatho. In the sim_expanded dataset (without clustering), MORA outperforms AugPatho's modes, but in all other cases, including all clustering scenarios, both AugPatho's modes perform significantly better than MORA. When post-clustering is applied, Centrifuger shows the lowest performance across all datasets, while post-clustering further improves AugPatho's results, bringing them close to MADRe_RC. Overall, MADRe_RC achieves performance comparable to MADRe, although this difference becomes more pronounced on more complex datasets. Figure 3B. shows the number of organisms identified by different tools on simulated datasets. An organism is considered identified if at least one read is classified under it. In all cases, there were no false negatives — all tools successfully identified the expected organisms. However, the number of additional (false positive) identifications varies. MADRe consistently reports significantly fewer false positives. For example, in the sim_small dataset, only 6 organisms were reported compared to the 4 expected, with 2 of them being extremely similar strains to those actually present in the sample. Additional metrics for organism-level identification, including TPs, FPs, FNs, accuracy, precision, recall, and F1 scores, are provided in Supplementary Table ST8, while classified read counts for each organism are listed in Supplementary Tables (ST5–ST7).

In addition, a Supplementary Figure S4 and Supplementary Table ST9 show Bray–Curtis (BC) distances [59] between the observed read count abundances and the ground-truth abundances, offering further insight into the similarity between the predicted and true community compositions. BC distance is one of the most commonly used distances to calculate the microbial abundance differences, and is described in the Methods section. From these results, it is evident that MADRe achieves the closest match to the ground truth, while AugPatho ID reports the poorest scores among strain-level tools, including Centrifuger. Kraken2, although computationally efficient, shows the weakest overall performance.

Classification of large-sized simulated datasets

To further assess classification performance under more realistic metagenomic conditions, we used the simulated datasets from the *PanTax* study. We used the same evaluation procedure as the one considered for the medium-sized simulated datasets. In addition to benchmarking the standard MADRe pipeline, we also evaluated a variant in which Myloasm was used as the assembler during the database reduction step, in order to examine how different assembly approaches influence MADRe's performance. This variant was not tested on the sim_low R9.4.1 dataset, as Myloasm is not suitable for reads with that error profile.

Figure 4 presents four radar plots, each corresponding to one of the four large sized datasets, and showing F1 scores for all evaluated tools, both with and without post-clustering. (Note that post-clustering values for Kraken2 are zero, since this step was not performed for that tool.) For each tool, the best obtained F1 score is indicated in parentheses beneath its name. Across most datasets, MADRe achieved the highest scores, including its versions using different assemblers. In some cases, MADRe_RC slightly outperformed MADRe, particularly on the sim_high dataset. Detailed evaluation statistics are provided in Supplementary Table ST15. Exact read counts obtained from classifications are listed in Supplementary Tables (ST11–ST14), while Bray–Curtis (BC) distances between observed and expected read-count abundances are shown in Supplementary Figure S5 and Supplementary Table ST16.

Inspection of read-count abundances in the sim_high dataset revealed that several genomes were not detected during the database reduction step, leading to a modest decrease in MADRe's performance compared to MADRe_RC.

When comparing assembler performance, MADRe runs based on Myloasm assemblies achieved slightly higher F1 scores than those using metaFlye. A closer look at read-count abundances revealed that 24 genomes were detected exclusively with Myloasm and 11 exclusively with metaFlye. Among the 11 missed by Myloasm, 9 belonged to the more abundant half of the community, whereas among the 24 detected only by Myloasm, just one was highly abundant. This pattern suggests that Myloasm-based contigs perform better for low-abundance strains, while metaFlye contigs remain more reliable for highly abundant ones.

Another observation from the sim_high dataset is that, for several highly abundant strains, most benchmarking tools reported substantially lower read-count abundances than the ground truth. For example, reads originating from NZ_CP012672.1 were predominantly assigned to NZ_CP102233.1, a nearly identical genome (ANI = 99.998) annotated under a different species (Sorangium cellulosum vs. Sorangium sp. So ce836). Because clustering was performed only among strains within the same species, this near-duplicate across species boundaries could not be resolved even after clustering.

As shown in Figure 4, all tools achieved higher F1 scores on ONT R10 and PacBio HiFi datasets compared to ONT R9, reflecting the higher base accuracy of these sequencing platforms. Interestingly, for the ONT R10 dataset, MADRe using Myloasm performed slightly worse than the metaFlye version, whereas for PacBio HiFi reads, Myloasm yielded marginally better results than the metaMDBG-based variant.

Taken together, these results, including the analyses of BC distances, demonstrate that MADRe, in all assembler configurations, consistently outperforms the other evaluated tools on the large sized simulated datasets.

Classification of Zymo mock communities datasets

To evaluate MADRe on real sequencing data, we conducted experiments on three different Zymo mock community datasets: ONT Zymo D6322, which consists of eight organisms (seven bacterial species and one fungus), and both the ONT and HiFi versions of Zymo D6331, which contain 21 organisms, including two fungi and five different strains of *Escherichia coli*. The primary challenge in the Zymo D6331 dataset is the ability to distinguish between these closely related *E. coli* strains. In this analysis, we excluded fungal genomes, focusing solely on bacterial classifications.

For Zymo mock communities, exact reference genomes of the strains present in the sample are available, along with their theoretical relative abundances provided by ZymoBIOMICS [60]. We supplemented our database with the Zymo reference genomes, assigning them separate labels. However, we did not use provided theoretical abundances in our analysis, as they may deviate from the expected values due to variations in library preparation [8, 30]. Instead, we established ground-truth read classifications. We mapped all reads to the expected bacterial reference genomes using Minimap2 and assigned true labels based on the best hit. These assignments were also used to determine the relative abundances. However, this process was not straightforward for the five *Escherichia coli* strains, as their high similarity led to ambiguous mappings. To address this, we leveraged our clustering method (explained in *Similar strains clustering* section), which grouped these five strains into three clusters. Specifically, strains B766 and B3008 each formed separate clusters, while the remaining three strains were grouped into a single cluster, indicating that they were too similar to be reliably distinguished at the strain level. This clustering result aligns with previous findings from metagenome assembly procedures [61], where B766 and B3008 were successfully assembled, while the other three strains were not.

Benchmarking with Centrifuger and Kraken2 was not performed for this experiment, as their database construction procedures do not support the inclusion of references with custom labels, which is essential for this evaluation.

Using this information, we incorporated the clustering results into our ground-truth labeling: reads originating from the same cluster were assigned the same label, ensuring a more accurate classification.

To evaluate performance, we calculated the BC distances (eq.9) between the observed read count abundances and the ground-truth abundances, both with and without post-clustering.

Figure 5 depicts radar plots showing the BC distances for the zymo D6322 ONT, zymo D6331 ONT, and zymo D6331 HiFi datasets. Dotted lines indicate BC distances computed using only true positive classifications based on the ground truth. In the first plot, which reports results for the D6322 dataset, MADRe clearly outperforms all other tools. The second and third plots display BC distances for the D6331 ONT and HiFi datasets, respectively. For the ONT dataset, when considering all classified reads, MADRe achieves the lowest BC distance. When focusing only on true positives, MADRe and MADRe_RC show comparable performance, indicating that the majority of reads classified by these tools are correctly assigned. In contrast, MORA exhibits a notably higher BC distance when evaluated only on true positives, suggesting less precise classification. For the HiFi dataset, overall distances for all the tools are significantly lower. Both AugPatho modes achieve slightly lower BC distances compared to MADRe. In Supplementary Figure S6, we present the corresponding results obtained after post-classification clustering of similar strains. Interestingly, for the ONT datasets, BC distances increased for both AugPatho and MADRe following clustering. Although the increase is not substantial, the clustering step led to elevated abundance estimates, resulting in a higher number of both false positives and true positives. This trend was not observed for the HiFi dataset, where MADRe achieved the best performance after clustering.

The exact read counts, used to calculate BC distance, are listed in the Supplementary Tables (ST17-ST19).

Table 2 presents the number of false-positive species and strain identifications. MADRe reports a significantly lower number of false positives at both levels compared to other tools. Supplementary Table ST20 provides a more detailed breakdown of the number of identifications. From this table, it is evident that MADRe's main limitation is the higher number of false negatives, primarily originating from low-abundance organisms that could not be detected using the assembly-based approach on which MADRe relies. This is further supported by the MADRe_RC results, where the number of false negatives is comparable to other tools. Nevertheless, MADRe consistently reports a substantially lower number of false positives. The table also includes AugPatho results from report outputs from both modes. These reports are generated after the final reassignment step and contain only abundance estimates. Consequently, they cannot be used directly for classification evaluation.

Table 2. False positive (FP) species and strains detected by different tools on Zymo datasets. An organism is considered a false positive if at least one read is classified under it, but it is not in the true community.

	Tool	D6331 ONT	D6331 HiFi	D6322 ONT
**FP Species**	MADRe	5	5	6
	MADRe_RC	391	53	385
	MORA	639	162	517
	AugPatho ID	266	10	327
	AugPatho REP	249	20	260
**FP Strains**	MADRe	386	114	52
	MADRe_RC	3441	1189	6441
	MORA	6251	4010	10357
	AugPatho ID	2641	518	6133
	AugPatho REP	2316	889	4675

While these reports show a significantly lower number of false positives, this reduction comes at the cost of a higher number of false negatives.

Classification of real anaerobic digester sludge metagenome

While Zymo mock communities represent real metagenomic data, they do not fully capture the complexity typically found in environmental or host-associated microbial communities. To better reflect realistic classification scenarios, we evaluated MADRe and the other competing tools on a real anaerobic digester sludge metagenome. As this dataset lacks ground truth, we focused on comparative analysis of classification outputs. All results presented here include post-clustering.

In this dataset, MADRe identified 1,320 reference strains (1,502 without clustering), while MADRe_RC reported 14,304 (19,067 without clustering), MORA 15,835 (23,516 without clustering), AugPatho ID 11,785 (16,488 without clustering), AugPatno REP 11,134 (14,604 without clustering) and Centrifuger 23,950 (28,450 without clustering). Out of 3,646,771 total reads, MADRe classified 575,052 (∼ 16%), MADRe_RC 696,961 (∼ 19%), MORA 696,839 (∼ 19%), AugPatho ID 898,906 (∼ 25%), AugPatho REP 737,714 (∼ 20%) and Centrifuger 1,350,537 (∼ 37%) reads.

Figure 6 illustrates percentile-normalized rank-abundance curves, highlighting differences in strain-level classification across the tools. The underlying read count abundance data used to generate this figure is provided in Supplementary Table ST21.

The curve for MADRe displays a consistent, moderately steep gradient throughout, with notable deviations at the beginning and end. The sharp rise at the beginning indicates the presence of a highly abundant strain, significantly more dominant than the others. This can be seen for the other tools as well. Toward the end, the curve drops sharply, likely reflecting false positives or low-confidence strain assignments. Compared to the other tools, MADRe shows a smoother and more gradual decline in the abundances of lower-ranked strains. In contrast, MADRe_RC, MORA, AugPatho and Centrifuger report a larger number of low-abundance strains, resulting in a more stepwise decline. The flat tail in their curves suggests that many strains are assigned near-zero abundances.

Figure 7 shows the relative abundances of the 20 most abundant strains reported by each tool, calculated relative to the total number of classified reads. We also generated an analogous visualization at the species level (Supplementary Figure S7), which additionally includes Kraken2 results. Among all tools, MADRe achieved the highest cumulative relative abundance for the top 20 strains, followed by AugPatho and MADRe_RC, while MORA and Centrifuger exhibited similar but substantially lower overall contributions from their top strains. Figure 7 highlights one notable strain-level discrepancy: the strain Paludibacter propionicigenes (accession number NC_022549.1, taxID 6135), which appeared among the top 20 only in AugPatho results. To investigate this discrepancy, we examined how reads classified as taxID 6135 by AugPatho were assigned by other tools. We found that most of these reads were classified as taxID 2148 or 264636 by the other approaches. As all three of these strains belong to the *Acholeplasmataceae* family, this pattern suggests the presence of shared genomic regions and potentially an unrepresented or novel genus within this family. To further examine this case, we mapped the relevant reads to all three references and found that none yielded strong, confident alignments, indicating that the true source strain is likely missing from the reference database. We then assembled the corresponding reads into contigs and classified them using Kraken2 against the full database. In 17 contigs classified under the expected family, the highest number of k-mers matched strain 2148, although the counts were low, again supporting the hypothesis of a missing true reference. Interestingly, strain 6135 is longer than both 2148 and 264636, and prior work on MORA has shown that AugPatho's scoring tends to favor longer, more complete genomes, which likely explains its preference for strain 6135 in this case.

Time and Memory Resources

Figure 8 presents the runtime and peak memory usage of the benchmarking tools on the ZymoD6331 ONT dataset which contained ∼1.7M reads.

Since majority of the tools, except Kraken2 and Centrifuger, rely on Minimap2 for read mapping, we categorized peak memory usage into components: memory used by Minimap2 and memory used by other operations. Similarly, CPU time was divided into time spent by Minimap2 and time spent on all other processing steps.

In the case of MADRe_RC, MORA, and AugPatho, the "other operations" category solely consists of the read reassignment algorithm. In contrast, for MADRe it includes assembly, HairSplitter, database reduction, and read reassignment. The role of Minimap2 also differs across MADRe and other tools. In MADRe_RC, MORA, and AugPatho, it is used for mapping reads to the large reference database, whereas in MADRe it is used both to map contigs to the large database and reads to the reduced one.

For HiFi reads, Minimap2 uses different parameters, and the MADRe pipeline employs metaMDBG instead of metaFlye for assembly. To account for these differences, Supplementary Table ST22 reports the same performance metrics for HiFi data.

When the dataset size increases, the situation changes. To illustrate this, we included runtime and memory usage results for the large simulated dataset sim_high (containing ∼5M reads) in Supplementary Table ST22. In this case, the peak RSS for MADRe is substantially higher (exceeding 200 GB), primarily due to the assembly process, while the peak RSS for Minimap2 during read mapping to the large database remains unchanged. However, mapping reads to such a large database requires the "−split-prefix" parameter in Minimap2, which generates temporary alignment files that are later merged at the end of the process. For this particular dataset, that procedure consumes approximately 1.2 TB of disk space, whereas the complete MADRe pipeline requires 160 GB (excluding database and read file sizes in both cases). Moreover, the entire MADRe pipeline is approximately 3.2x faster than the combination of Minimap2 with MORA or AugPatho. In contrast, Kraken2 and Centrifuge are substantially faster than mapping-based approaches and require considerably less disk space.

Discussion

In this work, we introduced MADRe, a metagenomic classification pipeline based on assembly-driven database reduction followed by read classification through mapping and reassignment. This approach enables accurate strain-level classification from large, multi-species databases without requiring prior knowledge of sample composition.

The first phase of MADRe reduces the reference database by identifying candidate strains likely to be present in the sample. Using assembly and an expectation–maximization (EM) soft clustering algorithm, this step aims to retain only the relevant references while eliminating unrelated ones. To take advantage of the longer contigs produced by standard assemblers, we avoided using strain-aware metagenome assemblers such as Strainberry [62], MetaBooster [63], HyLight [63], Strainy [64], and HairSplitter [49], which are known to yield shorter contigs. Instead, we used HairSplitter's functionality to estimate the number of collapsed strains for each contig and integrated this information with the mapping data of the initially strain-collapsed contigs. Our evaluation demonstrates that MADRe achieves effective database reduction while maintaining high recall.

Most existing strain-level classifiers either require single-species input or do not scale to large reference databases. Tools such as Kraken2, Sylph, or Centrifuger perform well at the species level and can handle large databases, making them valuable for pre-classification in strain-level workflows. However, such approaches generally require additional database preparation steps that are computationally intensive and impractical for complex metagenomic samples.

Mapping-based methods such as MORA and AugPatho represent another way to perform strain-level analysis on large databases. Nevertheless, our experiments showed that although MADRe incorporates an assembly step, typically considered both memory- and time-intensive, it required less memory than mapping raw reads directly to a large database, and even less than running Kraken2 on the same reference, when applied to a dataset of approximately 1.6 million ONT reads. For larger datasets exceeding 5 million reads, the assembly process becomes more memory demanding. However, MADRe remains substantially faster and requires considerably less disk space than mapping-based approaches. While the runtime and resource usage of Minimap2 could be reduced by using a smaller reference database, this would again require prior knowledge of the sample composition or risk omitting relevant strains. Among the evaluated strain-aware tools, MADRe is the fastest, providing an effective balance between computational efficiency and classification accuracy, and is thus well suited for scalable strain-level metagenomic analyses.

Our benchmarking analysis compared MADRe to MADRe_RC, MORA, the two AugPatho modes (PathoID and PathoREP), and, in several cases, to Centrifuger and Kraken2. For AugPatho, we used the updated SAM files generated during its reassignment step, in which individual reads in some cases can be associated with multiple references. This format may improve the detection of expected references but can also introduce ambiguity, potentially contributing to higher false-positive rates. On the new simulated datasets, MADRe achieved up to a 28% improvement over other state-of-the-art methods when no clustering of similar strains was applied, and up to a 10% improvement when clustering was used. A similar trend was observed for the more complex large sized simulated datasets. Although MADRe occasionally missed low-abundance strains in these datasets, it still produced more accurate classifications than competing tools. This is particularly important since MADRe focuses on precise read-level classification rather than on abundance estimation.

A major challenge in metagenomic evaluation is the scarcity of realistic benchmark datasets, which can lead to parameter overfitting across methods, often to well-known datasets such as the Zymo communities. This may explain observations like those in the D6331 ONT dataset, where MORA and AugPatho showed substantial discrepancies between BC distances calculated from all classified reads and those derived only from true positives - the distances for true positives were notably higher. In contrast, MADRe consistently achieved better results than other tools for both evaluation types, demonstrating robust classification performance.

One limitation of MADRe observed in the Zymo benchmarks is the higher number of false-negative identifications, largely stemming from low-abundance organisms that are difficult to capture through the assembly-based approach. However, a similar effect can be seen in AugPatho's final reports, which include only abundance estimates from the last reassignment step - these also exhibit increased false negatives. This highlights a broader issue in metagenomic classification: setting thresholds for reporting low-abundance taxa inevitably trades off between reducing false positives and increasing false negatives [65]. The identification and quantification of low-abundance organisms remain challenging problems. MADRe does not apply any automatic post-filtering, leaving the decision of whether to perform additional filtering or manual investigation of low-abundance taxa to the user.

A closer look at the composition of the Zymo datasets and the definitions of ground-truth labels provides additional insight into the observed differences in tool performance. We can clearly observe performance variation across the three Zymo datasets, which can be attributed to both the sequencing technology and the evaluation methodology. As expected, the D6331 HiFi dataset yielded the best results, reflecting the higher base-level accuracy of HiFi reads compared to ONT. At first glance, it may seem surprising that performance on D6322 ONT was lower than on D6331 ONT, since D6322 contains species from different genera and should, in principle, be easier to classify. The main factor explaining this discrepancy lies in how ground-truth labels were defined. For D6322, the evaluation was straightforward - each genome represented a distinct species, and thus, an exact species-level match was required for a correct classification. In contrast, D6331 includes five E. coli genomes, three of which have very high sequence identity (greater than 99.3% ANI score - calculated using fastANI). When constructing the ground truth for D6331, we clustered these three genomes and considered a read originating from any of them as correctly classified if it was assigned to any genome within that cluster. This less stringent criterion results in higher apparent performance for D6331 compared to D6322, an effect that applies uniformly across all evaluated tools.

In the real metagenomic dataset, MADRe classified fewer strains and focused on a confident subset of dominant organisms. In contrast, MADRe_RC, MORA, AugPatho, and Centrifuger reported a much larger number of low-abundance strains. While this may suggest higher sensitivity, many of these additional detections are likely spurious strain-level assignments, particularly in cases where the data do not support precise strain resolution. In this dataset, certain true references were absent from the database. Under these conditions, AugPatho tended to favor longer, highly similar genomes, MORA and MADRe_RC dispersed reads across multiple low-abundance strains, whereas MADRe mostly assigned reads to the reference sharing the greatest number of similar regions with the true organism.

This behavior was further investigated through a controlled experiment on a simulated dataset containing highly similar strains, described in detail in the Supplementary File (Similar Strains Experiment). This experiment demonstrated that mapping-based tools exhibit characteristic "attractor" behavior, often failing to proportionally distribute reads among near-identical strains. AugPatho and MORA, which rely on probabilistic or abundance-constrained models, frequently collapsed or misallocated reads to different representatives when the database or dataset composition changed. In contrast, MADRe consistently assigned reads to the most similar available reference, the centroid, thereby maintaining stable classifications. When centroid references were removed, MADRe dynam-

ically adapted by reallocating reads to the closest representative. These results highlight that MADRe's centroid-based strategy ensures stable and interpretable performance in challenging scenarios where ambiguity among nearly identical genomes is unavoidable.

These observations also emphasize a broader limitation of current long-read metagenomic classifiers: all existing methods struggle to resolve strains at extremely low sequence divergence. For this reason, in our evaluation we additionally report results at the cluster level, where highly similar genomes are grouped together based on their mapping profiles. This approach avoids penalizing tools for inevitable redistribution within such groups and provides a more biologically meaningful measure of performance. Unlike conventional clustering by average nucleotide identity (ANI), our method groups references according to shared mapping profiles, focusing on patterns reflected in the data rather than static reference similarity. This design supports the concept of sample-aware reference groupings that better capture functional and ecological relationships and could enhance classification accuracy in the presence of closely related organisms. Such clustering could also guide adaptive reference construction or real-time database refinement as additional samples are analyzed. Although clustering was used only for evaluation in this study and applied uniformly across all tools, future work will include deeper investigation of this method and its integration into the full MADRe pipeline.

MADRe is a modular pipeline composed of independent components, allowing easy adaptation to different tools, such as alternative assemblers. In the large sized simulated read experiments, we evaluated a MADRe version that used the Myloasm assembler instead of metaFlye and metaMDBG. The overall results were comparable. However, Myloasm showed better detection of low-abundance strains, whereas metaFlye performed slightly better for highly abundant ones. These findings indicate that different assemblers may be advantageous for different use cases. Consequently, we included Myloasm as an optional component within the MADRe pipeline, and future versions will support additional assemblers and related tools.

Beyond strain-level classification, MADRe's modular design, particularly its database reduction and probabilistic reassignment components, offers potential for broader applications. These include contig binning, assembly refinement, and functional gene profiling, where confident reference reduction and ambiguity-aware read handling are equally valuable.

Conclusion

In this study, we introduced MADRe, a novel pipeline for strain-level metagenomic classification of long-read sequencing data. MADRe combines long-read assembly, EM-based contig-to-reference mapping reassignment for database reduction, and probabilistic read reassignment to deliver accurate and efficient classification, even without prior knowledge of sample composition. Unlike many existing tools, MADRe is designed to operate with large, diverse databases spanning multiple taxonomic levels, enabling high-resolution classification while minimizing false positives.

The pipeline consists of two distinct steps: database reduction and read classification, both of which can be executed independently. If general insight into the strains present in a sample is required, the first step can be used alone. Conversely, when prior knowledge about the sample exists, or when a reduced reference set is already available, the read classification step can be applied independently. MADRe provides a practical, scalable, and modular solution for strain-level classification in complex microbial communities.

Methods

MADRe Database Reduction

The database reduction step, shown in Figure 1 and illustrated in more detail in Supplementary Figure S1, consists of two main phases: input file preparation and the database reduction.

In the input file preparation phase, raw long metagenomic reads are first assembled using metaFlye for ONT reads or metaMDBG for HiFi reads. When multiple strains of the same species are present in a sample, the assembly process can lead to strain collapse, producing contigs that represent a blend of closely related strains rather than distinct strain-specific sequences. Instead of using strain-aware metagenome assemblers, which typically generate shorter contigs, we chose to retain the longer contigs and infer strain-level complexity using HairSplitter functionality which estimates the number of collapsed strains per contig.

Assembled contigs are mapped to the reference database using Minimap2 with the *asm5* parameter preset, generating a PAF file as output. We chose this preset because, compared to *asm10* and *asm20*, it provides higher sensitivity, which is crucial for capturing more accurate and complete alignments of contigs to highly similar reference genomes. The MADRe database reduction process takes two key inputs: the estimated number of collapsed strains per contig determined by HairSplitter and the contig-to-reference mappings from Minimap2.

The database reduction process is based on the EM algorithm, which reassigns contigs to different references while performing soft clustering, allowing a single contig to be assigned to multiple references with different probabilities. The EM algorithm is widely used for handling ambiguous mappings in metagenomic classification [31, 29, 28, 30, 66, 43]. The implementation of the EM algorithm in MADRe is inspired by PathoScope2 [29] and EMU [30].

The database reduction process consists of three main steps. We can define a set of contigs as $C = \{c_1, c_2, \ldots, c_x\}$, where x is the number of contigs in the assembly. The set of references to which at least one contig is mapped is defined as $R = \{r_1, r_2, \ldots, r_g\}$, where g is the number of references. Additionally, let M represent the set of all of the mappings. In the first step, we compute a mapping score H for each mapping in the PAF file using the equation:

$$\mathrm{H} = 2 * \frac{\mathrm{N} \times ml}{\mathrm{N} + ml} \tag{1}$$

which represents the harmonic mean between the exact number of matches N and the mapping length *ml*. The *ml* is defined as the maximum value between the query mapping length and the reference mapping length. Applying the harmonic mean allows us to emphasize the smaller value, ensuring that a mapping does not receive an inflated score due, for example, to a very long but low-quality alignment.

The summarized mapping value S is then calculated for each contig-reference pair using:

$$\mathrm{S}(c_i, r_j) = \sum_{m \in M(c_i, r_j)} \mathrm{H}(p) \tag{2}$$

where $M(c_i, r_j)$ represents the set of mappings of contig c_i to reference r_j. This ensures that $\mathrm{S}(c_i, r_j)$ is computed by summing the mapping values of all instances where c_i maps to r_j, thus capturing all possible alignments between the contig and the reference. Following this, we divided mappings into *unique* and *non-unique*. Unique mappings occur when a contig maps exclusively to a single reference, whereas non-unique mappings represent ambiguous cases that require further resolution.

In the second step, non-unique mappings are processed using the EM algorithm, which iteratively refines contig assignments

based on mapping probabilities. The E-step updates the expected assignments of contigs, while the M-step re-estimates the parameters using the newly computed assignment probabilities from the previous iteration.

The probability of selecting a reference r_i is given by:

$$P(r_i) = \frac{1}{G}, \quad \text{where} \quad G = |R| \tag{3}$$

The conditional probability of c_i given r_i is expressed as:

$$P(c_i|r_i) = \frac{S(c_i, r_i)}{\max_{r_j \in R} S(c_i, r_j)} \tag{4}$$

The log-likelihood function $L(X)$ is given by:

$$L(C) = \sum_{i=1}^{X} \log \left(\sum_{j=1}^{G} P(c_i \mid r_j) \cdot P(r_j) \right) \tag{5}$$

The expectation step (E-step) updates the posterior probability $P(r_i \mid c_i)$ as follows:

$$P(r_i \mid c_i) = \frac{P(c_i \mid r_i) \cdot P(r_i)}{\sum_{j=1}^{G} P(c_i \mid r_j) \cdot P(r_j)} \tag{6}$$

The maximization step (M-step) updates the prior probability $P(r_i)$ as follows:

$$P(r_i) = \frac{\sum_{j=1}^{X} P(r_i \mid c_j)}{|M|} \tag{7}$$

The EM algorithm runs iteratively until it converges or reaches the maximum number of iterations set by the stopping criteria. Once the algorithm outputs posterior probabilities, these values are used to determine which references will be included in the reduced database.

Before selecting references, we first classify each contig at the species level. This is done by summing the posterior probabilities across all references belonging to a species and assigning the contig to the species with the highest total probability. After determining the species classification, we retain only posterior probabilities associated with references belonging to the selected species. Finally, for each contig, we select N + 2 reference genomes to include in the reduced database. The value of N is estimated based on the number of collapsed strains identified by HairSplitter. By default, MADRe adds two additional references to avoid excluding expected strains, although this offset can be adjusted through user parameters.

MADRe Read Classification

The read classification step in MADRe is designed to operate both with and without prior database reduction. The only requirement is a PAF file, the Minimap2 output containing read-to-database mappings, where each database sequence includes the corresponding taxonomic identifier. The MADRe read classification workflow is illustrated in Supplementary Figure S2.

The process begins by computing a mapping score for each alignment, defined as the ratio between the number of exact matches (N) and the mapping length (ml):

$$S = \frac{N}{ml}$$

Mappings are then categorized into unique and non-unique. Since a read can have multiple alignments, only the alignment with the highest score is retained for each read-reference pair. If a read has a single best-scoring mapping, it is classified as a unique mapping. Conversely, if multiple mappings share the same highest score, they are considered non-unique, and the read will go through a reassignment process. Formally:

$$\text{Unique if } S_{r,i} = \max_j(S_{r,j}) \text{ and this maximum is unique;}$$

$$\text{Non-unique if } S_{r,i} = \max_j(S_{r,j}) \text{ for two or more } j.$$

Before reassigning non-unique mappings, reads are first classified at the species level. For each read r, the maximum mapping score among all references belonging to a species s is computed as:

$$S_{r,s}^{\max} = \max_{i \in s}(S_{r,i})$$

The species with the highest $S_{r,s}^{\max}$ is selected as the species-level assignment for that read. Although the default assumption is that a read can be uniquely mapped to a single species but may map ambiguously to multiple strains within that species, this assumption does not always hold. In rare cases, two genomes from different species may share highly similar regions, making it difficult to determine the true origin of a read. In such situations, reads are randomly distributed between the corresponding species. These cases are uncommon and typically arise from taxonomic inconsistencies, for example when nearly identical strains according to taxonomy belong to different species.

To reassign non-unique mappings at the strain level, a species-specific clustering algorithm is applied. This algorithm evaluates the number of unique and non-unique mappings associated with each reference within the same species, identifying groups of references that share many mappings, indicating that they likely represent overlapping genomic regions and should form clusters.

The fundamental assumption is that a reference truly present in the sample should accumulate the highest number of mappings (both unique and high-confidence non-unique). Let M_i denote the total number of mappings to reference i:

$$M_i = U_i + N_i$$

where U_i and N_i represent the counts of unique and non-unique mappings, respectively. The expected references are identified as those with the highest M_i within each cluster, and all non-unique reads are reassigned to the most probable reference in that cluster:

$$r \in \text{cluster}(i) \Rightarrow r \rightarrow \arg \max_{j \in \text{cluster}(i)} M_j$$

This procedure ensures that ambiguous reads are redistributed toward references that are both well supported by unique evidence and consistent with the mapping structure observed across the sample. By reassigning reads to the most representative reference within each cluster, this methodology establishes MADRe's centroid-based behavior, maintaining stable and interpretable classifications even in the presence of highly similar strains.

Abundances calculation

At this stage, each read is assigned to a single reference genome. Based on these assignments, MADRe calculates the abundance of each detected strain. The primary abundance output file reports

the number of reads assigned to each reference. However, MADRe also provides an option to compute a length-normalized abundance, which accounts for both read and reference lengths. This alternative abundance metric is calculated as:

$$\text{Abundance}(r) = \frac{\sum_{i \in \text{Reads}_r} \textit{Length}(i)}{\text{Length}(r)} \quad \text{for } r \in R \tag{8}$$

where R is the set of the references and $Reads_r$ is set of the reads classified under reference r.

Similar strains clustering

The high similarity between closely related strains and the lack of a clear threshold for defining when two sequences represent the same strain, makes it difficult to ensure that a reference database contains only unique strain sequences [33]. Some entries may correspond to highly similar strains or even to multiple assemblies of the same strain. To address this, MADRe includes an optional reference clustering step within the read classification process, designed to group similar references based on shared read mappings.

This clustering step operates on the same mapping file used in the classification stage and produces two output files: one reporting the abundances of identified clusters and the other listing the representative reference for each cluster.

The clustering procedure is illustrated in Supplementary Figure S3. It begins by using the calculated mapping scores and species-level labels. For each species-specific subset, the algorithm identifies the highest-quality mapping for each read. A binary vector is constructed for each reference, where each bit indicates whether a given read strongly supports that reference. These binary vectors are then clustered using DBSCAN with precomputed Jaccard distances, eps = 0.9, and min_samples = 1. Cluster-level abundances are computed accordingly. As a result, the post-clustering abundance files report only the representative references for each cluster.

Evaluation details

Our evaluation is primarily focused on exact read-level taxonomic assignments and read count-based abundance estimates.

To ensure a fair comparison, all tools were benchmarked using the same large reference database. For tools requiring mapping files as input, namely MORA, AugPatho, and MADRe_RC, we used a unified set of read-to-reference alignments generated by Minimap2. All reads were mapped to the full database, and the resulting SAM file was used directly for MORA and AugPatho. This SAM file was subsequently converted to PAF format using Paftools, as required by MADRe_RC.

Simulated reads were generated with the Badread tool [53], applying the whole-metagenome simulation mode with default values for chimeric, junk, and random reads. The simulation was performed using the *nanopore2023* model, which corresponds to the ONT R10.4.1. The exact command used is provided in the Supplementary File.

In case of simulated datasets, ground truth was available for every read, including its corresponding strain-level taxID and reference accession. Using this information, we computed true positives (TP), false positives (FP), true negatives (TN), and false negatives (FN) by comparing the assigned strain-level taxIDs with the expected ones. For Kraken2, we extracted read IDs and assigned taxIDs from its output. If a read was assigned to a higher taxonomic level, even if it was taxonomic correct, we treated it as a false positive, as the evaluation strictly focused on strain-level classification.

For the simulated datasets and Zymo mock communities we calculated Bray-Curtis (BC) distance as:

$$\text{BC}(x, y) = \frac{\sum_{i=1}^{n} |x_i - y_i|}{\sum_{i=1}^{n} (x_i + y_i)} \tag{9}$$

Where $x = (x_1, x_2, \ldots, x_n)$ and $y = (y_1, y_2, \ldots, y_n)$ are the abundance vectors for two samples or profiles, n is the number of strains, x_i and y_i are the abundances of the ith strain in samples x and y, respectively. BC(x, y) is the Bray-Curtis dissimilarity or distance, ranging from 0 (identical composition) to 1 (completely disjoint).

In the case of medium-sized simulated datasets (sim_small, sim_medium and sim_expanded) and the real dataset, the evaluation was also performed at the species level (results presented in Supplementary Table ST8 and Supplementary Figure S7). All strain-level classifications were uplifted to their corresponding species, and read count abundances were calculated. For Kraken2, we used the species-level abundances reported in its summary file, limited to entries labeled with an "S" (species rank).

MADRe, MADRe_RC, and MORA each produce read-level classification files that associate each read with a reference genome. Since all tools shared the same mapping input, for AugPatho we ran PathoID and PathoREPORT steps, which output an updated SAM files and a report containing reference abundance estimates. In this updated SAM files, a single read can be associated with multiple references. For evaluation purposes, we allowed such multi-reference assignments, which may slightly benefit AugPatho by increasing the number of true positives, while also increasing the risk of false positives. These trade-offs are largely neutralized when clustering is applied, as similar strains typically end up grouped in the same cluster. With Centrifuger we encountered one limitation - Centrifuger cannot confidently assign a read to a specific reference sequence (e.g., when multiple chromosomes belong to the same strain), it often classifies the read under the NCBI strain-level taxid. In some cases, this strain taxid is identical to the species taxid, making it impossible to directly and fairly compare such classifications to those of other tools that operate at the sequence level. For benchmarking consistency, we therefore considered as true positives only the reads correctly classified under the expected reference sequence. It is important to note that this issue affected a relatively small fraction of reads (approximately 9000 out of 5 million reads in the 1000-genome dataset).

We used the same clustering across all tools to ensure consistency in cluster-based evaluation. Our clustering is based on read-to-reference mapping profiles, which can differ depending on the size and composition of the database. For example, when reads are mapped to a reduced database, the absence of certain similar references can make ambiguous mappings more resolvable. To avoid such inconsistencies, clustering was performed only once on the PAF file used for MADRe_RC, which contains read mappings to the complete reference database. During cluster-level evaluation, a classification was considered a true positive if the read was assigned to a reference that belongs to the same cluster as the ground truth reference:

$$\text{TP} = \begin{cases} 1, & \text{if } C(\hat{r}) = C(r_{\text{true}}) \\ 0, & \text{otherwise} \end{cases}$$

where $C(\hat{r})$ denotes the cluster of the predicted reference and $C(r_{\text{true}})$ denotes the cluster of the true reference. A classification is considered a true positive (TP) if both references belong to the same cluster.

All commands used to perform classification with the evaluated tools are listed in the Supplementary File.

Availability of source code and requirements

- Project name: MADRe - Metagenome Assembly driven Database Reduction
- Project home page: https://github.com/lbcb-sci/MADRe
- Operating system(s): UNIX
- Programming language: Python
- Other requirements: Environment Modules, Conda, Docker, see https://github.com/lbcb-sci/MADRe
- License: MIT

Data availability

The source code for MADRe is available at https://github.com/lbcb-sci/MADRe. Simulated data can be accessed via Zenodo. Zymo D6322 ONT dataset is obtained from BioProject PRJNA1240873, zymo D6331 ONT dataset is obtained from [55], and zymo D6331 PacBio HiFi dataset from [56].

Additional files

SupplementaryFile.pdf; SupplementaryTables.xlsx

Declarations

List of abbreviations

EM: expectation-maximization; TP: true positive; TN: true negative; FP: false positive; FN: false negative; BC: Bray-Curtis; ANI: average nucleotide identity; ONT: Oxford Nanopore Technologies; taxID: taxonomy identifier.

Competing Interests

M.Š. has been jointly funded by Oxford Nanopore Technologies and AI Singapore for the project AI-driven De Novo Diploid Assembler. The remaining authors declare no competing interests.

Funding

This work was supported by the Croatian Science Foundation under grants IP-2018-01-5886 (SIGMA) and MOBDOK-2023-2941, and by the Singapore Ministry of Health's National Medical Research Council, Singapore, under the grant MOH-000649-01 (Rapid diagnostic of infectious diseases based on nanopore sequencing and AI methods) – Individual Research Grant (NMRC/OFIRG/MOH-000649-00).

Acknowledgments

The authors thank Lune Angevin for testing the tool and providing valuable feedback.

Author's Contributions

K.K. and M.Š. conceived the study. J.L. designed and implemented the pipeline. K.K. supervised database reduction implementation. R.V. supervised read classification implementation. J.L. drafted the manuscript. K.K., R.V. and M.Š. revised the manuscript. All authors read and approved the manuscript.

References

1. Gilbert JA, Blaser MJ, Caporaso JG, Jansson JK, Lynch SV, Knight R. Current understanding of the human microbiome. Nature medicine 2018;24(4):392–400.
2. Ling LL, Schneider T, Peoples AJ, Spoering AL, Engels I, Conlon BP, et al. A new antibiotic kills pathogens without detectable resistance. Nature 2015;517(7535):455–459.
3. Lu J, Rincon N, Wood DE, Breitwieser FP, Pockrandt C, Langmead B, et al. Metagenome analysis using the Kraken software suite. Nature protocols 2022;17(12):2815–2839.
4. Zhang W, Liu Y, Li G, Xu J, Chen E, Schönhuth A, et al. Strain-level metagenomic profiling using pangenome graphs with PanTax. bioRxiv 2025;p. 2025–04.
5. Truong DT, Franzosa EA, Tickle TL, Scholz M, Weingart G, Pasolli E, et al. MetaPhlAn2 for enhanced metagenomic taxonomic profiling. Nature methods 2015;12(10):902–903.
6. Blanco-Míguez A, Beghini F, Cumbo F, McIver LJ, Thompson KN, Zolfo M, et al. Extending and improving metagenomic taxonomic profiling with uncharacterized species using MetaPhlAn 4. Nature Biotechnology 2023;41(11):1633–1644.
7. Ruscheweyh HJ, Milanese A, Paoli L, Sintsova A, Mende DR, Zeller G, et al. mOTUs: profiling taxonomic composition, transcriptional activity and strain populations of microbial communities. Current Protocols 2021;1(8):e218.
8. Chen X, Yin X, Shi X, Yan W, Yang Y, Liu L, et al. Melon: metagenomic long-read-based taxonomic identification and quantification using marker genes. Genome Biology 2024;25(1):226.
9. Costea PI, Munch R, Coelho LP, Paoli L, Sunagawa S, Bork P. metaSNV: a tool for metagenomic strain level analysis. PloS one 2017;12(7):e0182392.
10. Olm MR, Crits-Christoph A, Bouma-Gregson K, Firek BA, Morowitz MJ, Banfield JF. inStrain profiles population microdiversity from metagenomic data and sensitively detects shared microbial strains. Nature Biotechnology 2021;39(6):727–736.
11. Menzel P, Ng KL, Krogh A. Fast and sensitive taxonomic classification for metagenomics with Kaiju. Nature communications 2016;7(1):11257.
12. Buchfink B, Xie C, Huson DH. Fast and sensitive protein alignment using DIAMOND. Nature methods 2015;12(1):59–60.
13. Steinegger M, Söding J. MMseqs2 enables sensitive protein sequence searching for the analysis of massive data sets. Nature biotechnology 2017;35(11):1026–1028.
14. Huson DH, Albrecht B, Bağcı C, Bessarab I, Gorska A, Jolic D, et al. MEGAN-LR: new algorithms allow accurate binning and easy interactive exploration of metagenomic long reads and contigs. Biology direct 2018;13:1–17.
15. Marić J, Križanović K, Riondet S, Nagarajan N, Šikić M. Comparative analysis of metagenomic classifiers for long-read sequencing datasets. BMC bioinformatics 2024;25(1):15.
16. Wood DE, Lu J, Langmead B. Improved metagenomic analysis with Kraken 2. Genome biology 2019;20:1–13.
17. Breitwieser FP, Baker DN, Salzberg SL. KrakenUniq: confident and fast metagenomics classification using unique k-mer counts. Genome biology 2018;19:1–10.
18. Lu J, Breitwieser FP, Thielen P, Salzberg SL. Bracken: estimating species abundance in metagenomics data. PeerJ Computer Science 2017;3:e104.
19. Kim D, Song L, Breitwieser FP, Salzberg SL. Centrifuge: rapid and sensitive classification of metagenomic sequences. Genome research 2016;26(12):1721–1729.
20. Song L, Langmead B. Centrifuger: lossless compression of microbial genomes for efficient and accurate metagenomic sequence classification. Genome biology 2024;25(1):106.
21. Ounit R, Wanamaker S, Close TJ, Lonardi S. CLARK: fast and accurate classification of metagenomic and genomic sequences using discriminative k-mers. BMC genomics 2015;16:1–13.
22. Ounit R, Lonardi S. Higher classification sensitivity of

short metagenomic reads with CLARK-S. Bioinformatics 2016;32(24):3823–3825.

23. Piro VC, Dadi TH, Seiler E, Reinert K, Renard BY. ganon: precise metagenomics classification against large and up-to-date sets of reference sequences. Bioinformatics 2020;36(Supplement_1):i12–i20.
24. Piro VC, Reinert K. ganon2: up-to-date and scalable metagenomics analysis. bioRxiv 2023;p. 2023–12.
25. Ulrich JU, Renard BY. Fast and space-efficient taxonomic classification of long reads with hierarchical interleaved XOR filters. Genome Research 2024;34(6):914–924.
26. Shaw J, Yu YW. Metagenome profiling and containment estimation through abundance-corrected k-mer sketching with sylph. bioRxiv 2023;p. 2023–11.
27. Dilthey AT, Jain C, Koren S, Phillippy AM. Strain-level metagenomic assignment and compositional estimation for long reads with MetaMaps. Nature communications 2019;10(1):3066.
28. Hong C, Manimaran S, Shen Y, Perez-Rogers JF, Byrd AL, Castro-Nallar E, et al. PathoScope 2.0: a complete computational framework for strain identification in environmental or clinical sequencing samples. Microbiome 2014;2:1–15.
29. Francis OE, Bendall M, Manimaran S, Hong C, Clement NL, Castro-Nallar E, et al. Pathoscope: species identification and strain attribution with unassembled sequencing data. Genome research 2013;23(10):1721–1729.
30. Curry KD, Wang Q, Nute MG, Tyshaieva A, Reeves E, Soriano S, et al. Emu: species-level microbial community profiling of full-length 16S rRNA Oxford Nanopore sequencing data. Nature methods 2022;19(7):845–853.
31. Zheng A, Shaw J, Yu YW. Mora: abundance aware metagenomic read re-assignment for disentangling similar strains. BMC bioinformatics 2024;25(1):161.
32. Schaeffer L, Pimentel H, Bray N, Melsted P, Pachter L. Pseudoalignment for metagenomic read assignment. Bioinformatics 2017;33(14):2082–2088.
33. Van Rossum T, Ferretti P, Maistrenko OM, Bork P. Diversity within species: interpreting strains in microbiomes. Nature Reviews Microbiology 2020;18(9):491–506.
34. Luo C, Walk ST, Gordon DM, Feldgarden M, Tiedje JM, Konstantinidis KT. Genome sequencing of environmental Escherichia coli expands understanding of the ecology and speciation of the model bacterial species. Proceedings of the National Academy of Sciences 2011;108(17):7200–7205.
35. Kashtan N, Roggensack SE, Rodrigue S, Thompson JW, Biller SJ, Coe A, et al. Single-cell genomics reveals hundreds of coexisting subpopulations in wild Prochlorococcus. Science 2014;344(6182):416–420.
36. Schloissnig S, Arumugam M, Sunagawa S, Mitreva M, Tap J, Zhu A, et al. Genomic variation landscape of the human gut microbiome. Nature 2013;493(7430):45–50.
37. Yassour M, Jason E, Hogstrom LJ, Arthur TD, Tripathi S, Siljander H, et al. Strain-level analysis of mother-to-child bacterial transmission during the first few months of life. Cell host & microbe 2018;24(1):146–154.
38. van Dijk LR, Walker BJ, Straub TJ, Worby CJ, Grote A, Schreiber IV HL, et al. StrainGE: a toolkit to track and characterize low-abundance strains in complex microbial communities. Genome biology 2022;23(1):74.
39. Albanese D, Donati C. Strain profiling and epidemiology of bacterial species from metagenomic sequencing. Nature communications 2017;8(1):2260.
40. Roosaare M, Vaher M, Kaplinski L, Möls M, Andreson R, Lepamets M, et al. StrainSeeker: fast identification of bacterial strains from raw sequencing reads using user-provided guide trees. PeerJ 2017;5:e3353.
41. Siekaniec G, Roux E, Lemane T, Guédon E, Nicolas J. Identification of isolated or mixed strains from long reads: a challenge met on Streptococcus thermophilus using a MinION sequencer. Microbial genomics 2021;7(11):000654.
42. Dempster AP, Laird NM, Rubin DB. Maximum likelihood from incomplete data via the EM algorithm. Journal of the royal statistical society: series B (methodological) 1977;39(1):1–22.
43. Skoufos G, Almodaresi F, Zakeri M, Paulson JN, Patro R, Hatzigeorgiou AG, et al. AGAMEMNON: an Accurate metaGenomics And MEtatranscriptoMics quaNtificatiON analysis suite. Genome biology 2022;23(1):39.
44. Jain C, Rodriguez-R LM, Phillippy AM, Konstantinidis KT, Aluru S. High throughput ANI analysis of 90K prokaryotic genomes reveals clear species boundaries. Nature communications 2018;9(1):5114.
45. Koslicki D, White S, Ma C, Novikov A. YACHT: an ANI-based statistical test to detect microbial presence/absence in a metagenomic sample. Bioinformatics 2024;40(2):btae047.
46. Anyansi C, Straub TJ, Manson AL, Earl AM, Abeel T. Computational methods for strain-level microbial detection in colony and metagenome sequencing data. Frontiers in Microbiology 2020;11:1925.
47. LaPierre N, Alser M, Eskin E, Koslicki D, Mangul S. Metalign: efficient alignment-based metagenomic profiling via containment min hash. Genome biology 2020;21:1–15.
48. Koslicki D, Zabeti H. Improving minhash via the containment index with applications to metagenomic analysis. Applied Mathematics and Computation 2019;354:206–215.
49. Faure R, Lavenier D, Flot JF. HairSplitter: haplotype assembly from long, noisy reads. Peer Community Journal 2024;4.
50. Kolmogorov M, Bickhart DM, Behsaz B, Gurevich A, Rayko M, Shin SB, et al. metaFlye: scalable long-read metagenome assembly using repeat graphs. Nature methods 2020;17(11):1103–1110.
51. Benoit G, Raguideau S, James R, Phillippy AM, Chikhi R, Quince C. High-quality metagenome assembly from long accurate reads with metaMDBG. Nature Biotechnology 2024;42(9):1378–1383.
52. Shaw J, Marin MG, Li H. High-resolution metagenome assembly for modern long reads with myloasm. bioRxiv 2025;p. 2025–09.
53. Wick RR. Badread: simulation of error-prone long reads. Journal of Open Source Software 2019;4(36):1316.
54. Zhang W, Benchmarking datasets used in the manuscript "Strain-level metagenomic profiling using pangenome graphs with PanTax". Zenodo; 2025. https://zenodo.org/records/16885808, version v3; accessed 2025-10-19.
55. Liu L, Yang Y, Deng Y, Zhang T. Nanopore long-read-only metagenomics enables complete and high-quality genome reconstruction from mock and complex metagenomes. Microbiome 2022;10(1):209.
56. Portik DM, Brown CT, Pierce-Ward NT. Evaluation of taxonomic classification and profiling methods for long-read shotgun metagenomic sequencing datasets. BMC bioinformatics 2022;23(1):541.
57. Sereika M, Kirkegaard RH, Karst SM, Michaelsen TY, Sørensen EA, Wollenberg RD, et al. Oxford Nanopore R10. 4 long-read sequencing enables the generation of near-finished bacterial genomes from pure cultures and metagenomes without short-read or reference polishing. Nature methods 2022;19(7):823–826.
58. O'Leary NA, Wright MW, Brister JR, Ciufo S, Haddad D, McVeigh R, et al. Reference sequence (RefSeq) database at NCBI: current status, taxonomic expansion, and functional annotation. Nucleic acids research 2016;44(D1):D733–D745.
59. Bray JR, Curtis JT. An ordination of the upland forest communities of southern Wisconsin. Ecological monographs 1957;27(4):326–349.
60. Zymo Research, ZymoBIOMICS: Microbiomics Solutions; 2025. https://www.zymoresearch.com/pages/zymobiomics, accessed: 2025-03-14.

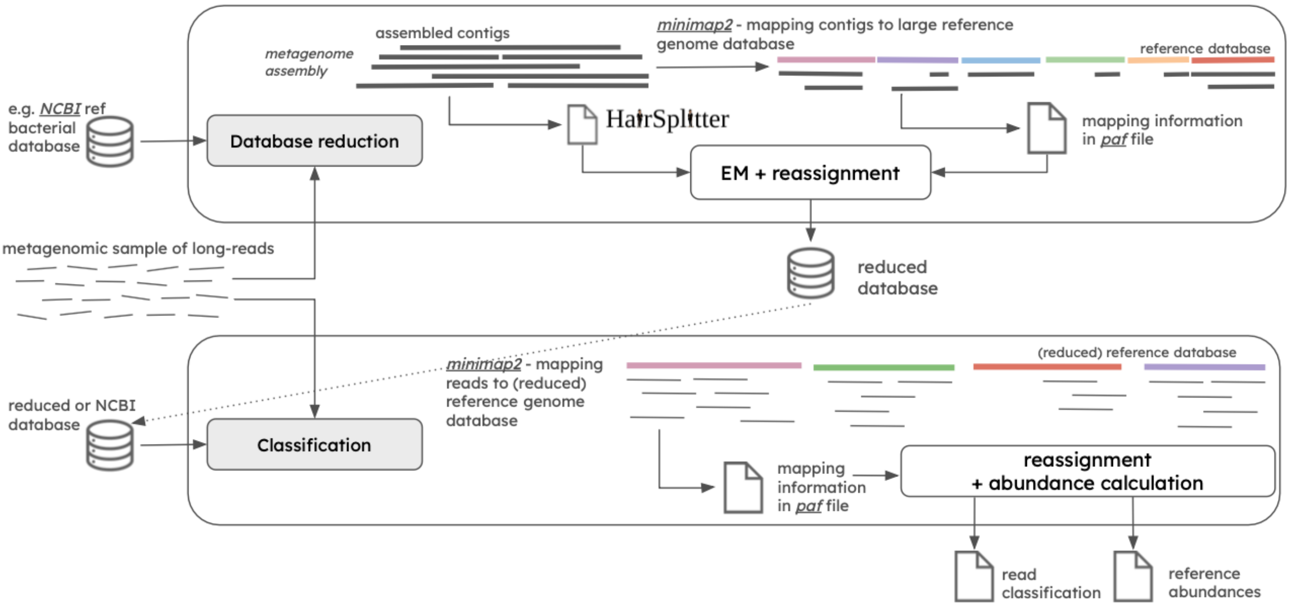

Figure 1. MADRe overall pipeline The first step of the pipeline performs database reduction using an EM-based contig-to-reference mapping procedure to identify organisms present in the sample. The second step involves read classification, which applies probabilistic read reassignment based on mapping information.

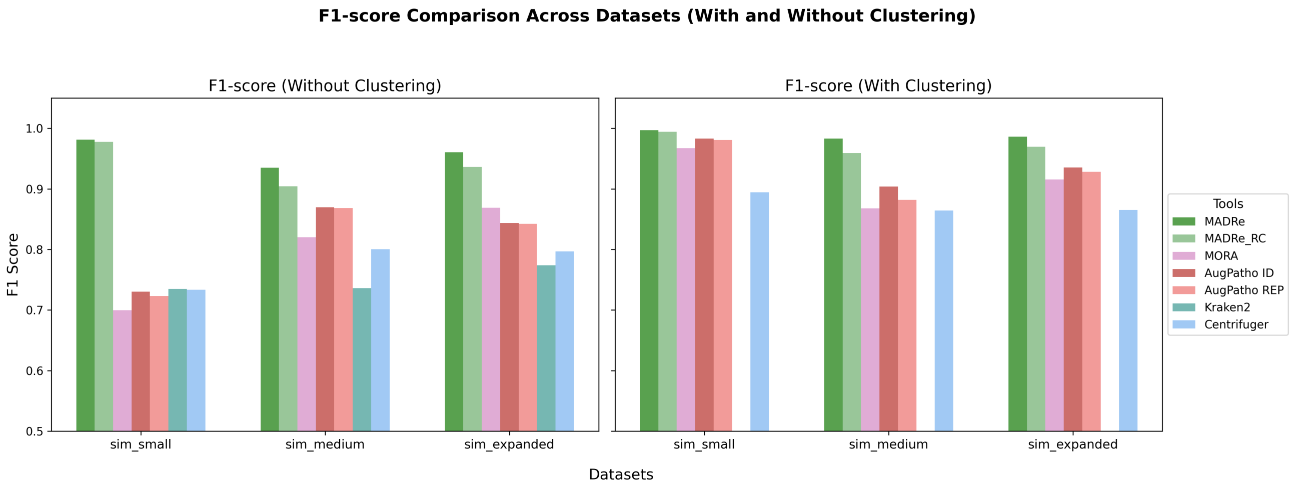

Figure 2. F1 scores of strain-level classification on medium-sized simulated reads, shown with and without post-clustering (grouping highly similar strains). Kraken2 results were omitted from the clustering analysis, as its output format does not support proper clustering.

61. Feng X, Cheng H, Portik D, Li H. Metagenome assembly of high-fidelity long reads with hifiasm-meta. Nature methods 2022;19(6):671–674.
62. Vicedomini R, Quince C, Darling AE, Chikhi R. Strainberry: automated strain separation in low-complexity metagenomes using long reads. Nature Communications 2021;12(1):4485.
63. Luo X, Kang X, Schönhuth A. Enhancing long-read-based strain-aware metagenome assembly. Frontiers in genetics 2022;13:868280.
64. Kazantseva E, Donmez A, Frolova M, Pop M, Kolmogorov M. Strainy: phasing and assembly of strain haplotypes from long-read metagenome sequencing. Nature Methods 2024;21(11):2034–2043.
65. Simon HY, Siddle KJ, Park DJ, Sabeti PC. Benchmarking metagenomics tools for taxonomic classification. Cell 2019;178(4):779–794.
66. Sapoval N, Liu Y, Curry KD, Kille B, Huang W, Kokroko N, et al. Lightweight taxonomic profiling of long-read metagenomic datasets with Lemur and Magnet. bioRxiv 2024;.

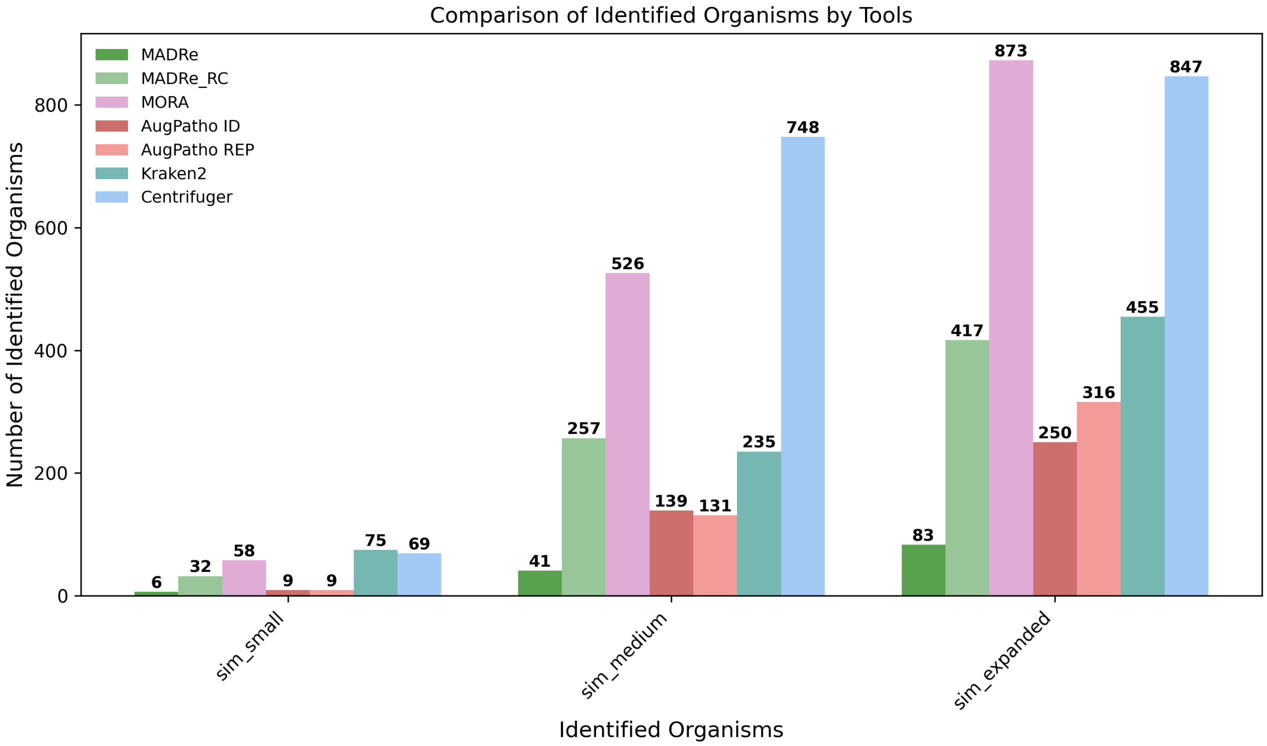

Figure 3. Number of identified organisms by different tools on simulated reads. An organism is considered identified if at least one read is classified under it. The sim_small dataset contains 4 strains, sim_medium contains 15 strains, and sim_expanded contains 30 strains. All tools successfully identified all expected strains, resulting in no false negatives.

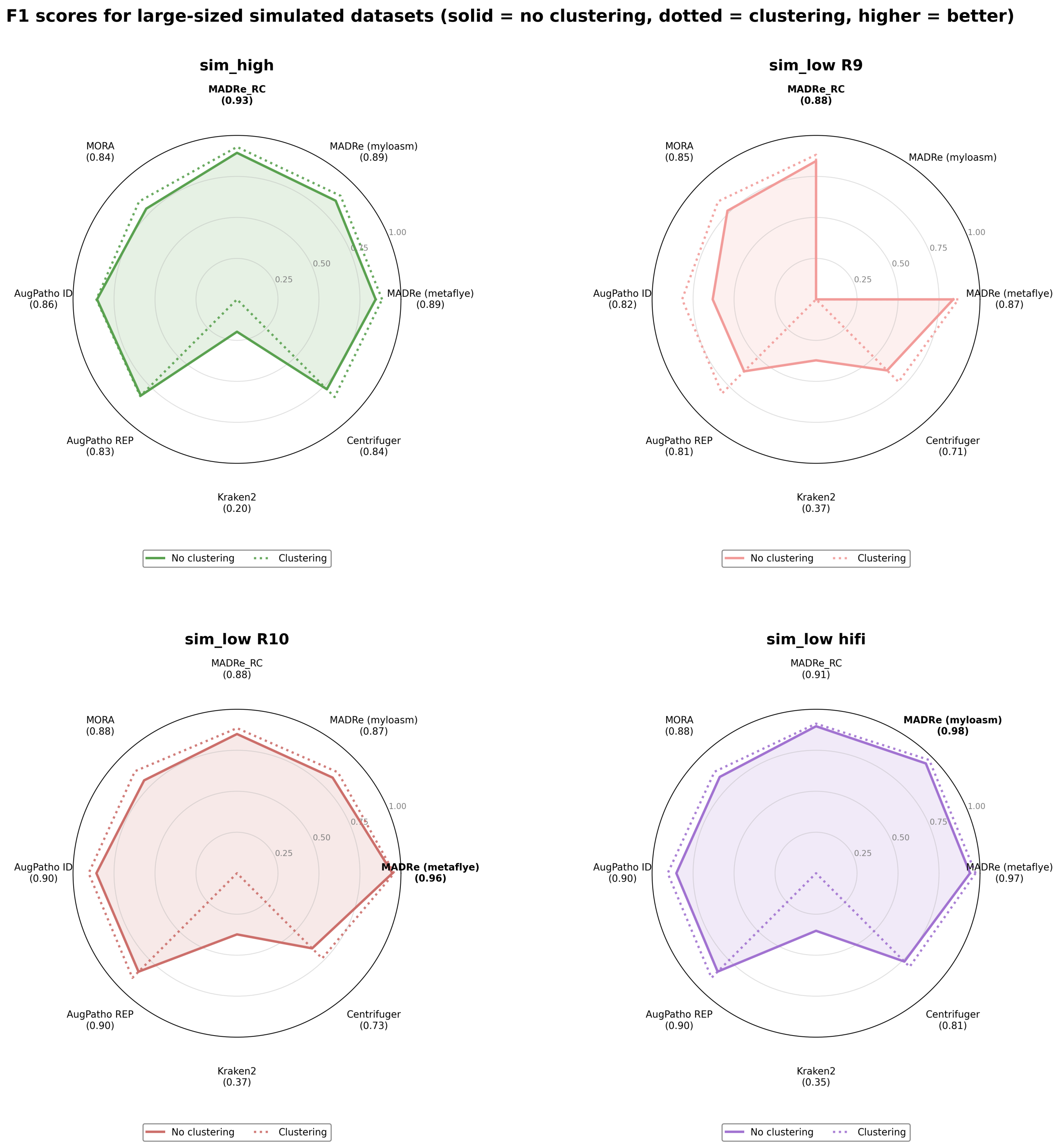

Figure 4. F1 scores for large-sized simulated datasets. Solid lines represent results without clustering, while dotted lines indicate results with clustering. Kraken2 clustering results are omitted, as its output format does not support clustering. Similarly, MADRe (Myloasm) results are excluded for ONT R9 data, since Myloasm is not designed for this type of sequencing data. In each plot, the best-performing tool is highlighted in bold, and the values in parentheses indicate the best performance achieved by each tool, with and without clustering.

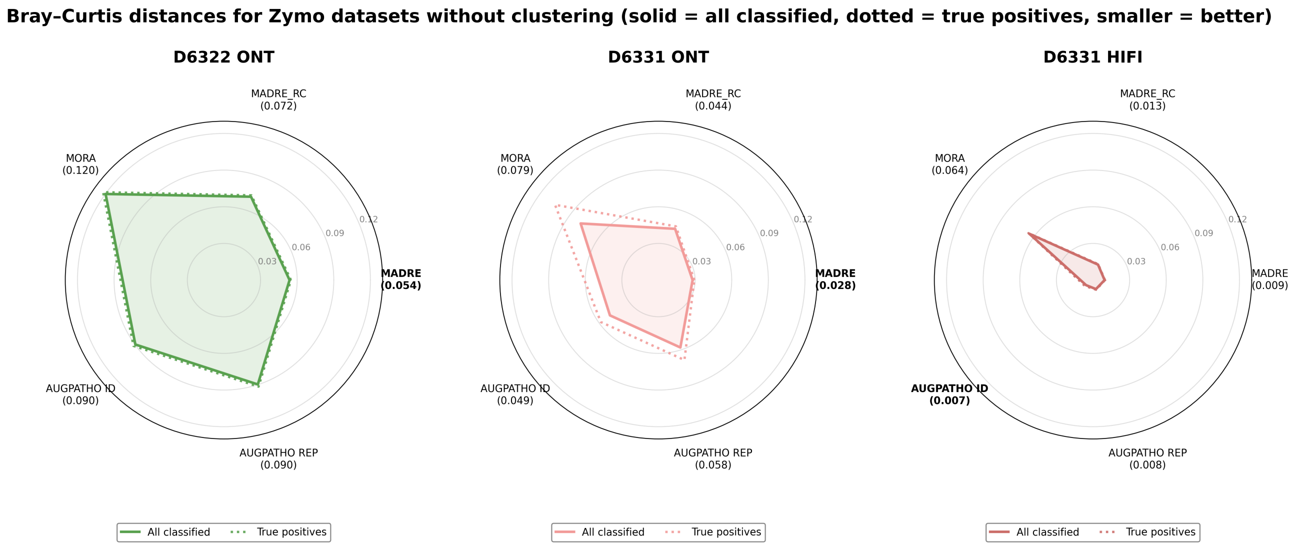

Figure 5. Bray–Curtis distances for Zymo datasets. The plots show changes in BC distance with and without post-clustering of similar strains. Solid lines represent distances based on all classified read counts, while dashed lines show distances calculated using only true positive (TP) read counts.

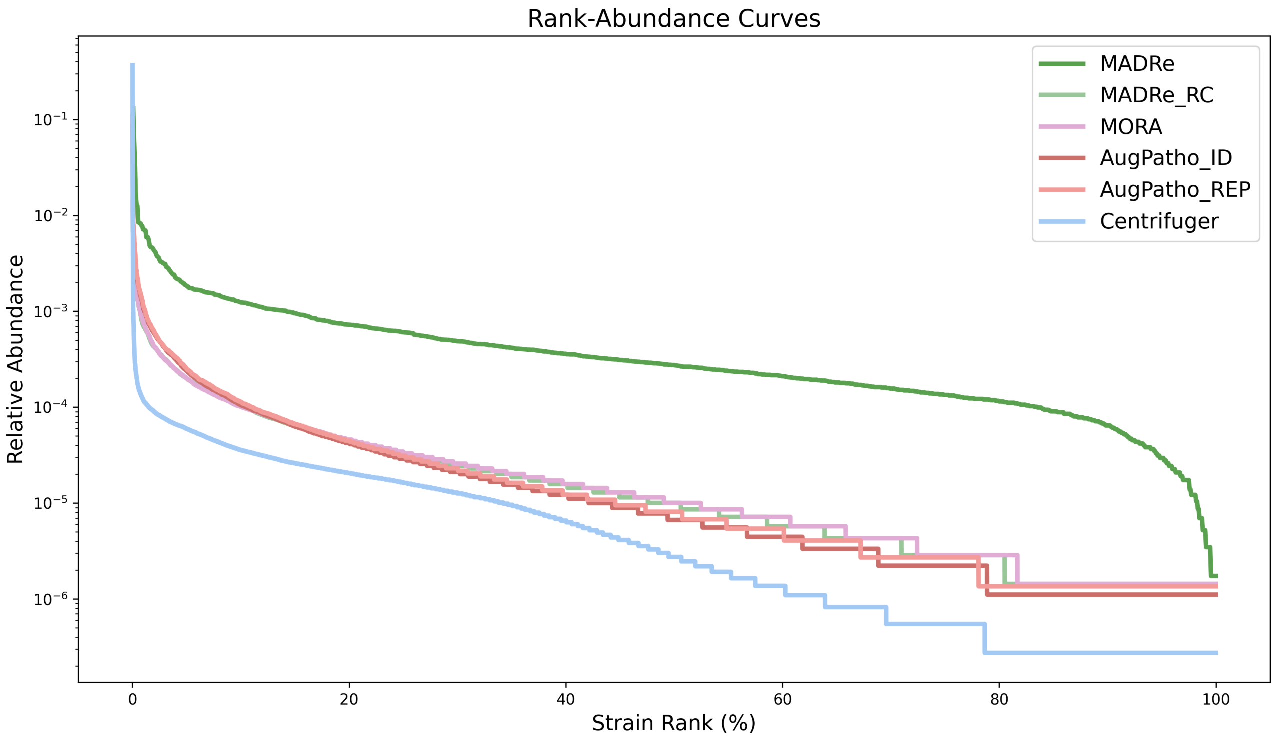

Figure 6. Percentile-normalized rank-abundance curves. The x-axis shows strain ranks expressed as percentiles, while the y-axis represents the relative abundance of each strain on a logarithmic scale.

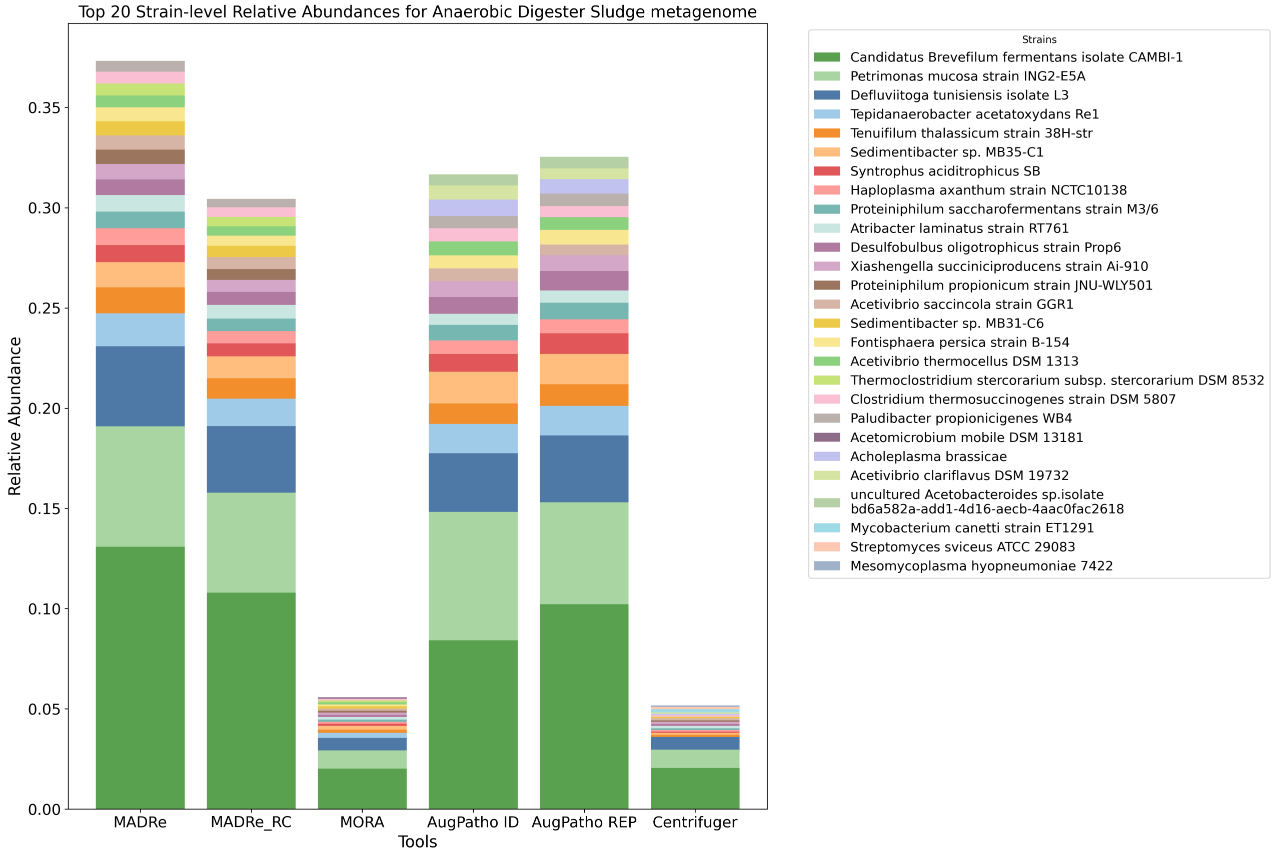

Figure 7. Real data strain-level abundances of the top 20 most abundant strains identified by each tool. Two strains are highlighted in red to illustrate cases where different tools classified reads originating from an unrepresented reference to distinct false positives that share similar genomic regions.

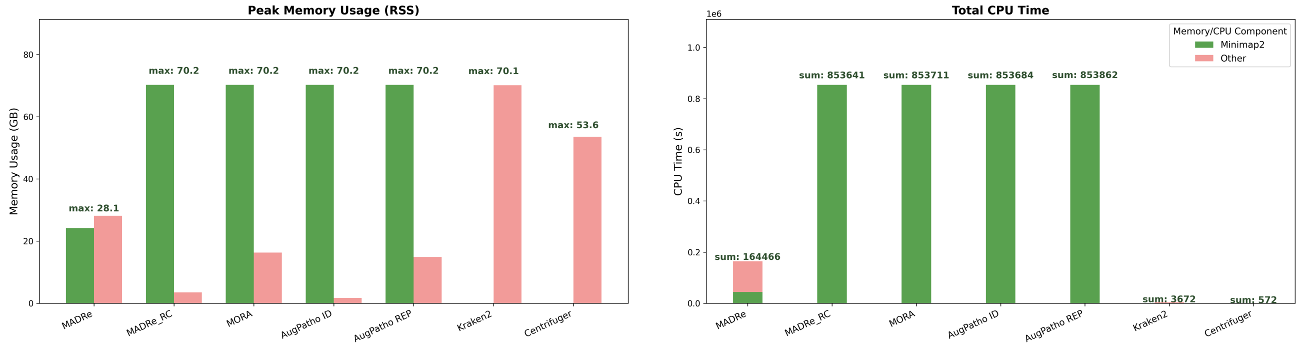

Figure 8. Memory (RSS peak in GB) and CPU time (in seconds) for different tools, split between Minimap2 mapping and other processing steps.

Figure 1

Click here to access/download;Figure;Fig1-MADRe_pipeline.pdf

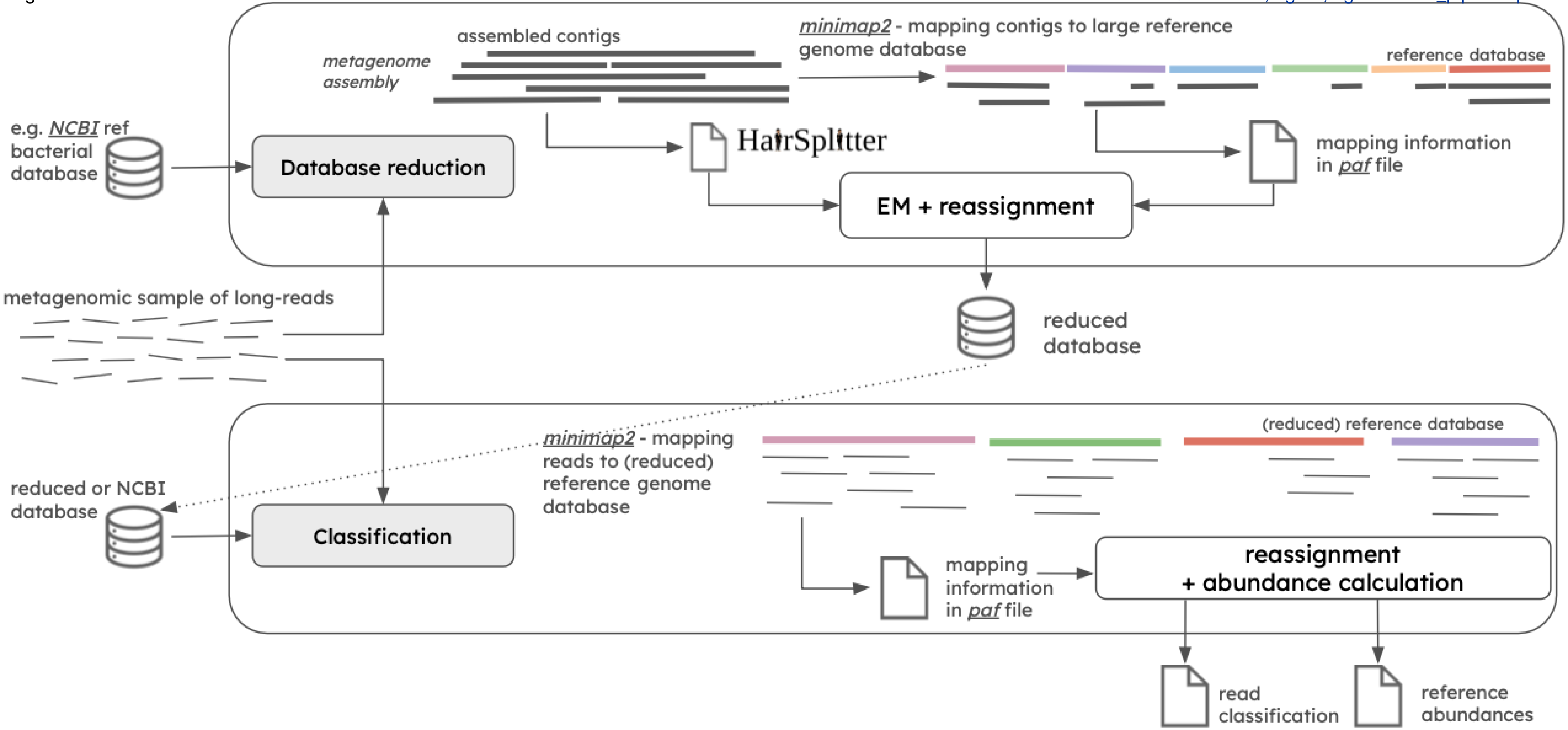

Figure 2

Click here to access/download;Figure;Fig2-F1_scores_medium_size_datasets.pdf

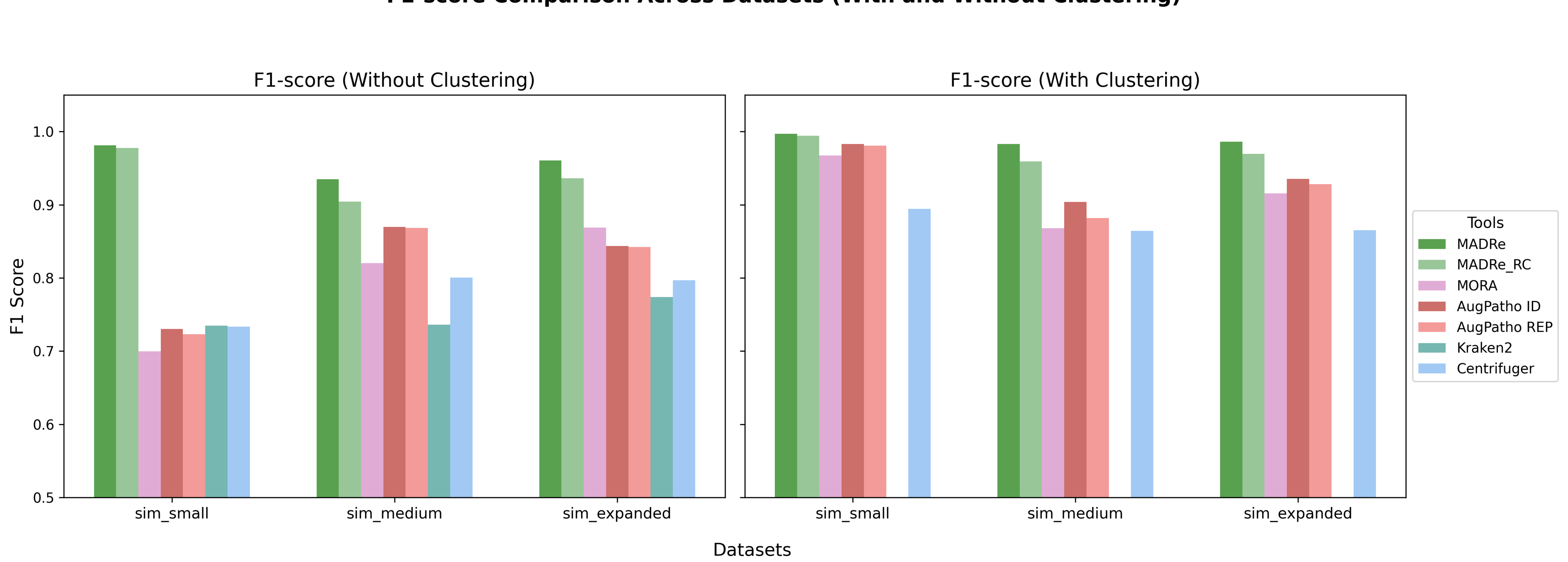

Figure 3

Click here to access/download;Figure;Fig3-Number_of_identified_organisms_medium_size_datasets.pdf

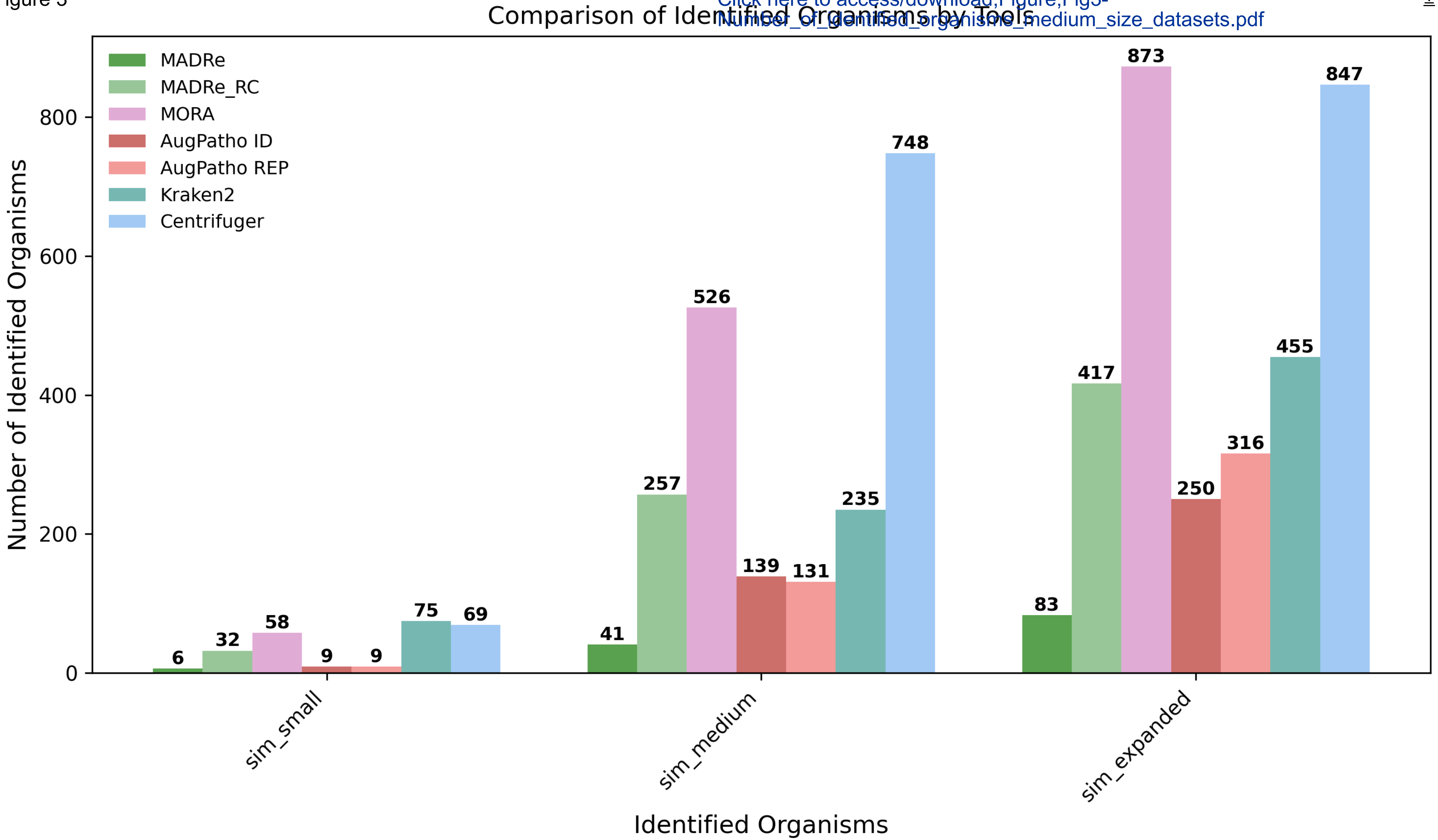

Figure 4
Click here to access/download;Figure;Fig4-F1_scores_large_size_datasets.pdf

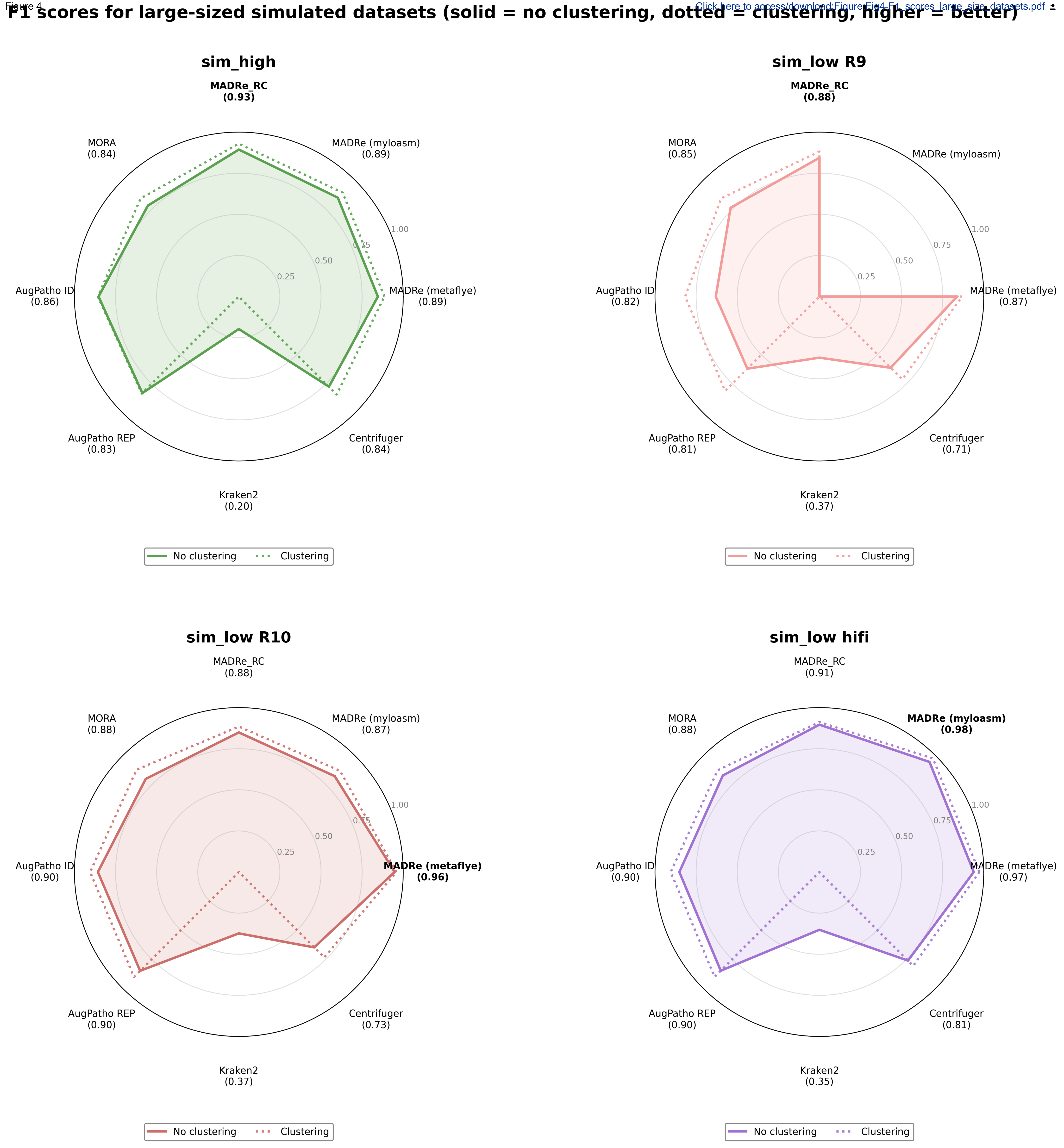

Click here to access/download;Figure;Fig5-BC_distances_zymo.pdf

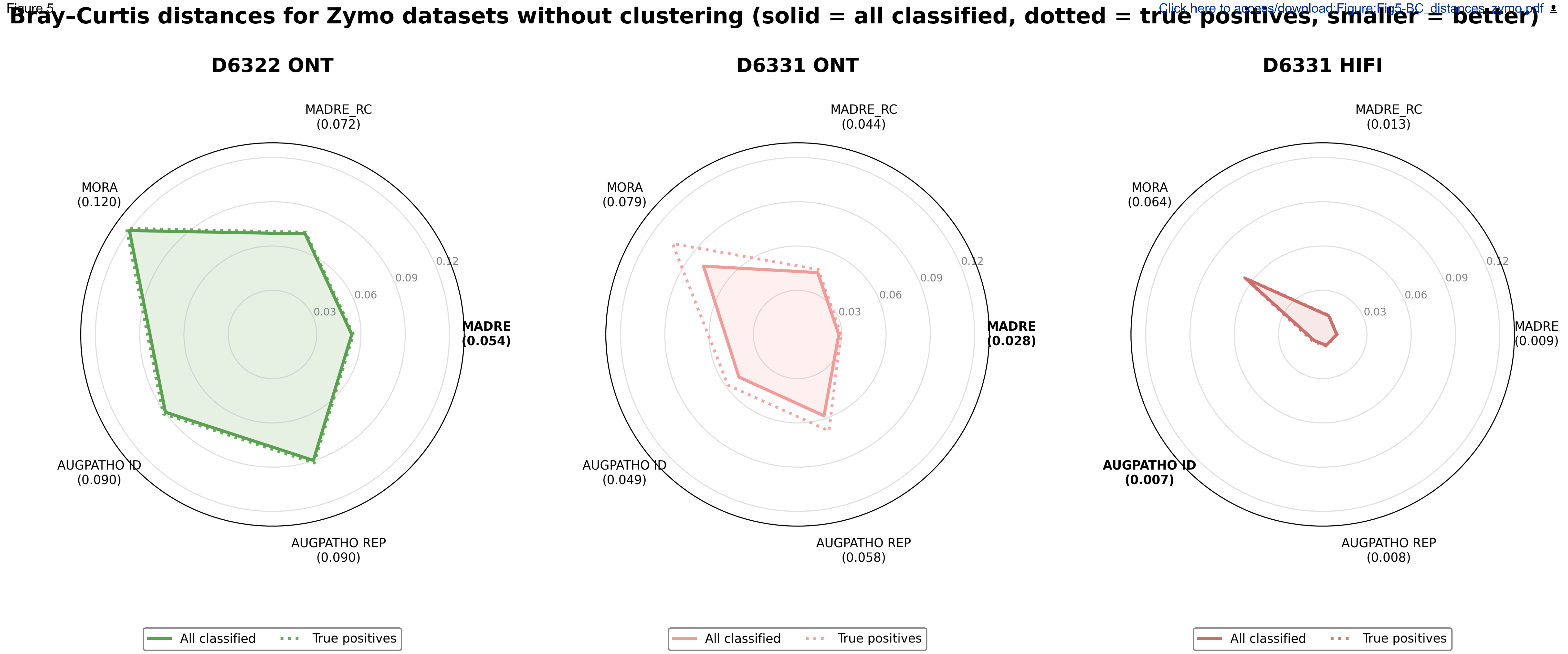

Figure 6

Click here to access/download;Figure;Fig6-rank_abundances_real_data.pdf

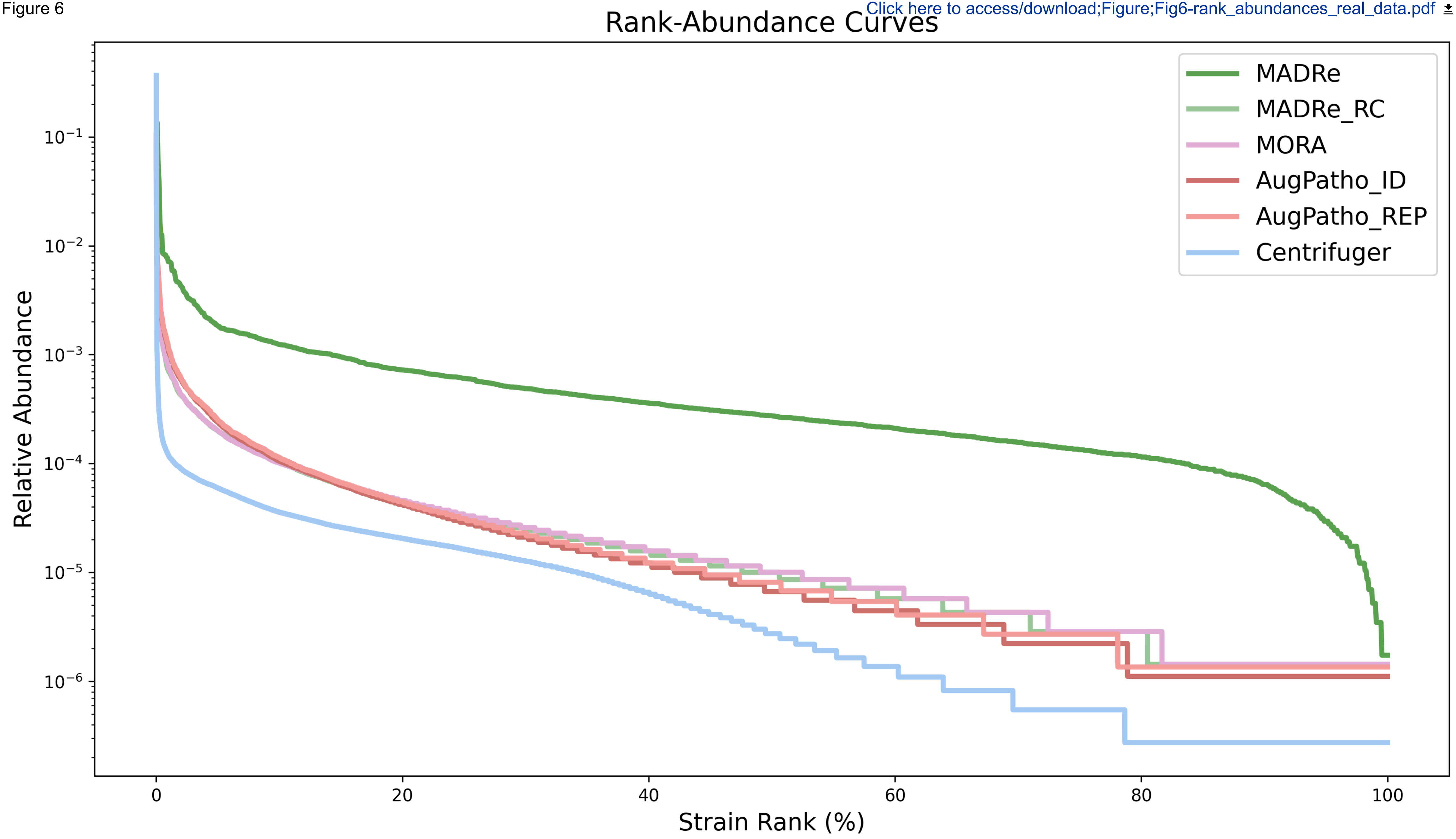

Figure 7

Click here to access/download;Figure;Fig7-relative_abundances_real_data.pdf

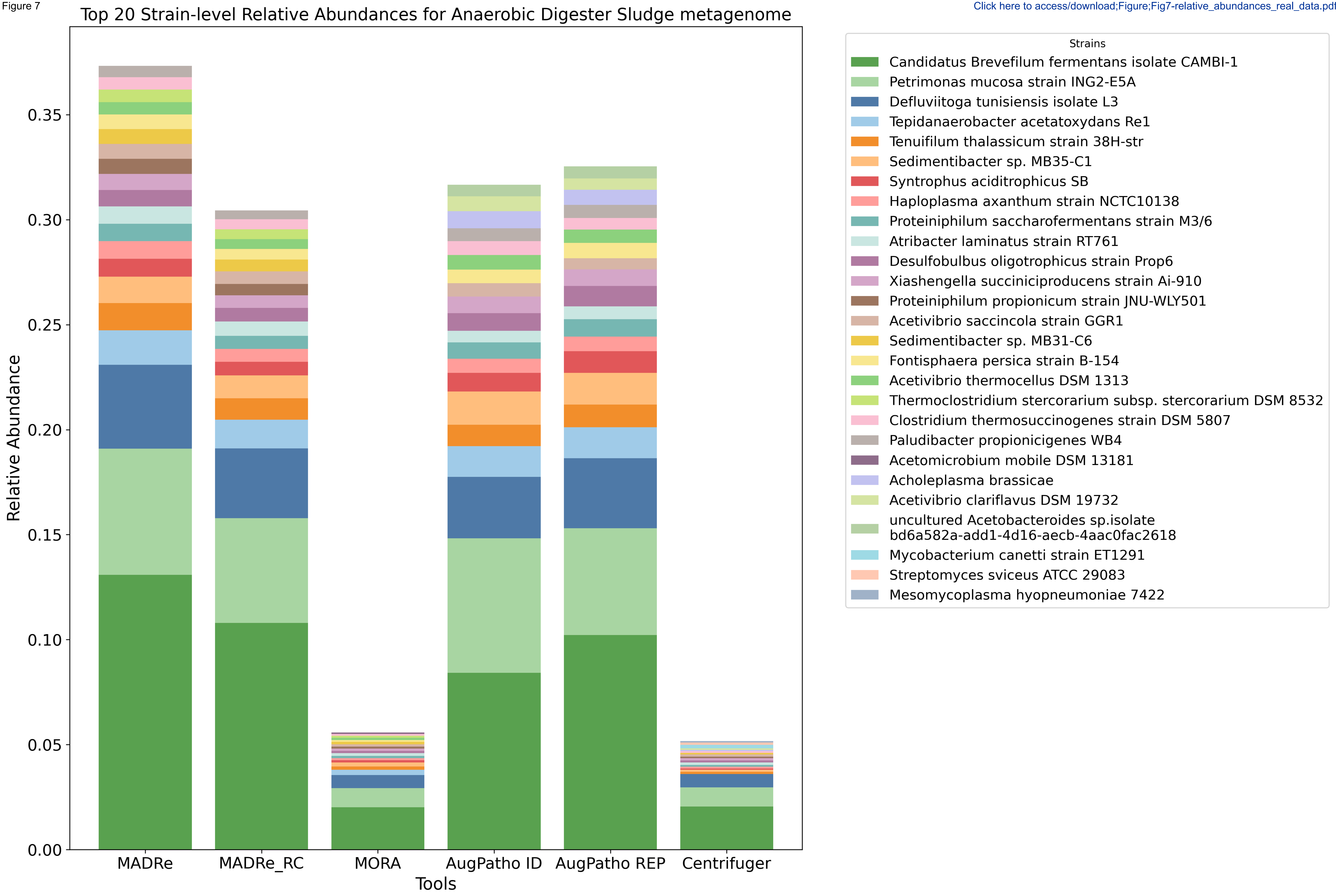

Figure 8

Click here to access/download;Figure;Fig8-computational_resources.pdf

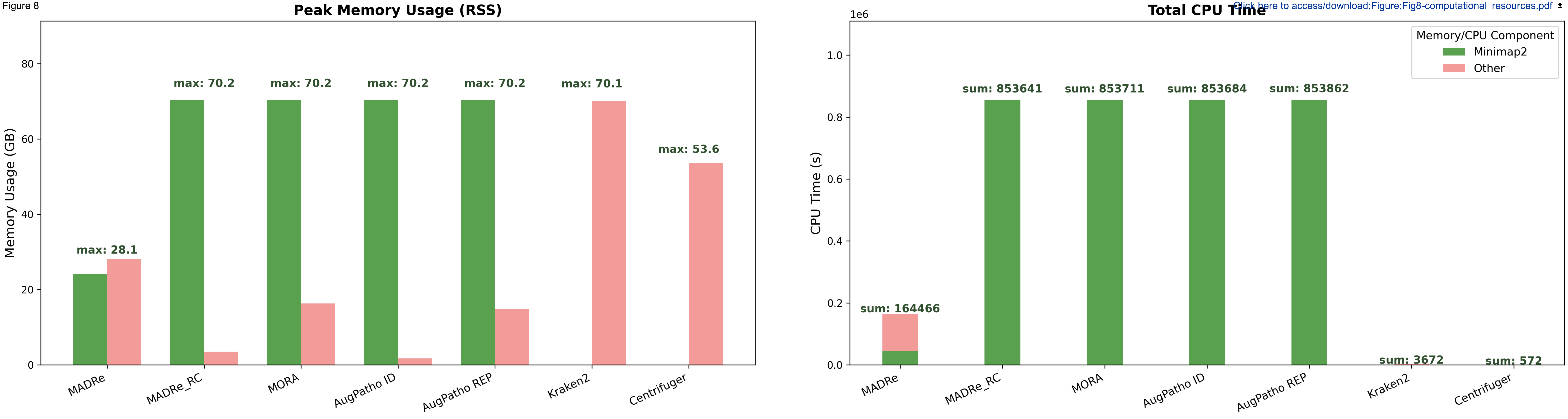

Supplementary Tables

Click here to access/download
Supplementary Material
MADRe-SupplementaryTables.xlsx

Supplementary File

Click here to access/download
Supplementary Material
MADRe-SupplementaryFile.pdf

Cover letter from first submission
Click here to access/download;Personal Cover;MADRe cover letter - GigaScience - first submission.pdf

Editorial Office
GigaScience
24 June 2025

Dear Editorial Team,

We are very excited to submit our manuscript, *“MADRe: Strain-Level Metagenomic Classification Through Assembly-Driven Database Reduction”* by Josipa Lipovac, Mile Šikić, Riccardo Vicedomini, and Krešimir Križanović, for consideration as a Technical Note in *GigaScience*.

In this study, we present MADRe, an open-source pipeline specifically designed for long-read metagenomic data. MADRe integrates metagenome assembly for reference database reduction with probabilistic, mapping-based read classification. This approach addresses a key challenge in metagenomics: resolving strain-level diversity in complex samples without prior knowledge of composition, while remaining scalable to large, diverse reference databases. Strain-level resolution is critical, as even closely related strains may differ in functional roles or pathogenicity.

Unlike many existing tools, MADRe avoids species-level database pre-filtering and supports databases containing over 100,000 reference genomes. By using genome assembly, MADRe enables accurate classification focused on confidently represented strains, while reducing computational demands. We evaluated MADRe across simulated datasets, mock communities, and a real metagenomic sample. On simulated datasets, MADRe achieved up to 20% improvement in correct strain-level assignments over state-of-the-art tools. On real mock community data, MADRe produced more accurate and precise abundance estimates, reducing distance to the ground truth by up to 39%. Additionally, MADRe reported significantly fewer false positives while maintaining high classification accuracy.

In line with *GigaScience*'s open science principles, MADRe emphasizes reproducibility and accessibility:

- Code is available under an OSI-approved license at https://github.com/lbcb-sci/MADRe;
- Docker images and Conda packages support reproducible deployment;
- All test datasets and outputs are available in public repositories for peer review.

Please note that while we have provided a BioProject accession for the Zymo D6322 dataset, the dataset will be made publicly available through an upcoming study. We will update the manuscript to include its DOI as soon as it is released.

All authors have approved the submitted version. The manuscript has not been published in a peer-reviewed journal but is available as a preprint on bioRxiv. There

are no issues regarding journal policies. We disclose that Mile Šikić is jointly funded by Oxford Nanopore Technologies and AI Singapore for a related project. The remaining authors declare no competing interests.

Thank you for considering our work. We look forward to your feedback.

Sincerely,

Josipa Lipovac
Faculty of Electrical Engineering and Computing, University of Zagreb
josipa.lipovac@fer.unizg.hr
(on behalf of all co-authors)

Click here to access/download;Personal Cover;MADRe cover letter - GigaScience - resubmission.pdf

Editorial Office
GigaScience

6th November 2025

Dear Editorial team,

We are pleased to resubmit our revised manuscript entitled **"MADRe: Strain-Level Metagenomic Classification Through Assembly-Driven Database Reduction"** for consideration in GigaScience.

We are grateful to the editor and the reviewers for the constructive and detailed feedback on our initial submission. We believe that all concerns raised were highly pertinent and have led us to significantly improve both the manuscript and the underlying software.

In this revised version, we have:

- **Addressed all reviewer comments**, incorporated the suggested changes into both the manuscript and the method, and thoroughly revised the text to clarify methodological details and enhance overall readability. **A detailed point-by-point response to the reviewers' comments accompanies this submission.**
- **Expanded the evaluation framework** by introducing multiple additional simulated datasets, including challenging cases with near-identical strains, and by incorporating additional comparative approaches. These updates were designed to fully address reviewer suggestions regarding robustness, accuracy, and the breadth of benchmarking.
- **Enhanced the software implementation** by carefully revisiting the species-level classification logic. Through additional benchmarking on complex simulated datasets, we identified and corrected inconsistencies in how low-similarity mappings were handled, resulting in improved precision and consistency of taxonomic assignments. We especially thank the reviewer for suggesting the use of more complex simulated datasets, as these experiments were instrumental in revealing and resolving this issue.
- **Improved usability, reproducibility, and performance.** In particular, we removed a previously hard-to-compile external dependency that caused installation issues, prepared openly available example datasets and detailed step-by-step tutorials to facilitate testing and reproducibility, and implemented several smaller code optimizations.
- **Updated all analyses and results** using the improved version of MADRe, ensuring consistency across figures, tables, and supplementary materials.

We sincerely thank the reviewers and editorial team for their time and constructive feedback, which have greatly contributed to improving the overall quality of our work. We hope that the revised manuscript now meets GigaScience's standards and look forward to your favorable consideration.

In line with GigaScience's scope, MADRe emphasizes reproducibility, modularity, and open science:

- All code is publicly available under an OSI-approved license;
- Conda packages are provided for reproducible deployment;
- All test datasets and outputs are available in public repositories to support peer review and reusability.

With kind regards,

Josipa Lipovac
Laboratory for Bioinformatics and Computational Biology, Faculty of Electrical Engineering and Computing, University of Zagreb, Zagreb, Croatia
josipa.lipovac@fer.unizg.hr
(on behalf of all co-authors)

 Click here to access/download;Personal Cover;MADRe - GigaScience - responses to reviewers.docx

We thank the reviewers for their constructive and valuable feedback on our manuscript.
Below, we provide *detailed responses* to each of the raised concerns.
Manuscript changes corresponding to the comments are indicated by line numbers.
All modifications in the manuscript are highlighted in yellow; if an entire section has been substantially modified, only the section title is highlighted.

Reviewer 1

Authors present MADRe a pipeline for classifying strains in metagenomics assembly. The pipeline evidently leverage contigs to reference mapping for the classification task. Authors have hosted the tool in Anaconda which is a fantastic initiative. Installation was a breeze and I personally appreciate this effort. However, I believe some helper scripts with complete examples could be included to run the tool out of the box on some test data.

We appreciate the reviewer's positive feedback regarding the Anaconda installation and their suggestion to include helper scripts and example data. In the revised version, we have substantially improved usability and reproducibility. Specifically, we fixed issues in the Conda installation and added a detailed step-by-step tutorial with an example dataset to our GitHub page (https://github.com/lbcb-sci/MADRe/blob/main/toy_example/Tutorial.md). This enables users to test and run the entire MADRe workflow.

Figure 1 must be reproduced with a higher resolution. Some of the text is illegible.

The figure has been replaced with a higher-resolution version to ensure all text and details are clearly legible.

Could the authors comment on how accuracy of PAF alignments affect the performance? In my understanding PAF does not perform pairwise alignment but rather use minimizers. Which means, the matching region might not be very accurate or be too short without match extensions. What was the reason for using PAF - is it to reduce performance overhead?

We appreciate the reviewer's question. Minimap2 outputs mapping positions in the Pairwise mApping Format (PAF) which may consist of either approximate (i.e., minimizer-based) or base-level alignments. MADRe performs two minimap2 runs, each serving a different purpose and using different alignment configurations.

In the first run, MADRe maps contigs to a large reference database. The goal of this first step is not to obtain highly accurate base-level alignments but rather to identify candidate reference genomes that share sequence similarity with the input contigs. Here, we intentionally use the fast minimizer-based mapping to reduce computational overhead, which is critical given the size of the reference database. For example, in the Zymo D6331 dataset used in our benchmarking, this approach was approximately 1.33x faster and required about 2x less memory than alignment-based strategy. Despite the approximate nature of this

alignment, we observed that combining it with a subsequent EM-based reassignment step yields robust organism identification.

In the second minimap2 run, MADRe maps reads to a reduced reference database derived from the first step. At this stage, highly accurate mappings are essential, as this step directly informs strain-level classification. To achieve this, we use the minimap2 parameter -c that outputs base-level alignments, ensuring that the alignments are sufficiently accurate for downstream reassignment.

*Exact minimap2 command lines and parameters used in both steps are provided in the **Supplementary File - "Tools versions and commands"** for full reproducibility.*

Given the nature of EM algorithm - I believe one contig will always match to one reference. However, there might be shared regions where the assembler has collapsed reads from two origins into one contig. Could you please comment on this, and if it fits add to limitations.

We appreciate the reviewer's thoughtful comment and agree with the observation. The reviewer is correct that assemblers often collapse reads from similar regions of different strains into a single contig. In such cases, it is important that one contig can contribute to the identification of multiple references.

*MADRe allows for this by computing a summarized mapping value for each contig-reference pair (as described in the **Methods section - lines 845-850**), taking into account all mappings of the contig to that reference. If a contig maps to multiple references, it is marked as a non-unique mapping and passed into the EM refinement step.*

The EM algorithm outputs posterior probabilities for each contig-reference pair. Based on these probabilities, MADRe assigns the contig to a maximum of N references, where N is an estimate of the number of strains collapsed into the contig. This estimate is based on the estimation obtained from the HairSplitter tool. In this way, a single contig can contribute to multiple strain assignments, addressing the issue of collapsed regions directly.

While the conducted work is substantial, I am curious to see how this stands up with other assemblers like BLAST and classification tools like Kraken2. Most importantly, because minimap2 and PAF is designed for READ to READ or READ to contig alignment.

We sincerely thank the reviewer for this thoughtful comment.
We would like to clarify that BLAST is a widely used sequence alignment tool known for its accuracy; however, it is considerably slower than modern mapping or classification approaches and is not optimized for long-read data. Therefore, it was not included in our comparisons, which focused on classification performance rather than alignment speed or sensitivity alone.

*Regarding Kraken2, we did include it in our benchmarking. Since Kraken2 is primarily designed for species-level classification and its output cannot be directly evaluated at the strain level, we specifically selected organisms in our medium-sized simulated datasets whose strain-level taxids differ from their species-level taxids, enabling a strain-level evaluation. This clarification is part of the section **Benchmarking details (lines 155–161)**.*

We hope that the previous answers help clarify how we adapted minimap2 for initial identification based on EM refinement, followed by a post-classification step to achieve accurate strain-level assignments.

Also the benchmark metrics must be re-considered. Please include more relevant metrics to measure genome completeness, recovery of genes. Use a tool that measure the quality of genomes using marker genes, length, etc (may be meta Quast). CPU and Memory benchmarks could be replaced by above qualitative genome metrics and these can go in the supplementary.

We appreciate the reviewer's suggestion. However, we would like to respectfully clarify that MADRe is a metagenomic classification tool, not a metagenome assembler. Although MADRe relies on metagenome assembly as an input, it uses assembled contigs primarily for the purpose of taxonomic identification, not for genome reconstruction.

As such, metrics that assess assembly quality, such as genome completeness, marker gene recovery, or contig length are not directly applicable for evaluating MADRe's classification performance. In fact, one of MADRe's strengths is its ability to make use of even short or fragmented contigs, which often arise in real-world samples, particularly for low-abundance or hard-to-assemble strains. These contigs can still carry enough signal to contribute meaningfully to accurate classification.

For this reason, we focused our benchmarking on taxonomic classification metrics including CPU and memory benchmarks, which are more relevant for evaluating the goals and output of MADRe. CPU and memory usage are highly relevant, as they reflect MADRe's computational efficiency, a key feature of the method, especially given the size of metagenomic datasets and reference databases. For this reason, we believe it is more appropriate to include resource usage benchmarks rather than genome quality metrics, and we have kept those results in the main text.

Reviewer 2

The manuscript presents MADRe, an assembly-driven workflow that (i) assembles the long reads, (ii) uses an EM procedure to select a reduced set of candidate reference genomes, and (iii) remaps all reads to this reduced set with a probabilistic re-assignment algorithm. The idea is attractive from a computational-efficiency standpoint, but several methodological questions remain unresolved.

At the nucleotide level, two strains of the same species can differ by only a handful of SNVs per 100 kb. Short Illumina reads (Q≈40, error rate ≈0.01 %) are currently the work-horse for detecting such subtle variation, whereas ONT R10.4 or even raw HiFi reads still have ≥0.1 - 1 % error after standard base-calling. Please explain quantitatively how MADRe overcomes this intrinsic accuracy gap when assigning a read or a contig to the correct strain. Is the EM step robust enough to separate strains that differ by ≤10 SNVs across an entire genome when the read error profile is at least an order of magnitude larger? The manuscript should include a benchmark in which the target strains differ by very few SNVs (≤0.01 % ANI) and

the pipeline is evaluated with both raw ONT/HiFi reads and Illumina reads. Without such evidence it remains unclear whether MADRe can truly deliver "strain-level" accuracy in the biological sense of the term.

*We thank the reviewer for this insightful and important question. To address it, we added a detailed description of controlled experiments in the Supplementary File (**Supplementary File - "Similar Strains Experiments"; Supplementary Table 23**) illustrating how MADRe behaves under specific scenarios, including cases with near-identical genomes (fastANI 99.98–99.9996%). In these experiments, we compared MADRe with direct competitors - MORA and AugPatho (PathoScope2) across several controlled conditions (removal of close references, altered abundances, and strain removal from starting dataset) to highlight the characteristic "attractor" behavior observed in all mapping-based tools. MADRe, which is centroid-based, consistently favored the most central genome within each group, while AugPatho and MORA tended to collapse or redistribute reads based on their respective probabilistic or abundance-driven models.*

These differences clarify why each method exhibits distinct attractor patterns and emphasize the inherent difficulty of resolving strains at ≤0.01% divergence. For this reason, in our evaluation we also report results at a "cluster level", where extremely similar genomes are grouped together based on mapping profiles. This approach avoids penalizing methods for inevitable redistribution within such groups and provides a more biologically meaningful measure of performance.

We did not include Illumina-only benchmarks, as the evaluated tools are specifically designed for long-read classification. Moreover, MADRe relies on contig assembly from long reads, and short-read assemblies generally lack the resolution and completeness required for a meaningful comparison.

*Exact commands and parameters for these experiments are provided in the Supplementary File for full reproducibility, and the related results are also mentioned in the **Discussion section (lines 726–741)**.*

In true microbiome samples (human gut, soil, marine) many strains have no close representative in RefSeq/GTDB. Traditional workflows (StrainPhlAn 4, metaSNV, DESMAN, MIDAS, etc.) therefore reconstruct strain haplotypes de-novo or relative to distant references rather than forcing each read onto the nearest known genome. MADRe never clarifies what it does when the correct strain is missing. Does the EM step collapse the reads onto the nearest neighbour? How are the resulting abundances or functional inferences interpreted?

*We thank the reviewer for raising this important point. To illustrate how MADRe behaves when the correct strain is missing from the reference database, we performed a dedicated test as part of the controlled experiments (**Supplementary File – Similar Strains Experiments; Supplementary Table 23**). In this test, we removed two genomes from the database: S. anginosus strain NCTC10713, which had two closely related strains still present, and A. equolifaciens subsp. celatus strain JCM 14811, which is more distinct from the remaining strain of the same species.*

When S. anginosus strain NCTC10713 was excluded, MADRe reassigned reads primarily to the most similar remaining genome, S. anginosus subsp. anginosus strain FDAARGOS_1569. In contrast, when A. equolifaciens subsp. celatus JCM 14811 was removed, most reads were reassigned to A. equolifaciens DSM 19450 (the only other strain of that species in the database), while a smaller fraction mapped to A. hattorii strain 8CFCBH1, the nearest related species outside that pair.

These results show that when exact strains are missing, MADRe's EM step redistributes reads to the closest remaining genomes based on sequence similarity. Consequently, abundance estimates and functional interpretations are best analyzed at the cluster level rather than at the level of individual strains, as this provides a more robust and biologically meaningful representation under such conditions.

*Exact commands and parameters for this experiment are provided in the Supplementary File for full reproducibility, and the related results are also mentioned in the **Discussion section (lines 726–741)**.*

A convincing evaluation would simulate a dataset in which there is several genome per species present in the database. At present the benchmark always contains an almost exact reference for each strain, so we do not learn how MADRe behaves in the real-world settings.

We thank the reviewer for this valuable comment. We believe there may have been a misunderstanding regarding our experimental setup. In all of our benchmarks, we used a large reference database that contained multiple strains of the same species - often with very high sequence similarity - so the evaluation was not limited to a single reference per strain.

*It is true, however, that the datasets used in the initial version of the manuscript did not include closely related strains among the reads themselves, which may have created the impression of near-exact references. To address this, we have now expanded our evaluation with additional datasets that include several highly similar strains of the same species. This new experiment provides a more direct assessment of MADRe's performance in realistic scenarios where closely related genomes coexist both in the database and within the input dataset (**Supplementary File - Similar Strains Experiments; Supplementary Table 23**).*

It is also important to emphasize that MADRe's primary goal is not de novo strain reconstruction, but rather accurate classification of reads to the most similar available reference, facilitating reliable downstream analyses such as abundance estimation and comparative genomics.

*Exact commands and parameters for this experiment are provided in the Supplementary File for full reproducibility, and the related results are also mentioned in the **Discussion section (lines 726–741)**.*

Kraken 2 is known to perform poorly at species assignment; Sylph is much better k-mer based classifiers at the species level. By including Kraken 2 but excluding state-of-the-art profilers such as MetaPhlAn 4.2 (which now uses Minimap2 and provides strain-clade resolution), StrainPhlAn 4, SYLPH, or MetaSNV, the study under-estimates the baseline.

We thank the reviewer for this valuable comment. Our selection of baselines was guided by practical considerations of comparability and scope. While Kraken2 is indeed known to be less accurate at the species level than some newer classifiers, it remains one of the most widely used tools in metagenomic studies and thus serves as a relevant benchmark for evaluating improvements relative to common practice.

We carefully considered including MetaPhlAn 4.2, StrainPhlAn 4, Sylph, and MetaSNV. However, each of these tools presents limitations that make direct comparison with MADRe challenging. MetaPhlAn 4.2 and StrainPhlAn 4 are marker-based approaches that reconstruct a per-species consensus genotype and position it in a phylogenetic tree, but they do not provide quantitative estimates for multiple coexisting strains. In mixed-strain samples, StrainPhlAn collapses the signal into a single consensus sequence, reporting only the dominant haplotype and discarding information about minority strains. This output is therefore not directly comparable to MADRe, which explicitly models strain mixtures and produces abundance profiles for multiple clusters of closely related genomes.

Sylph is a promising k-mer-based method with a lower false-positive rate than Kraken2; however, it does not generate reference labels for classified reads, preventing systematic evaluation against known ground truth within our benchmarking framework (section ***Benchmarking details - lines 161-167)****. MetaSNV, on the other hand, was specifically designed for high-coverage Illumina short-read data, where accurate SNV calling is feasible, and is therefore not applicable to the long-read datasets analyzed in this study.*

For these reasons, we restricted our evaluation to tools that produce abundance profiles directly comparable to MADRe. Nonetheless, we recognize that marker-based and SNV-based profilers represent important complementary strategies, and we have described that in the ***Introduction section (lines: 14-20)*** *to acknowledge these recent developments and clarify the intended scope of our comparisons.*

Tools such as MetaMaps, Centrifuge, PanTax, and StrainGE are dismissed on the ground that "indexing crashed"

We thank the reviewer for this comment and would like to clarify this point. In our benchmarking, we attempted to include tools such as MetaMaps, Centrifuge, PanTax, and StrainGE, but we encountered indexing failures when using the full database. One possible workaround would be to construct smaller, tool-specific databases, but we felt that this would not provide a fair comparison, since the core motivation behind MADRe is to enable classification against very large and comprehensive databases that are otherwise difficult or impossible to index. We also note that in our initial evaluation we did not include Centrifuger, the more recent successor of Centrifuge. However, we later added it to our comparison precisely because it is designed to handle large databases and thus provides a more appropriate benchmark against MADRe.

What are the parameters of DBSCAN ? Default ?

In our analyses, we used DBSCAN clustering with precomputed Jaccard distances, an ε (eps) parameter of 0.9, and min_samples = 1. These details were previously included in the

Supplementary File but have now been added to the main manuscript for clarity ***(Methods, lines: 965-967)****.*

Reviewer 3

While the supplementary tables provide detailed information about the datasets used for evaluating different tools, they do not report the total data size for each dataset or the genome coverage (×) achieved per strain. This information is important to assess what abundance levels of metagenomic data MADRe is applicable to.

*We thank the reviewer for this helpful suggestion. We originally reported the read count and total read length for each strain but did not explicitly include the calculated genome coverages. We have now added this information for our sim_small, sim_medium, and sim_expanded datasets (****Supplementary Table 1****), where per-strain coverage was computed based on total read length and genome size. For the Zymo datasets, we refer to the corresponding public data sources, which already provide detailed sequencing statistics. Similarly, for the newly included PanTax simulated datasets (large-scale simulated datasets), all relevant information, including per-strain coverage, is available in the PanTax supplementary materials [1].*

[1] Zhang, Wenhai, et al. "Strain-level metagenomic profiling using pangenome graphs with PanTax." *bioRxiv* (2025): 2025-04.

The authors could consider incorporating more complex simulated datasets. Currently, the three simulated datasets (sim_small, sim_medium, sim_expanded) differ by only ~5-fold between the lowest- and highest-abundance strains. In sim_small, there are only four strains, which is too limited in complexity. In sim_medium, the number of strains per species is identical, and the strain abundances are also very similar. In reality, metagenomic datasets contain different numbers of strains per species and display much greater abundance variation. Additionally, the real dataset used later (the anaerobic digester sludge metagenome) contains over 1,000 strains, which is a substantial contrast to the 4, 15, and 30 strains in the simulated datasets. There is no direct evidence demonstrating that MADRe can be applied effectively to much more complex metagenomes.

We thank the reviewer for this constructive suggestion. To address the concern about dataset complexity, we expanded our benchmarking to include four additional simulated datasets originally introduced in the PanTax study. Three of these datasets each contain 60 genomes from 30 different species, simulated using ONT R9.4.1, ONT R10, and HiFi error profiles, respectively. These datasets were obtained directly from the PanTax Zenodo repository.

In addition, we generated a fourth, large-scale dataset comprising 1,000 genomes from over 300 species, inspired by the CAMI challenge design. As the PanTax authors did not provide simulated reads for this large dataset, we used the published reference genomes and expected abundances to simulate reads with the Badread tool.

*All four PanTax-based datasets were incorporated into our benchmarking to evaluate MADRe (and other approaches) under substantially more complex and heterogeneous community compositions (**Supplementary File - Simulated Datasets; Supplementary Tables 10–16**). We also added a new section in the main manuscript that presents and analyzes those results (**Results section - Classification of large-sized simulated datasets**) Furthermore, to illustrate MADRe's behavior in strain-specific scenarios, we added targeted experiments described in **Supplementary File – Similar Strains Experiments**.*

In the evaluation, the authors could consider including Centrifuger, which is a recent upgrade of Centrifuge and supports large-scale database construction.

We thank the reviewer for this helpful suggestion. We have now included Centrifuger in nearly all of our benchmarking experiments. However, we observed a limitation when Centrifuger cannot confidently assign a read to a specific reference sequence (for example, when multiple chromosomes belong to the same strain). In such cases, it often classifies the read under the NCBI strain-level taxid, which in some instances is identical to the species-level taxid. This makes it impossible to directly and fairly compare those classifications with other tools that operate at the sequence level.

For benchmarking consistency, we therefore considered as true positives only the reads correctly classified under the expected reference sequence. It is important to note that this issue affected a relatively small fraction of reads (approximately 9,000 out of ~5 million reads in the 1,000-genome dataset).

We did not include Centrifuger in the Zymo dataset benchmarks because, during database construction, we were unable to ensure consistent labeling of expected reference sequences, which prevented a fair and reproducible comparison.

The authors should provide ANI values for the strains included in the evaluation.

We thank the reviewer for this useful suggestion. We have now included ANI values for all strains used in the evaluation as part of the supplementary material (Supplementary Tables 2–4 and 10). Specifically, ANI matrices are provided for the sim_small, sim_medium, and sim_expanded datasets, as well as for genomes included in targeted experiments assessing highly similar strains. For the simulated datasets originating from the PanTax study, which contain a large number of genomes, we additionally provide summary distributions showing how many genome pairs exceed predefined ANI thresholds.

In the main text, it is stated that "HairSplitter's functionality to estimate the number of collapsed strains per contig and integrated this information with the mapping data of the initially strain-collapsed contigs." However, the authors of HairSplitter have noted that HairSplitter requires reads to span at least five polymorphic loci for effective separation into haplotype groups, which poses challenges in highly similar regions. Therefore, it is not well suited to distinguish very similar strains. The authors could add datasets with strains of varying similarity to discuss whether MADRe's filtering results are impacted by HairSplitter, and whether this limitation is mitigated by the additional post-classification clustering.

We thank the reviewer for raising this important point. Indeed, HairSplitter was designed to estimate the number of strains contributing to a collapsed contig and, as noted by its authors, it may underestimate strain multiplicity in highly similar regions where reads do not span enough polymorphic loci. To mitigate this bias, MADRe does not rely on HairSplitter directly but instead uses its output as an approximate guide for the database reduction stage. Specifically, MADRe applies a small offset (+2 strains by default) to the number reported by HairSplitter to avoid underrepresentation. This introduces a controlled over-prediction, ensuring that all potentially relevant strains are retained for downstream reassignment.

The resulting noise is then resolved during the strictness-based filtering step: strains whose mapped contigs exhibit significantly lower mapping quality or reassignment probability (below the user-defined strictness threshold) are pruned from the reduced database. This two-stage strategy balances sensitivity and specificity, deliberately favoring false positives over false negatives during reduction, since missing a true strain would irreversibly bias abundance estimation. As shown in Table 1, this approach maintains high recall (all true strains retained) while keeping the number of extra strains limited. By default, MADRe uses a high strictness setting, but for datasets expected to contain many nearly identical strains, we recommend less strict parameters that modestly increase false positives while improving recovery of true coexisting strains. The use of these parameters is documented in our ***GitHub tutorial: https://github.com/lbcb-sci/MADRe/blob/main/toy_example/Tutorial.md.***

We also clarify that clustering is not part of the MADRe algorithmic pipeline itself. Clustering is applied only during evaluation - precomputed once over the full reference database and used uniformly across all methods to report cluster-level metrics. It therefore cannot "recover" genomes that are absent from a reduced database.

It would be helpful if Table 1 could show the reduction ratios and Table 2 could report F1 scores to reflect the accuracy of species- and strain-level identification. Reporting only the number of detected true positives is insufficient: a high TP count can result from low precision, making it difficult for readers to assess performance intuitively.

We thank the reviewer for this valuable comment. Reporting the reduction ratio would not be particularly informative in our case, as the values would be extremely small - given that the original database contains over 100,000 genomes, while the reduced databases include at most 234 genomes. Instead, we have made Table 1 more informative by adding the number of expected genomes and explicitly indicating the size of the starting database.

We also wish to clarify that Table 2 reports the number of false positives (FPs), not true positives (TPs). Because all approaches produce a large number of FPs relative to TPs, the F1 score alone does not adequately capture the distinction in performance we aim to illustrate. We therefore find that directly reporting the number of false positives provides a clearer and more interpretable comparison across methods.

Nevertheless, to provide a more comprehensive overview, we have expanded ***Supplementary Table 8*** *to include the full set of metrics: TP, TN, FP, FN, accuracy, precision, recall, and F1 score.*

In the Zymo dataset, AugPatho has the highest number of strain-level false positives. However, in the real anaerobic digester sludge metagenome dataset, the number of strains uniquely detected by other tools (such as MADRe) is much greater than those reported by AugPatho. It would be helpful to explain this discrepancy.

We thank the reviewer for this insightful observation. The differences in performance across the three datasets can be attributed both to sequencing technology and to the evaluation criteria used. As expected, the D6331 HiFi dataset yielded the best results due to the higher base-level accuracy of HiFi reads compared to ONT. At first glance, it may seem surprising that performance on D6322 ONT was lower than on D6331 ONT, since D6322 contains species from different genera and should, in principle, be easier to classify.

The main factor behind this apparent discrepancy lies in how ground-truth labels were defined. For D6322, evaluation was straightforward: each genome belongs to a distinct species, so we required exact species-level matches. In contrast, D6331 includes five E. coli genomes, three of which have very high sequence identity (greater than 99.3% ANI score). When constructing the ground truth, we clustered these three genomes and treated any read originating from one of them as correctly classified if it was assigned to any genome within that cluster. This less strict definition can lead to higher apparent performance on D6331 compared to D6322, and this effect applies equally to all evaluated tools.

We added this explanation to ***the Discussion section (lines: 691-712)****.*

AugPatho has two components: "The PathoID module" and "PathoReport" The authors only used "The PathoID module" for method comparison. However, "PathoReport" further improves the accuracy of AugPatho results. It would be helpful if the authors could explain why they did not run "PathoReport." Otherwise, the evaluation may appear less fair to AugPatho.

We thank the reviewer for this helpful comment. Initially, we did not include the PathoReport module due to technical issues encountered when integrating it with our custom mapping files. Specifically, because we used minimap2-generated mappings instead of AugPatho's internal alignment workflow, a hardcoded file path in the PathoReport code caused execution errors that prevented the module from running successfully.

We have now resolved this issue and rerun all relevant benchmarking experiments with both PathoID and PathoReport enabled. The updated results have been incorporated throughout the revised manuscript, ensuring a fair and comprehensive comparison that reflects the full functionality of AugPatho.

Supplementary Table 5 shows classification evaluation based on read counts in the three simulated datasets. It would be helpful to also evaluate performance by the number of detected species and strains, using metrics such as TP, TN, FP, FN, precision, recall, and F1 score.

We thank the reviewer for this helpful suggestion. We have now expanded ***Supplementary Table 8*** *to include evaluation based on the number of detected species and strains, reporting TP, TN, FP, FN, precision, recall, and F1 score metrics.*

The clustering of detected strains is an important feature. The authors should describe the clustering parameters and thresholds used in the study.
How sensitive are the results to these parameter choices?

We thank the reviewer for this comment. In our analyses, we used DBSCAN clustering with precomputed Jaccard distances, an ε (eps) parameter of 0.9, and min_samples = 1. These details have been added to the manuscript ***(Methods, lines: 965-967)****.*
It is important to note that clustering is not part of MADRe's core classification pipeline but rather serves as an evaluation step, applied equally across all methods to ensure a fair comparison. The purpose of clustering is to account for cases where highly similar or redundant genomes exist in the database, such as alternative assemblies of the same strain differing only due to assembly artifacts.
At this stage, MADRe provides users with the flexibility to decide how to handle the detected organisms. The current output represents centroid detections, which serve as a starting point for downstream metagenomic analyses. Thus, the clustering component primarily facilitates interpretation and downstream refinement rather than directly influencing MADRe's classification.
Alternative clustering strategies (e.g., based on ANI thresholds) could also be applied. However, our approach better reflects the similarity patterns present in the data. Future work will include a more detailed investigation of clustering strategies and their integration into the full MADRe pipeline.
We described this in ***Discussion (lines: 742-761)****.*

Additionally, in the Methods section, could the authors provide the exact formulas or evaluation procedures used when assessing results after clustering? Readers may wonder whether true-positive strains within a cluster could be masked by a representative strain classified as a false positive, thus impacting the reported F1 score.

We thank the reviewer for this thoughtful question and the opportunity to clarify our evaluation procedure. In our evaluation, if a read is assigned to the genome that belongs to a cluster that contains at least one true-positive (TP) genome, it is considered true positive as well. This ensures that true detections are not masked by the representative strain within the same cluster. This explanation can be found in the ***Methods - Evaluation details section (lines: 1042-1049)****.*

The authors should consider including additional popular long-read assemblers such as hifiasm and the latest ONT assemblers like Myloasm as alternatives within the MADRe workflow.

We thank the reviewer for this valuable suggestion. In our updated evaluation using the large-sized (PanTax) datasets, we included Myloasm as an assembler for both ONT R10 and HiFi data. The overall results were comparable to those obtained with metaFlye and metaMDBG, with only a slight improvement observed in the large 1,000-genome dataset (F1 = 0.85 for Myloasm vs. 0.84 for metaFlye; ***Supplementary Tables 15–16****).*

Interestingly, there were 24 genomes detected only when using Myloasm and 11 genomes detected only with metaFlye. Although Myloasm appears slightly better overall, we observed

that among the 11 genomes missed by Myloasm, 9 belong to the more abundant half of the community, whereas among the 24 genomes detected exclusively by Myloasm, only one was from that higher-abundance group. This suggests that Myloasm contigs perform better in detecting low-abundance strains, while metaFlye contigs are more robust for high-abundance strains.

These results highlight that the choice of assembler can influence detection sensitivity across different abundance ranges and are discussed in the main manuscript ***(Discussion - lines: 764-773)****. Accordingly, we have already integrated Myloasm into the MADRe workflow and plan to provide detailed guidelines and recommendations for using different long-read metagenomic assemblers in future MADRe releases.*

MADRe failed to run on hifi datasets, and several bugs were encountered. I strongly recommend that the authors provide a working minimal example on GitHub, or host a reproducible instance (e.g., via Code Ocean). In addition, the experimental code used for the manuscript should be made available to ensure reproducibility.

We thank the reviewer for this important comment. We have resolved the installation issues and verified that MADRe now runs successfully on HiFi datasets. To ensure full reproducibility and ease of use, we have prepared a detailed step-by-step tutorial with a working "toy example" available on our ***GitHub page: https://github.com/lbcb-sci/MADRe/blob/main/toy_example/Tutorial.md.*** *This tutorial includes all commands and expected outputs, allowing users to reproduce the complete MADRe workflow out of the box.*

In the main text (MADRe Read Classification section), the authors mention a species-specific algorithm for refining read assignments. Additionally, the supplementary materials (Read Classification section) state that "for each species, read assignments are refined using mapping-based probability reassignment." However, the manuscript does not describe the detailed procedure of this algorithm. It would be helpful if the authors could provide a more explicit explanation of this process. Clarifying this aspect would improve reproducibility and help readers better understand how the final read assignments are derived.

We thank the reviewer for this insightful comment and the opportunity to clarify this part of the workflow. The sentence cited by the reviewer refers to a detailed explanation already provided in the Methods section. However, to improve clarity and reproducibility, we have revised both ***the Supplementary File (Read Classification section)*** *and* ***the Methods section (MADRe Read Classification)*** *to make the description of the mapping-based probability reassignment algorithm more explicit and easier to follow.*

In Experiment 1, Table 1 refers to results from the database reduction step only, whereas in Experiment 2, Table 2 and Figure 2 refer to the full MADRe pipeline (reduction + classification). Reusing the same "MADRe" label across both contexts may confuse readers. It would be clearer to use distinct labels (e.g., MADRe-reduction, MADRe_RC, MADRe) to indicate which pipeline steps are being evaluated.

We thank the reviewer for this helpful observation. To avoid confusion, we have updated the manuscript to use distinct labels that clearly indicate which pipeline components are being evaluated (e.g., MADRe-reduction, MADRe_RC, MADRe). This change ensures that readers

can easily distinguish between the database reduction step and the full MADRe workflow (reduction + classification).

The Genome Taxonomy Database (GTDB) provides a comprehensive redefinition of bacterial and archaeal taxonomy and is increasingly adopted in the field. It would be helpful if the authors could provide guidance or a pipeline on how to use GTDB taxonomy with MADRe.

We thank the reviewer for this helpful suggestion. We have now added detailed guidance and accompanying scripts for preparing and integrating GTDB taxonomy with MADRe. These materials are included in the updated GitHub documentation and Supplementary Files, allowing users to easily build and use GTDB-based databases within the MADRe workflow.
