# Supplementary material for: MADRe: Strain-level metagenomic classification through assembly-driven database reduction
Source: Gigascience. 2026 Mar 23;15:giag030. doi: 10.1093/gigascience/giag030 (PMC13211987; doi:10.1093/gigascience/giag030)
Supplement: giag030_GIGA-D-25-00468_Revision_1 [file giag030_giga-d-25-00468_revision_1.pdf]

GigaScience

MADRe: Strain-Level Metagenomic Classification Through Assembly-Driven Database Reduction

--Manuscript Draft--

**Manuscript Number:**	GIGA-D-25-00468R1
**Full Title:**	MADRe: Strain-Level Metagenomic Classification Through Assembly-Driven Database Reduction
**Article Type:**	Technical Note
**Funding Information:**	Hrvatska Zaklada za Znanost (IP-2018-01-5886) — prof.dr.sc. Mile Šikić<br>Hrvatska Zaklada za Znanost (MOBDOK-2023-2941) — Ms Josipa Lipovac<br>Singapore Ministry of Health's National Medical Research Council (MOH-000649-01) — prof.dr.sc. Mile Šikić<br>DATACROSS (2024-2026, PK.1.1.10.0007) — dr.sc. Krešimir Križanović
**Abstract:**	Strain-level metagenomic classification is essential for understanding microbial diversity and functional potential, yet remains challenging, particularly when sample composition is unknown and reference databases are large and redundant. Here we present MADRe, a modular and scalable pipeline for long-read strain-level metagenomic classification based on Metagenome Assembly-Driven Database Reduction. Beyond system-level integration, MADRe introduces statistical strategies that leverage assembly-derived genomic context to guide database reduction and probabilistic read reassignment. Specifically, it combines long-read metagenome assembly, contig-to-reference reassignment using an expectation–maximization framework for reference reduction, and probabilistic read mapping reassignment on a reduced database to achieve sensitive and precise strain-level classification. We extensively evaluated MADRe on simulated datasets, mock communities, and a real anaerobic digester sludge metagenome. Across diverse similarity and coverage conditions, MADRe consistently improves precision by reducing false-positive strain detections. MADRe's design allows users to apply either the database reduction or read classification step individually. Using only the read classification step shows results on par with other tested tools. MADRe is open source and publicly available at https://github.com/lbcb-sci/MADRe.
**Corresponding Author:**	Josipa Lipovac<br>University of Zagreb Faculty of Electrical Engineering and Computing: Sveuciliste u Zagrebu Fakultet Elektrotehnike i Racunarstva<br>Zagreb, CROATIA
**Corresponding Author Secondary Information:**	
**Corresponding Author's Institution:**	University of Zagreb Faculty of Electrical Engineering and Computing: Sveuciliste u Zagrebu Fakultet Elektrotehnike i Racunarstva
**Corresponding Author's Secondary Institution:**	
**First Author:**	Josipa Lipovac
**First Author Secondary Information:**	
**Order of Authors:**	Josipa Lipovac<br>Mile Šikić<br>Riccardo Vicedomini<br>Krešimir Križanović
**Order of Authors Secondary Information:**	

Powered by Editorial Manager® and ProduXion Manager® from Aries Systems Corporation

Response to Reviewers:	For convenience, we have also attached a separate document containing cover letter and responses to facilitate the review process.<br><br>Editorial Office<br>GigaScience<br>2nd March 2026<br><br>Dear Editorial team,<br>We are pleased to resubmit our revised manuscript entitled "MADRe: Assembly-Driven Database Reduction for Strain-Level Long-Read Metagenomic Classification" for consideration in GigaScience.<br>We sincerely thank you and the reviewers for the constructive and insightful comments. We have carefully addressed all points raised and believe the manuscript has been substantially strengthened.<br>In this major revision, we have:<br>- Clarified strain-level evaluation for Centrifuger and Kraken2, including additional analysis and discussion of taxonomy-related limitations.<br>- Added new controlled synthetic experiments to better characterize performance across varying strain similarity and coverage levels.<br>- Expanded discussion of precision-recall trade-offs and clarified scenarios in which MADRe is most advantageous.<br>- Clarified limitations related to low-abundance strain detection and assembler sensitivity.<br>- Expanded discussion of computational trade-offs.<br>- Revised the Abstract and Discussion to better articulate MADRe's combined engineering and statistical contributions.<br>- Addressed all editorial comments, incorporated relevant recent GigaScience publications, and updated references to final published versions where applicable.<br><br>A detailed point-by-point response is provided in the accompanying document.<br>We appreciate your consideration and look forward to your feedback.<br><br>Sincerely,<br>Josipa Lipovac<br>Laboratory for Bioinformatics and Computational Biology, Faculty of Electrical Engineering and Computing, University of Zagreb, Zagreb, Croatia<br>josipa.lipovac@fer.unizg.hr<br>(on behalf of all co-authors)<br><br><br>We thank the reviewers for their constructive and valuable feedback on our manuscript. Below, we provide detailed responses to each of the raised concerns. Manuscript changes corresponding to the comments are indicated by line numbers. All modifications in the manuscript are highlighted in yellow.<br><br>---<br><br>1) "However, we observed a limitation when Centrifuger cannot confidently assign a read to a specific reference sequence (for example, when multiple chromosomes belong to the same strain). In such cases, it often classifies the read under the NCBI strain-level taxid, which in some instances is identical to the species-level taxid. This makes it impossible to directly and fairly compare those classifications with other tools that operate at the sequence level."<br><br>Although I agree this issue may not substantially affect the overall conclusions, the current handling of strain-level evaluation for Centrifuger is not sufficiently rigorous. The underlying problem is that Centrifuger (and Kraken2) rely on nodes.dmp and names.dmp, where the lowest taxonomic rank is often species or subspecies. As a result, these tools cannot report strain-level abundances directly in their standard output.<br>A more appropriate solution would be to assign custom, unique strain-level taxIDs for all reference genomes, allowing proper classification at the strain level. This approach has been discussed in https://github.com/mourisl/centrifuger/issues/18 and

Powered by Editorial Manager® and ProduXion Manager® from Aries Systems Corporation

https://github.com/jenniferlu717/Bracken/issues/113. Additionally, Centrifuger has an extra program, centrifuger-quant, that uses the EM algorithm to estimate abundance. The read assignment results produced by Centrifuger do not apply the EM algorithm.

→ answer:
We thank the reviewer for raising this important point regarding strain-level evaluation for Centrifuger (and similarly Kraken2). We agree that tools relying on the NCBI taxonomy hierarchy can face challenges when performing strain-level classification, particularly when strain-level taxonomic identifiers are unavailable or identical to species-level taxids.

In our study, we used reference databases that include strain-level taxonomy whenever such information is available. However, as noted by the reviewer and discussed previously in the literature, many recently submitted genome assemblies do not have unique strain-level taxids and are instead assigned only species- or subspecies-level identifiers (see, e.g., NCBI taxonomy limitations described in [1]).

To enable a rigorous and fair strain-level evaluation wherever possible, we designed our medium-sized simulated datasets using organisms that do possess unique strain-level taxids. This allowed us to directly evaluate strain-level classification performance for Kraken2 and Centrifuger under conditions where such evaluation is well-defined. For more complex datasets, where unique strain-level taxids are not available, we explicitly state in the manuscript (lines 1074-1085) that this ambiguity affects only a small fraction of reads. Importantly, even taking this into account, MADRe consistently demonstrates improved behavior compared to other methods.

The reviewer suggests assigning custom strain-level taxids for all reference genomes. While this is a valid approach and has been discussed in the community (e.g., Centrifuger and Bracken issue trackers), it would require modifying the standard NCBI taxonomy and rebuilding databases in a non-standard manner. Our goal in this work was to benchmark MADRe under realistic and widely used settings that reflect how these tools are typically applied in practice, rather than introducing custom taxonomic schemes that could limit reproducibility or comparability.

We additionally evaluated the impact of abundance re-estimation using the centrifuger-quant module on the medium-sized simulated datasets (Supplementary tables ST5-7). We observed only minimal changes in estimated read counts, generally in a favorable direction. However, centrifuger-quant reports abundance estimates rather than read-level classifications, which is central to MADRe's objective (lines: 666-673).

2) In the similarity experiment, some strains exhibit extremely high similarity, which makes proportional read distribution practically impossible for MADRe. To better characterize the performance limits of MADRe for accurate strain classification and abundance estimation, I recommend including additional simple synthetic mixtures at different combinations of similarity and coverage depth.

→ answer:
We agree that extremely high strain similarity represents a challenging scenario for any strain-level classification method. The high-similarity experiment included in the manuscript was intentionally designed as a stress test to probe the behavior of MADRe under such rigorous conditions. This experiment was added in response to a previous reviewer request, with the explicit goal of illustrating how MADRe behaves when strains are nearly indistinguishable at the sequence level. As anticipated, MADRe collapsed reads toward the nearest highly similar reference, and the results confirmed this behavior. Importantly, alternative approaches exhibited comparable limitations in this setting, although they resolved ambiguity in different ways (lines: 783-791; Supplementary File: Similar Strains Experiment; Supplementary Table ST23).
Rather than aiming to demonstrate optimal performance under extreme similarity, the purpose of this experiment was to provide transparency and interpretability - to clarify what MADRe's outputs represent when strain similarity exceeds the resolution supported by the data. This information is valuable for downstream analyses, where abundance estimates must be interpreted in the presence of nearly identical strains.
To further characterize the performance limits of MADRe across combinations of strain similarity and coverage depth, and to directly address the final part of the reviewer's

question, we conducted additional controlled synthetic experiments using reads simulated by Badread. We simulated four sets of mixtures, each containing three strains of varying similarity from E. coli, P. aeruginosa, S. aureus, and L. monocytogenes. These species were chosen following the Strainy study design [2] and because they are extensively represented in reference databases with many closely related strains, providing a realistic and challenging setting for strain-level discrimination. In each dataset, two additional strains from the sim_small dataset (taxid 446660) were included to introduce some inter-species context. For each species, the coverage of one strain was varied (20x, 10x, 5x, and 3x) to evaluate performance across coverage levels. The full dataset composition, ANI scores, and classification results are provided in Supplementary materials (Supplementary File: Coverage and Similarity Experiment; Supplementary Table ST24).
These experiments demonstrate that coverage depth does not substantially affect the detection of the correct strain, rather, performance is primarily influenced by the presence of multiple highly similar strains in the reference database. When expected strains were not recovered, this was primarily associated with extremely high similarity (>99.9% ANI) to another genome in the database, which prevented reliable discrimination. In such scenarios, MADRe consistently assigned reads to one of the highly similar strains, reflecting intrinsic resolution limits imposed by near-identical genomes rather than instability of the method. Together, these results confirm that MADRe remains robust across varying similarity and coverage conditions.
All other datasets and experiments in the study, including medium and large simulated datasets, Zymo mock communities, real metagenomic samples, and the additional synthetic mixtures described above, reflect realistic biological scenarios and demonstrate the typical performance of MADRe in practical applications. In those datasets, strains that were not recovered were often low-abundance, and in most of the cases these strains also exhibited very high similarity to another, typically more abundant reference genome. This indicates that the principal limiting factor is sequence similarity rather than coverage alone. Additionally, in all these experiments, MADRe was systematically benchmarked against state-of-the-art tools, providing a consistent benchmark across varying similarity and coverage conditions. In some complex communities, the detection of certain lower-abundance strains was affected. However, this mainly results from the presence of highly similar strains in both the dataset and the reference database. Despite this, MADRe consistently achieves competitive performance with a favorable balance between precision and recall.

We have revised the Discussion to explicitly address these conclusions (lines: 773-791).

3) Because long reads vary widely in length, read counts alone can be misleading. I strongly encourage reporting strain abundances rather than raw read counts, as abundances are more relevant for downstream applications.

→ answer:
We strongly agree with the reviewer that strain abundance estimates are generally more informative than raw read counts for downstream biological analyses, particularly when read lengths vary widely, as is common in long-read sequencing data.
For this reason, MADRe explicitly reports strain-level abundance estimates as one of its standard outputs. The abundance calculation procedure is described in detail in Section 5.2.1 (Abundance calculation).
In the experimental evaluation, we report classification results primarily in terms of read counts. This choice was made for clarity and comparability: all methods were evaluated on identical datasets containing the same set of reads, and under these conditions, read-count-based metrics and abundance-based metrics lead to consistent conclusions. Using read counts allows a more direct interpretation of classification correctness and simplifies comparison across tools without introducing additional normalization layers.

4) Finally, the authors should clarify whether MADRe's limitations in detecting low-abundance strains (referring more to low coverage) is entirely determined by the performance of the assembly tool, or whether additional factors influence this limitation.

→ answer:
We thank the reviewer for raising this point and agree that this should be discussed

Powered by Editorial Manager® and ProduXion Manager® from Aries Systems Corporation

more. MADRe’s sensitivity to low-abundance strains is influenced by two main factors. First, detection of low-abundance strains is fundamentally limited by the performance of the metagenome assembly step. If a strain is present at very low coverage, it may fail to assemble or may produce contigs that are too short, fragmented, or chimeric, containing insufficient strain-specific signal to support confident detection of the low-abundance strain. However, our controlled similarity-coverage experiments indicate that coverage alone is not the primary limiting factor within the evaluated range. Instead, challenges arise primarily when low-abundance strains coexist with highly similar and more abundant strains. In such cases, assembly may lead to partial signal collapse or insufficiently distinct contigs for confident separation during the database reduction step.In such cases, the strain cannot be detected by MADRe. Importantly, the use of assembly provides substantial benefits in reducing false positives and reducing the database, and similar low-coverage limitations exist for other strain-level methods that depend on sufficient genomic context.

Second, even when assembly enables detection of a low-abundance strain, additional challenges can arise during the read reassignment step when a highly similar (and high-abundance) strain is present. In such cases, if there are insufficient reads uniquely supporting the low-abundance strain, those reads may be reassigned to the dominant strain.

We have extended the Discussion to explicitly distinguish these two factors (lines: 749-791).

5) In Figure 4, please specify the sequencing technology used for sim_high. → ONT
"calculated using fastANI" →"calculated using fastANI".

-> answer:
The sequencing technology for sim_high (ONT) has now been specified in Figure 4, and the phrasing has been corrected to “calculated using fastANI.”

Reviewer #2: This manuscript presents MADRe, a modular pipeline for strain-level metagenomic classification from long-read data, emphasizing an assembly-driven database reduction strategy coupled with probabilistic reassignment. The work is methodologically sound and well aligned with the scope of GigaScience. However, the study can be benefit from the following revisions:

1) the study's main contribution is engineering and integration, rather than a fundamentally new statistical model. The authors thus should explicitly mention this in the Abstract as well as the Discussion part.

→ answer:
We thank the reviewer for this comment. We agree that a substantial contribution of MADRe lies in the careful engineering and integration of multiple components into a modular and scalable pipeline, which is essential for achieving robust strain-level classification in practice.
However, we would like to clarify that MADRe is not purely an engineering contribution. In particular, the database reduction strategy based on contig-to-reference reassignment using an expectation-maximization framework, as well as the subsequent probabilistic read reassignment using mapping profiles and operating on a reduced reference set, introduce methodological innovations that go beyond straightforward tool integration. These components define a new statistical formulation. To address the reviewer’s suggestion, we now explicitly state in the Abstract and Discussion (lines: 614-618) that MADRe’s contribution combines system-level engineering with novel statistical modeling choices, while emphasizing how these methodological components enable improved strain-level resolution.

2) although comparisons are reasonable, the manuscript could do more to clarify how MADRe compares against state-of-the-art strain-resolved tools under identical parameter tuning, and whether performance gains are consistent across different strain divergence levels.

→ answer:
We thank the reviewer for this comment and agree that clarity regarding comparison

settings is important.
All tools included in the benchmarking were executed using default or recommended parameters, without dataset-specific tuning, as described in the Methods section. This ensures a fair and consistent comparison across methods.
We note that there exist strain-resolved tools that assume prior knowledge of species identity and operate within a single-species context. As explicitly stated in the manuscript, such methods were not included in the comparison because MADRe is designed for realistic metagenomic scenarios where species composition is unknown and multiple species may be present simultaneously. Including single-species-restricted methods would therefore not provide a fair or meaningful comparison for the problem setting addressed in this study.
The evaluated comparative datasets used in this study span a broad range of strain divergence levels, including medium and large simulated datasets with moderate divergence, a dedicated high-similarity stress test, Zymo mock communities, and real metagenomic samples. Across these settings, MADRe demonstrates consistent performance trends relative to state-of-the-art tools.
To further characterize the performance limits of MADRe across combinations of strain similarity and coverage depth, we conducted additional controlled synthetic experiments (Supplementary File: Coverage and Similarity Experiment; Supplementary Table ST24), as noted in one of the previous responses. In these datasets, we simulated mixtures of three strains with varying ANI levels from E. coli, P. aeruginosa, S. aureus, and L. monocytogenes, and systematically varied the coverage of one strain (20x, 10x, 5x, and 3x). These experiments were designed to probe the sensitivity of MADRe under controlled divergence and abundance conditions. As stated in the answer to the previous reviewer, we demonstrated that coverage depth does not substantially affect the detection of the correct strain, rather, performance is primarily influenced by the presence of multiple highly similar strains in the reference database. When expected strains were not recovered, this was primarily associated with extremely high similarity (>99.9% ANI) to another genome in the database, which prevented reliable discrimination.
We have revised the Discussion to explicitly address these conclusions (lines: 773-791).
We trust that this expanded analysis addresses the reviewer's comment. If further clarification of the requested scenarios is needed, we would be grateful for additional guidance.

3) when comparing with existing tools, improvements appear primarily in precision, while recall trade-offs are less emphasized. The authors should explicitly discuss precision-recall trade-offs and clarify in which biological scenarios MADRe is most advantageous.

→ answer:
We agree with the reviewer that precision-recall trade-offs should be discussed more explicitly. In this study, we intentionally emphasize precision because MADRe is designed to reduce false-positive detections, which are a major challenge in strain-level metagenomic classification, particularly when using large and redundant reference databases.
At the genome identification level, a strain is considered detected if at least one read is classified to it. Under this definition, MADRe exhibits lower recall but higher precision compared to other approaches (e.g., results in ST20). This behavior reflects MADRe's identification strategy, which reduces false-positive strain detections at the cost of missing some low-support strains or sequences.
However, when read-level classification is considered, the picture is different. Although some strains may be identified by other methods but not by MADRe, the number of reads originating from such strains is typically low. In addition, when a strain is not retained during database reduction, its reads are often reassigned to the most similar reference in the reduced database. In such cases, read-level recall may remain largely unaffected, while strain-level precision can be influenced by the degree of similarity among reference genomes. For this reason, we placed greater emphasis on the precision metric. Furthermore, the number of false-negative reads is comparable between MADRe and other approaches, despite differences observed at the genome identification level.
In many evaluated datasets, the fraction of unclassified reads is low relative to the total number of reads, making genome-level recall less informative as a primary metric.

Powered by Editorial Manager® and ProduXion Manager® from Aries Systems Corporation

Nevertheless, we report recall, precision, and F1 scores for all experiments, with detailed results provided in the Supplementary Tables (ST8 and ST15). Notably, for large simulated datasets, MADRe achieves comparable or improved recall while simultaneously improving precision, which is reflected in competitive or higher F1 scores.
We have revised the Discussion to explicitly clarify this precision-recall trade-off and to highlight that MADRe is particularly advantageous in applications where precision is prioritized, such as settings in which false-positive strain detection carries greater consequences than missing extremely low-abundance organisms (lines: 760-772).

4) While database reduction is presented as efficient, the computational cost of assembly plus EM iterations is not deeply analyzed. The authors should include a concise runtime/memory comparison or at least a qualitative discussion of computational trade-offs.

→ answer:
We thank the reviewer for this comment. A detailed analysis of runtime and memory usage is already provided in the Supplementary Table (ST22), which reports CPU time and peak memory consumption for all major steps of the MADRe pipeline across two representative datasets, including database reduction.
The database reduction stage comprises: (i) metagenome assembly, (ii) mapping assembled contigs to the full reference database, (iii) the HairSplitter step used to detect collapsed strain signal, and (iv) the reference reduction algorithm itself. Among these, the assembly step is the most computationally demanding, particularly for large datasets. In contrast, contig-to-database mapping and the HairSplitter-based processing are substantially less resource-intensive than mapping all reads to a large reference database, and the reference reduction algorithm itself has minimal computational overhead.
We have clarified this computational trade-off in the revised manuscript (lines:645-655).

5) The approach implicitly assumes that metagenome assembly is sufficiently accurate and representative. However, in highly complex or low-coverage samples, assembly could be fragmented or biased. The authors should add a clearer discussion on the sensitivity to assembler choice and parameters.

→ answer:
We thank the reviewer for this comment and agree that metagenome assembly quality can influence strain detection, particularly in highly complex or low-coverage samples. MADRe is designed as a modular pipeline, allowing different assemblers and parameter settings to be used without modifying the core workflow. To assess sensitivity to assembler choice, we evaluated MADRe on large simulated datasets using Myloasm instead of metaFlye and metaMDBG. Overall performance trends were comparable, indicating that MADRe is not strongly dependent on a specific assembler. We observed complementary behavior, with Myloasm performing better for low-abundance strains and metaFlye performing slightly better for highly abundant strains. Based on these results, Myloasm is included as an optional component within the MADRe pipeline.
We note that strain-level assemblers were not considered, as they typically produce shorter and more fragmented contigs. MADRe's database reduction algorithm benefits from longer genomic context.
Regarding parameter sensitivity, all assemblers were executed using default or recommended settings. While different parameter choices may influence assembly quality, exploring extensive parameter tuning would substantially expand the scope of this study. We clarify this in the revised Discussion (lines:812-827).

REFERENCES:

[1] Federhen, Scott, et al. "Toward richer metadata for microbial sequences: replacing strain-level NCBI taxonomy taxids with BioProject, BioSample and Assembly records." Standards in genomic sciences 9.3 (2014): 1275-1277.
[2] Kazantseva, Ekaterina, et al. "Strainy: phasing and assembly of strain haplotypes from long-read metagenome sequencing." Nature Methods 21.11 (2024): 2034-2043.

Powered by Editorial Manager® and ProduXion Manager® from Aries Systems Corporation

	Editor Comments:<br><br>GigaScience has also published a number of relevant papers on strain-level metagenomic classification, machine learning-based host prediction, and pipeline development for microbiome analysis. Citing these papers would help contextualize your methodological contributions and strengthen the discussion of your results within the current literature landscape.<br><br>1. Yang Y, Dufault-Thompson K, Yan W, Cai T, Xie L, Jiang X. Large-scale genomic survey with deep learning-based method reveals strain-level phage specificity determinants. GigaScience. 2024;13:giae017. https://doi.org/10.1093/gigascience/giae017<br><br>2. Chen G, Jiang J, Sun Y. RNAVirHost: a machine learning–based method for predicting hosts of RNA viruses through viral genomes. GigaScience. 2024;13:giae059. https://doi.org/10.1093/gigascience/giae059<br><br>3. Arikan M, Muth T. gNOMO2: a comprehensive and modular pipeline for integrated multi-omics analyses of microbiomes. GigaScience. 2024;13:giae038. https://doi.org/10.1093/gigascience/giae038<br><br>4. Roach MJ, Beecroft SJ, Mihindukulasuriya KA, et al. Hecatomb: an integrated software platform for viral metagenomics. GigaScience. 2024;13:giae020. https://doi.org/10.1093/gigascience/giae020<br><br>5. Gao Y, Luo H, Lyu H, et al. Benchmarking short-read metagenomics tools for removing host contamination. GigaScience. 2025;14:giaf004. https://doi.org/10.1093/gigascience/giaf004<br><br>-> answer:<br>We thank the Editor for this helpful suggestion and for highlighting relevant recent publications in GigaScience. We agree that situating MADRe within the broader landscape of strain-level metagenomic and microbiome analysis methods strengthens the manuscript.<br>Accordingly, we have incorporated citations to Yang et al. (2024), Arikan and Muth (2024), and Gao et al. (2025), as these studies are directly relevant to strain-level genomic analysis, machine learning approaches applied to microbial genomics, and modular/benchmarking frameworks in metagenomic workflows.<br>We also carefully considered the studies by Chen et al. (2024) and Roach et al. (2024). However, these works focus primarily on RNA virus host prediction and viral metagenomics platforms, respectively. We believe that these studies fall outside the direct methodological scope of our work. For this reason, we did not incorporate them into the discussion to avoid overstretching the conceptual framing.<br>We appreciate the Editor's guidance in ensuring comprehensive contextualization within the journal's recent literature.<br><br>In addition, please ensure that all references are to the final, peer-reviewed published versions of articles. If you have cited any preprints (e.g., from bioRxiv) that have now been published in journals, please update those entries accordingly. This is an important step for the long-term archival stability and credibility of the reference list.<br><br>We have reviewed the entire reference list and updated all entries to cite the final peer-reviewed published versions where available, replacing preprint citations accordingly.
**Additional Information:**	
**Question**	**Response**
Are you submitting this manuscript to a	No

Powered by Editorial Manager® and ProduXion Manager® from Aries Systems Corporation

special series or article collection?	
**Experimental design and statistics**<br><br>Full details of the experimental design and statistical methods used should be given in the Methods section, as detailed in our Minimum Standards Reporting Checklist. Information essential to interpreting the data presented should be made available in the figure legends.<br><br>Have you included all the information requested in your manuscript?	Yes
**Resources**<br><br>A description of all resources used, including antibodies, cell lines, animals and software tools, with enough information to allow them to be uniquely identified, should be included in the Methods section. Authors are strongly encouraged to cite Research Resource Identifiers (RRIDs) for antibodies, model organisms and tools, where possible.<br><br>Have you included the information requested as detailed in our Minimum Standards Reporting Checklist?	Yes
**Availability of data and materials**<br><br>All datasets and code on which the conclusions of the paper rely must be either included in your submission or deposited in publicly available repositories (where available and ethically appropriate), referencing such data using a unique identifier in the references and in the "Availability of Data and Materials" section of your manuscript.<br><br>Have you have met the above requirement as detailed in our Minimum Standards Reporting Checklist?	Yes

GigaScience has policies and guidelines in place for the use of generative AI-writing tools such as ChatGPT. If you have used such writing tools to assist with writing the manuscript this must be declared and cited in the text. Authors should not list AI-writing tools and other AI-assisted technologies as an author or co-author and should acknowledge that they are fully responsible for text generated or refined by AI-writing tools.<p><br><br>A summary of use (particularly in the introduction or among methods) needs to be included at the end of the paper, and the outputs should also be included as a supplementary file hosted in GigaDB or other open repositories. Please read our guidelines for more information. <p><br><br>By submitting to GigaScience, you are aware of the journal's AI-writing tools policy, and if you have declared use of such tools below, you have acknowledged this where appropriate in your manuscript and have made a summary of use and outputs available. <p><br><b>AI-assisted writing tools have been used in the preparation of this manuscript?	No

Click here to access/download;LaTeX - Main Document (TeX file);MADRe_main.tex

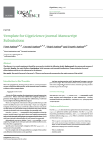

GigaScience, 2026, 1–19

doi: xx.xxxx/xxxx
Manuscript in Preparation
Technical Note

TECHNICAL NOTE

MADRe: Strain-Level Metagenomic Classification Through Assembly-Driven Database Reduction

Josipa Lipovac[1,*], Mile Šikić[1,2], Riccardo Vicedomini[3,†] and Krešimir Križanović[2,*†]

[1]Laboratory for Bioinformatics and Computational Biology, Faculty of Electrical Engineering and Computing, University of Zagreb, Zagreb, Croatia and [2]Laboratory of AI in Genomics, Genome Institute of Singapore, A*STAR, Singapore, Singapore and [3]Univ Rennes, CNRS, Inria, IRISA - UMR 6074, F-35000 Rennes, France

[*]josipa.lipovac@fer.unizg.hr; kresimir.krizanovic@fer.unizg.hr
[†]Contributed equally.

Abstract

Strain-level metagenomic classification is essential for understanding microbial diversity and functional potential, yet remains challenging, particularly when sample composition is unknown and reference databases are large and redundant. Here we present MADRe, a modular and scalable pipeline for long-read strain-level metagenomic classification based on **M**etagenome **A**ssembly-Driven **D**atabase **Re**duction. Beyond system-level integration, MADRe introduces statistical strategies that leverage assembly-derived genomic context to guide database reduction and probabilistic read reassignment. Specifically, it combines long-read metagenome assembly, contig-to-reference reassignment using an expectation–maximization framework for reference reduction, and probabilistic read mapping reassignment on a reduced database to achieve sensitive and precise strain-level classification. We extensively evaluated MADRe on simulated datasets, mock communities, and a real anaerobic digester sludge metagenome. Across diverse similarity and coverage conditions, MADRe consistently improves precision by reducing false-positive strain detections. MADRe's design allows users to apply either the database reduction or read classification step individually. Using only the read classification step shows results on par with other tested tools. MADRe is open source and publicly available at https://github.com/lbcb-sci/MADRe.

Key words: metagenomics; strain-level; metagenomic classification; database reduction

Background

Metagenomics enables the study of genetic material from complex microbial communities found in environments such as human gut, soil, or marine ecosystems. It provides a comprehensive view of microbial diversity and interactions within these environments [1, 2]. Modern microbiome studies increasingly rely on modular and multi-omics analysis pipelines to integrate taxonomic and functional signals across data types [3]. Despite these advances, a central challenge in metagenomic analysis is the accurate identification of organisms present in a sample, typically performed by comparing sequencing reads to reference genome databases[4].

A wide range of metagenomic classification tools have been developed, which can be broadly categorized into marker-based, DNA-to-protein and DNA-to-DNA approaches, as described in [5]. Marker-based tools, such as MetaPhlAn [6, 7], StrainPhlAn, mOTUs [8], and Melon [9], classify taxa using conserved, clade-specific marker genes. In addition to marker-based methods, SNV-based profilers (e.g., metaSNV [10] and InStrain [11], which combines both approaches) represent important strategies for strain detection and population tracking. However, most of these approaches are optimized for short-read data and rely on predefined marker sets or variant catalogs, which may not fully capture genomic diversity in complex or underrepresented microbial communities.

Compiled on: March 16, 2026.
Draft manuscript prepared by the author.

Key Points

- MADRe implements a two-step strategy for strain-level classification: it first identifies candidate strains via assembly-to-database mapping with EM-based reassignment, then classifies reads by mapping to a reduced database via probabilistic reassignment.
- Despite incorporating assembly, MADRe significantly reduces runtime and memory usage compared to mapping all reads to the full reference database.
- MADRe enables the use of large, diverse reference databases without prior knowledge of sample content, focusing classification on confidently assembled strains and substantially reducing false positives while maintaining high strain-level resolution.

DNA-to-protein tools, including Kaiju [12], DIAMOND [13], MMseqs2 [14], and MEGAN-LR [15] translate reads into amino acid sequences before aligning them to protein databases. DNA-to-DNA tools compare reads directly against genomic sequences and are commonly divided into k-mer-based and mapping-based tools [16]. K-mer-based tools such as Kraken2 [17], KrakenUniq [18], Bracken [19], Centrifuge [20], Centrifuger [21], CLARK/CLARK-S [22, 23], Ganon [24, 25], Taxor [26], and Sylph [27] are known for their speed and scalability to large databases, but often trade precision for speed. In contrast, mapping-based tools such as MetaMaps [28], PathoScope2 [29, 30], EMU [31] and MORA [32], which rely on read alignments and reassignment algorithms, offer higher precision at a greater computational cost.

Although k-mer-based tools, especially Kraken2 or Sylph, perform well at the species level, strain-level classification becomes increasingly challenging when sequences originate from closely related genomes [33]. However, resolving strain-level diversity is essential, as even closely related strains can exhibit substantial differences in gene content and function, with implications for microbial ecology, pathogenesis, and treatment outcomes [34, 35, 36, 37, 38, 39].

While most existing tools are optimized for short reads due to their low cost and high accuracy, long-read sequencing technologies such as Oxford Nanopore and PacBio HiFi are rapidly improving. Longer read lengths provide advantages for genome assembly, structural-variant detection, and improved strain-level resolution.

Several short-read-based tools are designed for strain-level classification within a single species, such as StrainGE [40], StrainEST [41], and StrainSeeker [42], as well as the long-read-based ORI [43]. Other tools, including PanTax [5], MetaMaps [28], Centrifuge [20], Centrifuger [21], PathoScope2 [29, 30], and MORA [32], are suitable for more complex, multi-species datasets and support short- and long-read strain-level metagenomic classification. PanTax is a pangenome-based approach that, while supporting multi-species datasets, faces scalability limitations when applied to very large reference databases. MetaMaps is a mapping-based tool capable of high-resolution classification but is known to be extremely computationally demanding [16]. Centrifuge is a k-mer-based tool designed to perform strain-level classification, but like PanTax, it encounters limitations when constructing indexes for very large reference databases. However, its successor, Centrifuger, introduces improved compression and indexing strategies that enable efficient classification across large-scale genome databases. PathoScope2 is an older tool that is no longer maintained and cannot be reliably executed due to outdated dependencies and software incompatibilities. Originally developed for strain-level classification of short reads, it is based on an expectation-maximization (EM) algorithm for read reassignment [44]. As part of the MORA study, the authors introduced a continuation of PathoScope2, referred to as AugPatho, which includes a modified version of the original algorithm adapted for use with long reads [32]. MORA extends this approach by combining the EM algorithm from Agamemnon [45] with a read reassignment strategy based on the Weapon-Target Assignment (WTA) problem. According to its authors, MORA represents the current state-of-the-art in mapping-based long-read metagenomic classification.

Beyond algorithmic differences among tools, practical performance in metagenomic workflows can also be influenced by upstream preprocessing decisions and dataset composition, which can substantially affect downstream taxonomic inference [46].

Reference databases often contain multiple assemblies of the same strain and typically lack consistent organization. To address this, some strain-level classification tools perform database preclustering according to Average Nucleotide Identity (ANI) scores. Previous studies have shown that there is no universal ANI threshold for defining strains [40, 41, 47, 48, 34]. Setting the threshold too high may erroneously separate assemblies of the same strain, whereas setting a threshold too low may incorrectly group different strains together.

Using large and diverse databases is important for accurate strain-level classification [49], but it also makes the analysis much more demanding to run. MetaAlign [50] uses containment MinHash [51] to reduce the reference database prior to alignment, improving runtime while maintaining high species-level precision. However, it is primarily designed for short reads, and strain-level resolution is not its main focus.

In this work, we introduce MADRe, a pipeline for long-read, strain-level metagenomic classification enhanced with Metagenome Assembly-Driven Database Reduction, consisting of two main phases: database reduction and read classification. In the database reduction step, MADRe combines long-read assembly with an EM algorithm that assigns assembled contigs to one or more references, reducing the reference database.

In the second, read classification step, MADRe performs mappings-based read reassignment. It resolves ambiguous read mappings by assigning each read to the most likely reference, based on mapping scores and probabilistic support.

We conducted an extensive evaluation of MADRe using simulated datasets, Zymo mock communities, and a real anaerobic digester sludge metagenome. The results demonstrate that MADRe achieves high precision and strain-level resolution while maintaining lower memory usage and runtime compared to existing tools. Additionally, the two steps of the MADRe pipeline can be run independently, and our results show that the read classification module (MADRe_RC) alone performs competitively. MADRe's approach enables the use of large, diverse reference databases spanning multiple taxonomic levels, making it well-suited for scenarios where no prior knowledge about the sample is available. Using assembled contigs to detect potentially present strains, MADRe focuses on confidently represented organisms. As a result, compared to state-of-the-art tools, it significantly reduces the number of false positives while maintaining high resolution strain-level classification.

Results

MADRe - method overview

The MADRe pipeline is designed for strain-level metagenomic classification, particularly in scenarios where prior knowledge of the sample composition is not available. Its primary goal is to enable accurate strain identification while reliably distinguishing truly abundant strains from false positives.

As illustrated in Figure 1, the MADRe pipeline consists of two main steps: database reduction and read classification. It takes as input a large bacterial reference database and raw long metagenomic reads, and produces two main outputs: a read classification file and a reference abundance file.

In the database reduction step, long reads are first assembled, and the resulting contigs are mapped to the large reference database. MADRe employs HairSplitter [52] to estimate the number of collapsed strains represented by each contig. Using both contig-to-reference mappings and the collapsed strain estimates, an EM algorithm is applied, followed by additional read-reassignment steps. This process generates a reduced reference database that highlights strains likely present in the sample. Although this reduction effectively narrows the search space, it may still include false positives introduced during the assembly process. These could be further filtered during the read classification step.

In the second step, reads are mapped to the reduced reference database, and ambiguous mappings are resolved through a reassignment procedure that leverages mapping scores and probabilistic support.

Both steps of the pipeline can be run independently, so when prior knowledge about the sample exists or when a reduced reference set is already available, the read classification step can be used on its own.

Benchmarking details

We benchmarked the MADRe pipeline against state-of-the-art tools developed for the same purpose: handling large reference databases while enabling strain-level classification. These tools include MORA and AugPatho (PathoScope2), for which we evaluated both of its key modules, PathoID and PathoReport. In some of the experiments, we also evaluated k-mer based tool Kraken2, one of the most widely used metagenomic classification tools, which is often considered the standard for species-level classification. Although Kraken2 is capable of assigning reads at the strain level, its evaluation is complicated by the use of taxonomic identifiers (taxIDs) that may refer to either species or strain ranks. Despite its popularity, recent work has shown that Sylph achieves superior performance for species-level abundance estimation, reporting fewer false positives. However, since Sylph is primarily designed for abundance profiling rather than direct read classification, we did not include it in our benchmarking, which focuses explicitly on classification accuracy. Additionally, we included MADRe_RC, a variant of MADRe that performs only the second step (*i.e.*, read classification) without prior database reduction.

We did not include MetaMaps, PanTax, or Centrifuge in our benchmarking analysis. In the case of MetaMaps, previous studies have reported crashes when attempting to build an index for the full Genome Taxonomy Database (GTDB), highlighting its scalability limitations [26, 32]. Similarly, our attempts to construct the same reference database for PanTax and Centrifuge, used successfully with other benchmarking tools, also failed due to crashes during the indexing process. Instead, we evaluated Centrifuger, a recent successor of Centrifuge that introduces improved compression and indexing strategies, enabling efficient classification on large-scale genome databases.

By default, MADRe employs metaFlye [53] for assembling Oxford Nanopore (ONT) reads and metaMDBG [54] for assembling PacBio HiFi reads. To assess the effect of different assembly strategies on database reduction, we also performed additional experiments using Myloasm [55], a recently developed assembler showing promising performance on metagenomic datasets.

All commands used to run the benchmarking tools are available in the Supplementary File (Tools versions and commands).

Datasets

As part of the benchmarking process, we evaluated the mentioned tools on simulated metagenome datasets, Zymo mock communities, and a real anareobic digester sludge metagenome.

For medium-sized simulated datasets, we selected a smaller subset of genomes representing species commonly found in the human gut microbiome. Reference genomes were required to be labeled as "complete" or "chromosome" in NCBI, and to have a strain-level taxID distinct from their species-level taxID. This criterion ensured that Kraken2 could be included in the evaluation.

Using Badread tool [56] we simulated three different metagenomic datasets:

i. **sim_small (4 strains)** – This dataset includes four different strain references: two strains from *Adlercreutzia equolifaciens* and two from *Streptococcus anginosus*, with varying relative abundances.
ii. **sim_medium (15 strains)** – This dataset contains 15 strain references distributed across five bacterial species (*i.e.*, *Helicobacter pylori*, *Cutibacterium acnes*, *Streptococcus intermedius*, *Streptococcus mutans*, *Lactococcus lactis*), with each species represented by three strains. At species level the abundances are different while strains of one species are equally abundant.
iii. **sim_expanded (30 strains)** – An extension of the sim_medium dataset, incorporating 15 additional strains from distinct species and maintaining variable abundance levels across species. Newly added species are listed in Supplementary Table ST1.

Exact genome information including accession numbers, strain and species taxIDs, genome lengths, genome coverages, ANI values (calculated usin fastANI [47], and number of simulated reads can be found in the Supplementary Tables (ST1-ST4).

Although these datasets can be used to assess MADRe's performance, they remain relatively simple and do not fully reflect the complexity of real metagenomic samples. Therefore, we expanded our benchmarking to include four additional simulated datasets originally used in the PanTax study [5] and we called them large-sized simulated datasets. Three of these datasets each contain 60 genomes coming from 30 species, simulated using ONT R9.4.1, ONT R10.4.1, and PacBio HiFi error profiles, respectively. These datasets were obtained directly from the PanTax Zenodo repository [57]. In addition, we generated a fourth, large-scale dataset comprising 1000 genomes from over 300 species, inspired by the CAMI challenge design. As simulated reads for this dataset were not available due to its size, we used the published reference genomes and expected abundances to simulate reads with the Badread tool. For these datasets, we additionally present distributions of ANI scores (calculated using fastANI), illustrating how many genome pairs exceed predefined ANI thresholds, as shown in Supplementary Table ST10.

We also tested MADRe using three Zymo mock communities: D6322 (ONT), D6331 (ONT) [58], and D6331 (PacBio HiFi) [59]. We included both D6311 ONT and D6311 PacBio HiFi datasets to demonstrate the pipeline's capability across different sequencing technologies.

To evaluate performance on a complex real-world dataset, we analyzed an anaerobic digester sludge metagenome dataset sequenced using ONT R10.4.1. reads [60]. This real metagenome represents the type of scenario for which MADRe is designed, where

a highly diverse sample is analyzed without prior knowledge of its taxonomic composition.

Database

To thoroughly evaluate strain-level classification and ensure sufficient taxonomic divergence for accurate strain-level detection, we used a database obtained via the Kraken2 interface by selecting the bacterial database. This database consists of 102,639 sequences, encompassing all RefSeq [61] complete bacterial genomes. The database was downloaded in December 2024. The exact command used for downloading the database is provided in the Supplementary File (Tools versions and commands).

For consistency, the same database was used across all tools and experiments.

Database Reduction

Table 1. Database reduction results. Comparison of MADRe's database reduction performance with two baseline models on simulated datasets using large database containing **102,639** sequences: baseline model 1 (BM1), which includes only the top-1 mapping for each contig, and baseline model 2 (BM2), which includes the top-3 mappings.

	Metric	sim_small	sim_medium	sim_expanded
number of genomes in dataset		4	15	30
**BM1**	# in reduced	7	34	68
	# missing strains	0	1	3
	# FP strains	3	19	38
	# FP species	1	0	6
**BM2**	# in reduced	14	107	234
	# missing strains	0	0	0
	# FP strains	10	92	204
	# FP species	5	11	78
**MADRe database reduction**	# in reduced	7	42	84
	# missing strains	0	0	0
	# FP strains	3	27	54
	# FP species	1	0	6

To assess the effectiveness of strain identification and database reduction, we evaluated the output of MADRe's database reduction step on simulated datasets by comparing it to two baseline models. More precisely, assembled contigs were mapped to the large reference database and the identification of organisms was carried out using the following strategies:

- Baseline Model 1 (BM1): For the reduced database, each contig's top reference genome, determined by the highest summarized harmonic mean mapping value (Methods, Equation 2), was included without performing any reassignment steps. BM1 represents the ideal reduction level under the assumption of a perfect assembly, where each contig corresponds to a single strain reference.
- Baseline Model 2 (BM2): For the reduced database, each contig's top three reference genomes, based on the highest summarized harmonic mean mapping values (Methods, Equation 2), were included without performing any reassignment steps. BM2 defines an upper bound on the number of references expected in the reduced database. Since our simulated metagenomes contain at most three strains per species, we assume that at most three strains could be collapsed into a single contig. This approach allows us to determine how many references should be retained in the reduced database to ensure that no true reference from the sample is missed.

The results, presented in Table 1, demonstrate that MADRe's database reduction achieves a high level of reduction while successfully retaining all expected strains. Additionally, when compared to BM2, MADRe reduces the number of false positive species, further highlighting its effectiveness.

Classification of medium-sized simulated datasets

For medium-sized simulated datasets we compared the classification performance of MADRe, MADRe_RC, MORA, AugPatho (in both PathoID and PathoReport modes), Kraken2, and Centrifuger. Since the exact source of each read is known, we define classification outcomes as follows: true positive (TP) if the read is classified under the expected strain (or expected cluster), true negative (TN) if the read is not classified and its Badread label is *random* or *junk*, false positive (FP) if the read is classified under the incorrect strain (or incorrect cluster), and false negative (FN) if the read is not classified but its label is different from *random* or *junk*.

We evaluated classification of simulated data with and without post-clustering. The post-clustering method described in the Methods section groups closely related strains based on read-to-reference mappings and assigns reads to clusters instead of individual strains. The same clustering approach was applied to all tools, utilizing read mappings to the full reference database. Kraken2's clustering results are not included, as taxID alone does not allow for an accurate evaluation of post-clustering performance.

The classification results of medium-sized simulated datasets (sim_small, sim_medium and sim_expanded) are presented in Figure 3 A., which shows the F1 scores for classification with and without post-clustering. The results demonstrate that both MADRe and MADRe_RC outperform all other approaches. Interestingly, on the sim_small dataset, which includes differently abundant strains of the same species, Kraken2 performs slightly better than Centrifuger, while Centrifuger achieves higher scores than MORA and AugPatho. For the remaining datasets, Centrifuger performs better than Kraken2 but worse than MORA and AugPatho. In the sim_expanded dataset (without clustering), MORA outperforms AugPatho's modes, but in all other cases, including all clustering scenarios, both AugPatho's modes perform significantly better than MORA. When post-clustering is applied, Centrifuger shows the lowest performance across all datasets, while post-clustering further improves AugPatho's results, bringing them close to MADRe_RC. Overall, MADRe_RC achieves performance comparable to MADRe, although this difference becomes more pronounced on more complex datasets. Figure 3B. shows the number of organisms identified by different tools on simulated datasets. An organism is considered identified if at least one read is classified under it. In all cases, there were no false negatives — all tools successfully identified the expected organisms. However, the number of additional (false positive) identifications varies. MADRe consistently reports significantly fewer false positives. For example, in the sim_small dataset, only 6 organisms were reported compared to the 4 expected, with 2 of them being extremely similar strains to those actually present in the sample. Additional metrics for organism-level identification, including TPs, FPs, FNs, accuracy, precision, recall, and F1 scores, are provided in Supplementary Table ST8, while classified read counts for each organism are listed in Supplementary Tables (ST5–ST7).

In addition, a Supplementary Figure S4 and Supplementary Table ST9 show Bray–Curtis (BC) distances [62] between the observed read count abundances and the ground-truth abundances, offering further insight into the similarity between the predicted and true community compositions. BC distance is one of the most commonly used distances to calculate the microbial abundance differences, and is described in the Methods section. From these results, it is

evident that MADRe achieves the closest match to the ground truth, while AugPatho ID reports the poorest scores among strain-level tools, including Centrifuger. Kraken2, although computationally efficient, shows the weakest overall performance.

Classification of large-sized simulated datasets

To further assess classification performance under more realistic metagenomic conditions, we used the simulated datasets from the *PanTax* study. We used the same evaluation procedure as the one considered for the medium-sized simulated datasets. In addition to benchmarking the standard MADRe pipeline, we also evaluated a variant in which Myloasm was used as the assembler during the database reduction step, in order to examine how different assembly approaches influence MADRe's performance. This variant was not tested on the sim_low R9.4.1 dataset, as Myloasm is not suitable for reads with that error profile.

Figure 4 presents four radar plots, each corresponding to one of the four large sized datasets, and showing F1 scores for all evaluated tools, both with and without post-clustering. (Note that post-clustering values for Kraken2 are zero, since this step was not performed for that tool.) For each tool, the best obtained F1 score is indicated in parentheses beneath its name. Across most datasets, MADRe achieved the highest scores, including its versions using different assemblers. In some cases, MADRe_RC slightly outperformed MADRe, particularly on the sim_high dataset. Detailed evaluation statistics are provided in Supplementary Table ST15. Exact read counts obtained from classifications are listed in Supplementary Tables (ST11–ST14), while Bray–Curtis (BC) distances between observed and expected read-count abundances are shown in Supplementary Figure S5 and Supplementary Table ST16.

Inspection of read-count abundances in the sim_high dataset revealed that several genomes were not detected during the database reduction step, leading to a modest decrease in MADRe's performance compared to MADRe_RC.

When comparing assembler performance, MADRe runs based on Myloasm assemblies achieved slightly higher F1 scores than those using metaFlye. A closer look at read-count abundances revealed that 24 genomes were detected exclusively with Myloasm and 11 exclusively with metaFlye. Among the 11 missed by Myloasm, 9 belonged to the more abundant half of the community, whereas among the 24 detected only by Myloasm, just one was highly abundant. This pattern suggests that Myloasm-based contigs perform better for low-abundance strains, while metaFlye contigs remain more reliable for highly abundant ones.

Another observation from the sim_high dataset is that, for several highly abundant strains, most benchmarking tools reported substantially lower read-count abundances than the ground truth. For example, reads originating from NZ_CP012672.1 were predominantly assigned to NZ_CP102233.1, a nearly identical genome (ANI = 99.998) annotated under a different species (Sorangium cellulosum vs. Sorangium sp. So ce836). Because clustering was performed only among strains within the same species, this near-duplicate across species boundaries could not be resolved even after clustering.

As shown in Figure 4, all tools achieved higher F1 scores on ONT R10 and PacBio HiFi datasets compared to ONT R9, reflecting the higher base accuracy of these sequencing platforms. Interestingly, for the ONT R10 dataset, MADRe using Myloasm performed slightly worse than the metaFlye version, whereas for PacBio HiFi reads, Myloasm yielded marginally better results than the metaMDBG-based variant.

Taken together, these results, including the analyses of BC distances, demonstrate that MADRe, in all assembler configurations, consistently outperforms the other evaluated tools on the large sized simulated datasets.

Classification of Zymo mock communities datasets

To evaluate MADRe on real sequencing data, we conducted experiments on three different Zymo mock community datasets: ONT Zymo D6322, which consists of eight organisms (seven bacterial species and one fungus), and both the ONT and HiFi versions of Zymo D6331, which contain 21 organisms, including two fungi and five different strains of *Escherichia coli*. The primary challenge in the Zymo D6331 dataset is the ability to distinguish between these closely related *E. coli* strains. In this analysis, we excluded fungal genomes, focusing solely on bacterial classifications.

For Zymo mock communities, exact reference genomes of the strains present in the sample are available, along with their theoretical relative abundances provided by ZymoBIOMICS [63]. We supplemented our database with the Zymo reference genomes, assigning them separate labels. However, we did not use provided theoretical abundances in our analysis, as they may deviate from the expected values due to variations in library preparation [9, 31]. Instead, we established ground-truth read classifications. We mapped all reads to the expected bacterial reference genomes using Minimap2 and assigned true labels based on the best hit. These assignments were also used to determine the relative abundances. However, this process was not straightforward for the five *Escherichia coli* strains, as their high similarity led to ambiguous mappings. To address this, we leveraged our clustering method (explained in *Similar strains clustering* section), which grouped these five strains into three clusters. Specifically, strains B766 and B3008 each formed separate clusters, while the remaining three strains were grouped into a single cluster, indicating that they were too similar to be reliably distinguished at the strain level. This clustering result aligns with previous findings from metagenome assembly procedures [64], where B766 and B3008 were successfully assembled, while the other three strains were not.

Benchmarking with Centrifuger and Kraken2 was not performed for this experiment, as their database construction procedures do not support the inclusion of references with custom labels, which is essential for this evaluation.

Using this information, we incorporated the clustering results into our ground-truth labeling: reads originating from the same cluster were assigned the same label, ensuring a more accurate classification.

To evaluate performance, we calculated the BC distances (eq.9) between the observed read count abundances and the ground-truth abundances, both with and without post-clustering.

Figure 5 depicts radar plots showing the BC distances for the zymo D6322 ONT, zymo D6331 ONT, and zymo D6331 HiFi datasets. Dotted lines indicate BC distances computed using only true positive classifications based on the ground truth. In the first plot, which reports results for the D6322 dataset, MADRe clearly outperforms all other tools. The second and third plots display BC distances for the D6331 ONT and HiFi datasets, respectively. For the ONT dataset, when considering all classified reads, MADRe achieves the lowest BC distance. When focusing only on true positives, MADRe and MADRe_RC show comparable performance, indicating that the majority of reads classified by these tools are correctly assigned. In contrast, MORA exhibits a notably higher BC distance when evaluated only on true positives, suggesting less precise classification. For the HiFi dataset, overall distances for all the tools are significantly lower. Both AugPatho modes achieve slightly lower BC distances compared to MADRe. In Supplementary Figure S6, we present the corresponding results obtained after post-classification clustering of similar strains. Interestingly, for the ONT datasets, BC distances increased for both AugPatho and MADRe following clustering. Although the increase is not substantial, the clustering step led to elevated abundance estimates, resulting in a higher number of both false positives and true positives. This trend was not observed for the HiFi dataset, where MADRe achieved the best performance after clustering.

Table 2. False positive (FP) species and strains detected by different tools on Zymo datasets. An organism is considered a false positive if at least one read is classified under it, but it is not in the true community.

	Tool	D6331 ONT	D6331 HiFi	D6322 ONT
**FP Species**	MADRe	5	5	6
	MADRe_RC	391	53	385
	MORA	639	162	517
	AugPatho ID	266	10	327
	AugPatho REP	249	20	260
**FP Strains**	MADRe	386	114	52
	MADRe_RC	3441	1189	6441
	MORA	6251	4010	10357
	AugPatho ID	2641	518	6133
	AugPatho REP	2316	889	4675

The exact read counts, used to calculate BC distance, are listed in the Supplementary Tables (ST17-ST19).

Table 2 presents the number of false-positive species and strain identifications. MADRe reports a significantly lower number of false positives at both levels compared to other tools. Supplementary Table ST20 provides a more detailed breakdown of the number of identifications. From this table, it is evident that MADRe's main limitation is the higher number of false negatives, primarily originating from low-abundance organisms that could not be detected using the assembly-based approach on which MADRe relies. This is further supported by the MADRe_RC results, where the number of false negatives is comparable to other tools. Nevertheless, MADRe consistently reports a substantially lower number of false positives. The table also includes AugPatho results from report outputs from both modes. These reports are generated after the final reassignment step and contain only abundance estimates. Consequently, they cannot be used directly for classification evaluation. While these reports show a significantly lower number of false positives, this reduction comes at the cost of a higher number of false negatives.

Classification of real anaerobic digester sludge metagenome

While Zymo mock communities represent real metagenomic data, they do not fully capture the complexity typically found in environmental or host-associated microbial communities. To better reflect realistic classification scenarios, we evaluated MADRe and the other competing tools on a real anaerobic digester sludge metagenome. As this dataset lacks ground truth, we focused on comparative analysis of classification outputs. All results presented here include post-clustering.

In this dataset, MADRe identified 1,320 reference strains (1,502 without clustering), while MADRe_RC reported 14,304 (19,067 without clustering), MORA 15,835 (23,516 without clustering), AugPatho ID 11,785 (16,488 without clustering), AugPatno REP 11,134 (14,604 without clustering) and Centrifuger 23,950 (28,450 without clustering). Out of 3,646,771 total reads, MADRe classified 575,052 (~ 16%), MADRe_RC 696,961 (~ 19%), MORA 696,839 (~ 19%), AugPatho ID 898,906 (~ 25%), AugPatho REP 737,714 (~ 20%) and Centrifuger 1,350,537 (~ 37%) reads.

Figure 6 illustrates percentile-normalized rank-abundance curves, highlighting differences in strain-level classification across the tools. The underlying read count abundance data used to generate this figure is provided in Supplementary Table ST21.

The curve for MADRe displays a consistent, moderately steep gradient throughout, with notable deviations at the beginning and end. The sharp rise at the beginning indicates the presence of a highly abundant strain, significantly more dominant than the others. This can be seen for the other tools as well. Toward the end, the curve drops sharply, likely reflecting false positives or low-confidence strain assignments. Compared to the other tools, MADRe shows a smoother and more gradual decline in the abundances of lower-ranked strains. In contrast, MADRe_RC, MORA, AugPatho and Centrifuger report a larger number of low-abundance strains, resulting in a more stepwise decline. The flat tail in their curves suggests that many strains are assigned near-zero abundances.

Figure 7 shows the relative abundances of the 20 most abundant strains reported by each tool, calculated relative to the total number of classified reads. We also generated an analogous visualization at the species level (Supplementary Figure S7), which additionally includes Kraken2 results. Among all tools, MADRe achieved the highest cumulative relative abundance for the top 20 strains, followed by AugPatho and MADRe_RC, while MORA and Centrifuger exhibited similar but substantially lower overall contributions from their top strains. Figure 7 highlights one notable strain-level discrepancy: the strain Paludibacter propionicigenes (accession number NC_022549.1, taxID 6135), which appeared among the top 20 only in AugPatho results. To investigate this discrepancy, we examined how reads classified as taxID 6135 by AugPatho were assigned by other tools. We found that most of these reads were classified as taxID 2148 or 264636 by the other approaches. As all three of these strains belong to the *Acholeplasmataceae* family, this pattern suggests the presence of shared genomic regions and potentially an unrepresented or novel genus within this family. To further examine this case, we mapped the relevant reads to all three references and found that none yielded strong, confident alignments, indicating that the true source strain is likely missing from the reference database. We then assembled the corresponding reads into contigs and classified them using Kraken2 against the full database. In 17 contigs classified under the expected family, the highest number of k-mers matched strain 2148, although the counts were low, again supporting the hypothesis of a missing true reference. Interestingly, strain 6135 is longer than both 2148 and 264636, and prior work on MORA has shown that AugPatho's scoring tends to favor longer, more complete genomes, which likely explains its preference for strain 6135 in this case.

Time and Memory Resources

Figure 8 presents the runtime and peak memory usage of the benchmarking tools on the ZymoD6331 ONT dataset which contained ~1.7M reads.

Since majority of the tools, except Kraken2 and Centrifuger, rely on Minimap2 for read mapping, we categorized peak memory usage into components: memory used by Minimap2 and memory used by other operations. Similarly, CPU time was divided into time spent by Minimap2 and time spent on all other processing steps.

In the case of MADRe_RC, MORA, and AugPatho, the "other operations" category solely consists of the read reassignment algorithm. In contrast, for MADRe it includes assembly, HairSplitter, database reduction, and read reassignment. The role of Minimap2 also differs across MADRe and other tools. In MADRe_RC, MORA, and AugPatho, it is used for mapping reads to the large reference database, whereas in MADRe it is used both to map contigs to the large database and reads to the reduced one.

For HiFi reads, Minimap2 uses different parameters, and the MADRe pipeline employs metaMDBG instead of metaFlye for assembly. To account for these differences, Supplementary Table ST22 reports the same performance metrics for HiFi data.

When the dataset size increases, the situation changes. To illustrate this, we included runtime and memory usage results for the large simulated dataset sim_high (containing ~5M reads) in Supplementary Table ST22. In this case, the peak RSS for MADRe

is substantially higher (exceeding 200 GB), primarily due to the assembly process, while the peak RSS for Minimap2 during read mapping to the large database remains unchanged. However, mapping reads to such a large database requires the "–split-prefix" parameter in Minimap2, which generates temporary alignment files that are later merged at the end of the process. For this particular dataset, that procedure consumes approximately 1.2 TB of disk space, whereas the complete MADRe pipeline requires 160 GB (excluding database and read file sizes in both cases). Moreover, the entire MADRe pipeline is approximately 3.2x faster than the combination of Minimap2 with MORA or AugPatho. In contrast, Kraken2 and Centrifuge are substantially faster than mapping-based approaches and require considerably less disk space.

Discussion

In this work, we introduced MADRe, a metagenomic classification pipeline based on assembly-driven database reduction followed by read classification through mapping and reassignment. By introducing statistical strategies for database reduction and read reassignment that explicitly leverage assembly-derived genomic context, MADRe represents a methodological contribution rather than an engineering integration alone. This approach enables accurate strain-level classification from large, multi-species databases without requiring prior knowledge of sample composition.

The first phase of MADRe reduces the reference database by identifying candidate strains that are likely to be present in the sample. Using assembly and an expectation–maximization (EM) soft clustering algorithm, this step aims to retain only the relevant references while eliminating unrelated ones. To take advantage of the longer contigs produced by standard assemblers, we avoided using strain-aware metagenome assemblers such as Strainberry [65], MetaBooster [66], HyLight [66], Strainy [67], and HairSplitter [52], which are known to yield shorter contigs. Instead, we used HairSplitter's functionality to estimate the number of collapsed strains for each contig and integrated this information with the mapping data of the initially strain-collapsed contigs. Our evaluation demonstrates that MADRe achieves effective database reduction while maintaining high recall.

Most existing strain-level classifiers either require single-species input or do not scale to large reference databases. Tools such as Kraken2, Sylph, or Centrifuger perform well at the species level and can handle large databases, making them valuable for pre-classification in strain-level workflows. However, such approaches generally require additional database preparation steps that are computationally intensive and impractical for complex metagenomic samples.

Mapping-based methods such as MORA and AugPatho represent another way to perform strain-level analysis on large databases. Nevertheless, our experiments showed that although MADRe incorporates an assembly step, typically considered both memory- and time-intensive, the overall memory footprint was lower than mapping raw reads directly to a large reference database, and even lower in memory than running Kraken2 on the same reference, when applied to a dataset of approximately 1.6 million ONT reads. This indicates that the upfront cost of assembly can be offset by the reduced complexity of downstream mapping. Importantly, the EM-based refinement and the reference reduction algorithm itself contribute only minimal additional computational overhead compared to assembly and large-scale read mapping. For larger datasets that exceed 5 million reads, the assembly process becomes more memory demanding. However, MADRe remains substantially faster and requires considerably less disk space than mapping-based approaches. While the runtime and resource usage of Minimap2 could be reduced by using a smaller reference database, this would again require prior knowledge of the sample composition or risk omitting relevant strains. Among the evaluated strain-level tools, MADRe is the fastest, providing an effective balance between computational efficiency and classification accuracy, and is thus well suited for scalable strain-level metagenomic analyses.

Our benchmarking analysis compared MADRe to MADRe_RC, MORA, the two AugPatho modes (PathoID and PathoREP), and, in several cases, to Centrifuger and Kraken2. For selected datasets, we additionally evaluated abundance re-estimation using Centrifuger-quant (Supplementary tables ST5-7), which reports read-count-based abundance estimates rather than read-level classifications and showed only minor differences compared to standard Centrifuger results; therefore, it was not included in all analyses. For AugPatho, we used the updated SAM files generated during its reassignment step, in which individual reads in some cases can be associated with multiple references. This format may improve the detection of expected references but can also introduce ambiguity, potentially contributing to higher false-positive rates. On the new simulated datasets, MADRe achieved up to a 28% improvement over other state-of-the-art methods when no clustering of similar strains was applied, and up to a 10% improvement when clustering was used. A similar trend was observed for the more complex large sized simulated datasets. Although MADRe occasionally missed low-abundance strains in these datasets, it still produced more accurate classifications than competing tools. This is particularly important since MADRe focuses on precise read-level classification rather than on abundance estimation.

A major challenge in metagenomic evaluation is the scarcity of realistic benchmark datasets, which can lead to parameter overfitting across methods, often to well-known datasets such as the Zymo communities. This may explain observations like those in the D6331 ONT dataset, where MORA and AugPatho showed substantial discrepancies between BC distances calculated from all classified reads and those derived only from true positives - the distances for true positives were notably higher. In contrast, MADRe consistently achieved better results than other tools for both evaluation types, demonstrating robust classification performance.

One limitation of MADRe observed in the Zymo benchmarks is the higher number of false-negative identifications, largely stemming from low-abundance organisms that are difficult to capture through the assembly-based approach, particularly when these organisms belong to species represented by a large number of closely related strains in the reference database. However, a similar effect can be seen in AugPatho's final reports, which include only abundance estimates from the last reassignment step - these also exhibit increased false negatives. This highlights a broader issue in metagenomic classification: setting thresholds for reporting low-abundance taxa inevitably trades off between reducing false positives and increasing false negatives [68]. The identification and quantification of low-abundance organisms remain challenging problems. MADRe does not apply any automatic post-filtering, leaving the decision of whether to perform additional filtering or manual investigation of low-abundance taxa to the user.

A closer look at the composition of the Zymo datasets and the definitions of ground-truth labels provides additional insight into the observed differences in tool performance. We can clearly observe performance variation across the three Zymo datasets, which can be attributed to both the sequencing technology and the evaluation methodology. As expected, the D6331 HiFi dataset yielded the best results, reflecting the higher base-level accuracy of HiFi reads compared to ONT. At first glance, it may seem surprising that performance on D6322 ONT was lower than on D6331 ONT, since D6322 contains species from different genera and should, in principle, be easier to classify. The main factor explaining this discrepancy lies in how ground-truth labels were defined. For D6322, the evaluation was straightforward - each genome represented a distinct species, and thus, an exact species-level match was required for a correct classification. In contrast, D6331 includes five E. coli genomes, three of which have very high sequence identity (greater than 99.3% ANI score - calculated using fastANI). When constructing the ground

truth for D6331, we clustered these three genomes and considered a read originating from any of them as correctly classified if it was assigned to any genome within that cluster. This less stringent criterion results in higher apparent performance for D6331 compared to D6322, an effect that applies uniformly across all evaluated tools.

In the real metagenomic dataset, MADRe classified fewer strains and focused on a confident subset of dominant organisms. In contrast, MADRe_RC, MORA, AugPatho, and Centrifuger reported a much larger number of low-abundance strains. While this may suggest higher sensitivity, many of these additional detections are likely spurious strain-level assignments, particularly in cases where the data do not support precise strain resolution. In this dataset, certain true references were absent from the database. Under these conditions, AugPatho tended to favor longer, highly similar genomes, MORA and MADRe_RC dispersed reads across multiple low-abundance strains, whereas MADRe mostly assigned reads to the reference sharing the greatest number of similar regions with the true organism.

MADRe's reduced detection of low-abundance strains arises primarily from limitations inherent to assembly-based approaches. At very low coverage, strains may fail to assemble or may produce contigs that are too short or fragmented to provide sufficient strain-specific signal for confident detection during database reduction. As a result, low-abundance strains may be excluded early in the workflow. This effect can be further amplified when closely related, higher-abundance strains are present, as assembly may preferentially represent the dominant genome, reducing the amount of discriminative sequence available for reliable separation.

These observations reflect an intentional precision–recall tradeoff in the design of MADRe. By reducing the reference database, MADRe prioritizes minimizing false-positive strain detections. At the genome-identification level, this may lead to reduced recall for extremely low-abundance strains. However, at the read level, the impact is typically limited because such strains contribute only a small fraction of total reads. When a strain is not retained during database reduction, its reads are frequently assigned to a highly similar reference, resulting in stable recall but a potential reduction in precision. From a biological perspective, this behavior is advantageous in scenarios where false-positive strain detection carries greater consequences than missing very low-abundance organisms.

As noted above, challenges extend beyond low-abundance effects and arise when both the reference database and the analyzed dataset contain genomes exhibiting extremely high sequence similarity. This was investigated through a dedicated high-similarity stress test (Supplementary File: Similar Strains Experiment; Supplementary Table ST23) and additional synthetic mixtures spanning combinations of similarity and coverage depth (Supplementary File: Coverage and Similarity Experiment; Supplementary Table ST24). These experiments demonstrate that when sequence similarity exceeds 99.9% ANI, strain-level discrimination becomes intrinsically ambiguous, largely independent of coverage. While very low coverage can limit detectability, the dominant constraint in these regimes is sequence similarity rather than abundance. Under such conditions, all evaluated methods approach fundamental resolution limits, although they differ in how ambiguity is handled. MADRe addresses this ambiguity by consistently assigning reads to the most similar available reference (the centroid), yielding stable behavior even when exact strain-level resolution is not supported by the data.

These observations also emphasize a broader limitation of current long-read metagenomic classifiers: all existing methods struggle to resolve strains at extremely low sequence divergence. For this reason, in our evaluation we additionally report results at the cluster level, where highly similar genomes are grouped together based on their mapping profiles. This approach avoids penalizing tools for inevitable redistribution within such groups and provides a more biologically meaningful measure of performance. Unlike conventional clustering by average nucleotide identity (ANI), our method groups references according to shared mapping profiles, focusing on patterns reflected in the data rather than static reference similarity. This design supports the concept of sample-aware reference groups that better capture functional and ecological relationships and could enhance classification accuracy in the presence of closely related organisms. Such clustering could also guide adaptive reference construction or real-time database refinement as additional samples are analyzed. Although clustering was used only for evaluation in this study and applied uniformly across all tools, future work will include deeper investigation of this method and its integration into the full MADRe pipeline.

As MADRe relies on metagenome assembly for database reduction, its performance can be influenced by assembly quality, particularly in highly complex or low-coverage samples. To assess sensitivity to assembler choice, we evaluated MADRe on large simulated datasets using Myloasm in addition to metaFlye and metaMDBG. Overall performance trends were comparable across assemblers, suggesting that MADRe is not strongly dependent on a specific assembly tool. We observed complementary behavior, with Myloasm showing improved detection of low-abundance strains and metaFlye performing slightly better for highly abundant ones. Strain-level assemblers were not considered, as they typically generate shorter and more fragmented contigs, whereas MADRe's reduction algorithm benefits from longer genomic context. While different parameter settings may influence assembly quality, all assemblers were executed using recommended configurations, and extensive parameter tuning was beyond the scope of this study.

Beyond strain-level classification, MADRe's modular design, particularly its database reduction and probabilistic reassignment components, offers potential for broader applications. These include contig binning, assembly refinement, and functional gene profiling, where confident reference reduction and ambiguity-aware read handling are equally valuable.

Conclusion

In this study, we introduced MADRe, a novel pipeline for strain-level metagenomic classification of long-read sequencing data. MADRe combines long-read assembly, EM-based contig-to-reference mapping reassignment for database reduction, and probabilistic read reassignment to deliver accurate and efficient classification, even without prior knowledge of sample composition. Unlike many existing tools, MADRe is designed to operate with large, diverse databases spanning multiple taxonomic levels, enabling high-resolution classification while minimizing false positives.

The pipeline consists of two distinct steps: database reduction and read classification, both of which can be executed independently. If general insight into the strains present in a sample is required, the first step can be used alone. Conversely, when prior knowledge about the sample exists, or when a reduced reference set is already available, the read classification step can be applied independently. MADRe provides a practical, scalable, and modular solution for strain-level classification in complex microbial communities.

Methods

MADRe Database Reduction

The database reduction step, shown in Figure 1 and illustrated in more detail in Supplementary Figure S1, consists of two main phases: input file preparation and the database reduction.

In the input file preparation phase, raw long metagenomic reads are first assembled using metaFlye for ONT reads or metaMDBG for HiFi reads. When multiple strains of the same species are present in

a sample, the assembly process can lead to strain collapse, producing contigs that represent a blend of closely related strains rather than distinct strain-specific sequences. Instead of using strain-aware metagenome assemblers, which typically generate shorter contigs, we chose to retain the longer contigs and infer strain-level complexity using HairSplitter functionality which estimates the number of collapsed strains per contig.

Assembled contigs are mapped to the reference database using Minimap2 with the *asm5* parameter preset, generating a PAF file as output. We chose this preset because, compared to *asm10* and *asm20*, it provides higher sensitivity, which is crucial for capturing more accurate and complete alignments of contigs to highly similar reference genomes. The MADRe database reduction process takes two key inputs: the estimated number of collapsed strains per contig determined by HairSplitter and the contig-to-reference mappings from Minimap2.

The database reduction process is based on the EM algorithm, which reassigns contigs to different references while performing soft clustering, allowing a single contig to be assigned to multiple references with different probabilities. The EM algorithm is widely used for handling ambiguous mappings in metagenomic classification [29, 30, 31, 32, 45, 69]. The implementation of the EM algorithm in MADRe is inspired by PathoScope2 [30] and EMU [31].

The database reduction process consists of three main steps. We can define a set of contigs as $C = \{c_1, c_2, \ldots, c_X\}$, where x is the number of contigs in the assembly. The set of references to which at least one contig is mapped is defined as $R = \{r_1, r_2, \ldots, r_g\}$, where g is the number of references. Additionally, let M represent the set of all of the mappings. In the first step, we compute a mapping score H for each mapping in the PAF file using the equation:

$$\mathrm{H} = 2 * \frac{\mathrm{N} \times ml}{\mathrm{N} + ml} \tag{1}$$

which represents the harmonic mean between the exact number of matches N and the mapping length ml. The ml is defined as the maximum value between the query mapping length and the reference mapping length. Applying the harmonic mean allows us to emphasize the smaller value, ensuring that a mapping does not receive an inflated score due, for example, to a very long but low-quality alignment.

The summarized mapping value S is then calculated for each contig-reference pair using:

$$\mathrm{S}(c_i, r_j) = \sum_{m \in M(c_i, r_j)} \mathrm{H}(p) \tag{2}$$

where $M(c_i, r_j)$ represents the set of mappings of contig c_i to reference r_j. This ensures that $\mathrm{S}(c_i, r_j)$ is computed by summing the mapping values of all instances where c_i maps to r_j, thus capturing all possible alignments between the contig and the reference. Following this, we divided mappings into *unique* and *non-unique*. Unique mappings occur when a contig maps exclusively to a single reference, whereas non-unique mappings represent ambiguous cases that require further resolution.

In the second step, non-unique mappings are processed using the EM algorithm, which iteratively refines contig assignments based on mapping probabilities. The E-step updates the expected assignments of contigs, while the M-step re-estimates the parameters using the newly computed assignment probabilities from the previous iteration.

The probability of selecting a reference r_i is given by:

$$P(r_i) = \frac{1}{G}, \quad \text{where} \quad G = |R| \tag{3}$$

The conditional probability of c_i given r_i is expressed as:

$$P(c_i|r_i) = \frac{\mathrm{S}(c_i, r_i)}{\max_{r_j \in R} \mathrm{S}(c_i, r_j)} \tag{4}$$

The log-likelihood function $L(X)$ is given by:

$$L(C) = \sum_{i=1}^{X} \log \left(\sum_{j=1}^{G} P(c_i \mid r_j) \cdot P(r_j) \right) \tag{5}$$

The expectation step (E-step) updates the posterior probability $P(r_i \mid c_i)$ as follows:

$$P(r_i \mid c_i) = \frac{P(c_i \mid r_i) \cdot P(r_i)}{\sum_{j=1}^{G} P(c_i \mid r_j) \cdot P(r_j)} \tag{6}$$

The maximization step (M-step) updates the prior probability $P(r_i)$ as follows:

$$P(r_i) = \frac{\sum_{j=1}^{X} P(r_i \mid c_j)}{|M|} \tag{7}$$

The EM algorithm runs iteratively until it converges or reaches the maximum number of iterations set by the stopping criteria. Once the algorithm outputs posterior probabilities, these values are used to determine which references will be included in the reduced database.

Before selecting references, we first classify each contig at the species level. This is done by summing the posterior probabilities across all references belonging to a species and assigning the contig to the species with the highest total probability. After determining the species classification, we retain only posterior probabilities associated with references belonging to the selected species. Finally, for each contig, we select N + 2 reference genomes to include in the reduced database. The value of N is estimated based on the number of collapsed strains identified by HairSplitter. By default, MADRe adds two additional references to avoid excluding expected strains, although this offset can be adjusted through user parameters.

MADRe Read Classification

The read classification step in MADRe is designed to operate both with and without prior database reduction. The only requirement is a PAF file, the Minimap2 output containing read-to-database mappings, where each database sequence includes the corresponding taxonomic identifier. The MADRe read classification workflow is illustrated in Supplementary Figure S2.

The process begins by computing a mapping score for each alignment, defined as the ratio between the number of exact matches (N) and the mapping length (ml):

$$S = \frac{N}{ml}$$

Mappings are then categorized into unique and non-unique. Since a read can have multiple alignments, only the alignment with the highest score is retained for each read-reference pair. If a read has a single best-scoring mapping, it is classified as a unique mapping. Conversely, if multiple mappings share the same highest score, they are considered non-unique, and the read will go through a reassignment process. Formally:

$$\text{Unique if } S_{r,i} = \max_{j}(S_{r,j}) \text{ and this maximum is unique;}$$

$$\text{Non-unique if } S_{r,i} = \max_{j}(S_{r,j}) \text{ for two or more } j.$$

Before reassigning non-unique mappings, reads are first classified at the species level. For each read r, the maximum mapping score among all references belonging to a species s is computed as:

$$S_{r,s}^{\max} = \max_{i \in s}(S_{r,i})$$

The species with the highest $S_{r,s}^{\max}$ is selected as the species-level assignment for that read. Although the default assumption is that a read can be uniquely mapped to a single species but may map ambiguously to multiple strains within that species, this assumption does not always hold. In rare cases, two genomes from different species may share highly similar regions, making it difficult to determine the true origin of a read. In such situations, reads are randomly distributed between the corresponding species. These cases are uncommon and typically arise from taxonomic inconsistencies, for example when nearly identical strains according to taxonomy belong to different species.

To reassign non-unique mappings at the strain level, a species-specific clustering algorithm is applied. This algorithm evaluates the number of unique and non-unique mappings associated with each reference within the same species, identifying groups of references that share many mappings, indicating that they likely represent overlapping genomic regions and should form clusters.

The fundamental assumption is that a reference truly present in the sample should accumulate the highest number of mappings (both unique and high-confidence non-unique). Let M_i denote the total number of mappings to reference i:

$$M_i = U_i + N_i$$

where U_i and N_i represent the counts of unique and non-unique mappings, respectively. The expected references are identified as those with the highest M_i within each cluster, and all non-unique reads are reassigned to the most probable reference in that cluster:

$$r \in \text{cluster}(i) \Rightarrow r \rightarrow \arg \max_{j \in \text{cluster}(i)} M_j$$

This procedure ensures that ambiguous reads are redistributed toward references that are both well supported by unique evidence and consistent with the mapping structure observed across the sample. By reassigning reads to the most representative reference within each cluster, this methodology establishes MADRe's centroid-based behavior, maintaining stable and interpretable classifications even in the presence of highly similar strains.

Abundances calculation

At this stage, each read is assigned to a single reference genome. Based on these assignments, MADRe calculates the abundance of each detected strain. The primary abundance output file reports the number of reads assigned to each reference. However, MADRe also provides an option to compute a length-normalized abundance, which accounts for both read and reference lengths. This alternative abundance metric is calculated as:

$$\text{Abundance}(r) = \frac{\sum_{i \in \text{Reads}_r} Length(i)}{\text{Length}(r)} \quad \text{for } r \in R \tag{8}$$

where R is the set of the references and $Reads_r$ is set of the reads classified under reference r.

Similar strains clustering

The high similarity between closely related strains and the lack of a clear threshold for defining when two sequences represent the same strain, makes it difficult to ensure that a reference database contains only unique strain sequences [34]. Some entries may correspond to highly similar strains or even to multiple assemblies of the same strain. To address this, MADRe includes an optional reference clustering step within the read classification process, designed to group similar references based on shared read mappings.

This clustering step operates on the same mapping file used in the classification stage and produces two output files: one reporting the abundances of identified clusters and the other listing the representative reference for each cluster.

The clustering procedure is illustrated in Supplementary Figure S3. It begins by using the calculated mapping scores and species-level labels. For each species-specific subset, the algorithm identifies the highest-quality mapping for each read. A binary vector is constructed for each reference, where each bit indicates whether a given read strongly supports that reference. These binary vectors are then clustered using DBSCAN with precomputed Jaccard distances, eps = 0.9, and min_samples = 1. Cluster-level abundances are computed accordingly. As a result, the post-clustering abundance files report only the representative references for each cluster.

Evaluation details

Our evaluation is primarily focused on exact read-level taxonomic assignments and read count-based abundance estimates.

To ensure a fair comparison, all tools were benchmarked using the same large reference database. For tools requiring mapping files as input, namely MORA, AugPatho, and MADRe_RC, we used a unified set of read-to-reference alignments generated by Minimap2. All reads were mapped to the full database, and the resulting SAM file was used directly for MORA and AugPatho. This SAM file was subsequently converted to PAF format using Paftools, as required by MADRe_RC.

Simulated reads were generated with the Badread tool [56], applying the whole-metagenome simulation mode with default values for chimeric, junk, and random reads. The simulation was performed using the *nanopore2023* model, which corresponds to the ONT R10.4.1. The exact command used is provided in the Supplementary File.

In case of simulated datasets, ground truth was available for every read, including its corresponding strain-level taxID and reference accession. Using this information, we computed true positives (TP), false positives (FP), true negatives (TN), and false negatives (FN) by comparing the assigned strain-level taxIDs with the expected ones. For Kraken2, we extracted read IDs and assigned taxIDs from its output. If a read was assigned to a higher taxonomic level, even if it was taxonomic correct, we treated it as a false positive, as the evaluation strictly focused on strain-level classification.

For the simulated datasets and Zymo mock communities we calculated Bray-Curtis (BC) distance as:

$$\text{BC}(x, y) = \frac{\sum_{i=1}^{n} |x_i - y_i|}{\sum_{i=1}^{n} (x_i + y_i)} \tag{9}$$

Where $x = (x_1, x_2, \ldots, x_n)$ and $y = (y_1, y_2, \ldots, y_n)$ are the abundance vectors for two samples or profiles, n is the number of strains, x_i and y_i are the abundances of the ith strain in samples x and y, respectively. BC(x, y) is the Bray-Curtis dissimilarity or distance, ranging from 0 (identical composition) to 1 (completely disjoint).

In the case of medium-sized simulated datasets (sim_small, sim_medium and sim_expanded) and the real dataset, the evaluation was also performed at the species level (results presented in Supplementary Table ST8 and Supplementary Figure S7). All strain-

level classifications were uplifted to their corresponding species, and read count abundances were calculated. For Kraken2, we used the species-level abundances reported in its summary file, limited to entries labeled with an “S” (species rank).

MADRe, MADRe_RC, and MORA each produce read-level classification files that associate each read with a reference genome. Since all tools shared the same mapping input, for AugPatho we ran PathoID and PathoREPORT steps, which output an updated SAM files and a report containing reference abundance estimates. In this updated SAM files, a single read can be associated with multiple references. For evaluation purposes, we allowed such multi-reference assignments, which may slightly benefit AugPatho by increasing the number of true positives, while also increasing the risk of false positives. These trade-offs are largely neutralized when clustering is applied, as similar strains typically end up grouped in the same cluster. With Centrifuger we encountered one limitation - Centrifuger cannot confidently assign a read to a specific reference sequence (e.g., when multiple chromosomes belong to the same strain), it often classifies the read under the NCBI strain-level taxid. In some cases, this strain taxid is identical to the species taxid, making it impossible to directly and fairly compare such classifications to those of other tools that operate at the sequence level. For benchmarking consistency, we therefore considered as true positives only the reads correctly classified under the expected reference sequence. It is important to note that this issue affected a relatively small fraction of reads (approximately 9000 out of 5 million reads in the 1000-genome dataset).

We used the same clustering across all tools to ensure consistency in cluster-based evaluation. Our clustering is based on read-to-reference mapping profiles, which can differ depending on the size and composition of the database. For example, when reads are mapped to a reduced database, the absence of certain similar references can make ambiguous mappings more resolvable. To avoid such inconsistencies, clustering was performed only once on the PAF file used for MADRe_RC, which contains read mappings to the complete reference database. During cluster-level evaluation, a classification was considered a true positive if the read was assigned to a reference that belongs to the same cluster as the ground truth reference:

$$\mathrm{TP} = \begin{cases} 1, & \text{if } C(\hat{r}) = C(r_{\mathrm{true}}) \\ 0, & \text{otherwise} \end{cases}$$

where $C(\hat{r})$ denotes the cluster of the predicted reference and $C(r_{\mathrm{true}})$ denotes the cluster of the true reference. A classification is considered a true positive (TP) if both references belong to the same cluster.

All commands used to perform classification with the evaluated tools are listed in the Supplementary File.

Availability of source code and requirements

- Project name: MADRe - Metagenome Assembly driven Database Reduction
- Project home page: https://github.com/lbcb-sci/MADRe
- Operating system(s): UNIX
- Programming language: Python
- Other requirements: Environment Modules, Conda, see https://github.com/lbcb-sci/MADRe
- License: MIT

Data availability

The source code for MADRe is available at [70]. Simulated data can be accessed via Zenodo [71]. Zymo D6322 ONT dataset is obtained from BioProject PRJNA1240873, zymo D6331 ONT dataset is obtained from [58], and zymo D6331 PacBio HiFi dataset from [59].

Additional files

SupplementaryFile.pdf; SupplementaryTables.xlsx

Declarations

List of abbreviations

EM: expectation-maximization; TP: true positive; TN: true negative; FP: false positive; FN: false negative; BC: Bray-Curtis; ANI: average nucleotide identity; ONT: Oxford Nanopore Technologies; taxID: taxonomy identifier.

Competing Interests

M.Š. has been jointly funded by Oxford Nanopore Technologies and AI Singapore for the project AI-driven De Novo Diploid Assembler. The remaining authors declare no competing interests.

Funding

This work was supported by the Croatian Science Foundation under grants IP-2018-01-5886 (SIGMA), DATACROSS (2024-2026, PK.1.1.10.0007), MOBDOK-2023-2941, and by the Singapore Ministry of Health’s National Medical Research Council, Singapore, under the grant MOH-000649-01 (Rapid diagnostic of infectious diseases based on nanopore sequencing and AI methods) – Individual Research Grant (NMRC/OFIRG/MOH-000649-00).

Acknowledgments

The authors thank Lune Angevin for testing the tool and providing valuable feedback.

Author’s Contributions

K.K. and M.Š. conceived the study. J.L. designed and implemented the pipeline. K.K. supervised database reduction implementation. R.V. supervised read classification implementation. J.L. drafted the manuscript. K.K., R.V. and M.Š. revised the manuscript. All authors read and approved the manuscript.

References

1. Gilbert JA, Blaser MJ, Caporaso JG, Jansson JK, Lynch SV, Knight R. Current understanding of the human microbiome. Nature medicine 2018;24(4):392–400. https://doi.org/10.1038/nm.4517.
2. Ling LL, Schneider T, Peoples AJ, Spoering AL, Engels I, Conlon BP, et al. A new antibiotic kills pathogens without detectable resistance. Nature 2015;517(7535):455–459. https://doi.org/10.1038/nature14098.
3. Arikan M, Muth T. gNOMO2: a comprehensive and modular pipeline for integrated multi-omics analyses of microbiomes. GigaScience 2024;13:giae038. https://doi.org/10.1093/gigascience/giae038.
4. Lu J, Rincon N, Wood DE, Breitwieser FP, Pockrandt C, Langmead B, et al. Metagenome analysis using the Kraken software suite. Nature Protocols 2022;17(12):2815–2839. https://doi.org/10.1038/s41596-022-00738-y.

5. Zhang W, Liu Y, Li G, Xu J, Chen E, Schönhuth A, et al. Strain-level metagenomic profiling using pangenome graphs with PanTax. Genome Research 2026;36(2):405–420. https://doi.org/10.1101/gr.280858.125.
6. Truong DT, Franzosa EA, Tickle TL, Scholz M, Weingart G, Pasolli E, et al. MetaPhlAn2 for enhanced metagenomic taxonomic profiling. Nature Methods 2015;12(10):902–903. https://doi.org/10.1038/nmeth.3589.
7. Blanco-Míguez A, Beghini F, Cumbo F, McIver LJ, Thompson KN, Zolfo M, et al. Extending and improving metagenomic taxonomic profiling with uncharacterized species using MetaPhlAn 4. Nature Biotechnology 2023;41(11):1633–1644. https://doi.org/10.1038/s41587-023-01688-w.
8. Ruscheweyh HJ, Milanese A, Paoli L, Sintsova A, Mende DR, Zeller G, et al. mOTUs: profiling taxonomic composition, transcriptional activity and strain populations of microbial communities. Current Protocols 2021;1(8):e218. https://doi.org/10.1002/cpz1.218.
9. Chen X, Yin X, Shi X, Yan W, Yang Y, Liu L, et al. Melon: metagenomic long-read-based taxonomic identification and quantification using marker genes. Genome Biology 2024;25(1):226. https://doi.org/10.1186/s13059-024-03363-y.
10. Costea PI, Munch R, Coelho LP, Paoli L, Sunagawa S, Bork P. metaSNV: a tool for metagenomic strain level analysis. PLOS ONE 2017;12(7):e0182392. https://doi.org/10.1371/journal.pone.0182392.
11. Olm MR, Crits-Christoph A, Bouma-Gregson K, Firek BA, Morowitz MJ, Banfield JF. inStrain profiles population microdiversity from metagenomic data and sensitively detects shared microbial strains. Nature Biotechnology 2021;39(6):727–736. https://doi.org/10.1038/s41587-020-00797-0.
12. Menzel P, Ng KL, Krogh A. Fast and sensitive taxonomic classification for metagenomics with Kaiju. Nature Communications 2016;7(1):11257. https://doi.org/10.1038/ncomms11257.
13. Buchfink B, Xie C, Huson DH. Fast and sensitive protein alignment using DIAMOND. Nature Methods 2015;12(1):59–60. https://doi.org/10.1038/nmeth.3176.
14. Steinegger M, Söding J. MMseqs2 enables sensitive protein sequence searching for the analysis of massive data sets. Nature Biotechnology 2017;35(11):1026–1028. https://doi.org/10.1038/nbt.3988.
15. Huson DH, Albrecht B, Bağcı C, Bessarab I, Gorska A, Jolic D, et al. MEGAN-LR: new algorithms allow accurate binning and easy interactive exploration of metagenomic long reads and contigs. Biology Direct 2018;13:1–17. https://doi.org/10.1186/s13062-018-0208-7.
16. Marić J, Križanović K, Riondet S, Nagarajan N, Šikić M. Comparative analysis of metagenomic classifiers for long-read sequencing datasets. BMC Bioinformatics 2024;25(1):15. https://doi.org/10.1186/s12859-024-05634-8.
17. Wood DE, Lu J, Langmead B. Improved metagenomic analysis with Kraken 2. Genome Biology 2019;20:257. https://doi.org/10.1186/s13059-019-1891-0.
18. Breitwieser FP, Baker DN, Salzberg SL. KrakenUniq: confident and fast metagenomics classification using unique k-mer counts. Genome biology 2018;19:1–10. https://doi.org/10.1186/s13059-018-1568-0.
19. Lu J, Breitwieser FP, Thielen P, Salzberg SL. Bracken: estimating species abundance in metagenomics data. PeerJ Computer Science 2017;3:e104. https://doi.org/10.7717/peerj-cs.104.
20. Kim D, Song L, Breitwieser FP, Salzberg SL. Centrifuge: rapid and sensitive classification of metagenomic sequences. Genome Research 2016;26(12):1721–1729. https://doi.org/10.1101/gr.210641.116.
21. Song L, Langmead B. Centrifuger: lossless compression of microbial genomes for efficient and accurate metagenomic sequence classification. Genome Biology 2024;25(1):106. https://doi.org/10.1186/s13059-024-03244-4.
22. Ounit R, Wanamaker S, Close TJ, Lonardi S. CLARK: fast and accurate classification of metagenomic and genomic sequences using discriminative k-mers. BMC genomics 2015;16:1–13. https://doi.org/10.1186/s12864-015-1419-2.
23. Ounit R, Lonardi S. Higher classification sensitivity of short metagenomic reads with CLARK-S. Bioinformatics 2016;32(24):3823–3825. https://doi.org/10.1093/bioinformatics/btw542.
24. Piro VC, Dadi TH, Seiler E, Reinert K, Renard BY. ganon: precise metagenomics classification against large and up-to-date sets of reference sequences. Bioinformatics 2020;36(Supplement 1):i12–i20. https://doi.org/10.1093/bioinformatics/btaa458.
25. Piro VC, Reinert K. ganon2: up-to-date and scalable metagenomics analysis. NAR Genomics and Bioinformatics 2025;7(3):lqaf094. https://doi.org/10.1093/nargab/lqaf094.
26. Ulrich JU, Renard BY. Fast and space-efficient taxonomic classification of long reads with hierarchical interleaved XOR filters. Genome Research 2024;34(6):914–924. https://doi.org/10.1101/gr.278623.123.
27. Shaw J, Yu YW. Rapid species-level metagenome profiling and containment estimation with sylph. Nature Biotechnology 2025;43(8):1348–1359. https://doi.org/10.1038/s41587-024-02412-y.
28. Dilthey AT, Jain C, Koren S, Phillippy AM. Strain-level metagenomic assignment and compositional estimation for long reads with MetaMaps. Nature Communications 2019;10(1):3066. https://doi.org/10.1038/s41467-019-10934-2.
29. Hong C, Manimaran S, Shen Y, Perez-Rogers JF, Byrd AL, Castro-Nallar E, et al. PathoScope 2.0: a complete computational framework for strain identification in environmental or clinical sequencing samples. Microbiome 2014;2:33. https://doi.org/10.1186/2049-2618-2-33.
30. Francis OE, Bendall M, Manimaran S, Hong C, Clement NL, Castro-Nallar E, et al. Pathoscope: species identification and strain attribution with unassembled sequencing data. Genome Research 2013;23(10):1721–1729. https://doi.org/10.1101/gr.150151.112.
31. Curry KD, Wang Q, Nute MG, Tyshaieva A, Reeves E, Soriano S, et al. Emu: species-level microbial community profiling of full-length 16S rRNA Oxford Nanopore sequencing data. Nature Methods 2022;19(7):845–853. https://doi.org/10.1038/s41592-022-01520-4.
32. Zheng A, Shaw J, Yu YW. Mora: abundance aware metagenomic read re-assignment for disentangling similar strains. BMC Bioinformatics 2024;25(1):161. https://doi.org/10.1186/s12859-024-05768-9.
33. Schaeffer L, Pimentel H, Bray N, Melsted P, Pachter L. Pseudoalignment for metagenomic read assignment. Bioinformatics 2017;33(14):2082–2088. https://doi.org/10.1093/bioinformatics/btx106.
34. Van Rossum T, Ferretti P, Maistrenko OM, Bork P. Diversity within species: interpreting strains in microbiomes. Nature Reviews Microbiology 2020;18(9):491–506. https://doi.org/10.1038/s41579-020-0368-1.
35. Luo C, Walk ST, Gordon DM, Feldgarden M, Tiedje JM, Konstantinidis KT. Genome sequencing of environmental Escherichia coli expands understanding of the ecology and speciation of the model bacterial species. Proceedings of the National Academy of Sciences 2011;108(17):7200–7205. https://doi.org/10.1073/pnas.1015622108.
36. Kashtan N, Roggensack SE, Rodrigue S, Thompson JW, Biller SJ, Coe A, et al. Single-cell genomics reveals hundreds of coexisting subpopulations in wild Prochlorococcus. Science 2014;344(6182):416–420. https://doi.org/10.1126/science.1248575.
37. Schloissnig S, Arumugam M, Sunagawa S, Mitreva M, Tap J, Zhu A, et al. Genomic variation landscape of the human gut

microbiome. Nature 2013;493(7430):45–50. https://doi.org/10.1038/nature11711.

38. Yassour M, Jason E, Hogstrom LJ, Arthur TD, Tripathi S, Siljander H, et al. Strain-level analysis of mother-to-child bacterial transmission during the first few months of life. Cell Host & Microbe 2018;24(1):146–154. https://doi.org/10.1016/j.chom.2018.06.007.

39. Yang Y, Dufault-Thompson K, Yan W, Cai T, Xie L, Jiang X. Large-scale genomic survey with deep learning-based method reveals strain-level phage specificity determinants. GigaScience 2024;13:giae017. https://doi.org/10.1093/gigascience/giae017.

40. van Dijk LR, Walker BJ, Straub TJ, Worby CJ, Grote A, Schreiber IV HL, et al. StrainGE: a toolkit to track and characterize low-abundance strains in complex microbial communities. Genome Biology 2022;23(1):74. https://doi.org/10.1186/s13059-022-02630-0.

41. Albanese D, Donati C. Strain profiling and epidemiology of bacterial species from metagenomic sequencing. Nature Communications 2017;8(1):2260. https://doi.org/10.1038/s41467-017-02209-5.

42. Roosaare M, Vaher M, Kaplinski L, Möls M, Andreson R, Lepamets M, et al. StrainSeeker: fast identification of bacterial strains from raw sequencing reads using user-provided guide trees. PeerJ 2017;5:e3353. https://doi.org/10.7717/peerj.3353.

43. Siekaniec G, Roux E, Lemane T, Guédon E, Nicolas J. Identification of isolated or mixed strains from long reads: a challenge met on Streptococcus thermophilus using a MinION sequencer. Microbial Genomics 2021;7(11):000654. https://doi.org/10.1099/mgen.0.000654.

44. Dempster AP, Laird NM, Rubin DB. Maximum likelihood from incomplete data via the EM algorithm. Journal of the Royal Statistical Society: Series B (Methodological) 1977;39(1):1–22. https://doi.org/10.1111/j.2517-6161.1977.tb01600.x.

45. Skoufos G, Almodaresi F, Zakeri M, Paulson JN, Patro R, Hatzigeorgiou AG, et al. AGAMEMNON: an Accurate metaGenomics And MEtatranscriptoMics quaNtificatiON analysis suite. Genome Biology 2022;23(1):39. https://doi.org/10.1186/s13059-022-02610-4.

46. Gao Y, Luo H, Lyu H, Yang H, Yousuf S, Huang S, et al. Benchmarking short-read metagenomics tools for removing host contamination. GigaScience 2025;14:giaf004. https://doi.org/10.1093/gigascience/giaf004.

47. Jain C, Rodriguez-R LM, Phillippy AM, Konstantinidis KT, Aluru S. High throughput ANI analysis of 90K prokaryotic genomes reveals clear species boundaries. Nature Communications 2018;9(1):5114. https://doi.org/10.1038/s41467-018-07641-9.

48. Koslicki D, White S, Ma C, Novikov A. YACHT: an ANI-based statistical test to detect microbial presence/absence in a metagenomic sample. Bioinformatics 2024;40(2):btae047. https://doi.org/10.1093/bioinformatics/btae047.

49. Anyansi C, Straub TJ, Manson AL, Earl AM, Abeel T. Computational methods for strain-level microbial detection in colony and metagenome sequencing data. Frontiers in Microbiology 2020;11:1925. https://doi.org/10.3389/fmicb.2020.01925.

50. LaPierre N, Alser M, Eskin E, Koslicki D, Mangul S. Metalign: efficient alignment-based metagenomic profiling via containment min hash. Genome Biology 2020;21(1):242. https://doi.org/10.1186/s13059-020-02159-0.

51. Koslicki D, Zabeti H. Improving minhash via the containment index with applications to metagenomic analysis. Applied Mathematics and Computation 2019;354:206–215. https://doi.org/10.1016/j.amc.2019.02.018.

52. Faure R, Lavenier D, Flot JF. HairSplitter: haplotype assembly from long, noisy reads. Peer Community Journal 2024;4. https://doi.org/10.24072/pcjournal.481.

53. Kolmogorov M, Bickhart DM, Behsaz B, Gurevich A, Rayko M, Shin SB, et al. metaFlye: scalable long-read metagenome assembly using repeat graphs. Nature Methods 2020;17(11):1103–1110. https://doi.org/10.1038/s41592-020-00971-x.

54. Benoit G, Raguideau S, James R, Phillippy AM, Chikhi R, Quince C. High-quality metagenome assembly from long accurate reads with metaMDBG. Nature Biotechnology 2024;42(9):1378–1383. https://doi.org/10.1038/s41587-023-01983-6.

55. Shaw J, Marin MG, Li H. High-resolution metagenome assembly for modern long reads with myloasm. bioRxiv 2025;https://doi.org/10.1101/2025.09.05.674543.

56. Wick RR. Badread: simulation of error-prone long reads. Journal of Open Source Software 2019;4(36):1316. https://doi.org/10.21105/joss.01316.

57. Zhang W, Benchmarking datasets used in the manuscript "Strain-level metagenomic profiling using pangenome graphs with PanTax". Zenodo; 2025. https://zenodo.org/records/16885808, version v3; accessed 2025-10-19.

58. Liu L, Yang Y, Deng Y, Zhang T. Nanopore long-read-only metagenomics enables complete and high-quality genome reconstruction from mock and complex metagenomes. Microbiome 2022;10(1):209. https://doi.org/10.1186/s40168-022-01415-8.

59. Portik DM, Brown CT, Pierce-Ward NT. Evaluation of taxonomic classification and profiling methods for long-read shotgun metagenomic sequencing datasets. BMC Bioinformatics 2022;23(1):541. https://doi.org/10.1186/s12859-022-05103-0.

60. Sereika M, Kirkegaard RH, Karst SM, Michaelsen TY, Sørensen EA, Wollenberg RD, et al. Oxford Nanopore R10.4 long-read sequencing enables the generation of near-finished bacterial genomes from pure cultures and metagenomes without short-read or reference polishing. Nature Methods 2022;19(7):823–826. https://doi.org/10.1038/s41592-022-01539-7.

61. O'Leary NA, Wright MW, Brister JR, Ciufo S, Haddad D, McVeigh R, et al. Reference sequence (RefSeq) database at NCBI: current status, taxonomic expansion, and functional annotation. Nucleic Acids Research 2016;44(D1):D733–D745. https://doi.org/10.1093/nar/gkv1189.

62. Bray JR, Curtis JT. An ordination of the upland forest communities of southern Wisconsin. Ecological Monographs 1957;27(4):326–349. https://doi.org/10.2307/1942268.

63. Zymo Research, ZymoBIOMICS: Microbiomics Solutions; 2025. https://www.zymoresearch.com/pages/zymobiomics, accessed: 2025-03-14.

64. Feng X, Cheng H, Portik D, Li H. Metagenome assembly of high-fidelity long reads with hifiasm-meta. Nature Methods 2022;19(6):671–674. https://doi.org/10.1038/s41592-022-01478-3.

65. Vicedomini R, Quince C, Darling AE, Chikhi R. Strainberry: automated strain separation in low-complexity metagenomes using long reads. Nature Communications 2021;12(1):4485. https://doi.org/10.1038/s41467-021-24515-9.

66. Luo X, Kang X, Schönhuth A. Enhancing long-read-based strain-aware metagenome assembly. Frontiers in Genetics 2022;13:868280. https://doi.org/10.3389/fgene.2022.868280.

67. Kazantseva E, Donmez A, Frolova M, Pop M, Kolmogorov M. Strainy: phasing and assembly of strain haplotypes from long-read metagenome sequencing. Nature Methods 2024;21(11):2034–2043. https://doi.org/10.1038/s41592-024-02424-1.

68. Simon HY, Siddle KJ, Park DJ, Sabeti PC. Benchmarking metagenomics tools for taxonomic classification. Cell 2019;178(4):779–794. https://doi.org/10.1016/j.cell.2019.07.010.

69. Sapoval N, Liu Y, Curry KD, Kille B, Huang W, Kokroko N, et al. Lightweight taxonomic profiling of long-read metagenomic

datasets with Lemur and Magnet. bioRxiv 2024;https://doi.org/10.1101/2024.06.01.596961.
70. Lipovac J, Lab Š, MADRe: Metagenome Assembly-Driven Database Reduction [Data set]. Zenodo.; 2026. https://github.com/lbcb-sci/MADRe, gitHub repository.
71. Lipovac J, Šikić Lab, MADRe: Metagenome Assembly-Driven Database Reduction; 2026. https://doi.org/10.5281/zenodo.18934466.

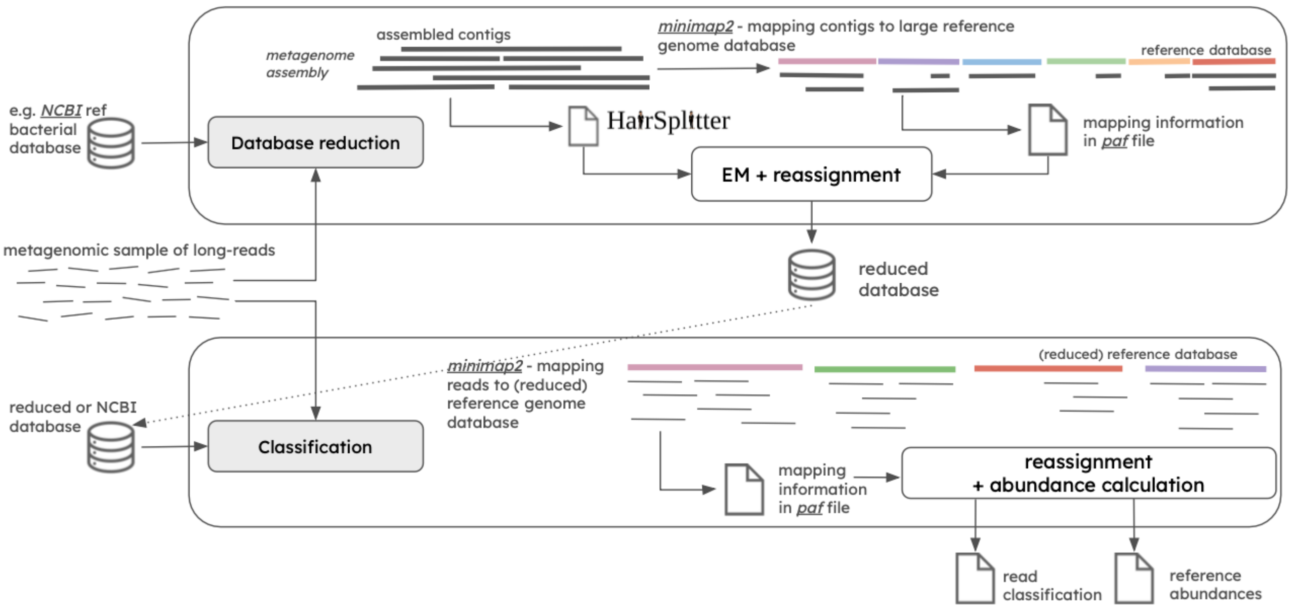

Figure 1. MADRe overall pipeline The first step of the pipeline performs database reduction using an EM-based contig-to-reference mapping procedure to identify organisms present in the sample. The second step involves read classification, which applies probabilistic read reassignment based on mapping information.

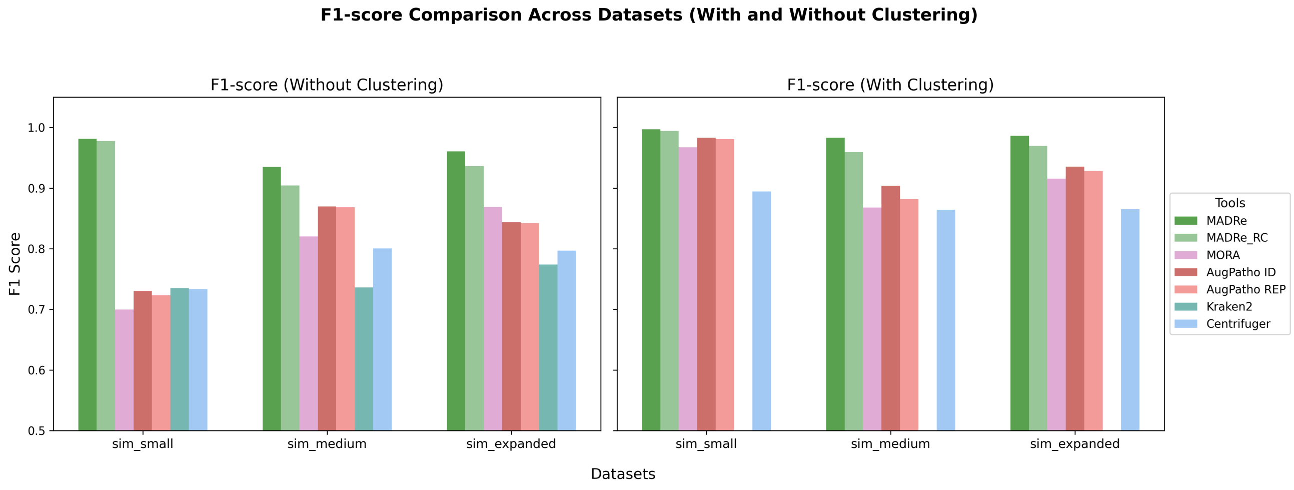

Figure 2. F1 scores of strain-level classification on medium-sized simulated reads, shown with and without post-clustering (grouping highly similar strains). Kraken2 results were omitted from the clustering analysis, as its output format does not support proper clustering.

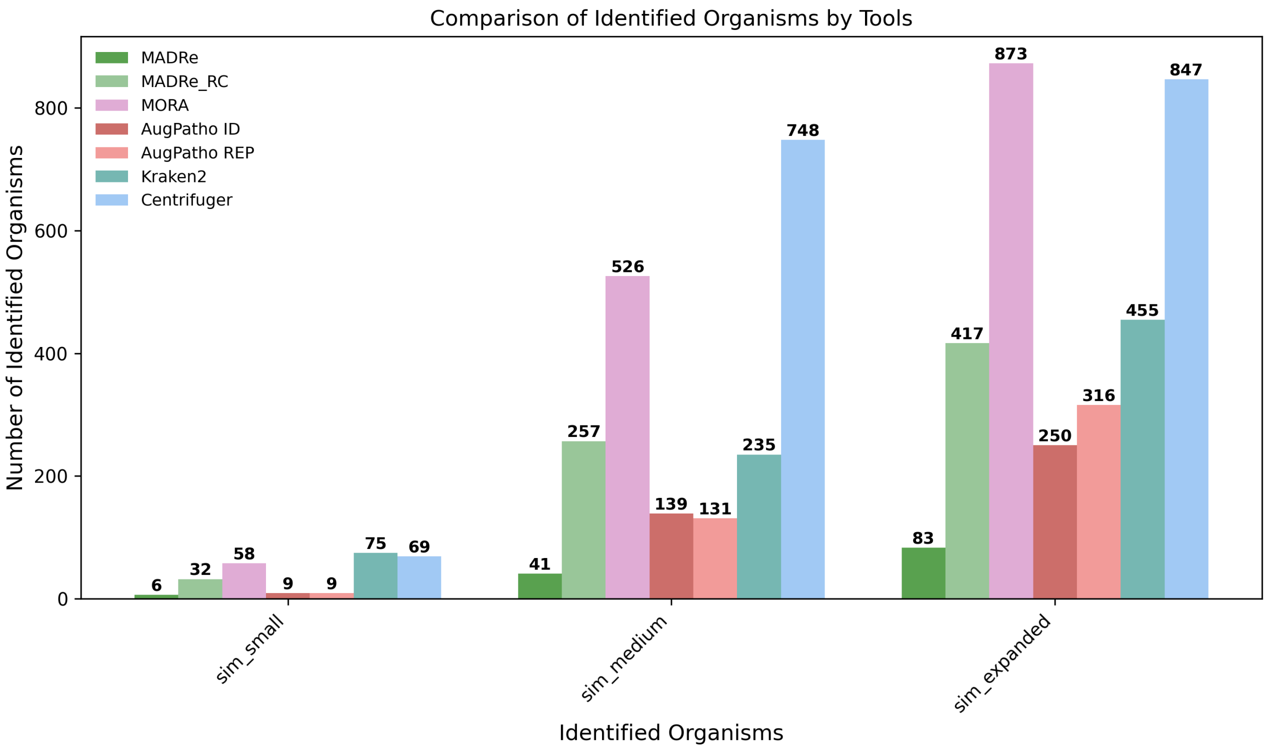

Figure 3. Number of identified organisms by different tools on simulated reads. An organism is considered identified if at least one read is classified under it. The sim_small dataset contains 4 strains, sim_medium contains 15 strains, and sim_expanded contains 30 strains. All tools successfully identified all expected strains, resulting in no false negatives.

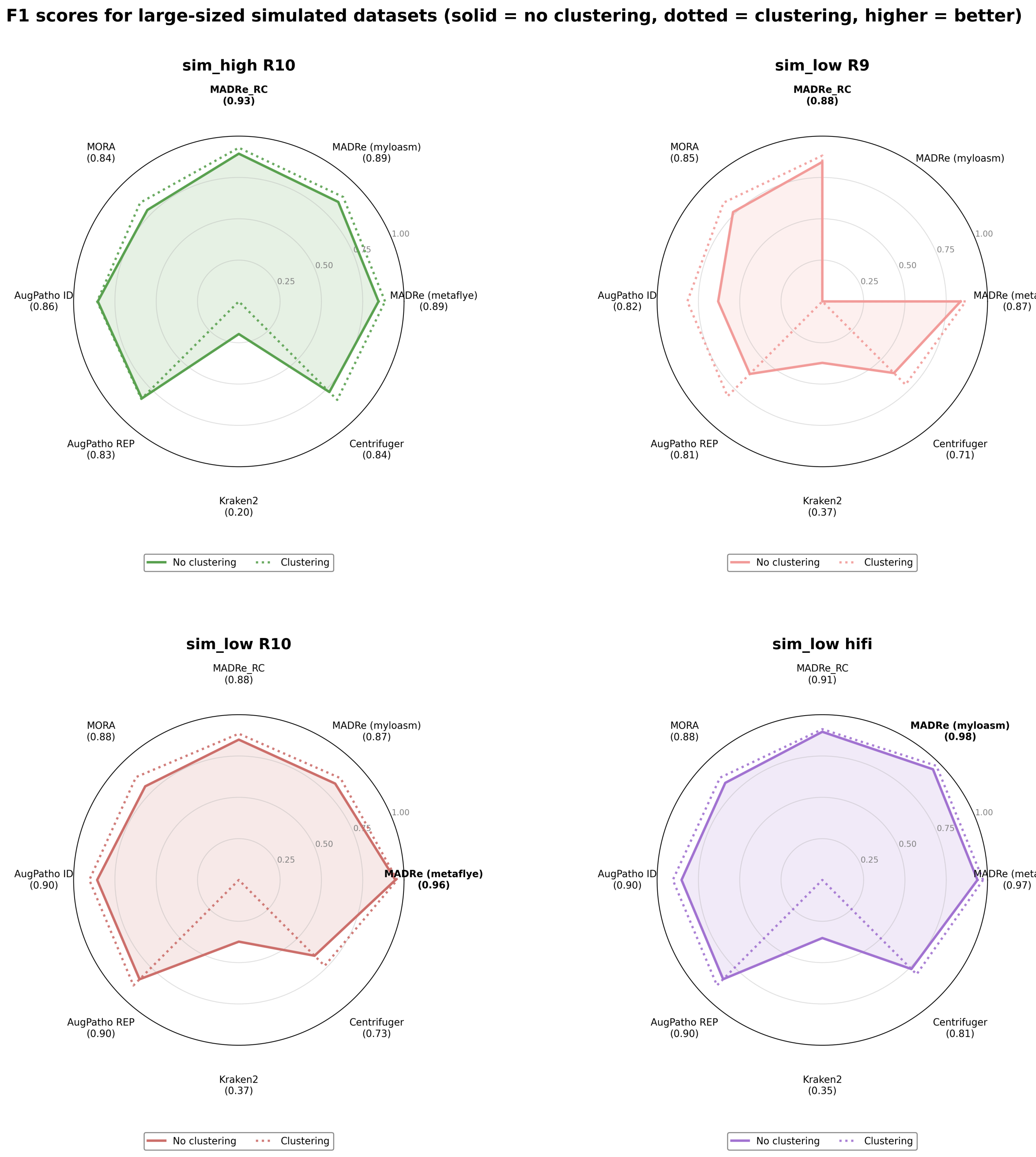

Figure 4. F1 scores for large-sized simulated datasets. Solid lines represent results without clustering, while dotted lines indicate results with clustering. Kraken2 clustering results are omitted, as its output format does not support clustering. Similarly, MADRe (Myloasm) results are excluded for ONT R9 data, since Myloasm is not designed for this type of sequencing data. In each plot, the best-performing tool is highlighted in bold, and the values in parentheses indicate the best performance achieved by each tool, with and without clustering.

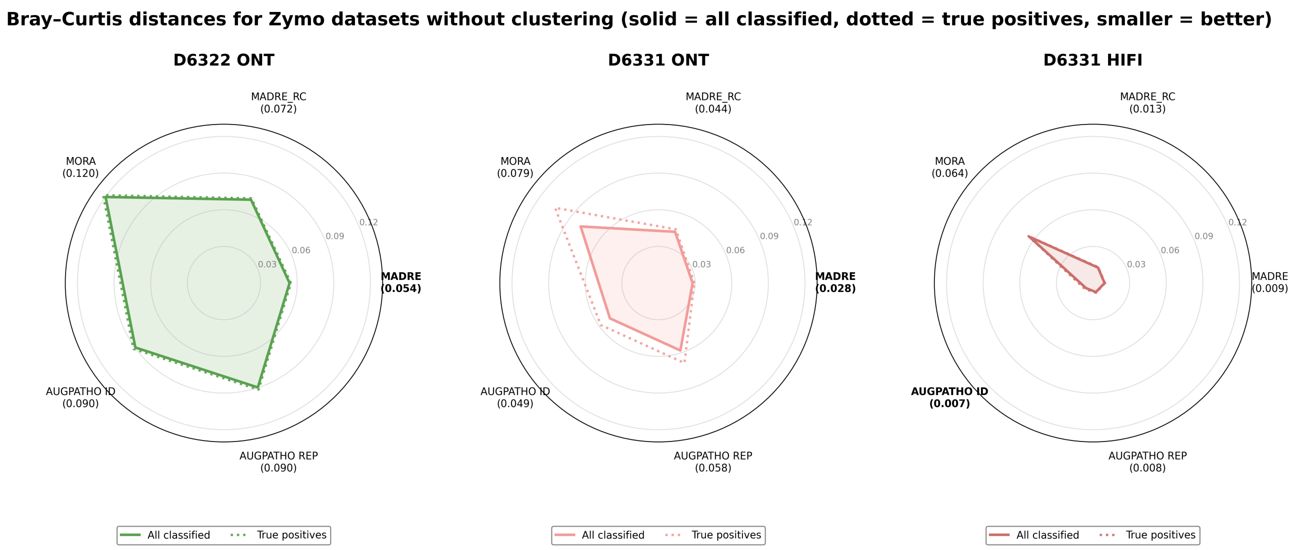

Figure 5. Bray–Curtis distances for Zymo datasets. The plots show changes in BC distance without post-clustering of similar strains. Solid lines represent distances based on all classified read counts, while dashed lines show distances calculated using only true positive (TP) read counts.

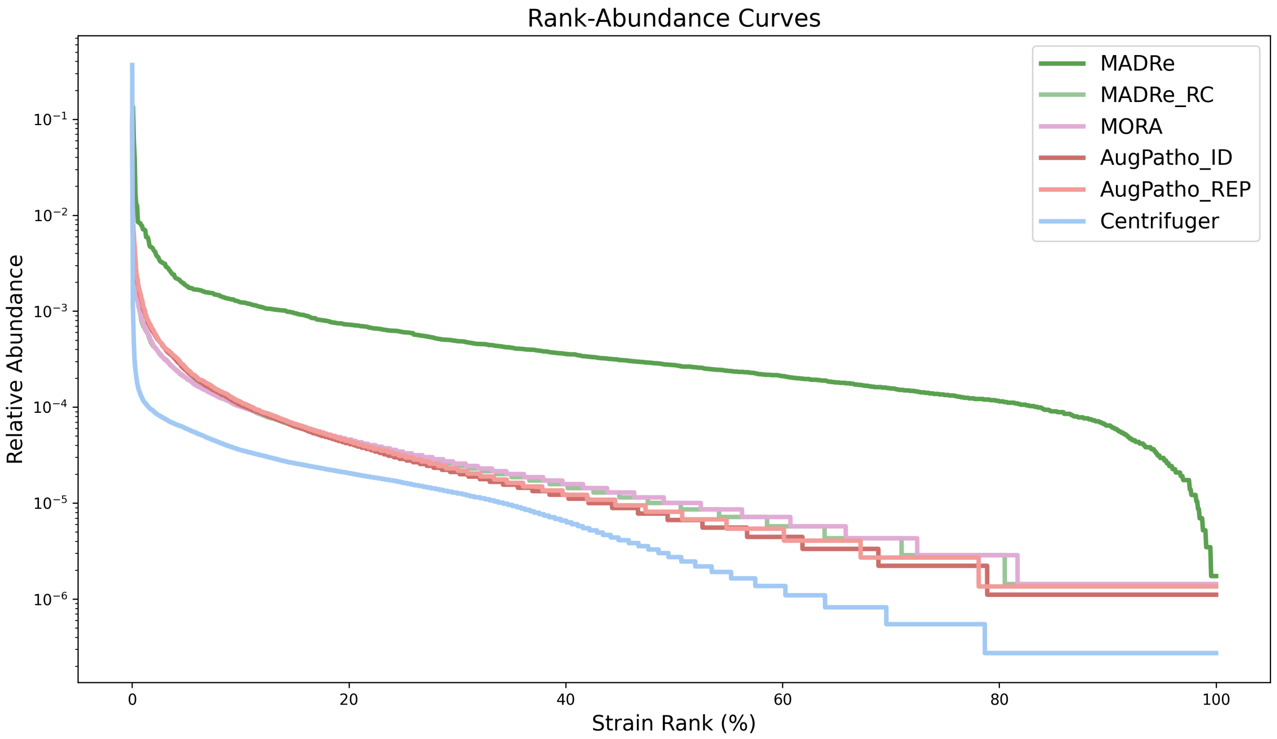

Figure 6. Percentile-normalized rank-abundance curves. The x-axis shows strain ranks expressed as percentiles, while the y-axis represents the relative abundance of each strain on a logarithmic scale.

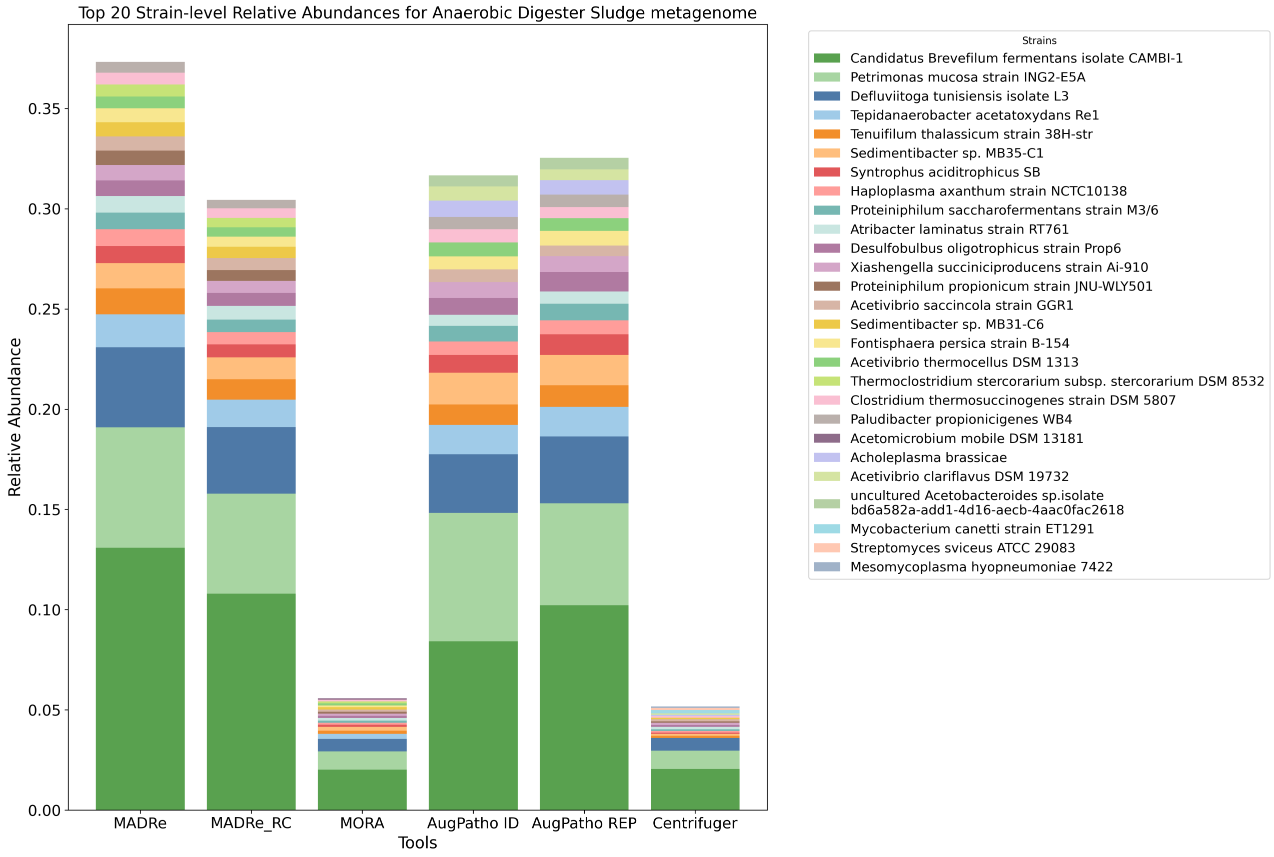

Figure 7. Real data strain-level abundances of the top 20 most abundant strains identified by each tool. Two strains are highlighted in red to illustrate cases where different tools classified reads originating from an unrepresented reference to distinct false positives that share similar genomic regions.

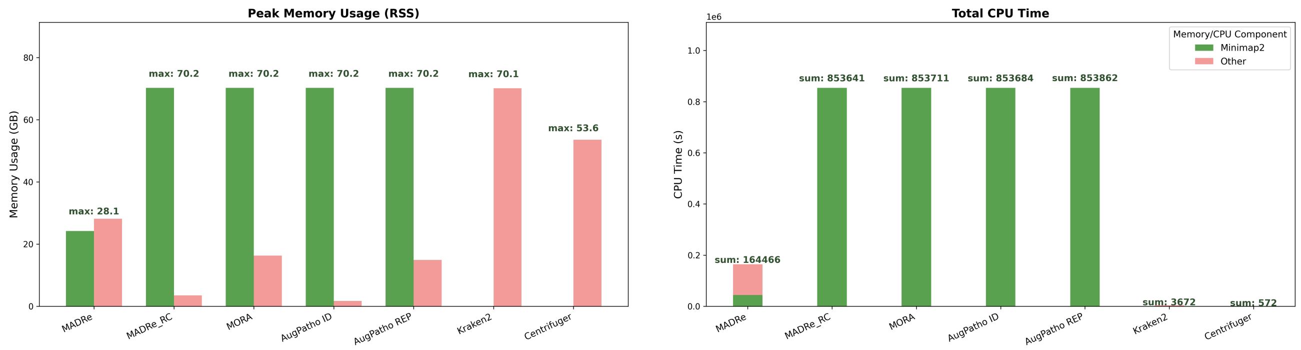

Figure 8. Memory (RSS peak in GB) and CPU time (in seconds) for different tools, split between Minimap2 mapping and other processing steps.

Click here to access/download;LaTeX - Other (clo, cls, sty etc.);example-grid-100x100pt.png

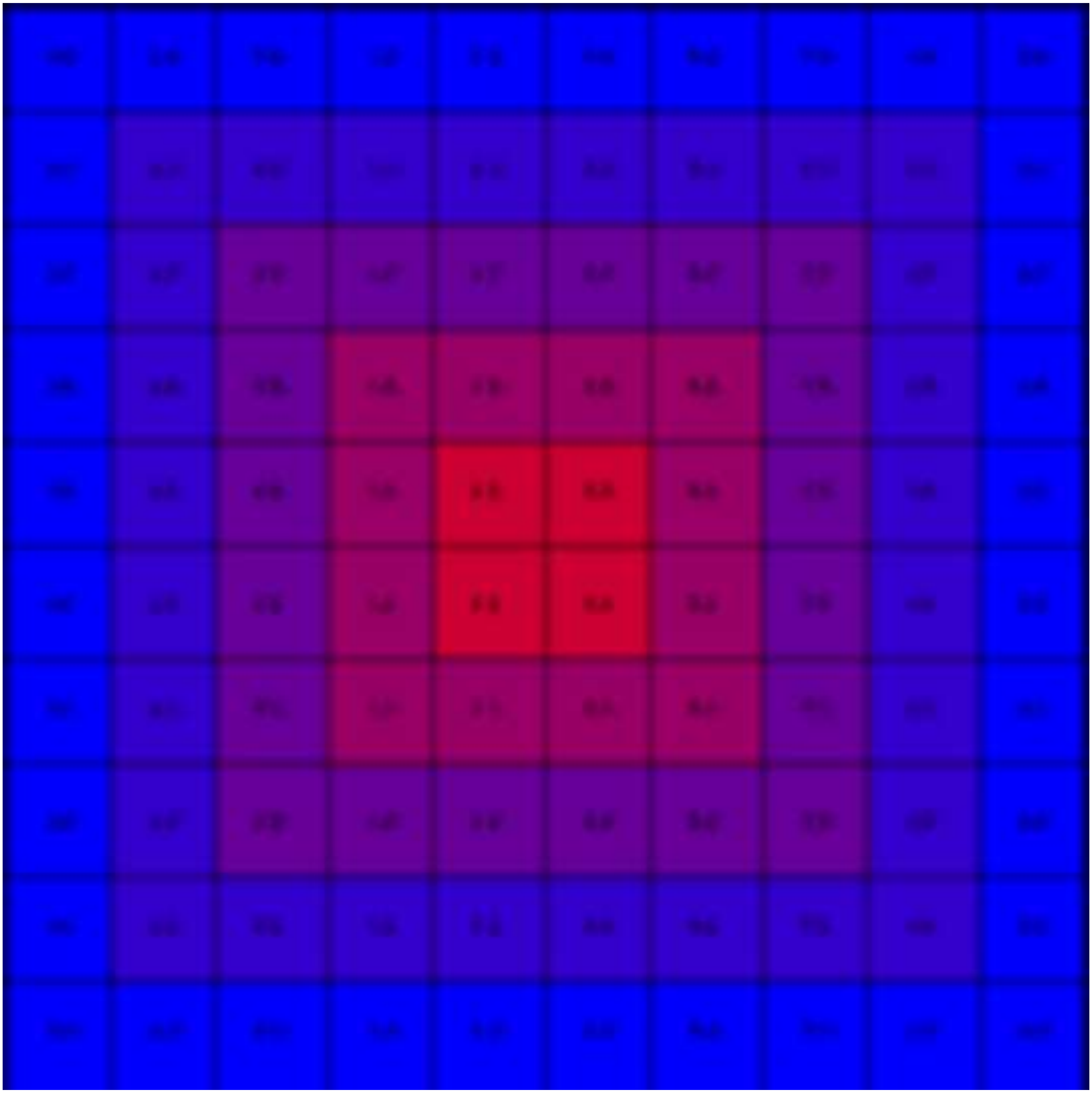

Click here to access/download;LaTeX - Other (clo, cls, sty etc.);example-image.png

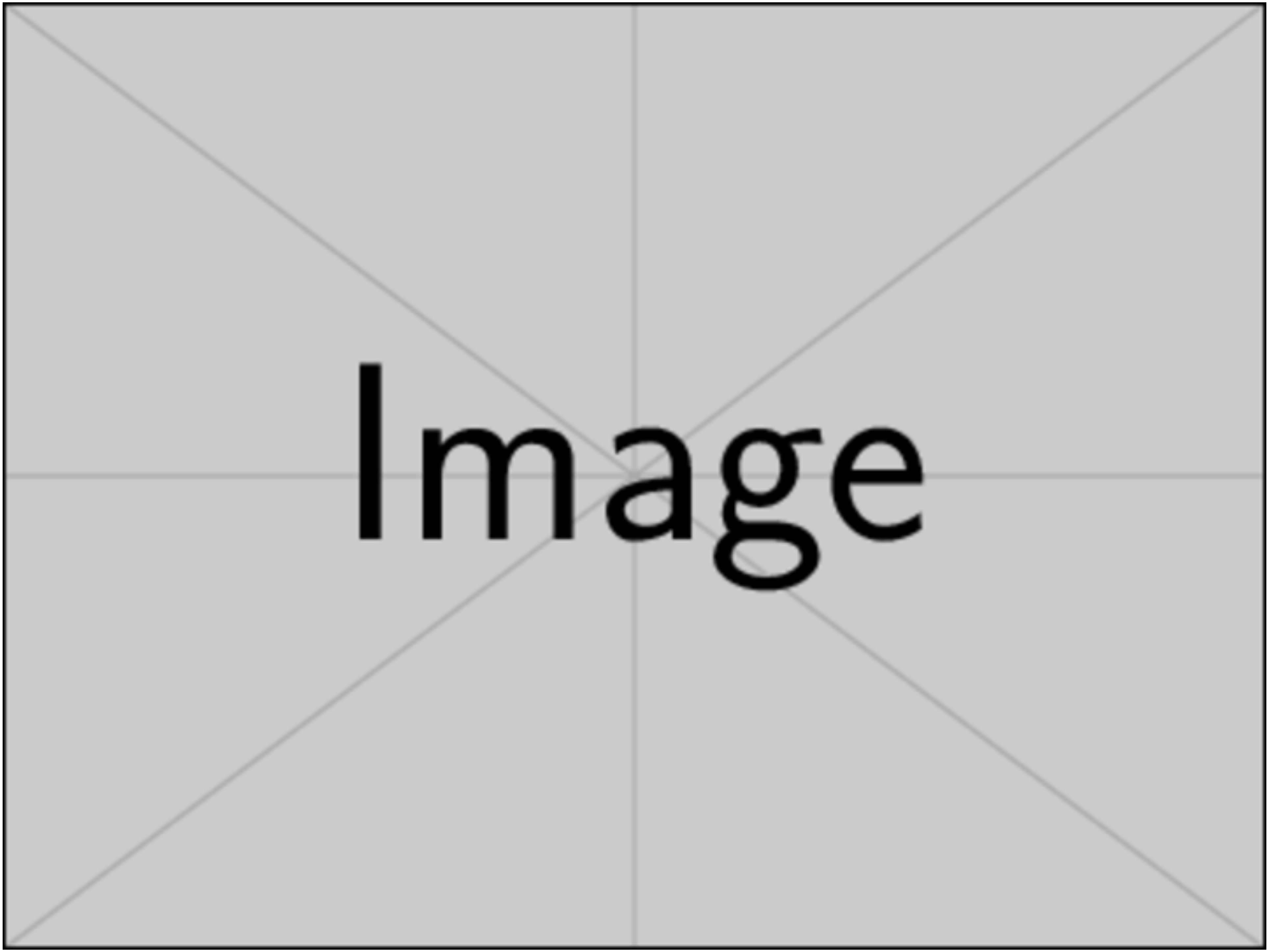

Manuscript

Click here to access/download;Manuscript;MADRe_manuscript.pdf

(GIGA)^n SCIENCE

GigaScience, 2026, 1–19

doi: xx.xxxx/xxxx
Manuscript in Preparation
Technical Note

TECHNICAL NOTE

MADRe: Strain-Level Metagenomic Classification Through Assembly-Driven Database Reduction

Josipa Lipovac[1,*], Mile Šikić[1,2], Riccardo Vicedomini[3,†] and Krešimir Križanović[2,*†]

[1]Laboratory for Bioinformatics and Computational Biology, Faculty of Electrical Engineering and Computing, University of Zagreb, Zagreb, Croatia and [2]Laboratory of AI in Genomics, Genome Institute of Singapore, A*STAR, Singapore, Singapore and [3]Univ Rennes, CNRS, Inria, IRISA - UMR 6074, F-35000 Rennes, France

[*]josipa.lipovac@fer.unizg.hr; kresimir.krizanovic@fer.unizg.hr
[†]Contributed equally.

Abstract

Strain-level metagenomic classification is essential for understanding microbial diversity and functional potential, yet remains challenging, particularly when sample composition is unknown and reference databases are large and redundant. Here we present MADRe, a modular and scalable pipeline for long-read strain-level metagenomic classification based on **M**etagenome **A**ssembly-Driven **D**atabase **Re**duction. Beyond system-level integration, MADRe introduces statistical strategies that leverage assembly-derived genomic context to guide database reduction and probabilistic read reassignment. Specifically, it combines long-read metagenome assembly, contig-to-reference reassignment using an expectation–maximization framework for reference reduction, and probabilistic read mapping reassignment on a reduced database to achieve sensitive and precise strain-level classification. We extensively evaluated MADRe on simulated datasets, mock communities, and a real anaerobic digester sludge metagenome. Across diverse similarity and coverage conditions, MADRe consistently improves precision by reducing false-positive strain detections. MADRe's design allows users to apply either the database reduction or read classification step individually. Using only the read classification step shows results on par with other tested tools. MADRe is open source and publicly available at https://github.com/lbcb-sci/MADRe.

Key words: metagenomics; strain-level; metagenomic classification; database reduction

Background

Metagenomics enables the study of genetic material from complex microbial communities found in environments such as human gut, soil, or marine ecosystems. It provides a comprehensive view of microbial diversity and interactions within these environments [1, 2]. Modern microbiome studies increasingly rely on modular and multi-omics analysis pipelines to integrate taxonomic and functional signals across data types [3]. Despite these advances, a central challenge in metagenomic analysis is the accurate identification of organisms present in a sample, typically performed by comparing sequencing reads to reference genome databases[4].

A wide range of metagenomic classification tools have been developed, which can be broadly categorized into marker-based, DNA-to-protein and DNA-to-DNA approaches, as described in [5]. Marker-based tools, such as MetaPhlAn [6, 7], StrainPhlAn, mOTUs [8], and Melon [9], classify taxa using conserved, clade-specific marker genes. In addition to marker-based methods, SNV-based profilers (e.g., metaSNV [10] and InStrain [11], which combines both approaches) represent important strategies for strain detection and population tracking. However, most of these approaches are optimized for short-read data and rely on predefined marker sets or variant catalogs, which may not fully capture genomic diversity in complex or underrepresented microbial communities.

Compiled on: March 16, 2026.
Draft manuscript prepared by the author.

Key Points

- MADRe implements a two-step strategy for strain-level classification: it first identifies candidate strains via assembly-to-database mapping with EM-based reassignment, then classifies reads by mapping to a reduced database via probabilistic reassignment.
- Despite incorporating assembly, MADRe significantly reduces runtime and memory usage compared to mapping all reads to the full reference database.
- MADRe enables the use of large, diverse reference databases without prior knowledge of sample content, focusing classification on confidently assembled strains and substantially reducing false positives while maintaining high strain-level resolution.

DNA-to-protein tools, including Kaiju [12], DIAMOND [13], MMseqs2 [14], and MEGAN-LR [15] translate reads into amino acid sequences before aligning them to protein databases. DNA-to-DNA tools compare reads directly against genomic sequences and are commonly divided into k-mer-based and mapping-based tools [16]. K-mer-based tools such as Kraken2 [17], KrakenUniq [18], Bracken [19], Centrifuge [20], Centrifuger [21], CLARK/CLARK-S [22, 23], Ganon [24, 25], Taxor [26], and Sylph [27] are known for their speed and scalability to large databases, but often trade precision for speed. In contrast, mapping-based tools such as MetaMaps [28], PathoScope2 [29, 30], EMU [31] and MORA [32], which rely on read alignments and reassignment algorithms, offer higher precision at a greater computational cost.

Although k-mer-based tools, especially Kraken2 or Sylph, perform well at the species level, strain-level classification becomes increasingly challenging when sequences originate from closely related genomes [33]. However, resolving strain-level diversity is essential, as even closely related strains can exhibit substantial differences in gene content and function, with implications for microbial ecology, pathogenesis, and treatment outcomes [34, 35, 36, 37, 38, 39].

While most existing tools are optimized for short reads due to their low cost and high accuracy, long-read sequencing technologies such as Oxford Nanopore and PacBio HiFi are rapidly improving. Longer read lengths provide advantages for genome assembly, structural-variant detection, and improved strain-level resolution.

Several short-read-based tools are designed for strain-level classification within a single species, such as StrainGE [40], StrainEST [41], and StrainSeeker [42], as well as the long-read-based ORI [43]. Other tools, including PanTax [5], MetaMaps [28], Centrifuge [20], Centrifuger [21], PathoScope2 [29, 30], and MORA [32], are suitable for more complex, multi-species datasets and support short- and long-read strain-level metagenomic classification. PanTax is a pangenome-based approach that, while supporting multi-species datasets, faces scalability limitations when applied to very large reference databases. MetaMaps is a mapping-based tool capable of high-resolution classification but is known to be extremely computationally demanding [16]. Centrifuge is a k-mer-based tool designed to perform strain-level classification, but like PanTax, it encounters limitations when constructing indexes for very large reference databases. However, its successor, Centrifuger, introduces improved compression and indexing strategies that enable efficient classification across large-scale genome databases. PathoScope2 is an older tool that is no longer maintained and cannot be reliably executed due to outdated dependencies and software incompatibilities. Originally developed for strain-level classification of short reads, it is based on an expectation-maximization (EM) algorithm for read reassignment [44]. As part of the MORA study, the authors introduced a continuation of PathoScope2, referred to as AugPatho, which includes a modified version of the original algorithm adapted for use with long reads [32]. MORA extends this approach by combining the EM algorithm from Agamemnon [45] with a read reassignment strategy based on the Weapon-Target Assignment (WTA) problem. According to its authors, MORA represents the current state-of-the-art in mapping-based long-read metagenomic classification.

Beyond algorithmic differences among tools, practical performance in metagenomic workflows can also be influenced by upstream preprocessing decisions and dataset composition, which can substantially affect downstream taxonomic inference [46].

Reference databases often contain multiple assemblies of the same strain and typically lack consistent organization. To address this, some strain-level classification tools perform database preclustering according to Average Nucleotide Identity (ANI) scores. Previous studies have shown that there is no universal ANI threshold for defining strains [40, 41, 47, 48, 34]. Setting the threshold too high may erroneously separate assemblies of the same strain, whereas setting a threshold too low may incorrectly group different strains together.

Using large and diverse databases is important for accurate strain-level classification [49], but it also makes the analysis much more demanding to run. MetaAlign [50] uses containment MinHash [51] to reduce the reference database prior to alignment, improving runtime while maintaining high species-level precision. However, it is primarily designed for short reads, and strain-level resolution is not its main focus.

In this work, we introduce MADRe, a pipeline for long-read, strain-level metagenomic classification enhanced with Metagenome Assembly-Driven Database Reduction, consisting of two main phases: database reduction and read classification. In the database reduction step, MADRe combines long-read assembly with an EM algorithm that assigns assembled contigs to one or more references, reducing the reference database.

In the second, read classification step, MADRe performs mappings-based read reassignment. It resolves ambiguous read mappings by assigning each read to the most likely reference, based on mapping scores and probabilistic support.

We conducted an extensive evaluation of MADRe using simulated datasets, Zymo mock communities, and a real anaerobic digester sludge metagenome. The results demonstrate that MADRe achieves high precision and strain-level resolution while maintaining lower memory usage and runtime compared to existing tools. Additionally, the two steps of the MADRe pipeline can be run independently, and our results show that the read classification module (MADRe_RC) alone performs competitively. MADRe's approach enables the use of large, diverse reference databases spanning multiple taxonomic levels, making it well-suited for scenarios where no prior knowledge about the sample is available. Using assembled contigs to detect potentially present strains, MADRe focuses on confidently represented organisms. As a result, compared to state-of-the-art tools, it significantly reduces the number of false positive identifications while maintaining high resolution strain-level classification.

Results

MADRe - method overview

The MADRe pipeline is designed for strain-level metagenomic classification, particularly in scenarios where prior knowledge of the sample composition is not available. Its primary goal is to enable accurate strain identification while reliably distinguishing truly abundant strains from false positives.

As illustrated in Figure 1, the MADRe pipeline consists of two main steps: database reduction and read classification. It takes as input a large bacterial reference database and raw long metagenomic reads, and produces two main outputs: a read classification file and a reference abundance file.

In the database reduction step, long reads are first assembled, and the resulting contigs are mapped to the large reference database. MADRe employs HairSplitter [52] to estimate the number of collapsed strains represented by each contig. Using both contig-to-reference mappings and the collapsed strain estimates, an EM algorithm is applied, followed by additional read-reassignment steps. This process generates a reduced reference database that highlights strains likely present in the sample. Although this reduction effectively narrows the search space, it may still include false positives introduced during the assembly process. These could be further filtered during the read classification step.

In the second step, reads are mapped to the reduced reference database, and ambiguous mappings are resolved through a reassignment procedure that leverages mapping scores and probabilistic support.

Both steps of the pipeline can be run independently, so when prior knowledge about the sample exists or when a reduced reference set is already available, the read classification step can be used on its own.

Benchmarking details

We benchmarked the MADRe pipeline against state-of-the-art tools developed for the same purpose: handling large reference databases while enabling strain-level classification. These tools include MORA and AugPatho (PathoScope2), for which we evaluated both of its key modules, PathoID and PathoReport. In some of the experiments, we also evaluated k-mer based tool Kraken2, one of the most widely used metagenomic classification tools, which is often considered the standard for species-level classification. Although Kraken2 is capable of assigning reads at the strain level, its evaluation is complicated by the use of taxonomic identifiers (taxIDs) that may refer to either species or strain ranks. Despite its popularity, recent work has shown that Sylph achieves superior performance for species-level abundance estimation, reporting fewer false positives. However, since Sylph is primarily designed for abundance profiling rather than direct read classification, we did not include it in our benchmarking, which focuses explicitly on classification accuracy. Additionally, we included MADRe_RC, a variant of MADRe that performs only the second step (*i.e.*, read classification) without prior database reduction.

We did not include MetaMaps, PanTax, or Centrifuge in our benchmarking analysis. In the case of MetaMaps, previous studies have reported crashes when attempting to build an index for the full Genome Taxonomy Database (GTDB), highlighting its scalability limitations [26, 32]. Similarly, our attempts to construct the same reference database for PanTax and Centrifuge, used successfully with other benchmarking tools, also failed due to crashes during the indexing process. Instead, we evaluated Centrifuger, a recent successor of Centrifuge that introduces improved compression and indexing strategies, enabling efficient classification on large-scale genome databases.

By default, MADRe employs metaFlye [53] for assembling Oxford Nanopore (ONT) reads and metaMDBG [54] for assembling PacBio HiFi reads. To assess the effect of different assembly strategies on database reduction, we also performed additional experiments using Myloasm [55], a recently developed assembler showing promising performance on metagenomic datasets.

All commands used to run the benchmarking tools are available in the Supplementary File (Tools versions and commands).

Datasets

As part of the benchmarking process, we evaluated the mentioned tools on simulated metagenome datasets, Zymo mock communities, and a real anareobic digester sludge metagenome.

For medium-sized simulated datasets, we selected a smaller subset of genomes representing species commonly found in the human gut microbiome. Reference genomes were required to be labeled as "complete" or "chromosome" in NCBI, and to have a strain-level taxID distinct from their species-level taxID. This criterion ensured that Kraken2 could be included in the evaluation.

Using Badread tool [56] we simulated three different metagenomic datasets:

i. **sim_small (4 strains)** – This dataset includes four different strain references: two strains from *Adlercreutzia equolifaciens* and two from *Streptococcus anginosus*, with varying relative abundances.
ii. **sim_medium (15 strains)** – This dataset contains 15 strain references distributed across five bacterial species (*i.e.*, *Helicobacter pylori*, *Cutibacterium acnes*, *Streptococcus intermedius*, *Streptococcus mutans*, *Lactococcus lactis*), with each species represented by three strains. At species level the abundances are different while strains of one species are equally abundant.
iii. **sim_expanded (30 strains)** – An extension of the sim_medium dataset, incorporating 15 additional strains from distinct species and maintaining variable abundance levels across species. Newly added species are listed in Supplementary Table ST1.

Exact genome information including accession numbers, strain and species taxIDs, genome lengths, genome coverages, ANI values (calculated usin fastANI [47], and number of simulated reads can be found in the Supplementary Tables (ST1-ST4).

Although these datasets can be used to assess MADRe's performance, they remain relatively simple and do not fully reflect the complexity of real metagenomic samples. Therefore, we expanded our benchmarking to include four additional simulated datasets originally used in the PanTax study [5] and we called them large-sized simulated datasets. Three of these datasets each contain 60 genomes coming from 30 species, simulated using ONT R9.4.1, ONT R10.4.1, and PacBio HiFi error profiles, respectively. These datasets were obtained directly from the PanTax Zenodo repository [57]. In addition, we generated a fourth, large-scale dataset comprising 1000 genomes from over 300 species, inspired by the CAMI challenge design. As simulated reads for this dataset were not available due to its size, we used the published reference genomes and expected abundances to simulate reads with the Badread tool. For these datasets, we additionally present distributions of ANI scores (calculated using fastANI), illustrating how many genome pairs exceed predefined ANI thresholds, as shown in Supplementary Table ST10.

We also tested MADRe using three Zymo mock communities: D6322 (ONT), D6331 (ONT) [58], and D6331 (PacBio HiFi) [59]. We included both D6311 ONT and D6311 PacBio HiFi datasets to demonstrate the pipeline's capability across different sequencing technologies.

To evaluate performance on a complex real-world dataset, we analyzed an anaerobic digester sludge metagenome dataset sequenced using ONT R10.4.1. reads [60]. This real metagenome represents the type of scenario for which MADRe is designed, where

a highly diverse sample is analyzed without prior knowledge of its taxonomic composition.

Database

To thoroughly evaluate strain-level classification and ensure sufficient taxonomic divergence for accurate strain-level detection, we used a database obtained via the Kraken2 interface by selecting the bacterial database. This database consists of 102,639 sequences, encompassing all RefSeq [61] complete bacterial genomes. The database was downloaded in December 2024. The exact command used for downloading the database is provided in the Supplementary File (Tools versions and commands).

For consistency, the same database was used across all tools and experiments.

Database Reduction

Table 1. Database reduction results. Comparison of MADRe's database reduction performance with two baseline models on simulated datasets using large database containing **102,639** sequences: baseline model 1 (BM1), which includes only the top-1 mapping for each contig, and baseline model 2 (BM2), which includes the top-3 mappings.

	**Metric**	**sim_small**	**sim_medium**	**sim_expanded**
number of genomes in dataset		4	15	30
**BM1**	# in reduced	7	34	68
	# missing strains	0	1	3
	# FP strains	3	19	38
	# FP species	1	0	6
**BM2**	# in reduced	14	107	234
	# missing strains	0	0	0
	# FP strains	10	92	204
	# FP species	5	11	78
**MADRe database reduction**	# in reduced	7	42	84
	# missing strains	0	0	0
	# FP strains	3	27	54
	# FP species	1	0	6

To assess the effectiveness of strain identification and database reduction, we evaluated the output of MADRe's database reduction step on simulated datasets by comparing it to two baseline models. More precisely, assembled contigs were mapped to the large reference database and the identification of organisms was carried out using the following strategies:

- Baseline Model 1 (BM1): For the reduced database, each contig's top reference genome, determined by the highest summarized harmonic mean mapping value (Methods, Equation 2), was included without performing any reassignment steps. BM1 represents the ideal reduction level under the assumption of a perfect assembly, where each contig corresponds to a single strain reference.
- Baseline Model 2 (BM2): For the reduced database, each contig's top three reference genomes, based on the highest summarized harmonic mean mapping values (Methods, Equation 2), were included without performing any reassignment steps. BM2 defines an upper bound on the number of references expected in the reduced database. Since our simulated metagenomes contain at most three strains per species, we assume that at most three strains could be collapsed into a single contig. This approach allows us to determine how many references should be retained in the reduced database to ensure that no true reference from the sample is missed.

The results, presented in Table 1, demonstrate that MADRe's database reduction achieves a high level of reduction while successfully retaining all expected strains. Additionally, when compared to BM2, MADRe reduces the number of false positive species, further highlighting its effectiveness.

Classification of medium-sized simulated datasets

For medium-sized simulated datasets we compared the classification performance of MADRe, MADRe_RC, MORA, AugPatho (in both PathoID and PathoReport modes), Kraken2, and Centrifuger. Since the exact source of each read is known, we define classification outcomes as follows: true positive (TP) if the read is classified under the expected strain (or expected cluster), true negative (TN) if the read is not classified and its Badread label is *random* or *junk*, false positive (FP) if the read is classified under the incorrect strain (or incorrect cluster), and false negative (FN) if the read is not classified but its label is different from *random* or *junk*.

We evaluated classification of simulated data with and without post-clustering. The post-clustering method described in the Methods section groups closely related strains based on read-to-reference mappings and assigns reads to clusters instead of individual strains. The same clustering approach was applied to all tools, utilizing read mappings to the full reference database. Kraken2's clustering results are not included, as taxID alone does not allow for an accurate evaluation of post-clustering performance.

The classification results of medium-sized simulated datasets (sim_small, sim_medium and sim_expanded) are presented in Figure 3 A., which shows the F1 scores for classification with and without post-clustering. The results demonstrate that both MADRe and MADRe_RC outperform all other approaches. Interestingly, on the sim_small dataset, which includes differently abundant strains of the same species, Kraken2 performs slightly better than Centrifuger, while Centrifuger achieves higher scores than MORA and AugPatho. For the remaining datasets, Centrifuger performs better than Kraken2 but worse than MORA and AugPatho. In the sim_expanded dataset (without clustering), MORA outperforms AugPatho's modes, but in all other cases, including all clustering scenarios, both AugPatho's modes perform significantly better than MORA. When post-clustering is applied, Centrifuger shows the lowest performance across all datasets, while post-clustering further improves AugPatho's results, bringing them close to MADRe_RC. Overall, MADRe_RC achieves performance comparable to MADRe, although this difference becomes more pronounced on more complex datasets. Figure 3B. shows the number of organisms identified by different tools on simulated datasets. An organism is considered identified if at least one read is classified under it. In all cases, there were no false negatives — all tools successfully identified the expected organisms. However, the number of additional (false positive) identifications varies. MADRe consistently reports significantly fewer false positives. For example, in the sim_small dataset, only 6 organisms were reported compared to the 4 expected, with 2 of them being extremely similar strains to those actually present in the sample. Additional metrics for organism-level identification, including TPs, FPs, FNs, accuracy, precision, recall, and F1 scores, are provided in Supplementary Table ST8, while classified read counts for each organism are listed in Supplementary Tables (ST5–ST7).

In addition, a Supplementary Figure S4 and Supplementary Table ST9 show Bray–Curtis (BC) distances [62] between the observed read count abundances and the ground-truth abundances, offering further insight into the similarity between the predicted and true community compositions. BC distance is one of the most commonly used distances to calculate the microbial abundance differences, and is described in the Methods section. From these results, it is

evident that MADRe achieves the closest match to the ground truth, while AugPatho ID reports the poorest scores among strain-level tools, including Centrifuger. Kraken2, although computationally efficient, shows the weakest overall performance.

Classification of large-sized simulated datasets

To further assess classification performance under more realistic metagenomic conditions, we used the simulated datasets from the *PanTax* study. We used the same evaluation procedure as the one considered for the medium-sized simulated datasets. In addition to benchmarking the standard MADRe pipeline, we also evaluated a variant in which Myloasm was used as the assembler during the database reduction step, in order to examine how different assembly approaches influence MADRe's performance. This variant was not tested on the sim_low R9.4.1 dataset, as Myloasm is not suitable for reads with that error profile.

Figure 4 presents four radar plots, each corresponding to one of the four large sized datasets, and showing F1 scores for all evaluated tools, both with and without post-clustering. (Note that post-clustering values for Kraken2 are zero, since this step was not performed for that tool.) For each tool, the best obtained F1 score is indicated in parentheses beneath its name. Across most datasets, MADRe achieved the highest scores, including its versions using different assemblers. In some cases, MADRe_RC slightly outperformed MADRe, particularly on the sim_high dataset. Detailed evaluation statistics are provided in Supplementary Table ST15. Exact read counts obtained from classifications are listed in Supplementary Tables (ST11–ST14), while Bray–Curtis (BC) distances between observed and expected read-count abundances are shown in Supplementary Figure S5 and Supplementary Table ST16.

Inspection of read-count abundances in the sim_high dataset revealed that several genomes were not detected during the database reduction step, leading to a modest decrease in MADRe's performance compared to MADRe_RC.

When comparing assembler performance, MADRe runs based on Myloasm assemblies achieved slightly higher F1 scores than those using metaFlye. A closer look at read-count abundances revealed that 24 genomes were detected exclusively with Myloasm and 11 exclusively with metaFlye. Among the 11 missed by Myloasm, 9 belonged to the more abundant half of the community, whereas among the 24 detected only by Myloasm, just one was highly abundant. This pattern suggests that Myloasm-based contigs perform better for low-abundance strains, while metaFlye contigs remain more reliable for highly abundant ones.

Another observation from the sim_high dataset is that, for several highly abundant strains, most benchmarking tools reported substantially lower read-count abundances than the ground truth. For example, reads originating from NZ_CP012672.1 were predominantly assigned to NZ_CP102233.1, a nearly identical genome (ANI = 99.998) annotated under a different species (Sorangium cellulosum vs. Sorangium sp. So ce836). Because clustering was performed only among strains within the same species, this near-duplicate across species boundaries could not be resolved even after clustering.

As shown in Figure 4, all tools achieved higher F1 scores on ONT R10 and PacBio HiFi datasets compared to ONT R9, reflecting the higher base accuracy of these sequencing platforms. Interestingly, for the ONT R10 dataset, MADRe using Myloasm performed slightly worse than the metaFlye version, whereas for PacBio HiFi reads, Myloasm yielded marginally better results than the metaMDBG-based variant.

Taken together, these results, including the analyses of BC distances, demonstrate that MADRe, in all assembler configurations, consistently outperforms the other evaluated tools on the large sized simulated datasets.

Classification of Zymo mock communities datasets

To evaluate MADRe on real sequencing data, we conducted experiments on three different Zymo mock community datasets: ONT Zymo D6322, which consists of eight organisms (seven bacterial species and one fungus), and both the ONT and HiFi versions of Zymo D6331, which contain 21 organisms, including two fungi and five different strains of *Escherichia coli*. The primary challenge in the Zymo D6331 dataset is the ability to distinguish between these closely related *E. coli* strains. In this analysis, we excluded fungal genomes, focusing solely on bacterial classifications.

For Zymo mock communities, exact reference genomes of the strains present in the sample are available, along with their theoretical relative abundances provided by ZymoBIOMICS [63]. We supplemented our database with the Zymo reference genomes, assigning them separate labels. However, we did not use provided theoretical abundances in our analysis, as they may deviate from the expected values due to variations in library preparation [9, 31]. Instead, we established ground-truth read classifications. We mapped all reads to the expected bacterial reference genomes using Minimap2 and assigned true labels based on the best hit. These assignments were also used to determine the relative abundances. However, this process was not straightforward for the five *Escherichia coli* strains, as their high similarity led to ambiguous mappings. To address this, we leveraged our clustering method (explained in *Similar strains clustering* section), which grouped these five strains into three clusters. Specifically, strains B766 and B3008 each formed separate clusters, while the remaining three strains were grouped into a single cluster, indicating that they were too similar to be reliably distinguished at the strain level. This clustering result aligns with previous findings from metagenome assembly procedures [64], where B766 and B3008 were successfully assembled, while the other three strains were not.

Benchmarking with Centrifuger and Kraken2 was not performed for this experiment, as their database construction procedures do not support the inclusion of references with custom labels, which is essential for this evaluation.

Using this information, we incorporated the clustering results into our ground-truth labeling: reads originating from the same cluster were assigned the same label, ensuring a more accurate classification.

To evaluate performance, we calculated the BC distances (eq.9) between the observed read count abundances and the ground-truth abundances, both with and without post-clustering.

Figure 5 depicts radar plots showing the BC distances for the zymo D6322 ONT, zymo D6331 ONT, and zymo D6331 HiFi datasets. Dotted lines indicate BC distances computed using only true positive classifications based on the ground truth. In the first plot, which reports results for the D6322 dataset, MADRe clearly outperforms all other tools. The second and third plots display BC distances for the D6331 ONT and HiFi datasets, respectively. For the ONT dataset, when considering all classified reads, MADRe achieves the lowest BC distance. When focusing only on true positives, MADRe and MADRe_RC show comparable performance, indicating that the majority of reads classified by these tools are correctly assigned. In contrast, MORA exhibits a notably higher BC distance when evaluated only on true positives, suggesting less precise classification. For the HiFi dataset, overall distances for all the tools are significantly lower. Both AugPatho modes achieve slightly lower BC distances compared to MADRe. In Supplementary Figure S6, we present the corresponding results obtained after post-classification clustering of similar strains. Interestingly, for the ONT datasets, BC distances increased for both AugPatho and MADRe following clustering. Although the increase is not substantial, the clustering step led to elevated abundance estimates, resulting in a higher number of both false positives and true positives. This trend was not observed for the HiFi dataset, where MADRe achieved the best performance after clustering.

Table 2. False positive (FP) species and strains detected by different tools on Zymo datasets. An organism is considered a false positive if at least one read is classified under it, but it is not in the true community.

	Tool	D6331 ONT	D6331 HiFi	D6322 ONT
**FP Species**	MADRe	5	5	6
	MADRe_RC	391	53	385
	MORA	639	162	517
	AugPatho ID	266	10	327
	AugPatho REP	249	20	260
**FP Strains**	MADRe	386	114	52
	MADRe_RC	3441	1189	6441
	MORA	6251	4010	10357
	AugPatho ID	2641	518	6133
	AugPatho REP	2316	889	4675

The exact read counts, used to calculate BC distance, are listed in the Supplementary Tables (ST17-ST19).

Table 2 presents the number of false-positive species and strain identifications. MADRe reports a significantly lower number of false positives at both levels compared to other tools. Supplementary Table ST20 provides a more detailed breakdown of the number of identifications. From this table, it is evident that MADRe's main limitation is the higher number of false negatives, primarily originating from low-abundance organisms that could not be detected using the assembly-based approach on which MADRe relies. This is further supported by the MADRe_RC results, where the number of false negatives is comparable to other tools. Nevertheless, MADRe consistently reports a substantially lower number of false positives. The table also includes AugPatho results from report outputs from both modes. These reports are generated after the final reassignment step and contain only abundance estimates. Consequently, they cannot be used directly for classification evaluation. While these reports show a significantly lower number of false positives, this reduction comes at the cost of a higher number of false negatives.

Classification of real anaerobic digester sludge metagenome

While Zymo mock communities represent real metagenomic data, they do not fully capture the complexity typically found in environmental or host-associated microbial communities. To better reflect realistic classification scenarios, we evaluated MADRe and the other competing tools on a real anaerobic digester sludge metagenome. As this dataset lacks ground truth, we focused on comparative analysis of classification outputs. All results presented here include post-clustering.

In this dataset, MADRe identified 1,320 reference strains (1,502 without clustering), while MADRe_RC reported 14,304 (19,067 without clustering), MORA 15,835 (23,516 without clustering), AugPatho ID 11,785 (16,488 without clustering), AugPatno REP 11,134 (14,604 without clustering) and Centrifuger 23,950 (28,450 without clustering). Out of 3,646,771 total reads, MADRe classified 575,052 ($\sim$ 16%), MADRe_RC 696,961 ($\sim$ 19%), MORA 696,839 ($\sim$ 19%), AugPatho ID 898,906 ($\sim$ 25%), AugPatho REP 737,714 ($\sim$ 20%) and Centrifuger 1,350,537 ($\sim$ 37%) reads.

Figure 6 illustrates percentile-normalized rank-abundance curves, highlighting differences in strain-level classification across the tools. The underlying read count abundance data used to generate this figure is provided in Supplementary Table ST21.

The curve for MADRe displays a consistent, moderately steep gradient throughout, with notable deviations at the beginning and end. The sharp rise at the beginning indicates the presence of a highly abundant strain, significantly more dominant than the others. This can be seen for the other tools as well. Toward the end, the curve drops sharply, likely reflecting false positives or low-confidence strain assignments. Compared to the other tools, MADRe shows a smoother and more gradual decline in the abundances of lower-ranked strains. In contrast, MADRe_RC, MORA, AugPatho and Centrifuger report a larger number of low-abundance strains, resulting in a more stepwise decline. The flat tail in their curves suggests that many strains are assigned near-zero abundances.

Figure 7 shows the relative abundances of the 20 most abundant strains reported by each tool, calculated relative to the total number of classified reads. We also generated an analogous visualization at the species level (Supplementary Figure S7), which additionally includes Kraken2 results. Among all tools, MADRe achieved the highest cumulative relative abundance for the top 20 strains, followed by AugPatho and MADRe_RC, while MORA and Centrifuger exhibited similar but substantially lower overall contributions from their top strains. Figure 7 highlights one notable strain-level discrepancy: the strain Paludibacter propionicigenes (accession number NC_022549.1, taxID 6135), which appeared among the top 20 only in AugPatho results. To investigate this discrepancy, we examined how reads classified as taxID 6135 by AugPatho were assigned by other tools. We found that most of these reads were classified as taxID 2148 or 264636 by the other approaches. As all three of these strains belong to the *Acholeplasmataceae* family, this pattern suggests the presence of shared genomic regions and potentially an unrepresented or novel genus within this family. To further examine this case, we mapped the relevant reads to all three references and found that none yielded strong, confident alignments, indicating that the true source strain is likely missing from the reference database. We then assembled the corresponding reads into contigs and classified them using Kraken2 against the full database. In 17 contigs classified under the expected family, the highest number of k-mers matched strain 2148, although the counts were low, again supporting the hypothesis of a missing true reference. Interestingly, strain 6135 is longer than both 2148 and 264636, and prior work on MORA has shown that AugPatho's scoring tends to favor longer, more complete genomes, which likely explains its preference for strain 6135 in this case.

Time and Memory Resources

Figure 8 presents the runtime and peak memory usage of the benchmarking tools on the ZymoD6331 ONT dataset which contained $\sim$1.7M reads.

Since majority of the tools, except Kraken2 and Centrifuger, rely on Minimap2 for read mapping, we categorized peak memory usage into components: memory used by Minimap2 and memory used by other operations. Similarly, CPU time was divided into time spent by Minimap2 and time spent on all other processing steps.

In the case of MADRe_RC, MORA, and AugPatho, the "other operations" category solely consists of the read reassignment algorithm. In contrast, for MADRe it includes assembly, HairSplitter, database reduction, and read reassignment. The role of Minimap2 also differs across MADRe and other tools. In MADRe_RC, MORA, and AugPatho, it is used for mapping reads to the large reference database, whereas in MADRe it is used both to map contigs to the large database and reads to the reduced one.

For HiFi reads, Minimap2 uses different parameters, and the MADRe pipeline employs metaMDBG instead of metaFlye for assembly. To account for these differences, Supplementary Table ST22 reports the same performance metrics for HiFi data.

When the dataset size increases, the situation changes. To illustrate this, we included runtime and memory usage results for the large simulated dataset sim_high (containing $\sim$5M reads) in Supplementary Table ST22. In this case, the peak RSS for MADRe

is substantially higher (exceeding 200 GB), primarily due to the assembly process, while the peak RSS for Minimap2 during read mapping to the large database remains unchanged. However, mapping reads to such a large database requires the "−split-prefix" parameter in Minimap2, which generates temporary alignment files that are later merged at the end of the process. For this particular dataset, that procedure consumes approximately 1.2 TB of disk space, whereas the complete MADRe pipeline requires 160 GB (excluding database and read file sizes in both cases). Moreover, the entire MADRe pipeline is approximately 3.2x faster than the combination of Minimap2 with MORA or AugPatho. In contrast, Kraken2 and Centrifuge are substantially faster than mapping-based approaches and require considerably less disk space.

Discussion

In this work, we introduced MADRe, a metagenomic classification pipeline based on assembly-driven database reduction followed by read classification through mapping and reassignment. By introducing statistical strategies for database reduction and read reassignment that explicitly leverage assembly-derived genomic context, MADRe represents a methodological contribution rather than an engineering integration alone. This approach enables accurate strain-level classification from large, multi-species databases without requiring prior knowledge of sample composition.

The first phase of MADRe reduces the reference database by identifying candidate strains that are likely to be present in the sample. Using assembly and an expectation–maximization (EM) soft clustering algorithm, this step aims to retain only the relevant references while eliminating unrelated ones. To take advantage of the longer contigs produced by standard assemblers, we avoided using strain-aware metagenome assemblers such as Strainberry [65], MetaBooster [66], HyLight [66], Strainy [67], and HairSplitter [52], which are known to yield shorter contigs. Instead, we used HairSplitter's functionality to estimate the number of collapsed strains for each contig and integrated this information with the mapping data of the initially strain-collapsed contigs. Our evaluation demonstrates that MADRe achieves effective database reduction while maintaining high recall.

Most existing strain-level classifiers either require single-species input or do not scale to large reference databases. Tools such as Kraken2, Sylph, or Centrifuger perform well at the species level and can handle large databases, making them valuable for pre-classification in strain-level workflows. However, such approaches generally require additional database preparation steps that are computationally intensive and impractical for complex metagenomic samples.

Mapping-based methods such as MORA and AugPatho represent another way to perform strain-level analysis on large databases. Nevertheless, our experiments showed that although MADRe incorporates an assembly step, typically considered both memory- and time-intensive, the overall memory footprint was lower than mapping raw reads directly to a large reference database, and even lower in memory than running Kraken2 on the same reference, when applied to a dataset of approximately 1.6 million ONT reads. This indicates that the upfront cost of assembly can be offset by the reduced complexity of downstream mapping. Importantly, the EM-based refinement and the reference reduction algorithm itself contribute only minimal additional computational overhead compared to assembly and large-scale read mapping. For larger datasets that exceed 5 million reads, the assembly process becomes more memory demanding. However, MADRe remains substantially faster and requires considerably less disk space than mapping-based approaches. While the runtime and resource usage of Minimap2 could be reduced by using a smaller reference database, this would again require prior knowledge of the sample composition or risk omitting relevant strains. Among the evaluated strain-level tools, MADRe is the fastest, providing an effective balance between computational efficiency and classification accuracy, and is thus well suited for scalable strain-level metagenomic analyses.

Our benchmarking analysis compared MADRe to MADRe_RC, MORA, the two AugPatho modes (PathoID and PathoREP), and, in several cases, to Centrifuger and Kraken2. For selected datasets, we additionally evaluated abundance re-estimation using Centrifuger-quant (Supplementary tables ST5-7), which reports read-count-based abundance estimates rather than read-level classifications and showed only minor differences compared to standard Centrifuger results; therefore, it was not included in all analyses. For AugPatho, we used the updated SAM files generated during its reassignment step, in which individual reads in some cases can be associated with multiple references. This format may improve the detection of expected references but can also introduce ambiguity, potentially contributing to higher false-positive rates. On the new simulated datasets, MADRe achieved up to a 28% improvement over other state-of-the-art methods when no clustering of similar strains was applied, and up to a 10% improvement when clustering was used. A similar trend was observed for the more complex large sized simulated datasets. Although MADRe occasionally missed low-abundance strains in these datasets, it still produced more accurate classifications than competing tools. This is particularly important since MADRe focuses on precise read-level classification rather than on abundance estimation.

A major challenge in metagenomic evaluation is the scarcity of realistic benchmark datasets, which can lead to parameter overfitting across methods, often to well-known datasets such as the Zymo communities. This may explain observations like those in the D6331 ONT dataset, where MORA and AugPatho showed substantial discrepancies between BC distances calculated from all classified reads and those derived only from true positives - the distances for true positives were notably higher. In contrast, MADRe consistently achieved better results than other tools for both evaluation types, demonstrating robust classification performance.

One limitation of MADRe observed in the Zymo benchmarks is the higher number of false-negative identifications, largely stemming from low-abundance organisms that are difficult to capture through the assembly-based approach, particularly when these organisms belong to species represented by a large number of closely related strains in the reference database. However, a similar effect can be seen in AugPatho's final reports, which include only abundance estimates from the last reassignment step - these also exhibit increased false negatives. This highlights a broader issue in metagenomic classification: setting thresholds for reporting low-abundance taxa inevitably trades off between reducing false positives and increasing false negatives [68]. The identification and quantification of low-abundance organisms remain challenging problems. MADRe does not apply any automatic post-filtering, leaving the decision of whether to perform additional filtering or manual investigation of low-abundance taxa to the user.

A closer look at the composition of the Zymo datasets and the definitions of ground-truth labels provides additional insight into the observed differences in tool performance. We can clearly observe performance variation across the three Zymo datasets, which can be attributed to both the sequencing technology and the evaluation methodology. As expected, the D6331 HiFi dataset yielded the best results, reflecting the higher base-level accuracy of HiFi reads compared to ONT. At first glance, it may seem surprising that performance on D6322 ONT was lower than on D6331 ONT, since D6322 contains species from different genera and should, in principle, be easier to classify. The main factor explaining this discrepancy lies in how ground-truth labels were defined. For D6322, the evaluation was straightforward - each genome represented a distinct species, and thus, an exact species-level match was required for a correct classification. In contrast, D6331 includes five E. coli genomes, three of which have very high sequence identity (greater than 99.3% ANI score - calculated using fastANI). When constructing the ground

truth for D6331, we clustered these three genomes and considered a read originating from any of them as correctly classified if it was assigned to any genome within that cluster. This less stringent criterion results in higher apparent performance for D6331 compared to D6322, an effect that applies uniformly across all evaluated tools.

In the real metagenomic dataset, MADRe classified fewer strains and focused on a confident subset of dominant organisms. In contrast, MADRe_RC, MORA, AugPatho, and Centrifuger reported a much larger number of low-abundance strains. While this may suggest higher sensitivity, many of these additional detections are likely spurious strain-level assignments, particularly in cases where the data do not support precise strain resolution. In this dataset, certain true references were absent from the database. Under these conditions, AugPatho tended to favor longer, highly similar genomes, MORA and MADRe_RC dispersed reads across multiple low-abundance strains, whereas MADRe mostly assigned reads to the reference sharing the greatest number of similar regions with the true organism.

MADRe's reduced detection of low-abundance strains arises primarily from limitations inherent to assembly-based approaches. At very low coverage, strains may fail to assemble or may produce contigs that are too short or fragmented to provide sufficient strain-specific signal for confident detection during database reduction. As a result, low-abundance strains may be excluded early in the workflow. This effect can be further amplified when closely related, higher-abundance strains are present, as assembly may preferentially represent the dominant genome, reducing the amount of discriminative sequence available for reliable separation.

These observations reflect an intentional precision–recall tradeoff in the design of MADRe. By reducing the reference database, MADRe prioritizes minimizing false-positive strain detections. At the genome-identification level, this may lead to reduced recall for extremely low-abundance strains. However, at the read level, the impact is typically limited because such strains contribute only a small fraction of total reads. When a strain is not retained during database reduction, its reads are frequently assigned to a highly similar reference, resulting in stable recall but a potential reduction in precision. From a biological perspective, this behavior is advantageous in scenarios where false-positive strain detection carries greater consequences than missing very low-abundance organisms.

As noted above, challenges extend beyond low-abundance effects and arise when both the reference database and the analyzed dataset contain genomes exhibiting extremely high sequence similarity. This was investigated through a dedicated high-similarity stress test (Supplementary File: Similar Strains Experiment; Supplementary Table ST23) and additional synthetic mixtures spanning combinations of similarity and coverage depth (Supplementary File: Coverage and Similarity Experiment; Supplementary Table ST24). These experiments demonstrate that when sequence similarity exceeds 99.9% ANI, strain-level discrimination becomes intrinsically ambiguous, largely independent of coverage. While very low coverage can limit detectability, the dominant constraint in these regimes is sequence similarity rather than abundance. Under such conditions, all evaluated methods approach fundamental resolution limits, although they differ in how ambiguity is handled. MADRe addresses this ambiguity by consistently assigning reads to the most similar available reference (the centroid), yielding stable behavior even when exact strain-level resolution is not supported by the data.

These observations also emphasize a broader limitation of current long-read metagenomic classifiers: all existing methods struggle to resolve strains at extremely low sequence divergence. For this reason, in our evaluation we additionally report results at the cluster level, where highly similar genomes are grouped together based on their mapping profiles. This approach avoids penalizing tools for inevitable redistribution within such groups and provides a more biologically meaningful measure of performance. Unlike conventional clustering by average nucleotide identity (ANI), our method groups references according to shared mapping profiles, focusing on patterns reflected in the data rather than static reference similarity. This design supports the concept of sample-aware reference groups that better capture functional and ecological relationships and could enhance classification accuracy in the presence of closely related organisms. Such clustering could also guide adaptive reference construction or real-time database refinement as additional samples are analyzed. Although clustering was used only for evaluation in this study and applied uniformly across all tools, future work will include deeper investigation of this method and its integration into the full MADRe pipeline.

As MADRe relies on metagenome assembly for database reduction, its performance can be influenced by assembly quality, particularly in highly complex or low-coverage samples. To assess sensitivity to assembler choice, we evaluated MADRe on large simulated datasets using Myloasm in addition to metaFlye and metaMDBG. Overall performance trends were comparable across assemblers, suggesting that MADRe is not strongly dependent on a specific assembly tool. We observed complementary behavior, with Myloasm showing improved detection of low-abundance strains and metaFlye performing slightly better for highly abundant ones. Strain-level assemblers were not considered, as they typically generate shorter and more fragmented contigs, whereas MADRe's reduction algorithm benefits from longer genomic context. While different parameter settings may influence assembly quality, all assemblers were executed using recommended configurations, and extensive parameter tuning was beyond the scope of this study.

Beyond strain-level classification, MADRe's modular design, particularly its database reduction and probabilistic reassignment components, offers potential for broader applications. These include contig binning, assembly refinement, and functional gene profiling, where confident reference reduction and ambiguity-aware read handling are equally valuable.

Conclusion

In this study, we introduced MADRe, a novel pipeline for strain-level metagenomic classification of long-read sequencing data. MADRe combines long-read assembly, EM-based contig-to-reference mapping reassignment for database reduction, and probabilistic read reassignment to deliver accurate and efficient classification, even without prior knowledge of sample composition. Unlike many existing tools, MADRe is designed to operate with large, diverse databases spanning multiple taxonomic levels, enabling high-resolution classification while minimizing false positives.

The pipeline consists of two distinct steps: database reduction and read classification, both of which can be executed independently. If general insight into the strains present in a sample is required, the first step can be used alone. Conversely, when prior knowledge about the sample exists, or when a reduced reference set is already available, the read classification step can be applied independently. MADRe provides a practical, scalable, and modular solution for strain-level classification in complex microbial communities.

Methods

MADRe Database Reduction

The database reduction step, shown in Figure 1 and illustrated in more detail in Supplementary Figure S1, consists of two main phases: input file preparation and the database reduction.

In the input file preparation phase, raw long metagenomic reads are first assembled using metaFlye for ONT reads or metaMDBG for HiFi reads. When multiple strains of the same species are present in

a sample, the assembly process can lead to strain collapse, producing contigs that represent a blend of closely related strains rather than distinct strain-specific sequences. Instead of using strain-aware metagenome assemblers, which typically generate shorter contigs, we chose to retain the longer contigs and infer strain-level complexity using HairSplitter functionality which estimates the number of collapsed strains per contig.

Assembled contigs are mapped to the reference database using Minimap2 with the *asm5* parameter preset, generating a PAF file as output. We chose this preset because, compared to *asm10* and *asm20*, it provides higher sensitivity, which is crucial for capturing more accurate and complete alignments of contigs to highly similar reference genomes. The MADRe database reduction process takes two key inputs: the estimated number of collapsed strains per contig determined by HairSplitter and the contig-to-reference mappings from Minimap2.

The database reduction process is based on the EM algorithm, which reassigns contigs to different references while performing soft clustering, allowing a single contig to be assigned to multiple references with different probabilities. The EM algorithm is widely used for handling ambiguous mappings in metagenomic classification [29, 30, 31, 32, 45, 69]. The implementation of the EM algorithm in MADRe is inspired by PathoScope2 [30] and EMU [31].

The database reduction process consists of three main steps. We can define a set of contigs as $C = \{c_1, c_2, \ldots, c_x\}$, where x is the number of contigs in the assembly. The set of references to which at least one contig is mapped is defined as $R = \{r_1, r_2, \ldots, r_g\}$, where g is the number of references. Additionally, let M represent the set of all of the mappings. In the first step, we compute a mapping score H for each mapping in the PAF file using the equation:

$$\mathrm{H} = 2 * \frac{\mathrm{N} \times ml}{\mathrm{N} + ml} \quad (1)$$

which represents the harmonic mean between the exact number of matches N and the mapping length *ml*. The *ml* is defined as the maximum value between the query mapping length and the reference mapping length. Applying the harmonic mean allows us to emphasize the smaller value, ensuring that a mapping does not receive an inflated score due, for example, to a very long but low-quality alignment.

The summarized mapping value S is then calculated for each contig-reference pair using:

$$\mathrm{S}(c_i, r_j) = \sum_{m \in M(c_i, r_j)} \mathrm{H}(p) \quad (2)$$

where $M(c_i, r_j)$ represents the set of mappings of contig c_i to reference r_j. This ensures that $\mathrm{S}(c_i, r_j)$ is computed by summing the mapping values of all instances where c_i maps to r_j, thus capturing all possible alignments between the contig and the reference. Following this, we divided mappings into *unique* and *non-unique*. Unique mappings occur when a contig maps exclusively to a single reference, whereas non-unique mappings represent ambiguous cases that require further resolution.

In the second step, non-unique mappings are processed using the EM algorithm, which iteratively refines contig assignments based on mapping probabilities. The E-step updates the expected assignments of contigs, while the M-step re-estimates the parameters using the newly computed assignment probabilities from the previous iteration.

The probability of selecting a reference r_i is given by:

$$P(r_i) = \frac{1}{G}, \quad \text{where} \quad G = |R| \quad (3)$$

The conditional probability of c_i given r_i is expressed as:

$$P(c_i | r_i) = \frac{\mathrm{S}(c_i, r_i)}{\max_{r_j \in R} \mathrm{S}(c_i, r_j)} \quad (4)$$

The log-likelihood function $L(X)$ is given by:

$$L(C) = \sum_{i=1}^{X} \log \left(\sum_{j=1}^{G} P(c_i \mid r_j) \cdot P(r_j) \right) \quad (5)$$

The expectation step (E-step) updates the posterior probability $P(r_i \mid c_i)$ as follows:

$$P(r_i \mid c_i) = \frac{P(c_i \mid r_i) \cdot P(r_i)}{\sum_{j=1}^{G} P(c_i \mid r_j) \cdot P(r_j)} \quad (6)$$

The maximization step (M-step) updates the prior probability $P(r_i)$ as follows:

$$P(r_i) = \frac{\sum_{j=1}^{X} P(r_i \mid c_j)}{|M|} \quad (7)$$

The EM algorithm runs iteratively until it converges or reaches the maximum number of iterations set by the stopping criteria. Once the algorithm outputs posterior probabilities, these values are used to determine which references will be included in the reduced database.

Before selecting references, we first classify each contig at the species level. This is done by summing the posterior probabilities across all references belonging to a species and assigning the contig to the species with the highest total probability. After determining the species classification, we retain only posterior probabilities associated with references belonging to the selected species. Finally, for each contig, we select N + 2 reference genomes to include in the reduced database. The value of N is estimated based on the number of collapsed strains identified by HairSplitter. By default, MADRe adds two additional references to avoid excluding expected strains, although this offset can be adjusted through user parameters.

MADRe Read Classification

The read classification step in MADRe is designed to operate both with and without prior database reduction. The only requirement is a PAF file, the Minimap2 output containing read-to-database mappings, where each database sequence includes the corresponding taxonomic identifier. The MADRe read classification workflow is illustrated in Supplementary Figure S2.

The process begins by computing a mapping score for each alignment, defined as the ratio between the number of exact matches (N) and the mapping length (ml):

$$S = \frac{N}{ml}$$

Mappings are then categorized into unique and non-unique. Since a read can have multiple alignments, only the alignment with the highest score is retained for each read-reference pair. If a read has a single best-scoring mapping, it is classified as a unique mapping. Conversely, if multiple mappings share the same highest score, they are considered non-unique, and the read will go through a reassignment process. Formally:

$$\text{Unique if } S_{r,i} = \max_j (S_{r,j}) \text{ and this maximum is unique;}$$

$$\text{Non-unique if } S_{r,i} = \max_j(S_{r,j}) \text{ for two or more } j.$$

Before reassigning non-unique mappings, reads are first classified at the species level. For each read r, the maximum mapping score among all references belonging to a species s is computed as:

$$S_{r,s}^{\max} = \max_{i \in s}(S_{r,i})$$

The species with the highest $S_{r,s}^{\max}$ is selected as the species-level assignment for that read. Although the default assumption is that a read can be uniquely mapped to a single species but may map ambiguously to multiple strains within that species, this assumption does not always hold. In rare cases, two genomes from different species may share highly similar regions, making it difficult to determine the true origin of a read. In such situations, reads are randomly distributed between the corresponding species. These cases are uncommon and typically arise from taxonomic inconsistencies, for example when nearly identical strains according to taxonomy belong to different species.

To reassign non-unique mappings at the strain level, a species-specific clustering algorithm is applied. This algorithm evaluates the number of unique and non-unique mappings associated with each reference within the same species, identifying groups of references that share many mappings, indicating that they likely represent overlapping genomic regions and should form clusters.

The fundamental assumption is that a reference truly present in the sample should accumulate the highest number of mappings (both unique and high-confidence non-unique). Let M_i denote the total number of mappings to reference i:

$$M_i = U_i + N_i$$

where U_i and N_i represent the counts of unique and non-unique mappings, respectively. The expected references are identified as those with the highest M_i within each cluster, and all non-unique reads are reassigned to the most probable reference in that cluster:

$$r \in \text{cluster}(i) \Rightarrow r \to \arg \max_{j \in \text{cluster}(i)} M_j$$

This procedure ensures that ambiguous reads are redistributed toward references that are both well supported by unique evidence and consistent with the mapping structure observed across the sample. By reassigning reads to the most representative reference within each cluster, this methodology establishes MADRe's centroid-based behavior, maintaining stable and interpretable classifications even in the presence of highly similar strains.

Abundances calculation

At this stage, each read is assigned to a single reference genome. Based on these assignments, MADRe calculates the abundance of each detected strain. The primary abundance output file reports the number of reads assigned to each reference. However, MADRe also provides an option to compute a length-normalized abundance, which accounts for both read and reference lengths. This alternative abundance metric is calculated as:

$$\text{Abundance}(r) = \frac{\sum_{i \in \text{Reads}_r} \mathit{Length}(i)}{\text{Length}(r)} \quad \text{for } r \in R \qquad (8)$$

where R is the set of the references and $Reads_r$ is set of the reads classified under reference r.

Similar strains clustering

The high similarity between closely related strains and the lack of a clear threshold for defining when two sequences represent the same strain, makes it difficult to ensure that a reference database contains only unique strain sequences [34]. Some entries may correspond to highly similar strains or even to multiple assemblies of the same strain. To address this, MADRe includes an optional reference clustering step within the read classification process, designed to group similar references based on shared read mappings.

This clustering step operates on the same mapping file used in the classification stage and produces two output files: one reporting the abundances of identified clusters and the other listing the representative reference for each cluster.

The clustering procedure is illustrated in Supplementary Figure S3. It begins by using the calculated mapping scores and species-level labels. For each species-specific subset, the algorithm identifies the highest-quality mapping for each read. A binary vector is constructed for each reference, where each bit indicates whether a given read strongly supports that reference. These binary vectors are then clustered using DBSCAN with precomputed Jaccard distances, eps = 0.9, and min_samples = 1. Cluster-level abundances are computed accordingly. As a result, the post-clustering abundance files report only the representative references for each cluster.

Evaluation details

Our evaluation is primarily focused on exact read-level taxonomic assignments and read count-based abundance estimates.

To ensure a fair comparison, all tools were benchmarked using the same large reference database. For tools requiring mapping files as input, namely MORA, AugPatho, and MADRe_RC, we used a unified set of read-to-reference alignments generated by Minimap2. All reads were mapped to the full database, and the resulting SAM file was used directly for MORA and AugPatho. This SAM file was subsequently converted to PAF format using Paftools, as required by MADRe_RC.

Simulated reads were generated with the Badread tool [56], applying the whole-metagenome simulation mode with default values for chimeric, junk, and random reads. The simulation was performed using the *nanopore2023* model, which corresponds to the ONT R10.4.1. The exact command used is provided in the Supplementary File.

In case of simulated datasets, ground truth was available for every read, including its corresponding strain-level taxID and reference accession. Using this information, we computed true positives (TP), false positives (FP), true negatives (TN), and false negatives (FN) by comparing the assigned strain-level taxIDs with the expected ones. For Kraken2, we extracted read IDs and assigned taxIDs from its output. If a read was assigned to a higher taxonomic level, even if it was taxonomic correct, we treated it as a false positive, as the evaluation strictly focused on strain-level classification.

For the simulated datasets and Zymo mock communities we calculated Bray-Curtis (BC) distance as:

$$\text{BC}(x, y) = \frac{\sum_{i=1}^{n} |x_i - y_i|}{\sum_{i=1}^{n} (x_i + y_i)} \qquad (9)$$

Where $x = (x_1, x_2, \ldots, x_n)$ and $y = (y_1, y_2, \ldots, y_n)$ are the abundance vectors for two samples or profiles, n is the number of strains, x_i and y_i are the abundances of the ith strain in samples x and y, respectively. $\text{BC}(x, y)$ is the Bray-Curtis dissimilarity or distance, ranging from 0 (identical composition) to 1 (completely disjoint).

In the case of medium-sized simulated datasets (sim_small, sim_medium and sim_expanded) and the real dataset, the evaluation was also performed at the species level (results presented in Supplementary Table ST8 and Supplementary Figure S7). All strain-

level classifications were uplifted to their corresponding species, and read count abundances were calculated. For Kraken2, we used the species-level abundances reported in its summary file, limited to entries labeled with an "S" (species rank).

MADRe, MADRe_RC, and MORA each produce read-level classification files that associate each read with a reference genome. Since all tools shared the same mapping input, for AugPatho we ran PathoID and PathoREPORT steps, which output an updated SAM files and a report containing reference abundance estimates. In this updated SAM files, a single read can be associated with multiple references. For evaluation purposes, we allowed such multi-reference assignments, which may slightly benefit AugPatho by increasing the number of true positives, while also increasing the risk of false positives. These trade-offs are largely neutralized when clustering is applied, as similar strains typically end up grouped in the same cluster. With Centrifuger we encountered one limitation - Centrifuger cannot confidently assign a read to a specific reference sequence (e.g., when multiple chromosomes belong to the same strain), it often classifies the read under the NCBI strain-level taxid. In some cases, this strain taxid is identical to the species taxid, making it impossible to directly and fairly compare such classifications to those of other tools that operate at the sequence level. For benchmarking consistency, we therefore considered as true positives only the reads correctly classified under the expected reference sequence. It is important to note that this issue affected a relatively small fraction of reads (approximately 9000 out of 5 million reads in the 1000-genome dataset).

We used the same clustering across all tools to ensure consistency in cluster-based evaluation. Our clustering is based on read-to-reference mapping profiles, which can differ depending on the size and composition of the database. For example, when reads are mapped to a reduced database, the absence of certain similar references can make ambiguous mappings more resolvable. To avoid such inconsistencies, clustering was performed only once on the PAF file used for MADRe_RC, which contains read mappings to the complete reference database. During cluster-level evaluation, a classification was considered a true positive if the read was assigned to a reference that belongs to the same cluster as the ground truth reference:

$$\mathrm{TP} = \begin{cases} 1, & \text{if } C(\hat{r}) = C(r_{\mathrm{true}}) \\ 0, & \text{otherwise} \end{cases}$$

where $C(\hat{r})$ denotes the cluster of the predicted reference and $C(r_{\mathrm{true}})$ denotes the cluster of the true reference. A classification is considered a true positive (TP) if both references belong to the same cluster.

All commands used to perform classification with the evaluated tools are listed in the Supplementary File.

Availability of source code and requirements

- Project name: MADRe - Metagenome Assembly driven Database Reduction
- Project home page: https://github.com/lbcb-sci/MADRe
- Operating system(s): UNIX
- Programming language: Python
- Other requirements: Environment Modules, Conda, see https://github.com/lbcb-sci/MADRe
- License: MIT

Data availability

The source code for MADRe is available at [70]. Simulated data can be accessed via Zenodo [71]. Zymo D6322 ONT dataset is obtained from BioProject PRJNA1240873, zymo D6331 ONT dataset is obtained from [58], and zymo D6331 PacBio HiFi dataset from [59].

Additional files

SupplementaryFile.pdf; SupplementaryTables.xlsx

Declarations

List of abbreviations

EM: expectation-maximization; TP: true positive; TN: true negative; FP: false positive; FN: false negative; BC: Bray-Curtis; ANI: average nucleotide identity; ONT: Oxford Nanopore Technologies; taxID: taxonomy identifier.

Competing Interests

M.Š. has been jointly funded by Oxford Nanopore Technologies and AI Singapore for the project AI-driven De Novo Diploid Assembler. The remaining authors declare no competing interests.

Funding

This work was supported by the Croatian Science Foundation under grants IP-2018-01-5886 (SIGMA), DATACROSS (2024-2026, PK.1.1.10.0007), MOBDOK-2023-2941, and by the Singapore Ministry of Health's National Medical Research Council, Singapore, under the grant MOH-000649-01 (Rapid diagnostic of infectious diseases based on nanopore sequencing and AI methods) – Individual Research Grant (NMRC/OFIRG/MOH-000649-00).

Acknowledgments

The authors thank Lune Angevin for testing the tool and providing valuable feedback.

Author's Contributions

K.K. and M.Š. conceived the study. J.L. designed and implemented the pipeline. K.K. supervised database reduction implementation. R.V. supervised read classification implementation. J.L. drafted the manuscript. K.K., R.V. and M.Š. revised the manuscript. All authors read and approved the manuscript.

References

1. Gilbert JA, Blaser MJ, Caporaso JG, Jansson JK, Lynch SV, Knight R. Current understanding of the human microbiome. Nature medicine 2018;24(4):392–400. https://doi.org/10.1038/nm.4517.
2. Ling LL, Schneider T, Peoples AJ, Spoering AL, Engels I, Conlon BP, et al. A new antibiotic kills pathogens without detectable resistance. Nature 2015;517(7535):455–459. https://doi.org/10.1038/nature14098.
3. Arikan M, Muth T. gNOMO2: a comprehensive and modular pipeline for integrated multi-omics analyses of microbiomes. GigaScience 2024;13:giae038. https://doi.org/10.1093/gigascience/giae038.
4. Lu J, Rincon N, Wood DE, Breitwieser FP, Pockrandt C, Langmead B, et al. Metagenome analysis using the Kraken software suite. Nature Protocols 2022;17(12):2815–2839. https://doi.org/10.1038/s41596-022-00738-y.

5. Zhang W, Liu Y, Li G, Xu J, Chen E, Schönhuth A, et al. Strain-level metagenomic profiling using pangenome graphs with PanTax. Genome Research 2026;36(2):405–420. https://doi.org/10.1101/gr.280858.125.
6. Truong DT, Franzosa EA, Tickle TL, Scholz M, Weingart G, Pasolli E, et al. MetaPhlAn2 for enhanced metagenomic taxonomic profiling. Nature Methods 2015;12(10):902–903. https://doi.org/10.1038/nmeth.3589.
7. Blanco-Míguez A, Beghini F, Cumbo F, McIver LJ, Thompson KN, Zolfo M, et al. Extending and improving metagenomic taxonomic profiling with uncharacterized species using MetaPhlAn 4. Nature Biotechnology 2023;41(11):1633–1644. https://doi.org/10.1038/s41587-023-01688-w.
8. Ruscheweyh HJ, Milanese A, Paoli L, Sintsova A, Mende DR, Zeller G, et al. mOTUs: profiling taxonomic composition, transcriptional activity and strain populations of microbial communities. Current Protocols 2021;1(8):e218. https://doi.org/10.1002/cpz1.218.
9. Chen X, Yin X, Shi X, Yan W, Yang Y, Liu L, et al. Melon: metagenomic long-read-based taxonomic identification and quantification using marker genes. Genome Biology 2024;25(1):226. https://doi.org/10.1186/s13059-024-03363-y.
10. Costea PI, Munch R, Coelho LP, Paoli L, Sunagawa S, Bork P. metaSNV: a tool for metagenomic strain level analysis. PLOS ONE 2017;12(7):e0182392. https://doi.org/10.1371/journal.pone.0182392.
11. Olm MR, Crits-Christoph A, Bouma-Gregson K, Firek BA, Morowitz MJ, Banfield JF. inStrain profiles population microdiversity from metagenomic data and sensitively detects shared microbial strains. Nature Biotechnology 2021;39(6):727–736. https://doi.org/10.1038/s41587-020-00797-0.
12. Menzel P, Ng KL, Krogh A. Fast and sensitive taxonomic classification for metagenomics with Kaiju. Nature Communications 2016;7(1):11257. https://doi.org/10.1038/ncomms11257.
13. Buchfink B, Xie C, Huson DH. Fast and sensitive protein alignment using DIAMOND. Nature Methods 2015;12(1):59–60. https://doi.org/10.1038/nmeth.3176.
14. Steinegger M, Söding J. MMseqs2 enables sensitive protein sequence searching for the analysis of massive data sets. Nature Biotechnology 2017;35(11):1026–1028. https://doi.org/10.1038/nbt.3988.
15. Huson DH, Albrecht B, Bağcı C, Bessarab I, Gorska A, Jolic D, et al. MEGAN-LR: new algorithms allow accurate binning and easy interactive exploration of metagenomic long reads and contigs. Biology Direct 2018;13:1–17. https://doi.org/10.1186/s13062-018-0208-7.
16. Marić J, Križanović K, Riondet S, Nagarajan N, Šikić M. Comparative analysis of metagenomic classifiers for long-read sequencing datasets. BMC Bioinformatics 2024;25(1):15. https://doi.org/10.1186/s12859-024-05634-8.
17. Wood DE, Lu J, Langmead B. Improved metagenomic analysis with Kraken 2. Genome Biology 2019;20:257. https://doi.org/10.1186/s13059-019-1891-0.
18. Breitwieser FP, Baker DN, Salzberg SL. KrakenUniq: confident and fast metagenomics classification using unique k-mer counts. Genome biology 2018;19:1–10. https://doi.org/10.1186/s13059-018-1568-0.
19. Lu J, Breitwieser FP, Thielen P, Salzberg SL. Bracken: estimating species abundance in metagenomics data. PeerJ Computer Science 2017;3:e104. https://doi.org/10.7717/peerj-cs.104.
20. Kim D, Song L, Breitwieser FP, Salzberg SL. Centrifuge: rapid and sensitive classification of metagenomic sequences. Genome Research 2016;26(12):1721–1729. https://doi.org/10.1101/gr.210641.116.
21. Song L, Langmead B. Centrifuger: lossless compression of microbial genomes for efficient and accurate metagenomic sequence classification. Genome Biology 2024;25(1):106. https://doi.org/10.1186/s13059-024-03244-4.
22. Ounit R, Wanamaker S, Close TJ, Lonardi S. CLARK: fast and accurate classification of metagenomic and genomic sequences using discriminative k-mers. BMC genomics 2015;16:1–13. https://doi.org/10.1186/s12864-015-1419-2.
23. Ounit R, Lonardi S. Higher classification sensitivity of short metagenomic reads with CLARK-S. Bioinformatics 2016;32(24):3823–3825. https://doi.org/10.1093/bioinformatics/btw542.
24. Piro VC, Dadi TH, Seiler E, Reinert K, Renard BY. ganon: precise metagenomics classification against large and up-to-date sets of reference sequences. Bioinformatics 2020;36(Supplement 1):i12–i20. https://doi.org/10.1093/bioinformatics/btaa458.
25. Piro VC, Reinert K. ganon2: up-to-date and scalable metagenomics analysis. NAR Genomics and Bioinformatics 2025;7(3):lqaf094. https://doi.org/10.1093/nargab/lqaf094.
26. Ulrich JU, Renard BY. Fast and space-efficient taxonomic classification of long reads with hierarchical interleaved XOR filters. Genome Research 2024;34(6):914–924. https://doi.org/10.1101/gr.278623.123.
27. Shaw J, Yu YW. Rapid species-level metagenome profiling and containment estimation with sylph. Nature Biotechnology 2025;43(8):1348–1359. https://doi.org/10.1038/s41587-024-02412-y.
28. Dilthey AT, Jain C, Koren S, Phillippy AM. Strain-level metagenomic assignment and compositional estimation for long reads with MetaMaps. Nature Communications 2019;10(1):3066. https://doi.org/10.1038/s41467-019-10934-2.
29. Hong C, Manimaran S, Shen Y, Perez-Rogers JF, Byrd AL, Castro-Nallar E, et al. PathoScope 2.0: a complete computational framework for strain identification in environmental or clinical sequencing samples. Microbiome 2014;2:33. https://doi.org/10.1186/2049-2618-2-33.
30. Francis OE, Bendall M, Manimaran S, Hong C, Clement NL, Castro-Nallar E, et al. Pathoscope: species identification and strain attribution with unassembled sequencing data. Genome Research 2013;23(10):1721–1729. https://doi.org/10.1101/gr.150151.112.
31. Curry KD, Wang Q, Nute MG, Tyshaieva A, Reeves E, Soriano S, et al. Emu: species-level microbial community profiling of full-length 16S rRNA Oxford Nanopore sequencing data. Nature Methods 2022;19(7):845–853. https://doi.org/10.1038/s41592-022-01520-4.
32. Zheng A, Shaw J, Yu YW. Mora: abundance aware metagenomic read re-assignment for disentangling similar strains. BMC Bioinformatics 2024;25(1):161. https://doi.org/10.1186/s12859-024-05768-9.
33. Schaeffer L, Pimentel H, Bray N, Melsted P, Pachter L. Pseudoalignment for metagenomic read assignment. Bioinformatics 2017;33(14):2082–2088. https://doi.org/10.1093/bioinformatics/btx106.
34. Van Rossum T, Ferretti P, Maistrenko OM, Bork P. Diversity within species: interpreting strains in microbiomes. Nature Reviews Microbiology 2020;18(9):491–506. https://doi.org/10.1038/s41579-020-0368-1.
35. Luo C, Walk ST, Gordon DM, Feldgarden M, Tiedje JM, Konstantinidis KT. Genome sequencing of environmental Escherichia coli expands understanding of the ecology and speciation of the model bacterial species. Proceedings of the National Academy of Sciences 2011;108(17):7200–7205. https://doi.org/10.1073/pnas.1015622108.
36. Kashtan N, Roggensack SE, Rodrigue S, Thompson JW, Biller SJ, Coe A, et al. Single-cell genomics reveals hundreds of coexisting subpopulations in wild Prochlorococcus. Science 2014;344(6182):416–420. https://doi.org/10.1126/science.1248575.
37. Schloissnig S, Arumugam M, Sunagawa S, Mitreva M, Tap J, Zhu A, et al. Genomic variation landscape of the human gut

microbiome. Nature 2013;493(7430):45–50. https://doi.org/10.1038/nature11711.

38. Yassour M, Jason E, Hogstrom LJ, Arthur TD, Tripathi S, Siljander H, et al. Strain-level analysis of mother-to-child bacterial transmission during the first few months of life. Cell Host & Microbe 2018;24(1):146–154. https://doi.org/10.1016/j.chom.2018.06.007.

39. Yang Y, Dufault-Thompson K, Yan W, Cai T, Xie L, Jiang X. Large-scale genomic survey with deep learning-based method reveals strain-level phage specificity determinants. GigaScience 2024;13:giae017. https://doi.org/10.1093/gigascience/giae017.

40. van Dijk LR, Walker BJ, Straub TJ, Worby CJ, Grote A, Schreiber IV HL, et al. StrainGE: a toolkit to track and characterize low-abundance strains in complex microbial communities. Genome Biology 2022;23(1):74. https://doi.org/10.1186/s13059-022-02630-0.

41. Albanese D, Donati C. Strain profiling and epidemiology of bacterial species from metagenomic sequencing. Nature Communications 2017;8(1):2260. https://doi.org/10.1038/s41467-017-02209-5.

42. Roosaare M, Vaher M, Kaplinski L, Möls M, Andreson R, Lepamets M, et al. StrainSeeker: fast identification of bacterial strains from raw sequencing reads using user-provided guide trees. PeerJ 2017;5:e3353. https://doi.org/10.7717/peerj.3353.

43. Siekaniec G, Roux E, Lemane T, Guédon E, Nicolas J. Identification of isolated or mixed strains from long reads: a challenge met on Streptococcus thermophilus using a MinION sequencer. Microbial Genomics 2021;7(11):000654. https://doi.org/10.1099/mgen.0.000654.

44. Dempster AP, Laird NM, Rubin DB. Maximum likelihood from incomplete data via the EM algorithm. Journal of the Royal Statistical Society: Series B (Methodological) 1977;39(1):1–22. https://doi.org/10.1111/j.2517-6161.1977.tb01600.x.

45. Skoufos G, Almodaresi F, Zakeri M, Paulson JN, Patro R, Hatzigeorgiou AG, et al. AGAMEMNON: an Accurate metaGenomics And MEtatranscriptoMics quaNtificatiON analysis suite. Genome Biology 2022;23(1):39. https://doi.org/10.1186/s13059-022-02610-4.

46. Gao Y, Luo H, Lyu H, Yang H, Yousuf S, Huang S, et al. Benchmarking short-read metagenomics tools for removing host contamination. GigaScience 2025;14:giaf004. https://doi.org/10.1093/gigascience/giaf004.

47. Jain C, Rodriguez-R LM, Phillippy AM, Konstantinidis KT, Aluru S. High throughput ANI analysis of 90K prokaryotic genomes reveals clear species boundaries. Nature Communications 2018;9(1):5114. https://doi.org/10.1038/s41467-018-07641-9.

48. Koslicki D, White S, Ma C, Novikov A. YACHT: an ANI-based statistical test to detect microbial presence/absence in a metagenomic sample. Bioinformatics 2024;40(2):btae047. https://doi.org/10.1093/bioinformatics/btae047.

49. Anyansi C, Straub TJ, Manson AL, Earl AM, Abeel T. Computational methods for strain-level microbial detection in colony and metagenome sequencing data. Frontiers in Microbiology 2020;11:1925. https://doi.org/10.3389/fmicb.2020.01925.

50. LaPierre N, Alser M, Eskin E, Koslicki D, Mangul S. Metalign: efficient alignment-based metagenomic profiling via containment min hash. Genome Biology 2020;21(1):242. https://doi.org/10.1186/s13059-020-02159-0.

51. Koslicki D, Zabeti H. Improving minhash via the containment index with applications to metagenomic analysis. Applied Mathematics and Computation 2019;354:206–215. https://doi.org/10.1016/j.amc.2019.02.018.

52. Faure R, Lavenier D, Flot JF. HairSplitter: haplotype assembly from long, noisy reads. Peer Community Journal 2024;4. https://doi.org/10.24072/pcjournal.481.

53. Kolmogorov M, Bickhart DM, Behsaz B, Gurevich A, Rayko M, Shin SB, et al. metaFlye: scalable long-read metagenome assembly using repeat graphs. Nature Methods 2020;17(11):1103–1110. https://doi.org/10.1038/s41592-020-00971-x.

54. Benoit G, Raguideau S, James R, Phillippy AM, Chikhi R, Quince C. High-quality metagenome assembly from long accurate reads with metaMDBG. Nature Biotechnology 2024;42(9):1378–1383. https://doi.org/10.1038/s41587-023-01983-6.

55. Shaw J, Marin MG, Li H. High-resolution metagenome assembly for modern long reads with myloasm. bioRxiv 2025;https://doi.org/10.1101/2025.09.05.674543.

56. Wick RR. Badread: simulation of error-prone long reads. Journal of Open Source Software 2019;4(36):1316. https://doi.org/10.21105/joss.01316.

57. Zhang W, Benchmarking datasets used in the manuscript "Strain-level metagenomic profiling using pangenome graphs with PanTax". Zenodo; 2025. https://zenodo.org/records/16885808, version v3; accessed 2025-10-19.

58. Liu L, Yang Y, Deng Y, Zhang T. Nanopore long-read-only metagenomics enables complete and high-quality genome reconstruction from mock and complex metagenomes. Microbiome 2022;10(1):209. https://doi.org/10.1186/s40168-022-01415-8.

59. Portik DM, Brown CT, Pierce-Ward NT. Evaluation of taxonomic classification and profiling methods for long-read shotgun metagenomic sequencing datasets. BMC Bioinformatics 2022;23(1):541. https://doi.org/10.1186/s12859-022-05103-0.

60. Sereika M, Kirkegaard RH, Karst SM, Michaelsen TY, Sørensen EA, Wollenberg RD, et al. Oxford Nanopore R10.4 long-read sequencing enables the generation of near-finished bacterial genomes from pure cultures and metagenomes without short-read or reference polishing. Nature Methods 2022;19(7):823–826. https://doi.org/10.1038/s41592-022-01539-7.

61. O'Leary NA, Wright MW, Brister JR, Ciufo S, Haddad D, McVeigh R, et al. Reference sequence (RefSeq) database at NCBI: current status, taxonomic expansion, and functional annotation. Nucleic Acids Research 2016;44(D1):D733–D745. https://doi.org/10.1093/nar/gkv1189.

62. Bray JR, Curtis JT. An ordination of the upland forest communities of southern Wisconsin. Ecological Monographs 1957;27(4):326–349. https://doi.org/10.2307/1942268.

63. Zymo Research, ZymoBIOMICS: Microbiomics Solutions; 2025. https://www.zymoresearch.com/pages/zymobiomics, accessed: 2025-03-14.

64. Feng X, Cheng H, Portik D, Li H. Metagenome assembly of high-fidelity long reads with hifiasm-meta. Nature Methods 2022;19(6):671–674. https://doi.org/10.1038/s41592-022-01478-3.

65. Vicedomini R, Quince C, Darling AE, Chikhi R. Strainberry: automated strain separation in low-complexity metagenomes using long reads. Nature Communications 2021;12(1):4485. https://doi.org/10.1038/s41467-021-24515-9.

66. Luo X, Kang X, Schönhuth A. Enhancing long-read-based strain-aware metagenome assembly. Frontiers in Genetics 2022;13:868280. https://doi.org/10.3389/fgene.2022.868280.

67. Kazantseva E, Donmez A, Frolova M, Pop M, Kolmogorov M. Strainy: phasing and assembly of strain haplotypes from long-read metagenome sequencing. Nature Methods 2024;21(11):2034–2043. https://doi.org/10.1038/s41592-024-02424-1.

68. Simon HY, Siddle KJ, Park DJ, Sabeti PC. Benchmarking metagenomics tools for taxonomic classification. Cell 2019;178(4):779–794. https://doi.org/10.1016/j.cell.2019.07.010.

69. Sapoval N, Liu Y, Curry KD, Kille B, Huang W, Kokroko N, et al. Lightweight taxonomic profiling of long-read metagenomic

datasets with Lemur and Magnet. bioRxiv 2024;https://doi.org/10.1101/2024.06.01.596961.

70. Lipovac J, Lab Š, MADRe: Metagenome Assembly-Driven Database Reduction [Data set]. Zenodo.; 2026. https://github.com/lbcb-sci/MADRe, gitHub repository.
71. Lipovac J, Šikić Lab, MADRe: Metagenome Assembly-Driven Database Reduction; 2026. https://doi.org/10.5281/zenodo.18934466.

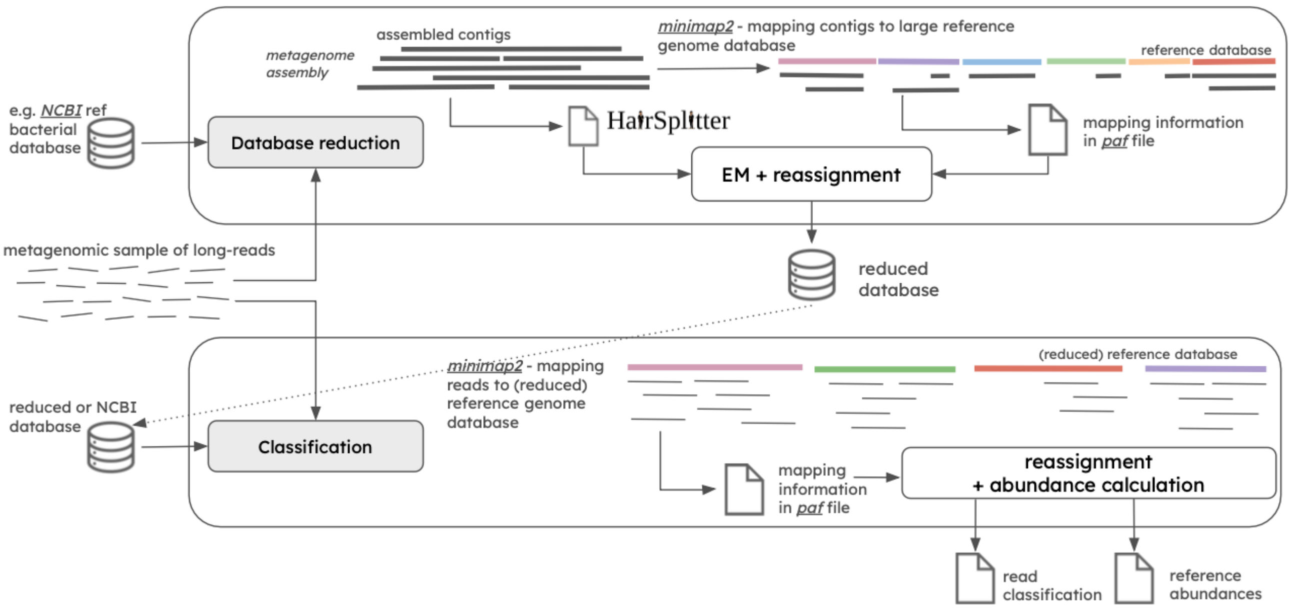

Figure 1. MADRe overall pipeline The first step of the pipeline performs database reduction using an EM-based contig-to-reference mapping procedure to identify organisms present in the sample. The second step involves read classification, which applies probabilistic read reassignment based on mapping information.

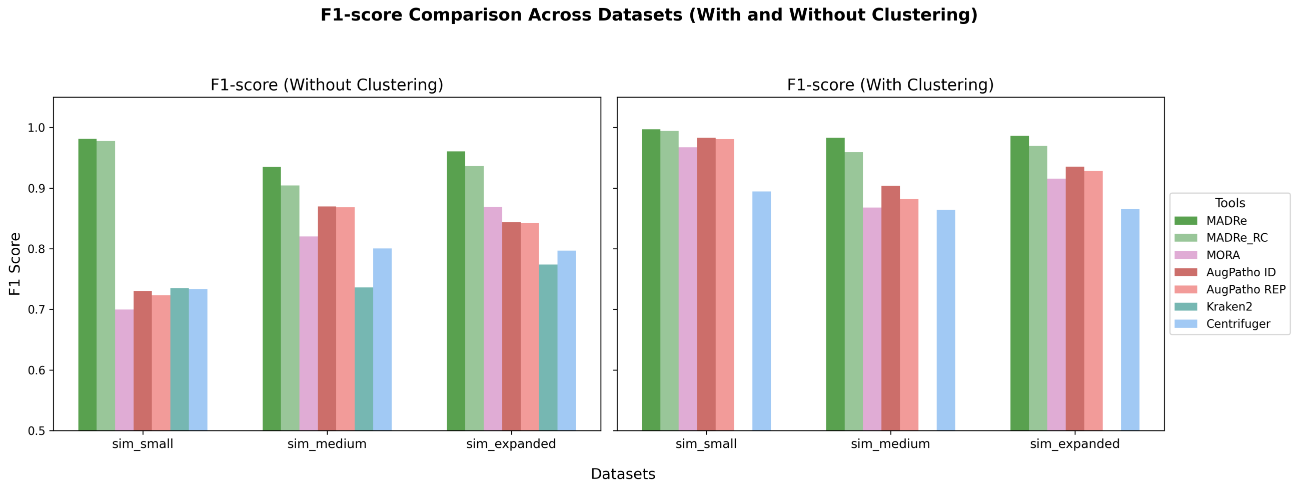

Figure 2. F1 scores of strain-level classification on medium-sized simulated reads, shown with and without post-clustering (grouping highly similar strains). Kraken2 results were omitted from the clustering analysis, as its output format does not support proper clustering.

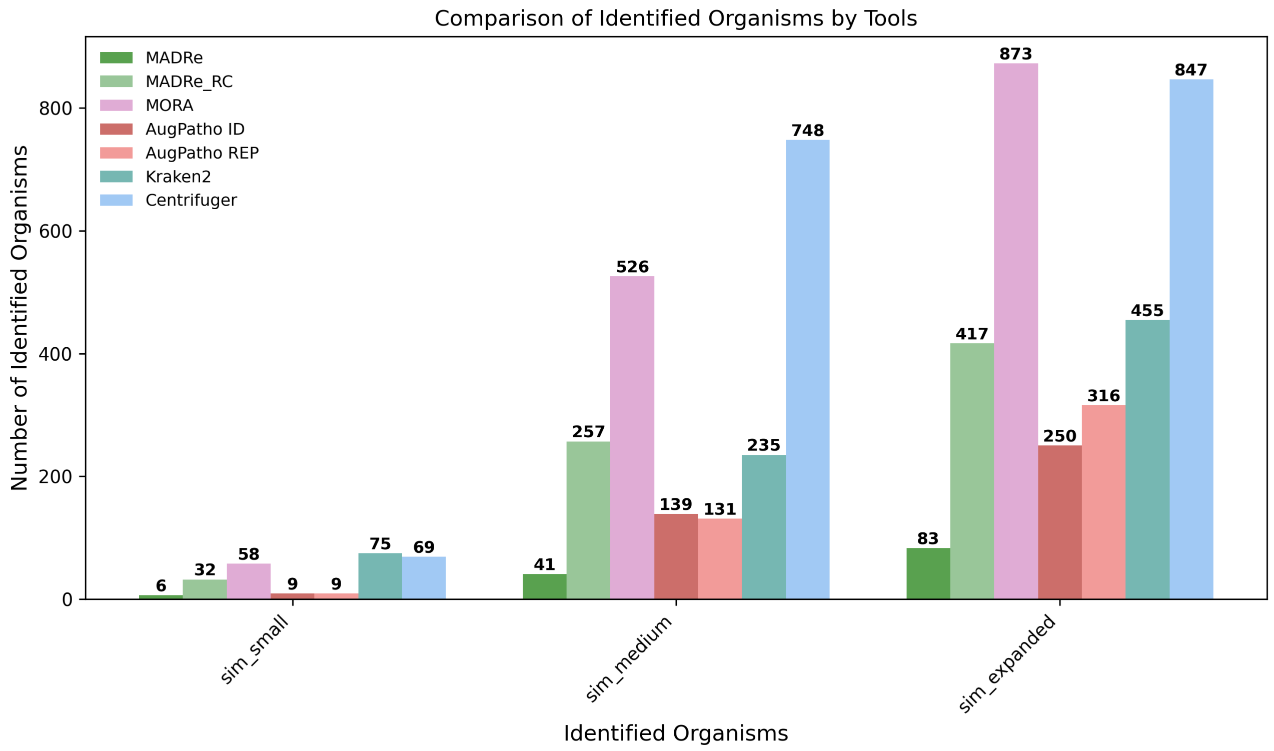

Figure 3. Number of identified organisms by different tools on simulated reads. An organism is considered identified if at least one read is classified under it. The sim_small dataset contains 4 strains, sim_medium contains 15 strains, and sim_expanded contains 30 strains. All tools successfully identified all expected strains, resulting in no false negatives.

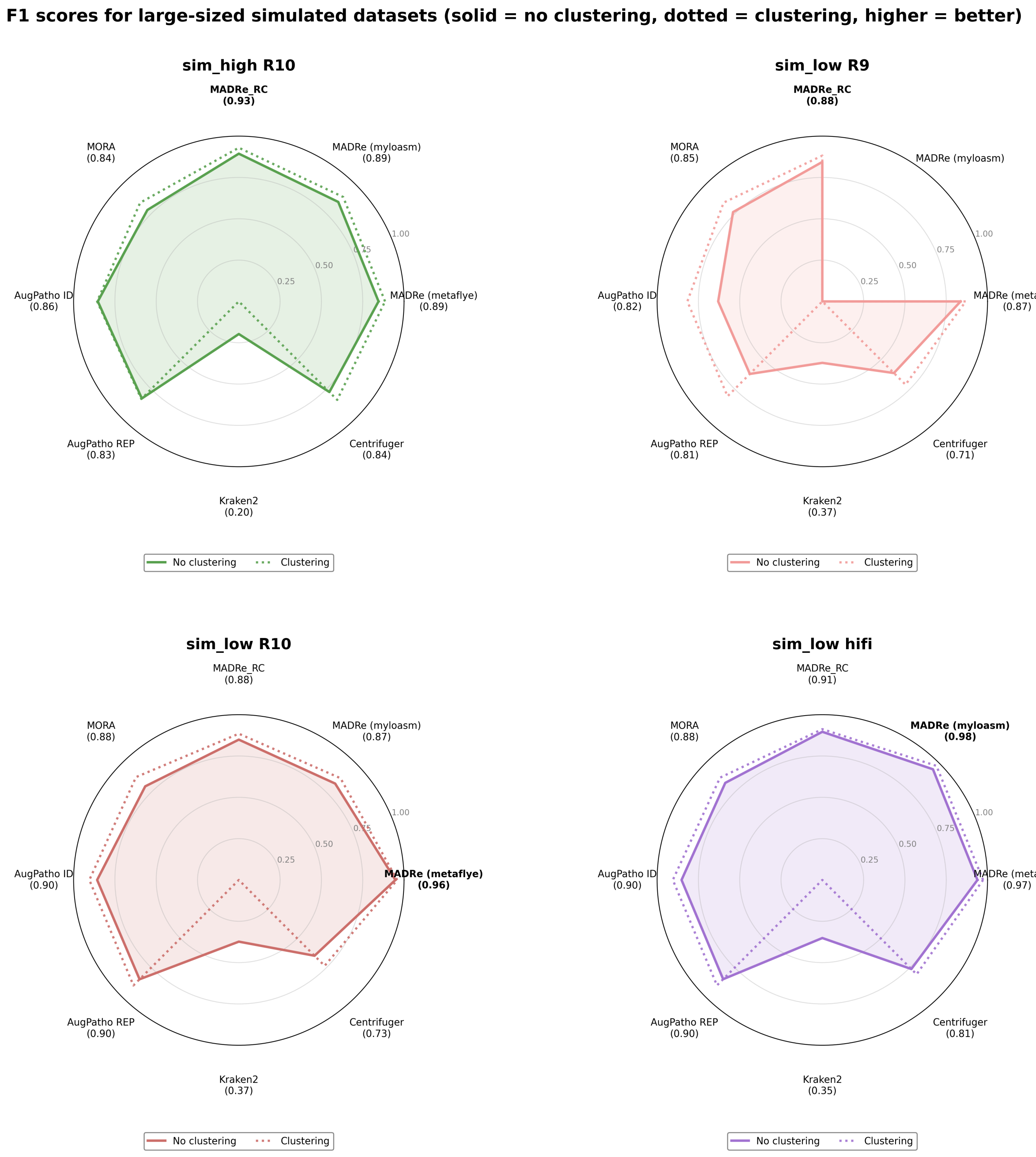

Figure 4. F1 scores for large-sized simulated datasets. Solid lines represent results without clustering, while dotted lines indicate results with clustering. Kraken2 clustering results are omitted, as its output format does not support clustering. Similarly, MADRe (Myloasm) results are excluded for ONT R9 data, since Myloasm is not designed for this type of sequencing data. In each plot, the best-performing tool is highlighted in bold, and the values in parentheses indicate the best performance achieved by each tool, with and without clustering.

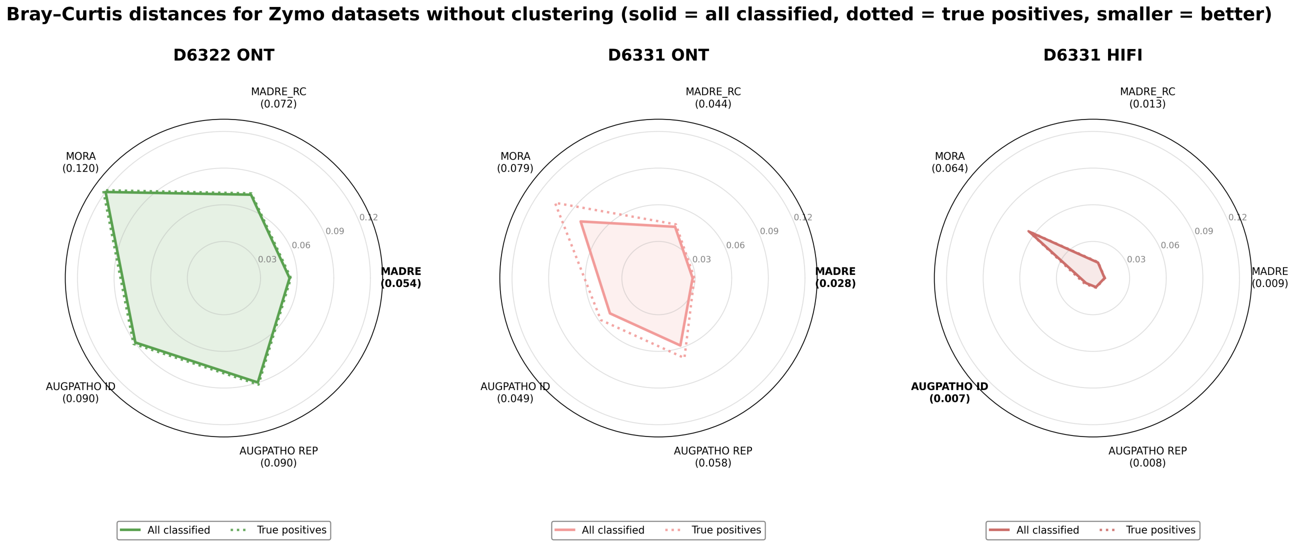

Figure 5. Bray–Curtis distances for Zymo datasets. The plots show changes in BC distance without post-clustering of similar strains. Solid lines represent distances based on all classified read counts, while dashed lines show distances calculated using only true positive (TP) read counts.

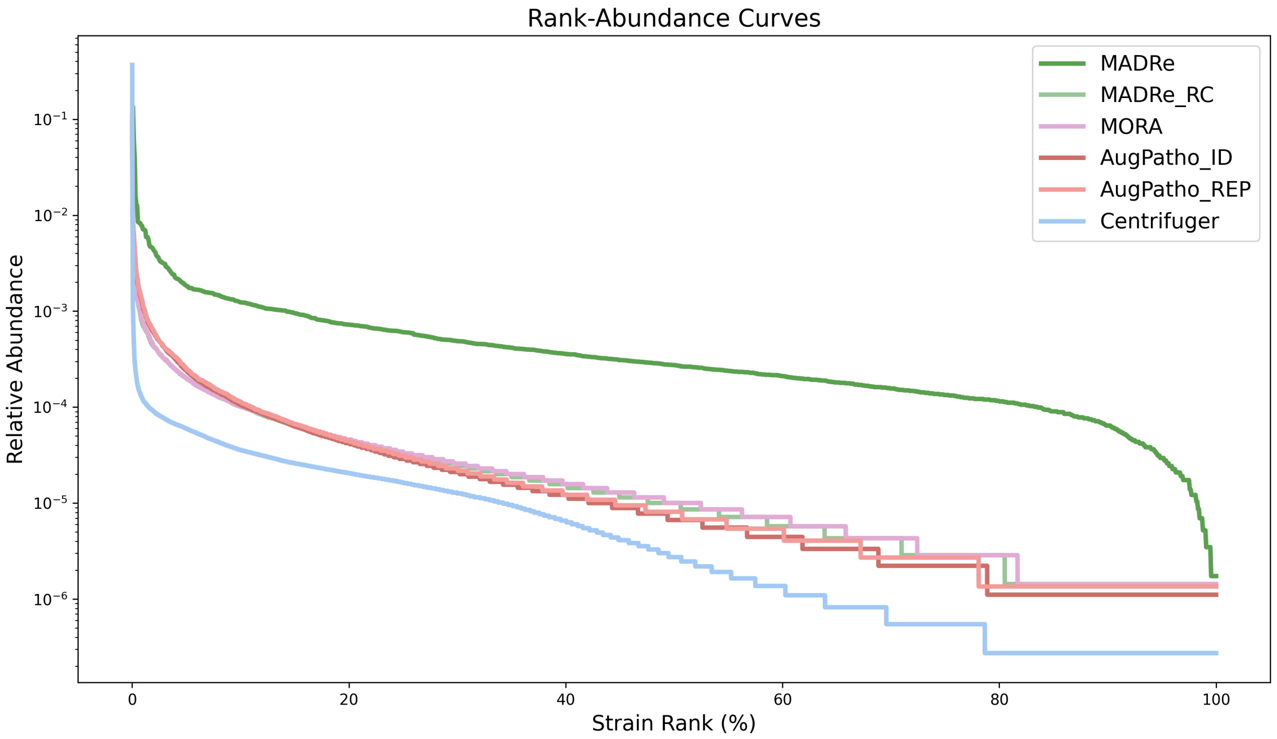

Figure 6. Percentile-normalized rank-abundance curves. The x-axis shows strain ranks expressed as percentiles, while the y-axis represents the relative abundance of each strain on a logarithmic scale.

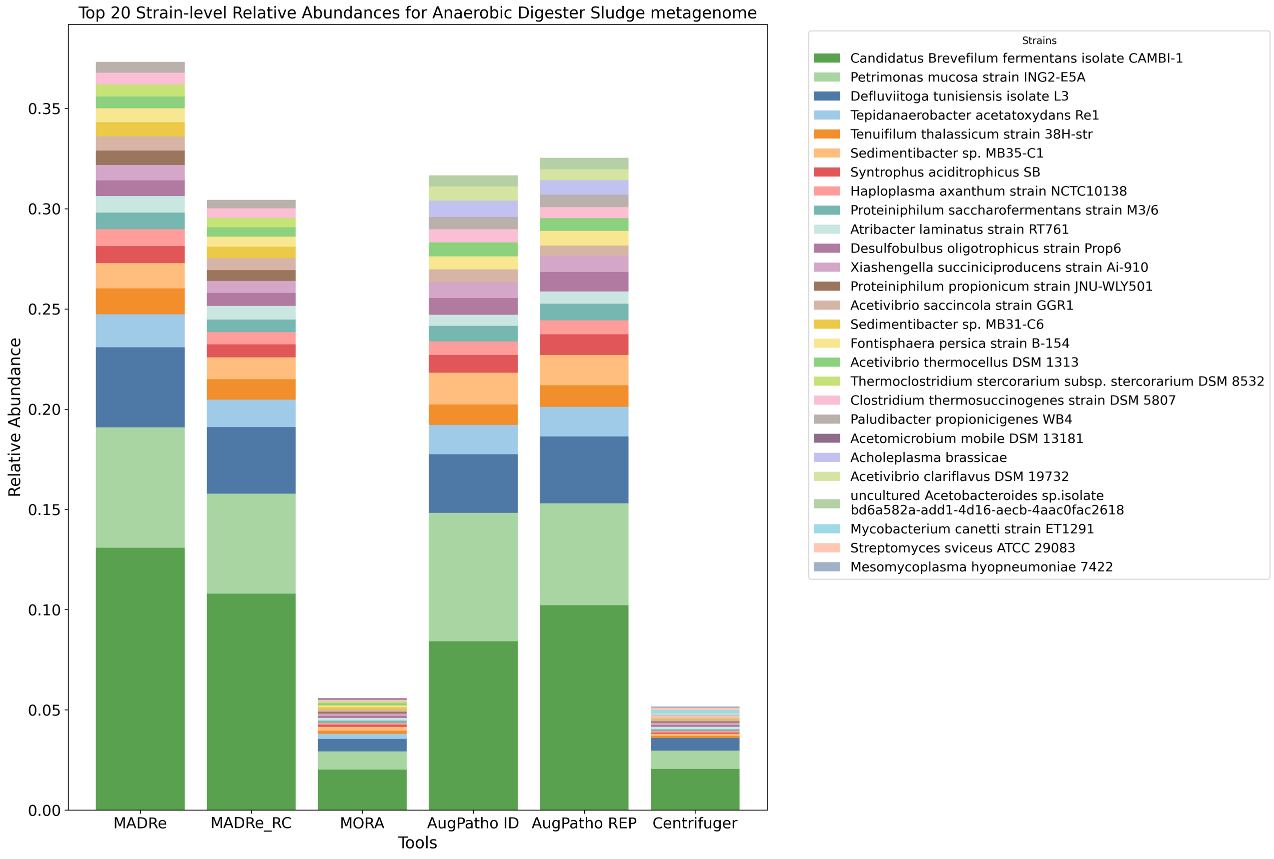

Figure 7. Real data strain-level abundances of the top 20 most abundant strains identified by each tool. Two strains are highlighted in red to illustrate cases where different tools classified reads originating from an unrepresented reference to distinct false positives that share similar genomic regions.

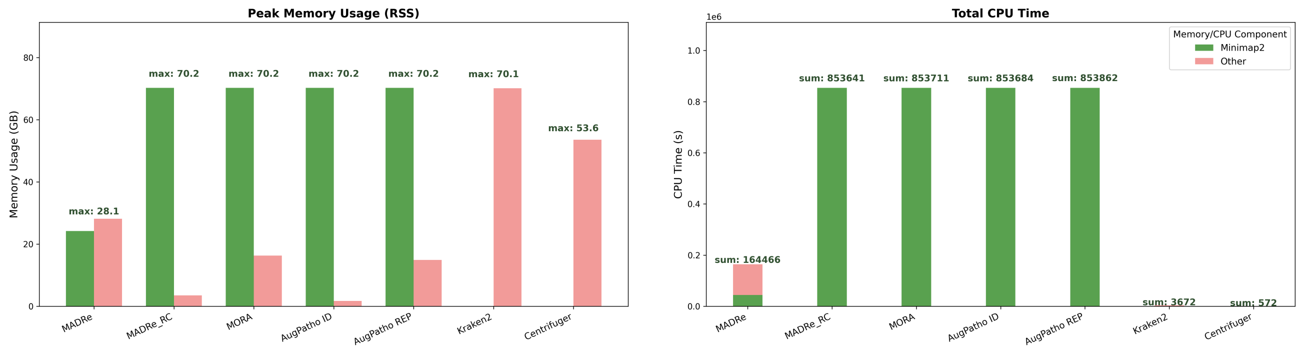

Figure 8. Memory (RSS peak in GB) and CPU time (in seconds) for different tools, split between Minimap2 mapping and other processing steps.

Supplementary File

Click here to access/download
Supplementary Material
MADRe_SupplementaryFile.pdf

Supplementary Tables

Click here to access/download
Supplementary Material
MADRe_SupplementaryTables.xlsx

Cover letter

Click here to access/download;Personal Cover;MADRe_CoverLetter.pdf

Editorial Office
GigaScience

2nd March 2026

Dear Editorial team,

We are pleased to resubmit our revised manuscript entitled **"MADRe: Assembly-Driven Database Reduction for Strain-Level Long-Read Metagenomic Classification"** for consideration in *GigaScience*.

We sincerely thank you and the reviewers for the constructive and insightful comments. We have carefully addressed all points raised and believe the manuscript has been substantially strengthened.

In this major revision, we have:

- Clarified strain-level evaluation for Centrifuger and Kraken2, including additional analysis and discussion of taxonomy-related limitations.
- Added new controlled synthetic experiments to better characterize performance across varying strain similarity and coverage levels.
- Expanded discussion of precision-recall trade-offs and clarified scenarios in which MADRe is most advantageous.
- Clarified limitations related to low-abundance strain detection and assembler sensitivity.
- Expanded discussion of computational trade-offs.
- Revised the Abstract and Discussion to better articulate MADRe's combined engineering and statistical contributions.
- Addressed all editorial comments, incorporated relevant recent *GigaScience* publications, and updated references to final published versions where applicable.

A detailed point-by-point response is provided in the accompanying document.

We appreciate your consideration and look forward to your feedback.

Sincerely,

Josipa Lipovac
Laboratory for Bioinformatics and Computational Biology, Faculty of Electrical Engineering and Computing, University of Zagreb, Zagreb, Croatia
josipa.lipovac@fer.unizg.hr
(on behalf of all co-authors)

Responses to reviewers' comments
Click here to access/download;Personal Cover;MADRe_ResponsesToReviewers.pdf

We thank the reviewers for their constructive and valuable feedback on our manuscript.
Below, we provide *detailed responses* to each of the raised concerns.
Manuscript changes corresponding to the comments are indicated by line numbers.
All modifications in the manuscript are highlighted in yellow

---

1) "However, we observed a limitation when Centrifuger cannot confidently assign a read to a specific reference sequence (for example, when multiple chromosomes belong to the same strain). In such cases, it often classifies the read under the NCBI strain-level taxid, which in some instances is identical to the species-level taxid. This makes it impossible to directly and fairly compare those classifications with other tools that operate at the sequence level."

Although I agree this issue may not substantially affect the overall conclusions, the current handling of strain-level evaluation for Centrifuger is not sufficiently rigorous. The underlying problem is that Centrifuger (and Kraken2) rely on nodes.dmp and names.dmp, where the lowest taxonomic rank is often species or subspecies. As a result, these tools cannot report strain-level abundances directly in their standard output.
A more appropriate solution would be to assign custom, unique strain-level taxIDs for all reference genomes, allowing proper classification at the strain level. This approach has been discussed in https://github.com/mourisl/centrifuger/issues/18 and https://github.com/jenniferlu717/Bracken/issues/113. Additionally, Centrifuger has an extra program, centrifuger-quant, that uses the EM algorithm to estimate abundance. The read assignment results produced by Centrifuger do not apply the EM algorithm.

→ answer:
We thank the reviewer for raising this important point regarding strain-level evaluation for Centrifuger (and similarly Kraken2). We agree that tools relying on the NCBI taxonomy hierarchy can face challenges when performing strain-level classification, particularly when strain-level taxonomic identifiers are unavailable or identical to species-level taxids.

In our study, we used reference databases that include strain-level taxonomy whenever such information is available. However, as noted by the reviewer and discussed previously in the literature, many recently submitted genome assemblies do not have unique strain-level taxids and are instead assigned only species- or subspecies-level identifiers (see, e.g., NCBI taxonomy limitations described in [1]).

To enable a rigorous and fair strain-level evaluation wherever possible, we designed our medium-sized simulated datasets using organisms that do possess unique strain-level taxids. This allowed us to directly evaluate strain-level classification performance for Kraken2 and Centrifuger under conditions where such evaluation is well-defined. For more complex datasets, where unique strain-level taxids are not available, we explicitly state in the manuscript (lines 1074-1085) that this ambiguity affects only a small fraction of reads. Importantly, even taking this into account, MADRe consistently demonstrates improved behavior compared to other methods.

The reviewer suggests assigning custom strain-level taxids for all reference genomes. While this is a valid approach and has been discussed in the community (e.g., Centrifuger and Bracken issue trackers), it would require modifying the standard NCBI taxonomy and rebuilding databases in a non-standard manner. Our goal in this work was to benchmark MADRe under realistic and widely used settings that reflect how these tools are typically applied in practice, rather than introducing custom taxonomic schemes that could limit reproducibility or comparability.

We additionally evaluated the impact of abundance re-estimation using the centrifuger-quant module on the medium-sized simulated datasets (Supplementary tables ST5-7). We observed only minimal changes in estimated read counts, generally in a favorable direction. However, centrifuger-quant reports abundance estimates rather than read-level classifications, which is central to MADRe's objective (lines: 666-673).

2) In the similarity experiment, some strains exhibit extremely high similarity, which makes proportional read distribution practically impossible for MADRe. To better characterize the performance limits of MADRe for accurate strain classification and abundance estimation, I recommend including additional simple synthetic mixtures at different combinations of similarity and coverage depth.

→ answer:
We agree that extremely high strain similarity represents a challenging scenario for any strain-level classification method. The high-similarity experiment included in the manuscript was intentionally designed as a stress test to probe the behavior of MADRe under such rigorous conditions. This experiment was added in response to a previous reviewer request, with the explicit goal of illustrating how MADRe behaves when strains are nearly indistinguishable at the sequence level. As anticipated, MADRe collapsed reads toward the nearest highly similar reference, and the results confirmed this behavior. Importantly, alternative approaches exhibited comparable limitations in this setting, although they resolved ambiguity in different ways (lines: 783-791; Supplementary File: Similar Strains Experiment; Supplementary Table ST23).

Rather than aiming to demonstrate optimal performance under extreme similarity, the purpose of this experiment was to provide transparency and interpretability - to clarify what MADRe's outputs represent when strain similarity exceeds the resolution supported by the data. This information is valuable for downstream analyses, where abundance estimates must be interpreted in the presence of nearly identical strains.

To further characterize the performance limits of MADRe across combinations of strain similarity and coverage depth, and to directly address the final part of the reviewer's question, we conducted additional controlled synthetic experiments using reads simulated by Badread. We simulated four sets of mixtures, each containing three strains of varying similarity from E. coli, P. aeruginosa, S. aureus, and L. monocytogenes. These species were chosen following the Strainy study design [2] and because they are extensively represented in reference databases with many closely related strains, providing a realistic and challenging setting for strain-level discrimination. In each dataset, two additional strains from the sim_small dataset (taxid

446660) were included to introduce some inter-species context. For each species, the coverage of one strain was varied (20x, 10x, 5x, and 3x) to evaluate performance across coverage levels. The full dataset composition, ANI scores, and classification results are provided in Supplementary materials (Supplementary File: Coverage and Similarity Experiment; Supplementary Table ST24).

These experiments demonstrate that coverage depth does not substantially affect the detection of the correct strain, rather, performance is primarily influenced by the presence of multiple highly similar strains in the reference database. When expected strains were not recovered, this was primarily associated with extremely high similarity (>99.9% ANI) to another genome in the database, which prevented reliable discrimination. In such scenarios, MADRe consistently assigned reads to one of the highly similar strains, reflecting intrinsic resolution limits imposed by near-identical genomes rather than instability of the method. Together, these results confirm that MADRe remains robust across varying similarity and coverage conditions.

All other datasets and experiments in the study, including medium and large simulated datasets, Zymo mock communities, real metagenomic samples, and the additional synthetic mixtures described above, reflect realistic biological scenarios and demonstrate the typical performance of MADRe in practical applications. In those datasets, strains that were not recovered were often low-abundance, and in most of the cases these strains also exhibited very high similarity to another, typically more abundant reference genome. This indicates that the principal limiting factor is sequence similarity rather than coverage alone. Additionally, in all these experiments, MADRe was systematically benchmarked against state-of-the-art tools, providing a consistent benchmark across varying similarity and coverage conditions. In some complex communities, the detection of certain lower-abundance strains was affected. However, this mainly results from the presence of highly similar strains in both the dataset and the reference database. Despite this, MADRe consistently achieves competitive performance with a favorable balance between precision and recall.

We have revised the Discussion to explicitly address these conclusions (lines: 773-791).

3) Because long reads vary widely in length, read counts alone can be misleading. I strongly encourage reporting strain abundances rather than raw read counts, as abundances are more relevant for downstream applications.

→ answer:

We strongly agree with the reviewer that strain abundance estimates are generally more informative than raw read counts for downstream biological analyses, particularly when read lengths vary widely, as is common in long-read sequencing data.

For this reason, MADRe explicitly reports strain-level abundance estimates as one of its standard outputs. The abundance calculation procedure is described in detail in Section 5.2.1 (Abundance calculation).

In the experimental evaluation, we report classification results primarily in terms of read counts. This choice was made for clarity and comparability: all methods were evaluated on

identical datasets containing the same set of reads, and under these conditions, read-count-based metrics and abundance-based metrics lead to consistent conclusions. Using read counts allows a more direct interpretation of classification correctness and simplifies comparison across tools without introducing additional normalization layers.

4) Finally, the authors should clarify whether MADRe's limitations in detecting low-abundance strains (referring more to low coverage) is entirely determined by the performance of the assembly tool, or whether additional factors influence this limitation.

→ answer:
We thank the reviewer for raising this point and agree that this should be discussed more. MADRe's sensitivity to low-abundance strains is influenced by two main factors.
First, detection of low-abundance strains is fundamentally limited by the performance of the metagenome assembly step. If a strain is present at very low coverage, it may fail to assemble or may produce contigs that are too short, fragmented, or chimeric, containing insufficient strain-specific signal to support confident detection of the low-abundance strain. However, our controlled similarity-coverage experiments indicate that coverage alone is not the primary limiting factor within the evaluated range. Instead, challenges arise primarily when low-abundance strains coexist with highly similar and more abundant strains. In such cases, assembly may lead to partial signal collapse or insufficiently distinct contigs for confident separation during the database reduction step.In such cases, the strain cannot be detected by MADRe. Importantly, the use of assembly provides substantial benefits in reducing false positives and reducing the database, and similar low-coverage limitations exist for other strain-level methods that depend on sufficient genomic context.

Second, even when assembly enables detection of a low-abundance strain, additional challenges can arise during the read reassignment step when a highly similar (and high-abundance) strain is present. In such cases, if there are insufficient reads uniquely supporting the low-abundance strain, those reads may be reassigned to the dominant strain.

We have extended the Discussion to explicitly distinguish these two factors (lines: 749-791).

5) In Figure 4, please specify the sequencing technology used for sim_high. → ONT
"calculated using fastANI" →"calculated using fastANI".

-> answer:
The sequencing technology for sim_high (ONT) has now been specified in Figure 4, and the phrasing has been corrected to "calculated using fastANI."

Reviewer #2: This manuscript presents MADRe, a modular pipeline for strain-level metagenomic classification from long-read data, emphasizing an assembly-driven database reduction strategy coupled with probabilistic reassignment. The work is methodologically sound and well aligned with the scope of GigaScience. However, the study can be benefit from the following revisions:

1, the study's main contribution is engineering and integration, rather than a fundamentally new statistical model. The authors thus should explicitly mention this in the Abstract as well as the Discussion part.

→ answer:

We thank the reviewer for this comment. We agree that a substantial contribution of MADRe lies in the careful engineering and integration of multiple components into a modular and scalable pipeline, which is essential for achieving robust strain-level classification in practice.

However, we would like to clarify that MADRe is not purely an engineering contribution. In particular, the database reduction strategy based on contig-to-reference reassignment using an expectation-maximization framework, as well as the subsequent probabilistic read reassignment using mapping profiles and operating on a reduced reference set, introduce methodological innovations that go beyond straightforward tool integration. These components define a new statistical formulation.

To address the reviewer's suggestion, we now explicitly state in the Abstract and Discussion (lines: 614-618) that MADRe's contribution combines system-level engineering with novel statistical modeling choices, while emphasizing how these methodological components enable improved strain-level resolution.

2, although comparisons are reasonable, the manuscript could do more to clarify how MADRe compares against state-of-the-art strain-resolved tools under identical parameter tuning, and whether performance gains are consistent across different strain divergence levels.

→ answer:
We thank the reviewer for this comment and agree that clarity regarding comparison settings is important.

All tools included in the benchmarking were executed using default or recommended parameters, without dataset-specific tuning, as described in the Methods section. This ensures a fair and consistent comparison across methods.

We note that there exist strain-resolved tools that assume prior knowledge of species identity and operate within a single-species context. As explicitly stated in the manuscript, such methods were not included in the comparison because MADRe is designed for realistic metagenomic scenarios where species composition is unknown and multiple species may be present simultaneously. Including single-species-restricted methods would therefore not provide a fair or meaningful comparison for the problem setting addressed in this study.

The evaluated comparative datasets used in this study span a broad range of strain divergence levels, including medium and large simulated datasets with moderate divergence, a dedicated high-similarity stress test, Zymo mock communities, and real metagenomic samples. Across these settings, MADRe demonstrates consistent performance trends relative to state-of-the-art tools.

To further characterize the performance limits of MADRe across combinations of strain similarity and coverage depth, we conducted additional controlled synthetic experiments (Supplementary File: Coverage and Similarity Experiment; Supplementary Table ST24), as noted in one of the previous responses. In these datasets, we simulated mixtures of three strains with varying ANI levels from E. coli, P. aeruginosa, S. aureus, and L. monocytogenes, and systematically varied the coverage of one strain (20x, 10x, 5x, and 3x). These experiments were designed to probe the sensitivity of MADRe under controlled divergence and abundance conditions. As stated in the answer to the previous reviewer, we demonstrated that coverage depth does not substantially affect the detection of the correct strain, rather, performance is primarily influenced by the presence of multiple highly similar strains in the reference database. When expected strains were not recovered, this was primarily associated with extremely high similarity (>99.9% ANI) to another genome in the database, which prevented reliable discrimination.

We have revised the Discussion to explicitly address these conclusions (lines: 773-791).

We trust that this expanded analysis addresses the reviewer's comment. If further clarification of the requested scenarios is needed, we would be grateful for additional guidance.

3, when comparing with existing tools, improvements appear primarily in precision, while recall trade-offs are less emphasized. The authors should explicitly discuss precision-recall trade-offs and clarify in which biological scenarios MADRe is most advantageous.

→ answer:
We agree with the reviewer that precision-recall trade-offs should be discussed more explicitly. In this study, we intentionally emphasize precision because MADRe is designed to reduce false-positive detections, which are a major challenge in strain-level metagenomic classification, particularly when using large and redundant reference databases.

At the genome identification level, a strain is considered detected if at least one read is classified to it. Under this definition, MADRe exhibits lower recall but higher precision compared to other approaches (e.g., results in ST20). This behavior reflects MADRe's identification strategy, which reduces false-positive strain detections at the cost of missing some low-support strains or sequences.

However, when read-level classification is considered, the picture is different. Although some strains may be identified by other methods but not by MADRe, the number of reads originating from such strains is typically low. In addition, when a strain is not retained during database reduction, its reads are often reassigned to the most similar reference in the reduced database. In such cases, read-level recall may remain largely unaffected, while strain-level precision can be influenced by the degree of similarity among reference genomes. For this reason, we placed greater emphasis on the precision metric. Furthermore, the number of false-negative reads is comparable between MADRe and other approaches, despite differences observed at the genome identification level.

In many evaluated datasets, the fraction of unclassified reads is low relative to the total number of reads, making genome-level recall less informative as a primary metric. Nevertheless, we

report recall, precision, and F1 scores for all experiments, with detailed results provided in the Supplementary Tables (ST8 and ST15). Notably, for large simulated datasets, MADRe achieves comparable or improved recall while simultaneously improving precision, which is reflected in competitive or higher F1 scores.

We have revised the Discussion to explicitly clarify this precision-recall trade-off and to highlight that MADRe is particularly advantageous in applications where precision is prioritized, such as settings in which false-positive strain detection carries greater consequences than missing extremely low-abundance organisms (lines: 760-772).

4, While database reduction is presented as efficient, the computational cost of assembly plus EM iterations is not deeply analyzed. The authors should include a concise runtime/memory comparison or at least a qualitative discussion of computational trade-offs.

→ answer:
We thank the reviewer for this comment. A detailed analysis of runtime and memory usage is already provided in the Supplementary Table (ST22), which reports CPU time and peak memory consumption for all major steps of the MADRe pipeline across two representative datasets, including database reduction.

The database reduction stage comprises: (i) metagenome assembly, (ii) mapping assembled contigs to the full reference database, (iii) the HairSplitter step used to detect collapsed strain signal, and (iv) the reference reduction algorithm itself. Among these, the assembly step is the most computationally demanding, particularly for large datasets. In contrast, contig-to-database mapping and the HairSplitter-based processing are substantially less resource-intensive than mapping all reads to a large reference database, and the reference reduction algorithm itself has minimal computational overhead.

We have clarified this computational trade-off in the revised manuscript (lines:645-655).

5, The approach implicitly assumes that metagenome assembly is sufficiently accurate and representative. However, in highly complex or low-coverage samples, assembly could be fragmented or biased. The authors should add a clearer discussion on the sensitivity to assembler choice and parameters.

→ answer:
We thank the reviewer for this comment and agree that metagenome assembly quality can influence strain detection, particularly in highly complex or low-coverage samples.

MADRe is designed as a modular pipeline, allowing different assemblers and parameter settings to be used without modifying the core workflow. To assess sensitivity to assembler choice, we evaluated MADRe on large simulated datasets using Myloasm instead of metaFlye and metaMDBG. Overall performance trends were comparable, indicating that MADRe is not strongly dependent on a specific assembler. We observed complementary behavior, with Myloasm performing better for low-abundance strains and metaFlye performing slightly better

for highly abundant strains. Based on these results, Myloasm is included as an optional component within the MADRe pipeline.

We note that strain-level assemblers were not considered, as they typically produce shorter and more fragmented contigs. MADRe's database reduction algorithm benefits from longer genomic context.

Regarding parameter sensitivity, all assemblers were executed using default or recommended settings. While different parameter choices may influence assembly quality, exploring extensive parameter tuning would substantially expand the scope of this study. We clarify this in the revised Discussion (lines:812-827).

REFERENCES:

[1] Federhen, Scott, et al. "Toward richer metadata for microbial sequences: replacing strain-level NCBI taxonomy taxids with BioProject, BioSample and Assembly records." Standards in genomic sciences 9.3 (2014): 1275-1277.
[2] Kazantseva, Ekaterina, et al. "Strainy: phasing and assembly of strain haplotypes from long-read metagenome sequencing." Nature Methods 21.11 (2024): 2034-2043.

Editor Comments:

GigaScience has also published a number of relevant papers on strain-level metagenomic classification, machine learning-based host prediction, and pipeline development for microbiome analysis. Citing these papers would help contextualize your methodological contributions and strengthen the discussion of your results within the current literature landscape.

1. Yang Y, Dufault-Thompson K, Yan W, Cai T, Xie L, Jiang X. Large-scale genomic survey with deep learning-based method reveals strain-level phage specificity determinants. GigaScience. 2024;13:giae017. https://doi.org/10.1093/gigascience/giae017

2. Chen G, Jiang J, Sun Y. RNAVirHost: a machine learning–based method for predicting hosts of RNA viruses through viral genomes. GigaScience. 2024;13:giae059. https://doi.org/10.1093/gigascience/giae059

3. Arikan M, Muth T. gNOMO2: a comprehensive and modular pipeline for integrated multi-omics analyses of microbiomes. GigaScience. 2024;13:giae038. https://doi.org/10.1093/gigascience/giae038

4. Roach MJ, Beecroft SJ, Mihindukulasuriya KA, et al. Hecatomb: an integrated software platform for viral metagenomics. GigaScience. 2024;13:giae020. https://doi.org/10.1093/gigascience/giae020

5. Gao Y, Luo H, Lyu H, et al. Benchmarking short-read metagenomics tools for removing host contamination. GigaScience. 2025;14:giaf004.
https://doi.org/10.1093/gigascience/giaf004

-> answer:
We thank the Editor for this helpful suggestion and for highlighting relevant recent publications in GigaScience. We agree that situating MADRe within the broader landscape of strain-level metagenomic and microbiome analysis methods strengthens the manuscript.

Accordingly, we have incorporated citations to Yang et al. (2024), Arikan and Muth (2024), and Gao et al. (2025), as these studies are directly relevant to strain-level genomic analysis, machine learning approaches applied to microbial genomics, and modular/benchmarking frameworks in metagenomic workflows.

We also carefully considered the studies by Chen et al. (2024) and Roach et al. (2024). However, these works focus primarily on RNA virus host prediction and viral metagenomics platforms, respectively. We believe that these studies fall outside the direct methodological scope of our work. For this reason, we did not incorporate them into the discussion to avoid overstretching the conceptual framing.

We appreciate the Editor's guidance in ensuring comprehensive contextualization within the journal's recent literature.

In addition, please ensure that all references are to the final, peer-reviewed published versions of articles. If you have cited any preprints (e.g., from bioRxiv) that have now been published in journals, please update those entries accordingly. This is an important step for the long-term archival stability and credibility of the reference list.

We have reviewed the entire reference list and updated all entries to cite the final peer-reviewed published versions where available, replacing preprint citations accordingly.
