# Supplementary material for: MADRe: Strain-level metagenomic classification through assembly-driven database reduction
Source: Gigascience. 2026 Mar 23;15:giag030. doi: 10.1093/gigascience/giag030 (PMC13211987; doi:10.1093/gigascience/giag030)
Supplement: giag030_Authors_Response_To_Reviewer_Comments_original_submission [file giag030_authors_response_to_reviewer_comments_original_submission.pdf]

Author's Response To Reviewer Comments

For convenience, we have also attached a separate document containing cover letter and responses to facilitate the review process.

Editorial Office
GigaScience
2nd March 2026

Dear Editorial team,
We are pleased to resubmit our revised manuscript entitled “MADRe: Assembly-Driven Database Reduction for Strain-Level Long-Read Metagenomic Classification” for consideration in GigaScience.
We sincerely thank you and the reviewers for the constructive and insightful comments. We have carefully addressed all points raised and believe the manuscript has been substantially strengthened.
In this major revision, we have:
- Clarified strain-level evaluation for Centrifuger and Kraken2, including additional analysis and discussion of taxonomy-related limitations.
- Added new controlled synthetic experiments to better characterize performance across varying strain similarity and coverage levels.
- Expanded discussion of precision-recall trade-offs and clarified scenarios in which MADRe is most advantageous.
- Clarified limitations related to low-abundance strain detection and assembler sensitivity.
- Expanded discussion of computational trade-offs.
- Revised the Abstract and Discussion to better articulate MADRe’s combined engineering and statistical contributions.
- Addressed all editorial comments, incorporated relevant recent GigaScience publications, and updated references to final published versions where applicable.

A detailed point-by-point response is provided in the accompanying document.
We appreciate your consideration and look forward to your feedback.

Sincerely,
Josipa Lipovac
Laboratory for Bioinformatics and Computational Biology, Faculty of Electrical Engineering and Computing, University of Zagreb, Zagreb, Croatia
josipa.lipovac@fer.unizg.hr
(on behalf of all co-authors)

We thank the reviewers for their constructive and valuable feedback on our manuscript. Below, we provide detailed responses to each of the raised concerns. Manuscript changes corresponding to the comments are indicated by line numbers. All modifications in the manuscript are highlighted in yellow.

----

1) "However, we observed a limitation when Centrifuger cannot confidently assign a read to a specific reference sequence (for example, when multiple chromosomes belong to the same strain). In such cases, it often classifies the read under the NCBI strain-level taxid, which in some instances is identical to the species-level taxid. This makes it impossible to directly and fairly compare those classifications with other tools that operate at the sequence level."

Although I agree this issue may not substantially affect the overall conclusions, the current handling of strain-level evaluation for Centrifuger is not sufficiently rigorous. The underlying problem is that Centrifuger (and Kraken2) rely on nodes.dmp and names.dmp, where the lowest taxonomic rank is often

species or subspecies. As a result, these tools cannot report strain-level abundances directly in their standard output.
A more appropriate solution would be to assign custom, unique strain-level taxIDs for all reference genomes, allowing proper classification at the strain level. This approach has been discussed in https://github.com/mourisl/centrifuger/issues/18 and https://github.com/jenniferlu717/Bracken/issues/113. Additionally, Centrifuger has an extra program, centrifuger-quant, that uses the EM algorithm to estimate abundance. The read assignment results produced by Centrifuger do not apply the EM algorithm.

→ answer:
We thank the reviewer for raising this important point regarding strain-level evaluation for Centrifuger (and similarly Kraken2). We agree that tools relying on the NCBI taxonomy hierarchy can face challenges when performing strain-level classification, particularly when strain-level taxonomic identifiers are unavailable or identical to species-level taxids.

In our study, we used reference databases that include strain-level taxonomy whenever such information is available. However, as noted by the reviewer and discussed previously in the literature, many recently submitted genome assemblies do not have unique strain-level taxids and are instead assigned only species- or subspecies-level identifiers (see, e.g., NCBI taxonomy limitations described in [1]).

To enable a rigorous and fair strain-level evaluation wherever possible, we designed our medium-sized simulated datasets using organisms that do possess unique strain-level taxids. This allowed us to directly evaluate strain-level classification performance for Kraken2 and Centrifuger under conditions where such evaluation is well-defined. For more complex datasets, where unique strain-level taxids are not available, we explicitly state in the manuscript (lines 1074-1085) that this ambiguity affects only a small fraction of reads. Importantly, even taking this into account, MADRe consistently demonstrates improved behavior compared to other methods.

The reviewer suggests assigning custom strain-level taxids for all reference genomes. While this is a valid approach and has been discussed in the community (e.g., Centrifuger and Bracken issue trackers), it would require modifying the standard NCBI taxonomy and rebuilding databases in a non-standard manner. Our goal in this work was to benchmark MADRe under realistic and widely used settings that reflect how these tools are typically applied in practice, rather than introducing custom taxonomic schemes that could limit reproducibility or comparability.

We additionally evaluated the impact of abundance re-estimation using the centrifuger-quant module on the medium-sized simulated datasets (Supplementary tables ST5-7). We observed only minimal changes in estimated read counts, generally in a favorable direction. However, centrifuger-quant reports abundance estimates rather than read-level classifications, which is central to MADRe's objective (lines: 666-673).

2) In the similarity experiment, some strains exhibit extremely high similarity, which makes proportional read distribution practically impossible for MADRe. To better characterize the performance limits of MADRe for accurate strain classification and abundance estimation, I recommend including additional simple synthetic mixtures at different combinations of similarity and coverage depth.

→ answer:
We agree that extremely high strain similarity represents a challenging scenario for any strain-level classification method. The high-similarity experiment included in the manuscript was intentionally designed as a stress test to probe the behavior of MADRe under such rigorous conditions. This experiment was added in response to a previous reviewer request, with the explicit goal of illustrating how MADRe behaves when strains are nearly indistinguishable at the sequence level. As anticipated, MADRe collapsed reads toward the nearest highly similar reference, and the results confirmed this behavior. Importantly, alternative approaches exhibited comparable limitations in this setting, although they resolved ambiguity in different ways (lines: 783-791; Supplementary File: Similar Strains Experiment; Supplementary Table ST23).
Rather than aiming to demonstrate optimal performance under extreme similarity, the purpose of this experiment was to provide transparency and interpretability - to clarify what MADRe's outputs represent

when strain similarity exceeds the resolution supported by the data. This information is valuable for downstream analyses, where abundance estimates must be interpreted in the presence of nearly identical strains.

To further characterize the performance limits of MADRe across combinations of strain similarity and coverage depth, and to directly address the final part of the reviewer's question, we conducted additional controlled synthetic experiments using reads simulated by Badread. We simulated four sets of mixtures, each containing three strains of varying similarity from E. coli, P. aeruginosa, S. aureus, and L. monocytogenes. These species were chosen following the Strainy study design [2] and because they are extensively represented in reference databases with many closely related strains, providing a realistic and challenging setting for strain-level discrimination. In each dataset, two additional strains from the sim_small dataset (taxid 446660) were included to introduce some inter-species context. For each species, the coverage of one strain was varied (20x, 10x, 5x, and 3x) to evaluate performance across coverage levels. The full dataset composition, ANI scores, and classification results are provided in Supplementary materials (Supplementary File: Coverage and Similarity Experiment; Supplementary Table ST24).

These experiments demonstrate that coverage depth does not substantially affect the detection of the correct strain, rather, performance is primarily influenced by the presence of multiple highly similar strains in the reference database. When expected strains were not recovered, this was primarily associated with extremely high similarity (>99.9% ANI) to another genome in the database, which prevented reliable discrimination. In such scenarios, MADRe consistently assigned reads to one of the highly similar strains, reflecting intrinsic resolution limits imposed by near-identical genomes rather than instability of the method. Together, these results confirm that MADRe remains robust across varying similarity and coverage conditions.

All other datasets and experiments in the study, including medium and large simulated datasets, Zymo mock communities, real metagenomic samples, and the additional synthetic mixtures described above, reflect realistic biological scenarios and demonstrate the typical performance of MADRe in practical applications. In those datasets, strains that were not recovered were often low-abundance, and in most of the cases these strains also exhibited very high similarity to another, typically more abundant reference genome. This indicates that the principal limiting factor is sequence similarity rather than coverage alone. Additionally, in all these experiments, MADRe was systematically benchmarked against state-of-the-art tools, providing a consistent benchmark across varying similarity and coverage conditions. In some complex communities, the detection of certain lower-abundance strains was affected. However, this mainly results from the presence of highly similar strains in both the dataset and the reference database. Despite this, MADRe consistently achieves competitive performance with a favorable balance between precision and recall.

We have revised the Discussion to explicitly address these conclusions (lines: 773-791).

3) Because long reads vary widely in length, read counts alone can be misleading. I strongly encourage reporting strain abundances rather than raw read counts, as abundances are more relevant for downstream applications.

→ answer:

We strongly agree with the reviewer that strain abundance estimates are generally more informative than raw read counts for downstream biological analyses, particularly when read lengths vary widely, as is common in long-read sequencing data.

For this reason, MADRe explicitly reports strain-level abundance estimates as one of its standard outputs. The abundance calculation procedure is described in detail in Section 5.2.1 (Abundance calculation).

In the experimental evaluation, we report classification results primarily in terms of read counts. This choice was made for clarity and comparability: all methods were evaluated on identical datasets containing the same set of reads, and under these conditions, read-count-based metrics and abundance-based metrics lead to consistent conclusions. Using read counts allows a more direct interpretation of classification correctness and simplifies comparison across tools without introducing additional normalization layers.

4) Finally, the authors should clarify whether MADRe's limitations in detecting low-abundance strains (referring more to low coverage) is entirely determined by the performance of the assembly tool, or whether additional factors influence this limitation.

→ answer:
We thank the reviewer for raising this point and agree that this should be discussed more. MADRe's sensitivity to low-abundance strains is influenced by two main factors.
First, detection of low-abundance strains is fundamentally limited by the performance of the metagenome assembly step. If a strain is present at very low coverage, it may fail to assemble or may produce contigs that are too short, fragmented, or chimeric, containing insufficient strain-specific signal to support confident detection of the low-abundance strain. However, our controlled similarity-coverage experiments indicate that coverage alone is not the primary limiting factor within the evaluated range. Instead, challenges arise primarily when low-abundance strains coexist with highly similar and more abundant strains. In such cases, assembly may lead to partial signal collapse or insufficiently distinct contigs for confident separation during the database reduction step.In such cases, the strain cannot be detected by MADRe. Importantly, the use of assembly provides substantial benefits in reducing false positives and reducing the database, and similar low-coverage limitations exist for other strain-level methods that depend on sufficient genomic context.

Second, even when assembly enables detection of a low-abundance strain, additional challenges can arise during the read reassignment step when a highly similar (and high-abundance) strain is present. In such cases, if there are insufficient reads uniquely supporting the low-abundance strain, those reads may be reassigned to the dominant strain.

We have extended the Discussion to explicitly distinguish these two factors (lines: 749-791).

5) In Figure 4, please specify the sequencing technology used for sim_high. → ONT
"calculated using fastANI" →"calculated using fastANI".

-> answer:
The sequencing technology for sim_high (ONT) has now been specified in Figure 4, and the phrasing has been corrected to "calculated using fastANI."

Reviewer #2: This manuscript presents MADRe, a modular pipeline for strain-level metagenomic classification from long-read data, emphasizing an assembly-driven database reduction strategy coupled with probabilistic reassignment. The work is methodologically sound and well aligned with the scope of GigaScience. However, the study can be benefit from the following revisions:

1) the study's main contribution is engineering and integration, rather than a fundamentally new statistical model. The authors thus should explicitly mention this in the Abstract as well as the Discussion part.

→ answer:
We thank the reviewer for this comment. We agree that a substantial contribution of MADRe lies in the careful engineering and integration of multiple components into a modular and scalable pipeline, which is essential for achieving robust strain-level classification in practice.
However, we would like to clarify that MADRe is not purely an engineering contribution. In particular, the database reduction strategy based on contig-to-reference reassignment using an expectation-maximization framework, as well as the subsequent probabilistic read reassignment using mapping profiles and operating on a reduced reference set, introduce methodological innovations that go beyond straightforward tool integration. These components define a new statistical formulation.
To address the reviewer's suggestion, we now explicitly state in the Abstract and Discussion (lines: 614-618) that MADRe's contribution combines system-level engineering with novel statistical modeling choices, while emphasizing how these methodological components enable improved strain-level resolution.

2) although comparisons are reasonable, the manuscript could do more to clarify how MADRe compares against state-of-the-art strain-resolved tools under identical parameter tuning, and whether performance gains are consistent across different strain divergence levels.

→ answer:
We thank the reviewer for this comment and agree that clarity regarding comparison settings is important.

All tools included in the benchmarking were executed using default or recommended parameters, without dataset-specific tuning, as described in the Methods section. This ensures a fair and consistent comparison across methods.
We note that there exist strain-resolved tools that assume prior knowledge of species identity and operate within a single-species context. As explicitly stated in the manuscript, such methods were not included in the comparison because MADRe is designed for realistic metagenomic scenarios where species composition is unknown and multiple species may be present simultaneously. Including single-species-restricted methods would therefore not provide a fair or meaningful comparison for the problem setting addressed in this study.
The evaluated comparative datasets used in this study span a broad range of strain divergence levels, including medium and large simulated datasets with moderate divergence, a dedicated high-similarity stress test, Zymo mock communities, and real metagenomic samples. Across these settings, MADRe demonstrates consistent performance trends relative to state-of-the-art tools.
To further characterize the performance limits of MADRe across combinations of strain similarity and coverage depth, we conducted additional controlled synthetic experiments (Supplementary File: Coverage and Similarity Experiment; Supplementary Table ST24), as noted in one of the previous responses. In these datasets, we simulated mixtures of three strains with varying ANI levels from E. coli, P. aeruginosa, S. aureus, and L. monocytogenes, and systematically varied the coverage of one strain (20x, 10x, 5x, and 3x). These experiments were designed to probe the sensitivity of MADRe under controlled divergence and abundance conditions. As stated in the answer to the previous reviewer, we demonstrated that coverage depth does not substantially affect the detection of the correct strain, rather, performance is primarily influenced by the presence of multiple highly similar strains in the reference database. When expected strains were not recovered, this was primarily associated with extremely high similarity (>99.9% ANI) to another genome in the database, which prevented reliable discrimination.
We have revised the Discussion to explicitly address these conclusions (lines: 773-791).
We trust that this expanded analysis addresses the reviewer’s comment. If further clarification of the requested scenarios is needed, we would be grateful for additional guidance.

3) when comparing with existing tools, improvements appear primarily in precision, while recall trade-offs are less emphasized. The authors should explicitly discuss precision-recall trade-offs and clarify in which biological scenarios MADRe is most advantageous.

→ answer:
We agree with the reviewer that precision-recall trade-offs should be discussed more explicitly. In this study, we intentionally emphasize precision because MADRe is designed to reduce false-positive detections, which are a major challenge in strain-level metagenomic classification, particularly when using large and redundant reference databases.
At the genome identification level, a strain is considered detected if at least one read is classified to it. Under this definition, MADRe exhibits lower recall but higher precision compared to other approaches (e.g., results in ST20). This behavior reflects MADRe’s identification strategy, which reduces false-positive strain detections at the cost of missing some low-support strains or sequences.
However, when read-level classification is considered, the picture is different. Although some strains may be identified by other methods but not by MADRe, the number of reads originating from such strains is typically low. In addition, when a strain is not retained during database reduction, its reads are often reassigned to the most similar reference in the reduced database. In such cases, read-level recall may remain largely unaffected, while strain-level precision can be influenced by the degree of similarity among reference genomes. For this reason, we placed greater emphasis on the precision metric. Furthermore, the number of false-negative reads is comparable between MADRe and other approaches, despite differences observed at the genome identification level.
In many evaluated datasets, the fraction of unclassified reads is low relative to the total number of reads, making genome-level recall less informative as a primary metric. Nevertheless, we report recall, precision, and F1 scores for all experiments, with detailed results provided in the Supplementary Tables (ST8 and ST15). Notably, for large simulated datasets, MADRe achieves comparable or improved recall while simultaneously improving precision, which is reflected in competitive or higher F1 scores.
We have revised the Discussion to explicitly clarify this precision-recall trade-off and to highlight that MADRe is particularly advantageous in applications where precision is prioritized, such as settings in which false-positive strain detection carries greater consequences than missing extremely low-abundance

organisms (lines: 760-772).

4) While database reduction is presented as efficient, the computational cost of assembly plus EM iterations is not deeply analyzed. The authors should include a concise runtime/memory comparison or at least a qualitative discussion of computational trade-offs.

→ answer:
We thank the reviewer for this comment. A detailed analysis of runtime and memory usage is already provided in the Supplementary Table (ST22), which reports CPU time and peak memory consumption for all major steps of the MADRe pipeline across two representative datasets, including database reduction. The database reduction stage comprises: (i) metagenome assembly, (ii) mapping assembled contigs to the full reference database, (iii) the HairSplitter step used to detect collapsed strain signal, and (iv) the reference reduction algorithm itself. Among these, the assembly step is the most computationally demanding, particularly for large datasets. In contrast, contig-to-database mapping and the HairSplitter-based processing are substantially less resource-intensive than mapping all reads to a large reference database, and the reference reduction algorithm itself has minimal computational overhead.
We have clarified this computational trade-off in the revised manuscript (lines:645-655).

5) The approach implicitly assumes that metagenome assembly is sufficiently accurate and representative. However, in highly complex or low-coverage samples, assembly could be fragmented or biased. The authors should add a clearer discussion on the sensitivity to assembler choice and parameters.

→ answer:
We thank the reviewer for this comment and agree that metagenome assembly quality can influence strain detection, particularly in highly complex or low-coverage samples.
MADRe is designed as a modular pipeline, allowing different assemblers and parameter settings to be used without modifying the core workflow. To assess sensitivity to assembler choice, we evaluated MADRe on large simulated datasets using Myloasm instead of metaFlye and metaMDBG. Overall performance trends were comparable, indicating that MADRe is not strongly dependent on a specific assembler. We observed complementary behavior, with Myloasm performing better for low-abundance strains and metaFlye performing slightly better for highly abundant strains. Based on these results, Myloasm is included as an optional component within the MADRe pipeline.
We note that strain-level assemblers were not considered, as they typically produce shorter and more fragmented contigs. MADRe's database reduction algorithm benefits from longer genomic context.
Regarding parameter sensitivity, all assemblers were executed using default or recommended settings. While different parameter choices may influence assembly quality, exploring extensive parameter tuning would substantially expand the scope of this study. We clarify this in the revised Discussion (lines:812-827).

REFERENCES:

[1] Federhen, Scott, et al. "Toward richer metadata for microbial sequences: replacing strain-level NCBI taxonomy taxids with BioProject, BioSample and Assembly records." Standards in genomic sciences 9.3 (2014): 1275-1277.
[2] Kazantseva, Ekaterina, et al. "Strainy: phasing and assembly of strain haplotypes from long-read metagenome sequencing." Nature Methods 21.11 (2024): 2034-2043.

Editor Comments:

GigaScience has also published a number of relevant papers on strain-level metagenomic classification, machine learning-based host prediction, and pipeline development for microbiome analysis. Citing these papers would help contextualize your methodological contributions and strengthen the discussion of your results within the current literature landscape.

1. Yang Y, Dufault-Thompson K, Yan W, Cai T, Xie L, Jiang X. Large-scale genomic survey with deep learning-based method reveals strain-level phage specificity determinants. GigaScience. 2024;13:giae017.

https://doi.org/10.1093/gigascience/giae017

2. Chen G, Jiang J, Sun Y. RNAVirHost: a machine learning–based method for predicting hosts of RNA viruses through viral genomes. GigaScience. 2024;13:giae059. https://doi.org/10.1093/gigascience/giae059

3. Arikan M, Muth T. gNOMO2: a comprehensive and modular pipeline for integrated multi-omics analyses of microbiomes. GigaScience. 2024;13:giae038. https://doi.org/10.1093/gigascience/giae038

4. Roach MJ, Beecroft SJ, Mihindukulasuriya KA, et al. Hecatomb: an integrated software platform for viral metagenomics. GigaScience. 2024;13:giae020. https://doi.org/10.1093/gigascience/giae020

5. Gao Y, Luo H, Lyu H, et al. Benchmarking short-read metagenomics tools for removing host contamination. GigaScience. 2025;14:giaf004. https://doi.org/10.1093/gigascience/giaf004

-> answer:
We thank the Editor for this helpful suggestion and for highlighting relevant recent publications in GigaScience. We agree that situating MADRe within the broader landscape of strain-level metagenomic and microbiome analysis methods strengthens the manuscript.
Accordingly, we have incorporated citations to Yang et al. (2024), Arikan and Muth (2024), and Gao et al. (2025), as these studies are directly relevant to strain-level genomic analysis, machine learning approaches applied to microbial genomics, and modular/benchmarking frameworks in metagenomic workflows.
We also carefully considered the studies by Chen et al. (2024) and Roach et al. (2024). However, these works focus primarily on RNA virus host prediction and viral metagenomics platforms, respectively. We believe that these studies fall outside the direct methodological scope of our work. For this reason, we did not incorporate them into the discussion to avoid overstretching the conceptual framing.
We appreciate the Editor's guidance in ensuring comprehensive contextualization within the journal's recent literature.

In addition, please ensure that all references are to the final, peer-reviewed published versions of articles. If you have cited any preprints (e.g., from bioRxiv) that have now been published in journals, please update those entries accordingly. This is an important step for the long-term archival stability and credibility of the reference list.

We have reviewed the entire reference list and updated all entries to cite the final peer-reviewed published versions where available, replacing preprint citations accordingly.

Editorial Manager®

Cookie Preference Center

We use cookies which are necessary to make our site work. We may also use additional cookies to analyze, improve and personalize our content and your digital experience. For more information, see our Cookie Policy.

You may choose not to allow some types of cookies. However, blocking some types may impact your experience of our site and the services we are able to offer. See the different category headings below to find out more or change your settings.

Allow all

Manage Consent Preferences

Strictly Necessary Cookies

Always active

These cookies are necessary for the website to function and cannot be switched off in our systems. They are usually only set in response to actions made by you which amount to a request for services, such as setting your privacy preferences, logging in or filling in forms. You can set your browser to block or alert you about these cookies, but some parts of the site will not then work.

Functional Cookies

These cookies enable the website to provide enhanced functionality and personalisation. They may be set by us or by third party providers whose services we have added to our pages. If you do not allow these cookies then some or all of these services may not function properly.

Performance Cookies

These cookies allow us to count visits and traffic sources so we can measure and improve the performance of our site. They help us to know which pages are the most and least popular and see how visitors move around the site.

Targeting Cookies

These cookies may be set through our site by our advertising partners. They may be used by those companies to build a profile of your interests and show you relevant adverts on other sites. If you do not allow these cookies, you will experience less targeted advertising.

Cookie List

Clear

Apply Cancel
Consent Leg.Interest

Confirm my choices

Power
