# Supplementary material for: MADRe: Strain-level metagenomic classification through assembly-driven database reduction
Source: Gigascience. 2026 Mar 23;15:giag030. doi: 10.1093/gigascience/giag030 (PMC13211987; doi:10.1093/gigascience/giag030)
Supplement: giag030_Reviewer_1_Report_original_submission — Reviewer 1 -- 11/19/2025 [file giag030_reviewer_1_report_original_submission.pdf]

Reviewer Report

Title: MADRe: Strain-Level Metagenomic Classification Through Assembly-Driven Database Reduction

Version: Original Submission **Date:** 11/19/2025

Reviewer name: Reviewer 1

Reviewer Comments to Author:

I have no significant concerns with the MADRe methodology, and the current datasets provide sufficient evidence of its strain-level performance. However, several issues still need to be addressed.The reponse states:"However, we observed a limitation when Centrifuger cannot confidently assign a read to a specific reference sequence (for example, when multiple chromosomes belong to the same strain). In such cases, it often classifies the read under the NCBI strain-level taxid, which in some instances is identical to the species-level taxid. This makes it impossible to directly and fairly compare those classifications with other tools that operate at the sequence level."Although I agree this issue may not substantially affect the overall conclusions, the current handling of strain-level evaluation for Centrifuger is not sufficiently rigorous. The underlying problem is that Centrifuger (and Kraken2) rely on nodes.dmp and names.dmp, where the lowest taxonomic rank is often species or subspecies. As a result, these tools cannot report strain-level abundances directly in their standard output.A more appropriate solution would be to assign custom, unique strain-level taxIDs for all reference genomes, allowing proper classification at the strain level. This approach has been discussed in https://github.com/mourisl/centrifuger/issues/18 and https://github.com/jenniferlu717/Bracken/issues/113. Additionally, Centrifuger has an extra program, centrifuger-quant, that uses the EM algorithm to estimate abundance. The read assignment results produced by Centrifuger do not apply the EM algorithm.In the similarity experiment, some strains exhibit extremely high similarity, which makes proportional read distribution practically impossible for MADRe. To better characterize the performance limits of MADRe for accurate strain classification and abundance estimation, I recommend including additional simple synthetic mixtures at different combinations of similarity and coverage depth. Because long reads vary widely in length, read counts alone can be misleading. I strongly encourage reporting strain abundances rather than raw read counts, as abundances are more relevant for downstream applications.Finally, the authors should clarify whether MADRe's limitations in detecting low-abundance strains (referring more to low coverage) is entirely determined by the performance of the assembly tool, or whether additional factors influence this limitation.In Figure 4, please specify the sequencing technology used for sim_high."calculated usin fastANI" â†’"calculated using fastANI".

Level of Interest

Please indicate how interesting you found the manuscript: Choose an item.

Quality of Written English

Please indicate the quality of language in the manuscript: Choose an item.

Declaration of Competing Interests

Please complete a declaration of competing interests, considering the following questions:

- Have you in the past five years received reimbursements, fees, funding, or salary from an organisation that may in any way gain or lose financially from the publication of this manuscript, either now or in the future?
- Do you hold any stocks or shares in an organisation that may in any way gain or lose financially from the publication of this manuscript, either now or in the future?
- Do you hold or are you currently applying for any patents relating to the content of the manuscript?
- Have you received reimbursements, fees, funding, or salary from an organization that holds or has applied for patents relating to the content of the manuscript?
- Do you have any other financial competing interests?
- Do you have any non-financial competing interests in relation to this paper?

If you can answer no to all of the above, write 'I declare that I have no competing interests' below. If your reply is yes to any, please give details below.

I declare that I have no competing interests

I agree to the open peer review policy of the journal. I understand that my name will be included on my report to the authors and, if the manuscript is accepted for publication, my named report including any attachments I upload will be posted on the website along with the authors' responses. I agree for my report to be made available under an Open Access Creative Commons CC-BY license (http://creativecommons.org/licenses/by/4.0/). I understand that any comments which I do not wish to be included in my named report can be included as confidential comments to the editors, which will not be published.

Choose an item.

To further support our reviewers, we have joined with Publons, where you can gain additional credit to further highlight your hard work (see: https://publons.com/journal/530/gigascience). On publication of this paper, your review will be automatically added to Publons, you can then choose whether or not to claim your Publons credit. I understand this statement.

Yes Choose an item.
