# Supplementary material for: MADRe: Strain-level metagenomic classification through assembly-driven database reduction
Source: Gigascience. 2026 Mar 23;15:giag030. doi: 10.1093/gigascience/giag030 (PMC13211987; doi:10.1093/gigascience/giag030)
Supplement: giag030_Reviewer_2_Report_original_submission — Reviewer 2 -- 1/23/2026 [file giag030_reviewer_2_report_original_submission.pdf]

Reviewer Report

Title: MADRe: Strain-Level Metagenomic Classification Through Assembly-Driven Database Reduction

Version: Original Submission **Date:** 1/23/2026

Reviewer name: Reviewer 2

Reviewer Comments to Author:

This manuscript presents MADRe, a modular pipeline for strain-level metagenomic classification from long-read data, emphasizing an assembly-driven database reduction strategy coupled with probabilistic reassignment. The work is methodologically sound and well aligned with the scope of GigaScience. However, the study can be benefit from the following revisions:1, the study's main contribution is engineering and integration, rather than a fundamentally new statistical model. The authors thus should explicitly mention this in the Abstract as well as the Discussion part.2, although comparisons are reasonable, the manuscript could do more to clarify how MADRe compares against state-of-the-art strain-resolved tools under identical parameter tuning, and whether performance gains are consistent across different strain divergence levels.3, when comparing with existing tools, improvements appear primarily in precision, while recall trade-offs are less emphasized. The authors should explicitly discuss precision-recall trade-offs and clarify in which biological scenarios MADRe is most advantageous.4, While database reduction is presented as efficient, the computational cost of assembly plus EM iterations is not deeply analyzed. The authors should include a concise runtime/memory comparison or at least a qualitative discussion of computational trade-offs.5, The approach implicitly assumes that metagenome assembly is sufficiently accurate and representative. However, in highly complex or low-coverage samples, assembly could be fragmented or biased. The authors should add a clearer discussion on the sensitivity to assembler choice and parameters.

Level of Interest

Please indicate how interesting you found the manuscript: Choose an item.

Quality of Written English

Please indicate the quality of language in the manuscript: Choose an item.

Declaration of Competing Interests

Please complete a declaration of competing interests, considering the following questions:

- Have you in the past five years received reimbursements, fees, funding, or salary from an organisation that may in any way gain or lose financially from the publication of this manuscript, either now or in the future?

- Do you hold any stocks or shares in an organisation that may in any way gain or lose financially from the publication of this manuscript, either now or in the future?
- Do you hold or are you currently applying for any patents relating to the content of the manuscript?
- Have you received reimbursements, fees, funding, or salary from an organization that holds or has applied for patents relating to the content of the manuscript?
- Do you have any other financial competing interests?
- Do you have any non-financial competing interests in relation to this paper?

If you can answer no to all of the above, write 'I declare that I have no competing interests' below. If your reply is yes to any, please give details below.

I declare that I have no competing interests

I agree to the open peer review policy of the journal. I understand that my name will be included on my report to the authors and, if the manuscript is accepted for publication, my named report including any attachments I upload will be posted on the website along with the authors' responses. I agree for my report to be made available under an Open Access Creative Commons CC-BY license (http://creativecommons.org/licenses/by/4.0/). I understand that any comments which I do not wish to be included in my named report can be included as confidential comments to the editors, which will not be published.

Choose an item.

To further support our reviewers, we have joined with Publons, where you can gain additional credit to further highlight your hard work (see: https://publons.com/journal/530/gigascience). On publication of this paper, your review will be automatically added to Publons, you can then choose whether or not to claim your Publons credit. I understand this statement.

Yes Choose an item.
